# Targeting Neuroinflammation and Cognitive Decline: First-in-Class Dual Butyrylcholinesterase and p38α Mitogen-Activated Protein Kinase Inhibitors

**DOI:** 10.1021/acs.jmedchem.5c00933

**Published:** 2025-08-08

**Authors:** Svit Ferjančič Benetik, Matic Proj, Damijan Knez, Urban Košak, Anže Meden, Katja Krajšek, Anja Pišlar, Selena Horvat, Urban Švajger, Nataša Tešić, Lenka Pulkrabkova, Ondrej Soukup, Adam Skarka, Rudolf Andrys, Xavier Brazzolotto, Alexandre Igert, Florian Nachon, Jose Dias, Jan Detka, Joanna Gdula-Argasińska, Elżbieta Wyska, Małgorzata Szafarz, Aleksandra Manik, Natalia Płachtij, Kamil Musílek, Kinga Sałat, Aleš Obreza, Stanislav Gobec

**Affiliations:** † Faculty of Pharmacy, Department of Pharmaceutical Chemistry, University of Ljubljana, 1000 Ljubljana, Slovenia; ‡ Department for Therapeutic Services, Blood Transfusion Center of Slovenia, 1000 Ljubljana, Slovenia; § Biomedical Research Centre, University Hospital in Hradec Kralove, 500 05 Hradec Kralove, Czech Republic; ∥ Faculty of Science, Department of Chemistry, University of Hradec Kralove, 500 03 Hradec Kralove, Czech Republic; ⊥ Département de Toxicologie et Risques Chimiques, Institut de Recherche Biomédicale des Armées, 91220 Paris, France; # Department of Pharmacodynamics, Chair of Pharmacodynamics, Faculty of Pharmacy, 49573 Jagiellonian University Medical College, 9 Medyczna St., 30-688 Krakow, Poland; ∇ Department of Radioligands, Faculty of Pharmacy, 49573 Jagiellonian University Medical College, 9 Medyczna St., 30-688 Krakow, Poland; ○ Department of Pharmacokinetics and Physical Pharmacy, Jagiellonian University Medical College, 9 Medyczna St., 30-688 Kraków, Poland

## Abstract

The currently approved drugs for the treatment of Alzheimer’s disease (AD) fail to address its interconnected pathological processes. Inhibition of butyrylcholinesterase (BChE) and p38α mitogen-activated protein kinase (p38α MAPK) offers an innovative dual approach to mitigate two major drivers of neurodegeneration in AD: cholinergic deficit and neuroinflammation. Using structure-based drug design and a library of known p38α MAPK inhibitors, we developed first-in-class, selective dual BChE/p38α MAPK inhibitors with balanced activity against both targets. The X-ray crystal structures of the two most promising molecules bound to both enzymes were solved. Those ligands effectively reduced the production of proinflammatory markers in vitro and ex vivo in phytohemagglutinin/lipopolysaccharide neuroinflammation models. Remarkably, these compounds also significantly improved cognition in scopolamine- and lipopolysaccharide-induced models of cognitive dysfunction in mice. Because our dual-acting inhibitors target both the symptoms and the underlying neuropathology, they offer an innovative and comprehensive strategy to combat AD.

## Graphical abstract

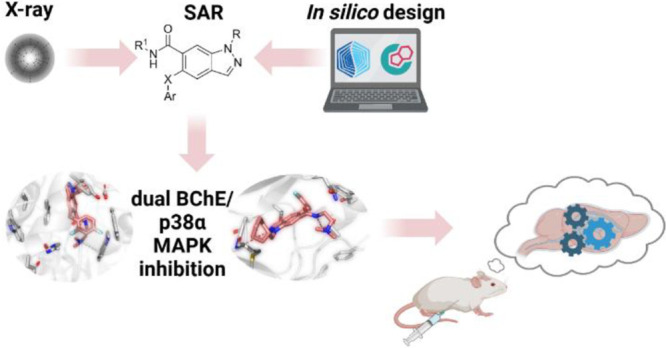

## 1 Introduction

Neurodegenerative diseases (NDDs) are characterized by an etiologically unexplained progressive structural and functional deterioration of neuronal networks that impair communication, memory, cognition, and behavior.^1^ The multifactorial nature of NDDs makes it practically impossible to intervene in the deleterious pathophysiological changes of the disease by single-targeting approaches. Polytherapy and multitarget directed ligands (MTDLs) have thus emerged as a promising strategy to tackle neurodegeneration.^2^ However, polytherapy often presents challenges, particularly in elderly patients, due to unpredictable pharmacokinetics, leading to drug–drug interactions.^3,4^ On the other hand, MTDLs retain the benefits of polytherapy while minimizing drug–drug interactions and associated side effects, ultimately improving patient adherence.^3,5^ Having similar potencies against all modulated targets, MTDLs can achieve a synergistic effect.^2^ This not only reduces the risk of high drug dose-related toxicities but also surpasses single-target approaches by offering greater efficacy against a plethora of neuropathological hallmarks of NDDs.

This approach may be particularly effective in Alzheimer’s disease (AD), the most common NDD, characterized by synaptic dysfunction, impaired proteostasis, and chronic inflammation. The loss of basal forebrain cholinergic neurons projecting to the hippocampus and cortex, along with the discovery that cholinesterase inhibitors (ChEIs) improve cognitive abilities in AD patients, led to the development of the first pharmacologic treatment for AD-ChEIs: donepezil, galantamine, and rivastigmine.^6–8^ Aiming to not only alleviate symptoms but also alter the underlying disease processes, research has since shifted toward extra- and intracellular pathological aggregates of fibrillar proteins–amyloid β (Aβ) and tau protein, respectively.^9,10^

Despite extensive research on both targets, which led to the recent FDA approval of monoclonal antibodies against Aβ species (aducanumab, lecanemab, and donanemab),^11–13^ many therapeutics targeting tau and Aβ have failed in clinical trials. Anti-Aβ antibodies have been associated with amyloid-related imaging abnormalities, including edema, effusions, cerebral microhemorrhages, and superficial siderosis.^14^ Furthermore, aducanumab has already been discontinued by its manufacturer.^15–17^ As a result, researchers are continuing to explore novel MTDL combinations. Most commonly, ChEIs are combined with compounds targeting various enzymes involved in tau phosphorylation, oxidative stress, or Aβ processing (Figure 1).^18–24^ The compounds presented in Figure 1 were designed not only to modulate biochemical markers of the disease but also to enhance patients’ cognitive function. Dual target engagement was accomplished by integrating consensus structural features for effective binding into a single molecule. Although molecules A–D exhibit considerable promise, none comprehensively address all the key pathological processes underlying AD progression. This highlights the ongoing need to identify novel enzyme targets beyond ChE inhibition in order to effectively address all major hallmarks of this neurodegenerative disorder.

**Figure 1:**
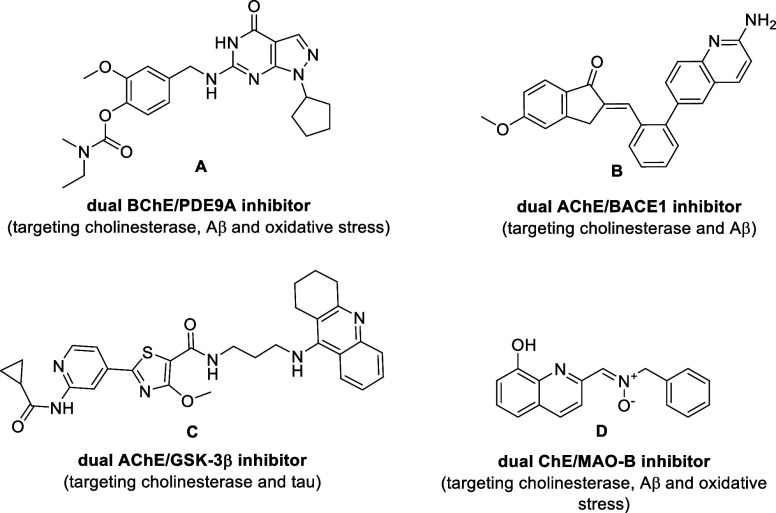
Examples of MTDLs acting as dual inhibitors of ChE and enzymes involved in tau phosphorylation, oxidative stress, or Aβ processing. These include phosphodiesterase 9A (PDE9A, compound **A** ^23^), β-secretase 1 (BACE-1, compound **B** ^24^), glycogen synthase kinase-3β (GSK-3β, compound **C** ^22^), or monoamine oxidase-B (MAO-B, compound **D** ^21^).

An auspicious target for treating AD is a microglial p38α mitogen-activated protein kinase (p38α MAPK), which is activated by soluble oligomeric Aβ.^25^ Activated microglia increase the production of mature IL-1β,^26^ which augments tau phosphorylation and subsequent tangle formation via p38α MAPK-mediated hyperphosphorylation of tau protein at Thr50, Thr69, and Thr181.^27^ p38α MAPK also prevents β-secretase degradation, leading to increased Aβ production.^28^ In this way, the relentless neuroinflammatory cycle perpetuates itself, with p38α MAPK serving as a key enzyme in its amplification. As a direct consequence of these neuroinflammatory processes, the growing number of oligomeric Aβ plaques provokes an increase in matrix metalloproteinase-9 expression, which impairs neurotrophic growth factor synthesis, leading to a decrease in vesicular acetylcholine (ACh) transporters and thus a decline in cholinergic signaling.^29,30^ While acetylcholinesterase (AChE) activity decreases even by 45% in AD, the activity of butyrylcholinesterase (BChE) increases by as much as 90%, particularly in the hippocampus and temporal cortex. Peripheral parasympathetic side effects typical of AChE inhibitors are not observed with BChE inhibitors.^31^ Additionally, BChE inhibitors have been shown to reduce Aβ40 and Aβ42 levels in transgenic mice overexpressing human Aβ.^32^ These findings imply that BChE inhibition may be advantageous over AChE and mixed ChE inhibition. Although recent anti-AD drugs in clinical trials successfully modulate aberrant biochemical signaling pathways, they still fail to ameliorate cognitive decline in patients, which requires ChEI-mediated restoration of pre- and postsynaptic cholinergic connectivity. Meanwhile, p38α-MAPK plays a central role in several AD hypotheses, particularly neuroinflammation, which is a major contributor to impaired cholinergic signaling. To date, no studies have shown that simultaneous inhibition of these two enzymes has synergistic effects in the pathophysiology of AD. However, some of us have recently suggested^33^ that in vivo findings on separate targets together with the in silico feasibility of their binding sites present strong evidence that dual BChE/p38α MAPK inhibition could provide a novel approach to address the complex interplay of aberrant signaling pathways in AD.

The combination of two pharmacophoric structural scaffolds in an MTDL can be achieved through merging, contiguity, or simple fusion via a linker.^34^ Type I p38α MAPK inhibitors share consensus structural features (Figure 2): a six-membered heterocycle with a heteroatom (oxygen or nitrogen) that forms a hydrogen bond with Met109 amide in the p38α MAPK hinge region. Additionally, an aromatic fragment occupies the hydrophobic region I (HRI) (Figure 2, wheat sticks, left),^35^ while a five-membered heterocycle engages in hydrogen bonding with the Asp168-Phe169-Gly170 (DFG) pocket. Molecular docking of type I p38α MAPK inhibitors into the active site of hBChE^36^ revealed that they are able to occupy all primary binding pockets. The six-membered heterocycle resides in the acyl-binding pocket, the aromatic ring extends toward the peripheral anionic site (PAS), and the five-membered heterocycle—when substituted with a basic functional group—can interact with the choline-binding pocket (Figure 2; right). Based on these structural insights, our working hypothesis employed a combined pharmacophore approach starting from known scaffolds of type I p38α MAPK inhibitors^33^—the same strategy used for all currently known MTDLs targeting p38α MAPK together with another enzyme. Notable examples include SB202190 and SP600125, which act as dual p38α MAPK/JNK inhibitors,^37^ and pexmetinib, a dual p38α MAPK/Tie-2 inhibitor.^38^ While these ligands are primarily being investigated for cancer treatment, Koch and colleagues developed low-nanomolar dual p38α MAPK/GSK-3β inhibitors with potential to target neuroinflammatory processes in AD.^39^ Neuroinflammation is also a major hallmark of Lewy body dementia, for which neflamapimod, a selective p38α/β MAPK inhibitor, is currently being evaluated in phase IIb clinical trials. Building on this, Bolognesi and colleagues have successfully integrated a propargylamine moiety into the neflamapimod scaffold, resulting in a dual p38α MAPK/MAO-B ligand with neuroprotective properties in neuronal cells under toxic insult.^40^ Following these innovative approaches, we present here a new series of p38α MAPK targeting hybrids developed for neurodegenerative diseases.

**Figure 2:**
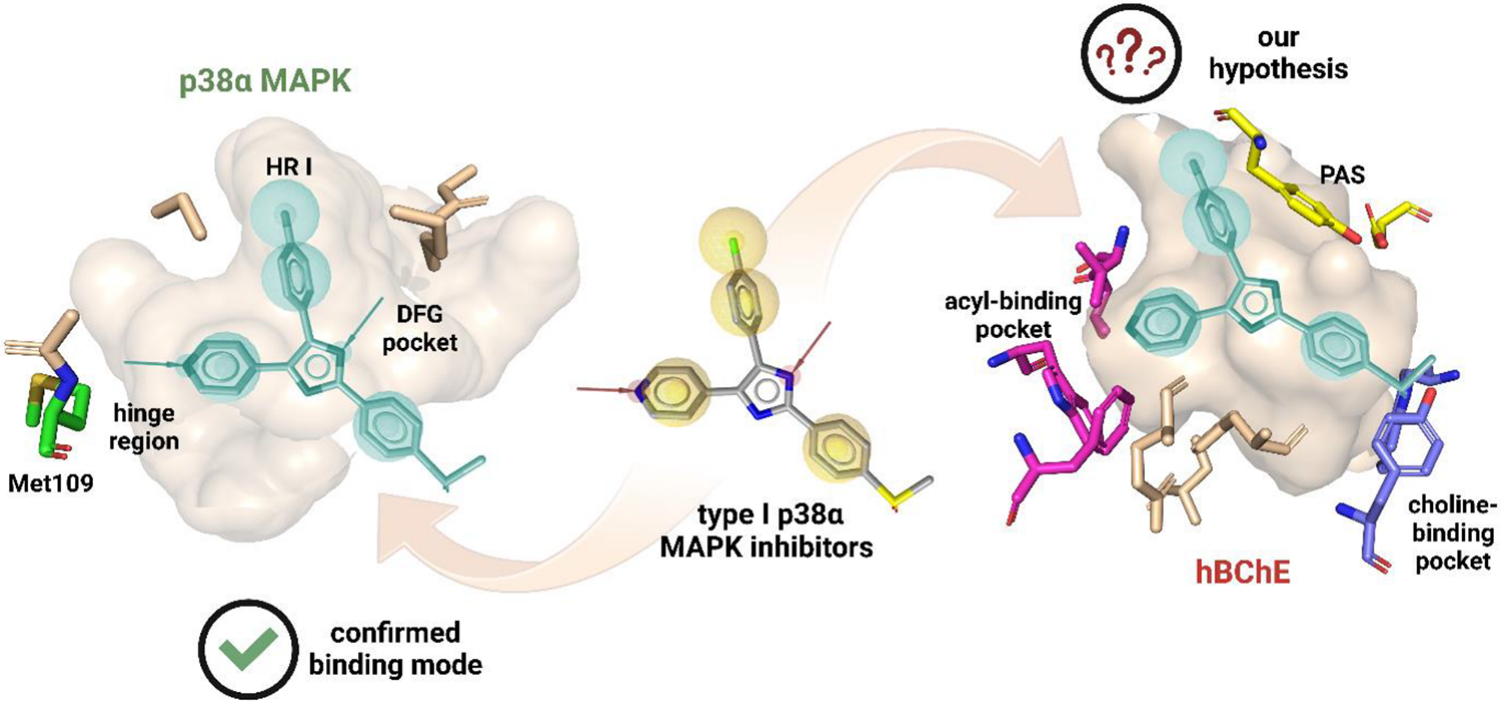
Proposed hypothesis for the development of a dual hBChE/p38α MAPK inhibitor starting from type I p38α MAPK inhibitors. Wheat surfaces show enzyme’s active sites. The acyl-binding pocket of hBChE is shown in magenta, while the choline-binding pocket and peripheral anionic site (PAS) are shown in violet and yellow, respectively. A type I p38α MAPK inhibitor is shown as a pharmacophore model of an exemplary ligand. The PDB structures used to generate the image are 1A9U (human p38α MAPK) and 6QAA (hBChE).

In this work, we describe the design, synthesis, and comprehensive biological evaluation of first-in-class MTDLs targeting both BChE and p38α MAPK. After synthesizing the novel dual BChE/p38α MAPK inhibitors, we obtained X-ray crystal structures of the most promising molecules bound to both enzymes and subjected them to in vitro, in vivo, and ex vivo assays, where they demonstrated procognitive and anti-inflammatory effects.

## 2 Results and Discussion

### 2.1 Discovery and Optimization of the Dual hBChE/p38α MAPK Hit Inhibitor

To test our working hypothesis for developing dual hBChE/p38α MAPK inhibitors, we first used an in silico-based workflow to identify hit compounds. Briefly, 172 commercially available p38α MAPK inhibitors were extracted from the ChEMBL and PDB databases and docked to the active site of hBChE. The hits obtained were clustered and visually inspected for occupancy of the hBChE’s acyl-binding pocket and the presence of a cation−π interaction with Trp82 in the choline-binding pocket. Eight structurally diverse compounds were acquired and evaluated in vitro for activity against recombinant hBChE and recombinant human p38α MAPK using the modified Ellman’s method^41^ and the ADP-Glo assay,^42^ respectively (see the Supporting Information for details). ARRY-371797, an orally bioavailable p38α MAPK inhibitor developed by Pfizer Inc.^43^ (Figure 3), with IC_50_ values of 12.0 μM and 0.13 μM for hBChE and p38α MAPK, respectively, was the only compound with the desired inhibitory profile (Table S1).

**Figure 3:**
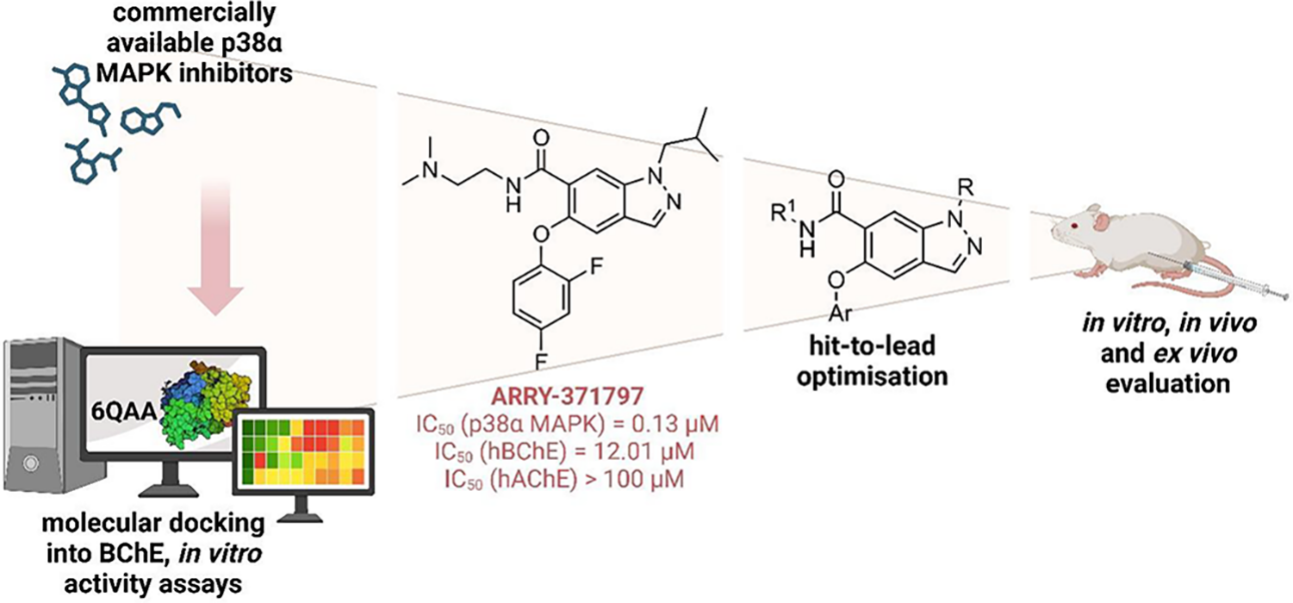
Discovery and optimization of hit compound ARRY-371797.

Docking and molecular dynamics (MD) simulations were then used to predict the putative binding modes of ARRY-371797 in hBChE and p38α MAPK active sites. For hBChE, docking placed ARRY-371797’s 2,4-difluorophenoxy moiety in the acyl-binding pocket, *N*,*N*-dimethylethyl moiety pointing toward the oxyanion hole, and the isobutyl chain toward Trp82 and Trp430 of the choline-binding pocket (Figure 4A). Meanwhile, the MD hinted at the possible π–π interaction of the positively charged *N*,*N*-dimethylethyl moiety with Trp82 in the choline-binding pocket (Figure 4C). For p38α MAPK, docking predicted a key hydrogen bond of the *N* ^2^-indazole nitrogen of ARRY-371797 with Met109 of the p38α MAPK hinge region, the occupancy of HRI by the 2,4-difluorophenoxy group, and of hydrophobic region II (HRII) by the isobutyl group (Figure 4B). The *N*,*N*-dimethylethyl moiety was oriented toward the solvent and formed interactions with Asp168 in the DFG pocket. In MD, however, the *N*,*N*-dimethylethylamide formed hydrogen bonds via water bridges with Asp168 and Val30, and a cation−π interaction with Tyr35 (Figure 4D).

**Figure 4:**
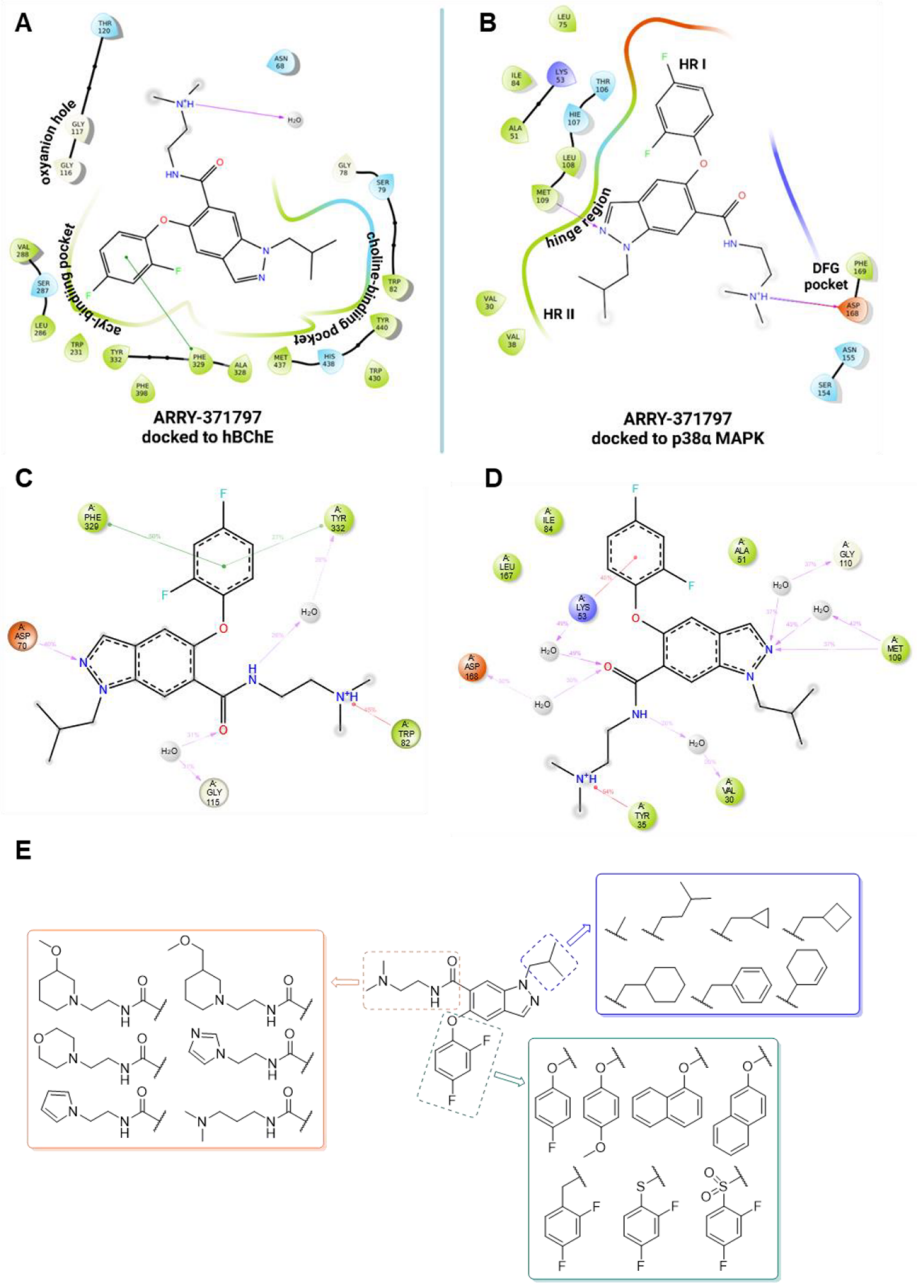
Most frequent docking poses of the hit compound ARRY-371797 in hBChE (A) and p38α MAPK (B). MD simulations of the binding of the hit compound ARRY-371797 in hBChE (C) and p38α MAPK (D). An overview of all modifications of the hit compound ARRY-371797 during the hit optimization phase (E).

Therefore, the hit compound ARRY-371797 offered three sites amenable to modifications: the isobutyl group, the 2,4-difluorophenoxy group, and the amide moiety. We replaced the 2,4-difluorobenzene group with bulkier aromatic rings or introduced benzyl and thiophenyl moieties to evaluate how this affects the binding to the acyl-binding pocket of hBChE and the occupancy of the HRI of p38α MAPK. Different alkyl substituents were introduced at the *N* ^1^ and *N* ^2^ nitrogen atoms of the indazole to probe how the occupancy of hydrophobic region II (HRII) in p38α MAPK affects the structure–activity relationships (SARs) of the inhibitors. Finally, tertiary amines of different lengths and nitrogen heterocycles were introduced into the amide group to explore the interactions with the DFG pocket of p38α MAPK and the PAS of hBChE (Figure 4E).

### 2.2 Synthesis and In Vitro Evaluation of New BChE/p38α MAPK Dual Inhibitors

The general synthetic pathway for most ARRY-371797 analogues consists of two converging routes (Schemes 1 and S1).

**Scheme 1:**
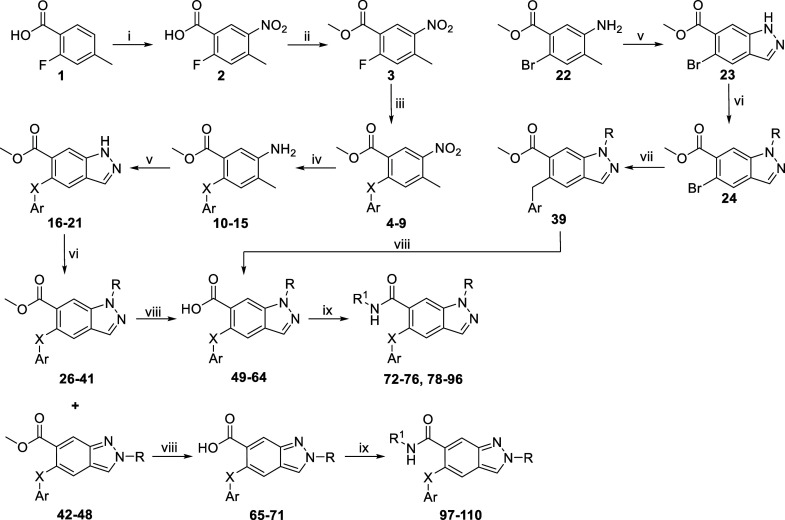
(i) HNO_3_, H_2_SO_4_, 0 °C; (ii) MeI, DIPEA, DMF, r.t.; (iii) Cs_2_CO_3_, 2,4-Difluorophenol, DMF, r.t. or K_2_CO_3_, 2,4-Difluorothiophenol, DMSO, 150 °C, MW; (iv) H_2_ (1 atm), 10% Pd/C, EtOH, r.t.; (v) CH_3_COOH, CH_3_COO^–^Na^+^, *tert*-Butyl Nitrite, CHCl_3_, r.t.; (vi) K_2_CO_3_, Corresponding Alkyl Bromide, DMF, 60 °C; (vii) (1) 2,4-Difluorobenzyl Bromide, Zinc Dust, TMSCl, 1,2-Dibromoethane, THF, 0 °C to r.t.; (2) **24**, Pd­(PPh_3_)_4_, THF, 60 °C; (viii) (1) 1 M LiOH­(aq), THF, reflux; (2) 1 M HCl­(aq); (ix) Et_3_N, TBTU, Corresponding Amine, DCM, 0 °C to r.t

Briefly, after nitration of compound **1** using HNO_3_ in H_2_SO_4_, nitrobenzoic acid **2** was protected by forming a methyl ester using MeI. Compounds **4–9** were then obtained by nucleophilic aromatic substitution using Cs_2_CO_3_ in anhydrous DMF at 60 °C for phenols, and K_2_CO_3_ in anhydrous DMSO in a microwave reactor at 150 °C for thiophenols. Following reduction of the nitro group by catalytic hydrogenation, indazoles **16–21** and **23** were synthesized by ring closure from the substituted *o*-toluidine via the *N*-nitroso intermediate.^44^ Compound **39** was obtained by Negishi coupling of an aryl bromide **24** with an in situ-prepared 2,4-difluorobenzylzinc bromide. Compounds **16–21** and **24** were alkylated with the corresponding alkyl bromides in an S_N_2 reaction using K_2_CO_3_ as base, yielding a mixture of *N* ^1^- and *N* ^2^-alkylated indazoles **26–41** and **42–48**, which were separated by column chromatography. After hydrolysis of the carboxylic acid esters in 1 M NaOH­(aq), amides **72–96** and **97–109** were prepared using TBTU as coupling reagent.

All final compounds were evaluated for their in vitro enzyme inhibitory activity on hAChE, hBChE, and human p38α MAPK (Tables 1, S2, and S3). Ligands with a residual enzymatic activity (RA) of more than 50% at a concentration of 100 μM were classified as inactive. The most interesting compounds are listed in Table 1 (the others can be found in Tables S2 and S3).

**Table 1:**
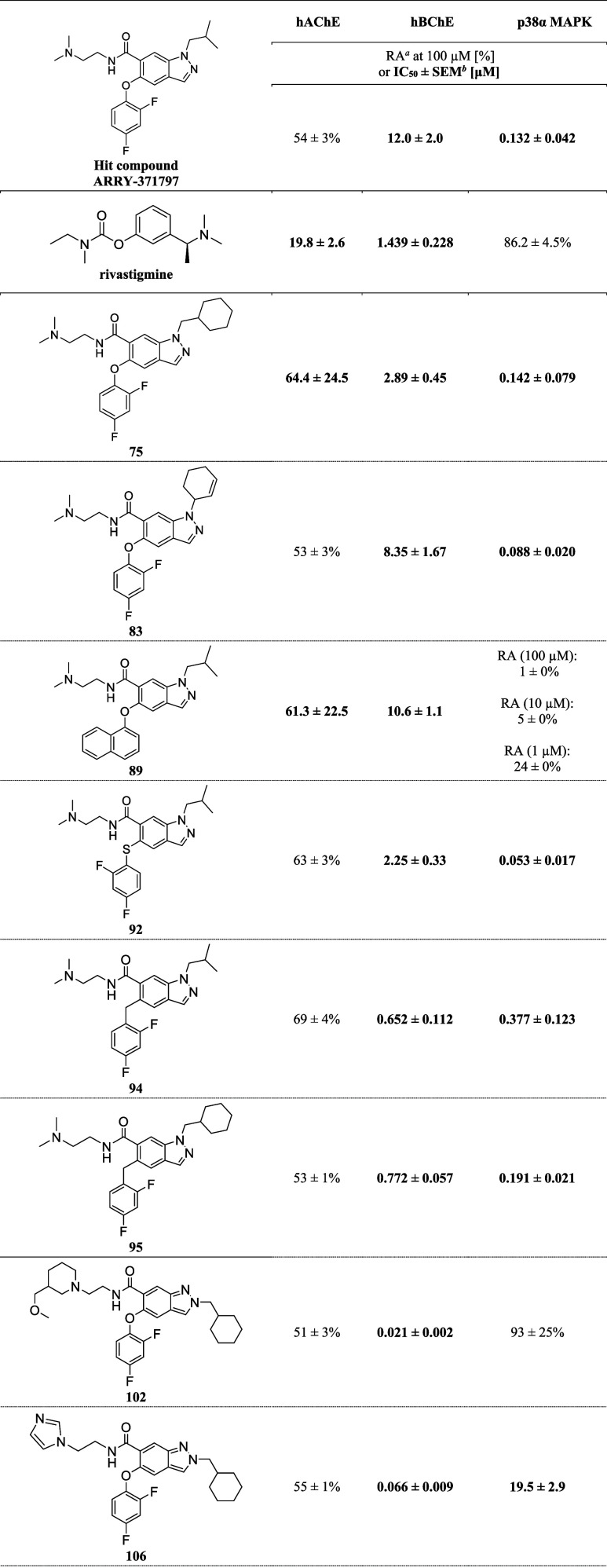
Structures and In Vitro hAChE, hBChE, and p38α MAPK Inhibitory Potencies (RA or IC_50_ Values) of Rivastigmine, the Parent Hit Compound ARRY-371797, and Its Most Promising Derivatives

a SEM—standard error of the mean, data are the average of two independent experiments, each performed in triplicate.

b RA—residual activity (mean ± standard deviation of one independent experiment performed in triplicate).

### 2.3 Kinase Selectivity, Structure–Activity Relationship, and Crystal Structures of Compounds 94 and 95

Compounds **94** and **95** showed balanced submicromolar inhibitory potency against hBChE and p38α MAPK, while they did not inhibit AChE at a concentration of 100 μM (Table 1), which was also one of the main objectives to diminish potential side effects. Second, these two compounds were analyzed for hBChE inhibition in the SH-SY5Y human neuroblastoma cell line,^45^ where they both outperformed the control ethopropazine at both 10 and 1 μM concentrations (Figure S1). Third, to further assess their suitability for in vivo evaluation, both compound **94** and compound **95** (at concentrations of 10 and 1 μM) were tested for selectivity against a panel of 103 kinases using the KINOMEscan assay.^46^ 94 of them were part of a diversity kinase panel, while JNK2, JNK3, ERK2, LCK, MAPKAPK5, NLK, p38β, p38γ, and p38δ MAPK represented the most phylogenetically and structurally related kinases to p38α MAPK according to the human kinome. Compounds **94** and **95** showed high selectivity for p38α MAPK. The KINOMEscan assay showed that off-target inhibition was limited only to structurally related kinases (p38β MAPK, JNK1/2/3, and CDK3) and was absent at 1 μM, where only p38α MAPK was inhibited among the 103 kinases tested (Table S4).

The crystal structures of compounds **94** and **95** in complex with hBChE (PDB codes 9I5P and 9I5O, Figure 5A,C and Table S5) revealed identical binding modes of the two ligands. Even though these *N* ^1^-alkylated indazoles do not occupy the acyl-binding pocket, they still exhibit potent hBChE inhibition (Table 1). Here, the indazole moiety is oriented toward the entrance of the gorge; therefore, a change in the alkyl group does not drastically affect hBChE inhibition. The tertiary amine forms a cation−π interaction (distances 3.9 Å for compound **94** and 4.0 Å for compound **95**) with Trp82. Substitution of the *N*,*N*-dimethylethylamine with larger functional groups improves BChE inhibition, but only in the case of *N* ^2^-alkylated indazoles such as compound **102**, where both the acyl- and choline-binding pockets are sufficiently occupied. The 3-(methoxymethyl)­piperidine of **102** extends toward the catalytic triad where additional interactions are possible (PDB code 9I5Q, Figure S2). The 2,4-difluorobenzyl group lies above Trp82 and forms hydrophobic interactions with Ala328. However, the mean ring distances of 4.9 and 5.0 Å for **94** and **95**, respectively, to Trp430 indicate that there is no edge-to-face π–π stacking. Apparently, the benzyl group allows a suitable rotation and size of the compound to sufficiently occupy this part of the choline-binding pocket compared to the phenoxy analogue **75**.

**Figure 5:**
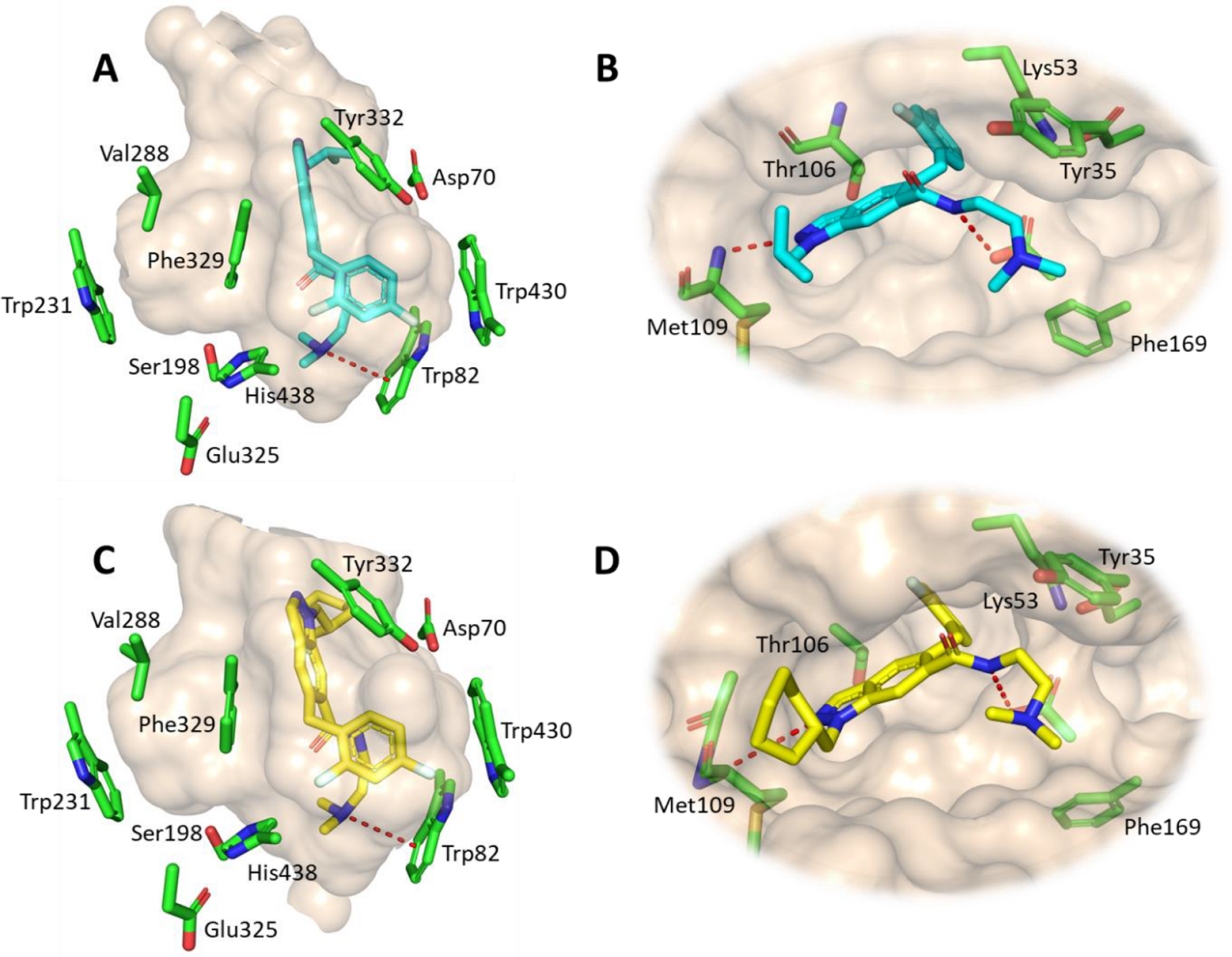
Crystal structures of compounds **94** and **95** in the hBChE active site (A and C, PDB codes 9I5P and 9I5O) and in the ATP-binding pocket of human p38α MAPK (B and D, PDB codes 9D7N and 9D75). Compounds **94** and **95** are shown as cyan and yellow sticks, respectively, and the enzymes are shown as wheat-colored outer surfaces with the most important amino acid residues as green sticks. Hydrogen bonds and cation−π interactions are presented as red dashed lines.

The binding of compounds **94** and **95** to human p38α MAPK (PDB codes 9D7N and 9D75, Figure 5B,D and Table S6) corresponds to the binding mode of known p38α MAPK inhibitors (PDB: 3GCP, 3QUE).^47,48^ The *N* ^2^ nitrogen of indazole forms a hydrogen bond with Met109 (3.1 Å for compound **94** and 5.1 Å for compound **95**). On the contrary, when the *N* ^2^ nitrogen is substituted, this hydrogen bonding is prevented, and kinase inhibition is weakened—this is evident in compounds **102** and **106**. The bulky isobutyl and cyclohexylmethyl groups occupy HR II, delineated by Val30, Ile108, Gly110, Ala111, and Asp112. The cyclohexenyl moiety introduction (compound **83**) to mimic the 2,6-dichloro group of neflamapimod,^49^ results in even better occupancy of HR II compared to the isobutyl fragment of ARRY-371797. The 2,4-difluorobenzene sits in HR I defined by Ala51, Lys53, Leu75, Ile84, Leu104, Thr106, and Leu167.^50^ The optimal distance of 7.9 Å between the hydrogen bond donor on the indazole core (i.e., the *N* ^2^ nitrogen of the indazole) and the 2,4-difluorobenzyl group allows exquisite selectivity toward the structurally and phylogenetically most related kinases.^51^ The introduction of larger fragments, such as 2,4-difluorothiophenyl (**92**) and naphthyl (**89–90**), retains or even improves p38α MAPK inhibition. The amide group is critical for effective inhibition of p38α MAPK as it acts as a hydrogen bond donor interacting with Asp168 of the DFG pocket (3.2 Å for compound **94** and 3.6 Å for compound **95**). The tertiary amine (i.e., the dimethylamine group) faces the solvent, which explains the retention of p38α MAPK inhibition when this functional group was not present (Table S2).

### 2.4 In Vitro Cytotoxicity, Pharmacokinetics, and Anti-Inflammatory Effects

Cytotoxicity studies on HepG2 liver cancer, SH-SY5Y neuroblastoma, and BV2 microglia cell lines using the MTS assay showed that compounds **94** and **95** did not affect cell viability at concentrations of 5 μM or less (Figure S4). Following these results, the neuroprotective effect against Aβ-treated SH-SY5Y cells and gliaprotective effect on the BV2 cell line for both compounds **94** and **95** were evaluated (Figures S5 and S6). To predict brain penetration of compounds **94** [calculated log *D*_7.4_ = 2.13] and **95** [calculated log *D*_7.4_ = 2.77], a well-established BBB Parallel Artificial Membrane Permeability Assay (BBB-PAMPA) was used.^52,53^ High passive permeability of the artificial membrane (effective permeability coefficient (*Pe*) values above 10 × 10^–6^ cm s^–1^) was determined for both compounds. The *Pe* values of 10.4 (**94**) and 13.2 × 10^–6^ cm s^–1^ (**95**) are comparable to those of the ChEIs approved for the treatment of AD-donepezil (*Pe* = 9.8 × 10^–6^ cm s^–1^) and rivastigmine (*Pe* = 12.6 × 10^–6^ cm s^–1^) (Table S7). Phase I metabolite stabilities using human liver microsomes showed that during a 5-h incubation with compounds **94** and **95**, the only significant metabolites were the mono*-N*-demethylated products. With only 18.7% of mono *N*-demethylated **94** and 7.9% of demethylated **95** compared to the parent compound, our compounds exhibit a high degree of metabolic stability (Table S8).

The anti-inflammatory effect of dual BChE/p38α MAPK inhibitors was investigated in an in vitro assay using peripheral blood mononuclear cells (PBMCs) stimulated with phyto­hemagglutinin (PHA) and neflamapimod as a positive control. There is increasing evidence that peripheral immune cells are actively involved in neuroinflammation.^54,55^ PHA is known to stimulate PBMCs by binding to glycosylated residues on T cell receptors (TCRs), which causes their cross-linking and subsequent triggering of calcium influx. Ca-dependent pathways then activate protein kinase C (PKC) and MAPK pathways, leading to increased cytokine production.^56,57^ Treatment with both compound **94**/**95** or neflamapimod significantly inhibited the production of pro-inflammatory cytokines IL-8, IL-1β, IL-6, IL-10, and TNF-α at concentrations of 1, 2.5, and 5 μM (Figure 6A–F), confirming the anti-inflammatory effect of p38α MAPK inhibition. At the same time, the production of the anti-inflammatory cytokine IL-12 was increased, albeit at a low level, when the PBMCs were treated with compounds **94** and **95**. This is consistent with several previous findings in which increased production of IL-12 after p38 MAPK inhibition was detected in various innate immune cells in the LPS model of neuroinflammation (PHA is frequently substituted for LPS to trigger the cellular innate immune response^58^). For example, Jarnicki et al. have shown that p38α MAPK inhibition can augment type 1, antitumor efficacy of dendritic cells (DCs) by increasing IL-12 production upon TLR stimulation.^59^ Similarly, Marriott et al. have shown that the p38α/β MAPK inhibitor SB203580 can significantly upregulate LPS-induced IL-12 production in human monocytes. The latter effect was shown to be IFN-γ-dependent, suggesting the need for T cell help and an interplay between IFN-γ signaling and p38α MAPK pathway in IL-12 regulation.^60^

**Figure 6:**
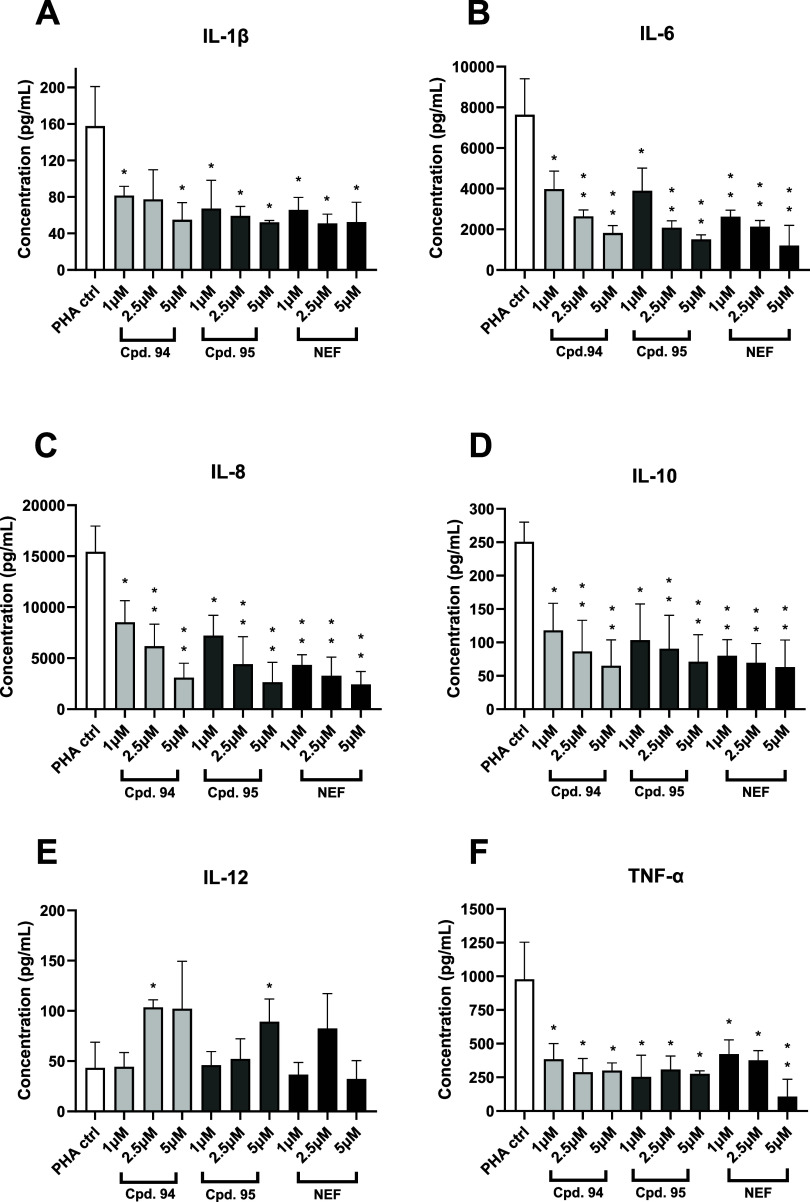
Effect on cytokine production by PBMC cells treated with PHA followed by compounds **94**, **95**, and neflamapimod (NEF) (A) IL-1β, (B) IL-6, (C) IL-8, (D) IL-10, (E) IL-12 and (F) TNF-α. PBMC cells were treated with compounds **94** and **95** (1 and 5 μM), and PHA (1 μg/mL). Results are shown as means (±SEM) of three independent experiments. Statistical analysis: Student’s *t* test Significance vs control treated only with PHA: * *p* < 0.05, ** *p* < 0.01; PHA = phytohemagglutinin.

### 2.5 In Vivo and Ex Vivo Studies

#### 2.5.1 Behavioral Tasks

To evaluate the procognitive properties of compounds **94** and **95** resulting from dual BChE/p38α MAPK inhibition, two mouse models of cognitive impairment were used, namely the scopolamine model,^61^ which is the model for the cholinergic deficit observed in AD, and the LPS model, which is the model for the neuroinflammation leading to cognitive dysfunction observed in various neurodegenerative diseases.^62,63^ In the scopolamine model, a scopolamine dose of 1 mg/kg was used for two reasons: (i) this dose leads to marked cognitive impairment as a result of antagonistic effects on muscarinic ACh receptors,^64^ and (ii) scopolamine at a dose of 2 mg/kg was also shown to increase pro-inflammatory cytokines and various inflammasome components in the hippocampus, prefrontal cortex and amygdala of mice. Such pro-inflammatory effects were not observed at a dose of 1 mg/kg.^65^

Three behavioral tests, i.e., the passive avoidance (PA), the Morris water maze (MWM), and the novel object recognition (NOR) task, were used to assess the effect of compounds **94** and **95** on the cognitive functions of adult male Albino Swiss (CD-1) and C57BL/6J mice. Since unimpaired locomotor activity is required to exclude false positives in these cognitive tasks, we evaluated motor coordination of animals using the rotarod and locomotor activity tests after treatment with compounds **94** and **95** (10 mg/kg dose; i.p. administration). As the compounds showed no motor adverse effects (SI, Figures S7 and S8), we continued with the PA, NOR, and MWM tasks. In the MWM task and ex vivo examination of the LPS-treated mice, the efficacy of compounds **94** and **95** was compared with that of the marketed ChEI rivastigmine. Although rivastigmine is a mixed ChEI and does not inhibit p38α MAPK, it was shown to attenuate LPS-stimulated proinflammatory cytokines in the BV2 cell line^66^ and reduce LPS-induced IL-2 levels when administered to mice (dose: 1 mg/kg, i.p.).^67^ These anti-inflammatory properties of rivastigmine could be explained by the cholinergic anti-inflammatory signaling pathway. Here, ACh from neuronal and non-neuronal sources exerts central and peripheral anti-inflammatory effects by reducing ROS production and cytokine release,^68^ and rivastigmine administration has been shown to be associated with this signaling pathway.^69^

To gain a deeper insight into the role of p38α MAPK in cognition and compare our compounds with selective p38α/β MAPK inhibitors in clinical trials, an additional experiment was performed to assess the procognitive potential of neflamapimod, which was evaluated in mouse models of cognitive decline caused by scopolamine or LPS.

#### 2.5.2 Scopolamine-Induced Amnesia

Mice were first subjected to the PA task. In this experiment, fear-motivated avoidance behavior due to an aversive stimulus (i.e., a foot shock) is used to assess long-term contextual memory based on negative reinforcement. In the PA task, cognition-enhancing drugs generally increase step-through latency, indicating improved memory retention as rodents require more time to re-enter the compartment associated with an aversive stimulus. This suggests enhanced consolidation or recall of the aversive experience.^70^ Compounds **94** and **95** were tested at two fixed doses (5 and 10 mg/kg). A statistically significant prolonged step-through latency was observed in the retention trial comparing scopolamine-treated control mice and mice treated with a combination of scopolamine and compound **94** at a 10 mg/kg dose and mice treated with scopolamine and compound **95** at a 10 mg/kg dose (Figure 7). Compounds **94** and **95** were not statistically significantly effective in this assay at the lower dose (5 mg/kg).

**Figure 7:**
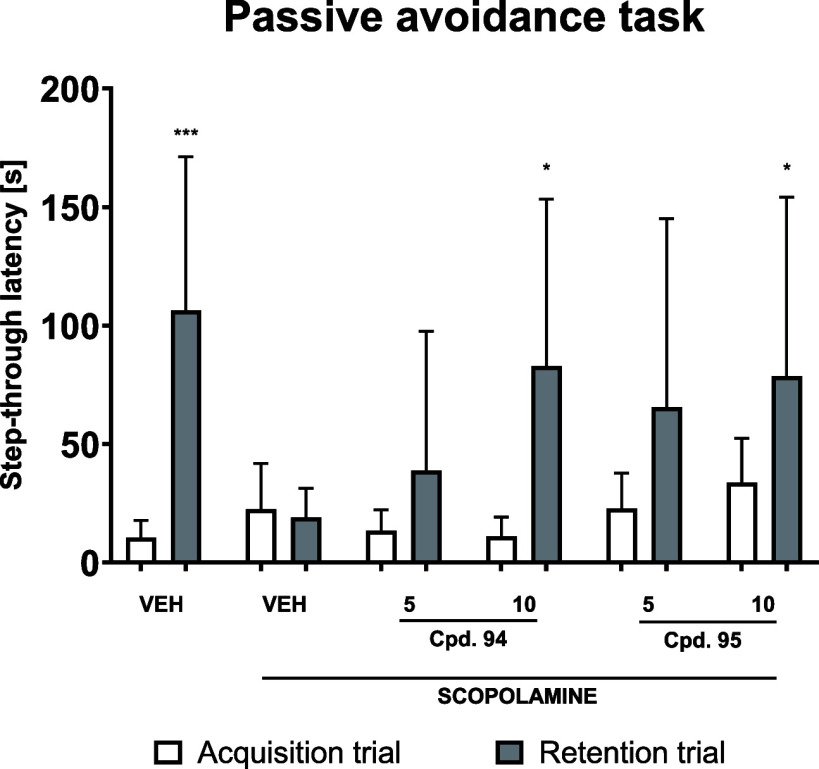
Effect of compounds **94** and **95** (doses: 5 and 10 mg/kg; i.p.) on scopolamine-induced cognitive deficits assessed in the PA task. Results are shown as mean step-through latency (±SEM) assessed in the acquisition trial and 24 h later (in the retention trial) for *n* = 9–10 mice. Statistical analysis: repeated measures ANOVA followed by Dunnett’s post hoc comparison. Significance vs scopolamine-treated control mice in the respective trial: * *p* < 0.05, *** *p* < 0.001. VEH = vehicle.

The procognitive efficacy of rivastigmine in the PA task carried out in scopolamine-induced memory-impaired mice was reported previously. This earlier study showed its procognitive activity in the PA task at doses of 1 and 2.5 mg/kg.^21^ The effect of neflamapimod on scopolamine-induced memory decline measured in the PA task is shown in Figure 8A. This experiment did not confirm memory-improving properties of neflamapimod at a wide dose range (1–30 mg/kg).

**Figure 8:**
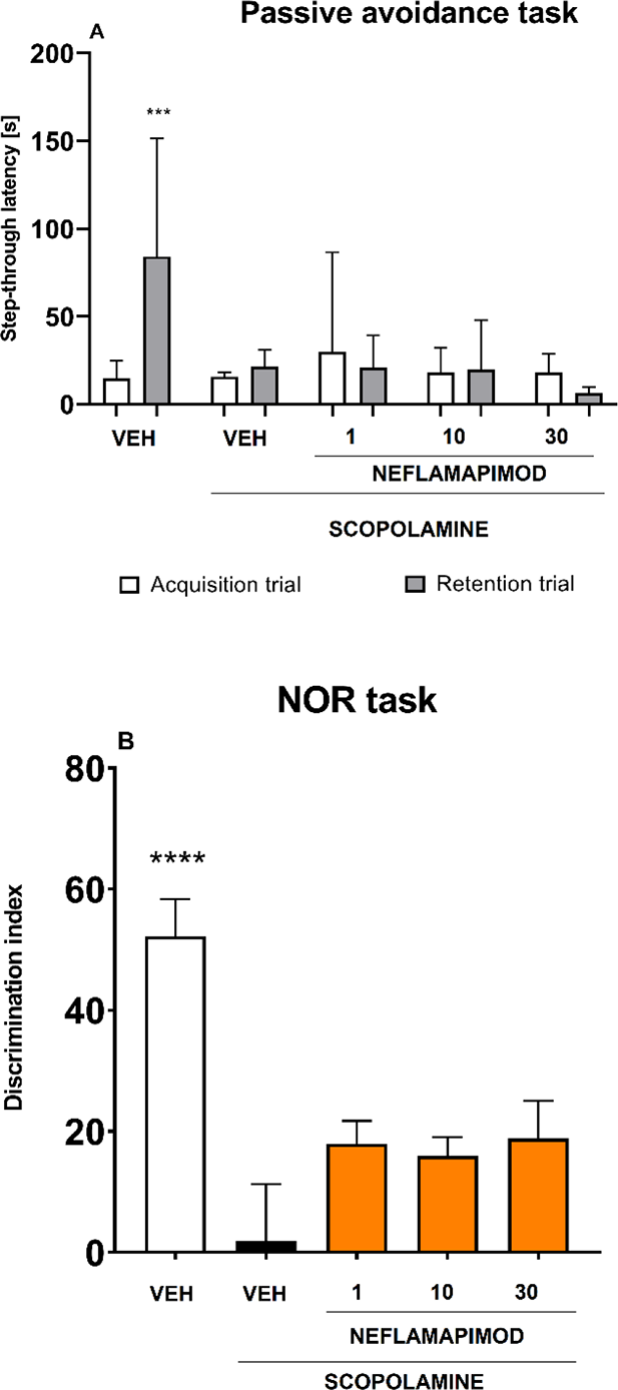
Effect of neflamapimod (doses: 1, 10, and 30 mg/kg; i.p.) on scopolamine-induced cognitive deficits assessed in the PA (A) and in the NOR task (B). Results are shown as mean step-through latency (±SEM) assessed in the acquisition trial and 24 h later (in the retention trial) for *n* = 9–10 mice (A) and results are shown as mean discrimination index (±SEM) for *n* = 8–10 mice (B). Statistical analysis: (A) repeated measures ANOVA, or (B) one-way ANOVA followed by Dunnett’s post hoc comparison. Significance vs scopolamine-treated control mice: *** *p* < 0.001, **** *p* < 0.0001. VEH = vehicle.

After the PA, the NOR task was performed. This task is based on the natural tendency of rodents to explore new objects in the absence of external reinforcement and measures working memory, attention, and preference for novelty in rodents.^71^ Cognition-enhancing drugs typically improve performance in the NOR test by increasing the discrimination index (DI), indicating better recognition and preference for the novel object over the familiar one.^72^ Indazoles **94** and **95** were again tested at two doses (5 and 10 mg/kg). Mice treated with scopolamine and indazole **94** or **95** (both at a dose of 10 mg/kg), respectively, showed a statistically significant preference for the novel object compared to mice treated with scopolamine alone. Similar to performance in the PA task, compounds **94** and **95** were statistically ineffective at a dose of 5 mg/kg (Figure 9). As shown previously,^21^ rivastigmine was effective in the PA and NOR tasks at the 1 and 2.5 mg/kg doses, but caused muscle tremor and transient motor coordination deficits in mice shortly after administration, already at the dose of 1 mg/kg. For this reason, it was not possible to compare compounds **94** and **95** with rivastigmine at the same dose (i.e., 10 mg/kg).

**Figure 9:**
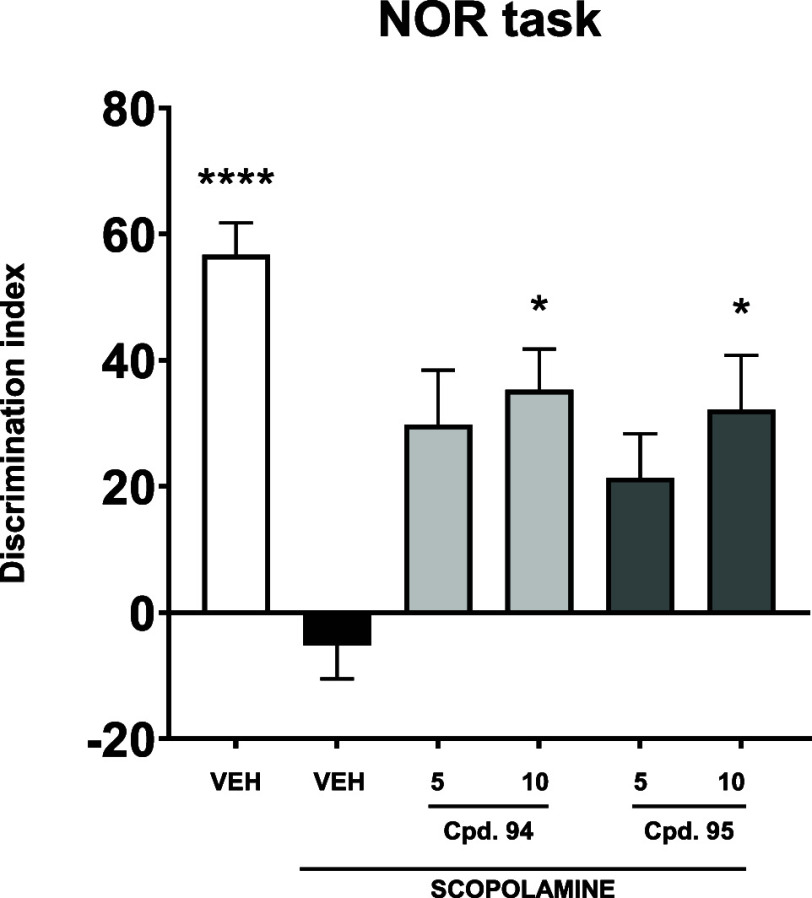
Effects of compounds **94** and **95** (doses: 5 and 10 mg/kg, i.p.) on scopolamine-induced cognitive deficits assessed in the NOR task. Results are shown as mean ± SEM for *n* = 8–10 mice. Statistical analysis: one-way ANOVA followed by Dunnett’s post hoc comparison. Significance vs scopolamine-treated control: * *p* < 0.05, **** *p* < 0.0001. VEH = vehicle.

In the mouse model of scopolamine-induced amnesia mimicking cholinergic deficit underlying AD, neflamapimod was also tested in the NOR task, but the results obtained for this compound (Figure 8B) did not reveal its memory-improving properties. To sum up this part of the in vivo study, the inhibition of p38α MAPK as a single mechanism is not sufficient to attenuate cognitive decline due to cholinergic deficit caused by scopolamine. Compared to the vehicle, neflamapimod at the highest dose tested (30 mg/kg, i.p.) did not affect animals’ locomotor activity (number of light-beam crossings—vehicle-treated group: 2068 ± 342 and neflamapimod-treated group: 2481 ± 195). In the rotarod test carried out at 6, 18, and 24 rpm, all mice from the vehicle-treated group and the neflamapimod-treated group were able to spend 60 s on the rotarod. Taken together, both assays confirmed that neflamapimod at doses tested in cognitive assays does not impair animals’ motor functions.

#### 2.5.3 LPS Model of Neuroinflammation

##### 2.5.3.1 Effect on Learning (Days 1–6)

In the LPS-induced model of neuroinflammation, compounds **94**, **95** (10 mg/kg dose; i.p.), and rivastigmine (1 mg/kg dose; i.p.) were tested in the MWM task after their subchronic administration to mice. The rodent’s performance in the MWM task highly relies on the hippocampal function.^73^ Given that LPS administration over a 7 day injection period leads to neuronal loss in the hippocampus due to neuroinflammation,^74^ this task seemed particularly well-suited to evaluate the neuroprotective properties of ligands **94** and **95**. Compounds with neuroprotective effects can mitigate cognitive deficits induced by LPS treatment in mice subjected to the MWM task. This is reflected in reduced escape latency scores, indicating improved spatial learning and memory as the mice find the hidden platform more quickly.^70^

As can be seen in Figure 10, statistically significantly lower escape latencies were observed on day 3 in the LPS + rivastigmine-treated group compared to the LPS-treated control group. However, in the rivastigmine-treated mice, the improvement in spatial learning was only transient and was not observed on days 4–6. On the other hand, mice treated with LPS and compound **94** (or **95**) showed a statistically significant decrease in escape latency from day 2 (**94**) or day 3 (**95**) to the end of the acquisition trial (day 6) compared to mice treated with LPS alone (Figure 10). These results clearly show that both **94** and **95** improved cognitive function in LPS-treated mice and were more efficient than the positive control, rivastigmine.

**Figure 10:**
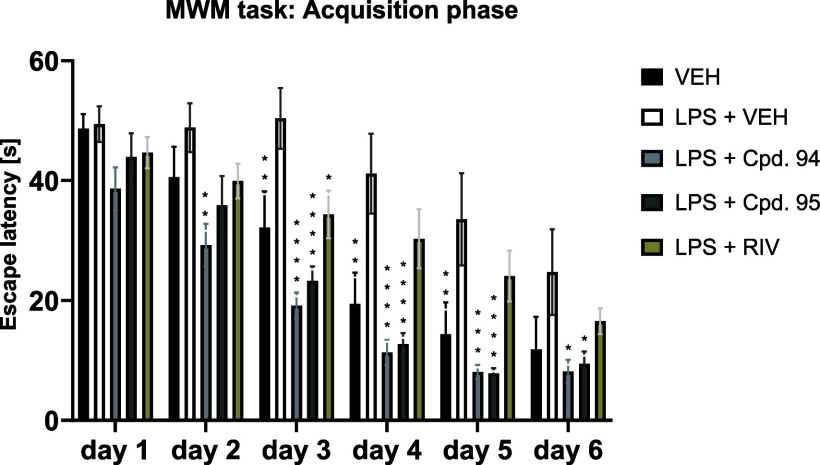
Effect of compounds **94**, **95** (dose: 10 mg/kg, i.p.) and the positive control rivastigmine (RIV, dose 1 mg/kg, i.p.) on spatial learning measured as escape latency and assessed in LPS-treated mice during the acquisition phase (days 1–6) of the MWM task. Results are shown as mean latency [s] to reach the hidden platform ± SEM for *n* = 9–10. Statistical analysis: repeated measures ANOVA followed by Dunnett’s post hoc comparison. Significance vs LPS-treated control on the respective test day: * *p* < 0.05, ** *p* < 0.01, *** *p* < 0.001, **** *p* < 0.0001. VEH = vehicle; LPS = lipopolysaccharide; Cpd. 94 = compound 94; Cpd. 95 = compound 95.

##### 2.5.3.2 Effect on Memory Retention (Day 7, Drug-Off Trial)

On day 7, which was a drug-off retention trial of the MWM task, the following parameters were measured: (1) latency to NW zone entry, (2) time in NW zone, (3) time spent at the target zone (i.e., the former platform location), and (4) the number of NW zone entries. A statistically significant difference was noted in the latency to NW zone entry between the LPS-treated control group and mice treated with LPS and **95** (*p* < 0.05, Figure 11A). Considering this, it can be concluded that the test compound **95** positively affected spatial memory retention measured in the MWM task. It also has to be noted that the reference drug tested in this study, rivastigmine, was not effective in the LPS model of neuroinflammation accompanied by memory decline (Figures 10 and 11).

**Figure 11:**
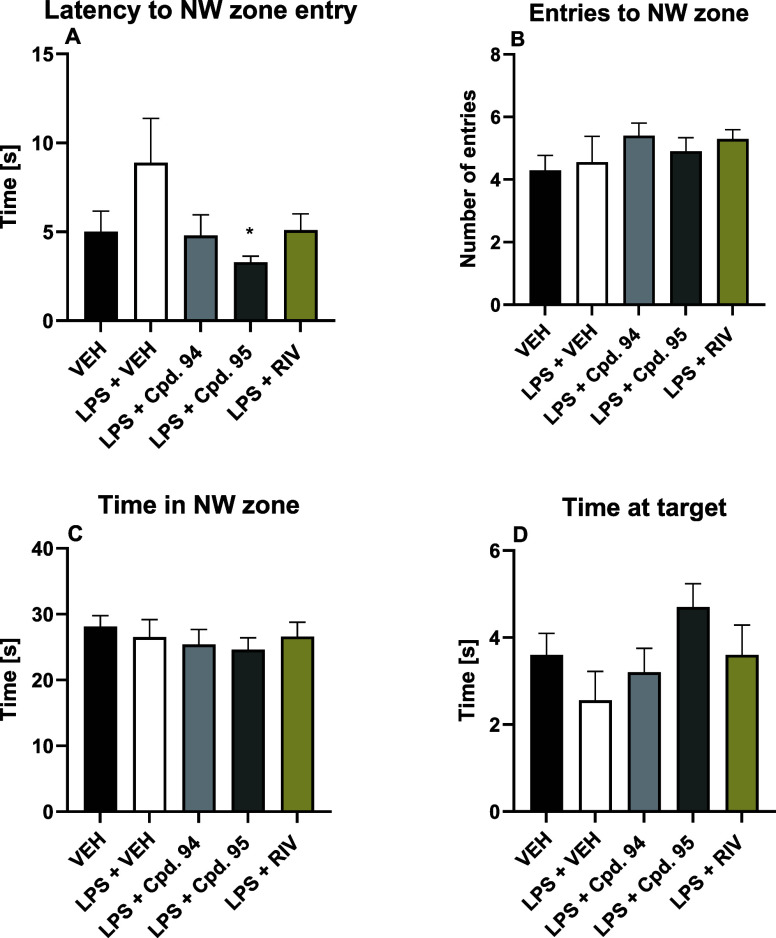
Effect of **94** and **95** (dose: 10 mg/kg, i.p.) and rivastigmine (RIV, dose: 1 mg/kg, i.p.) on memory retention measured in the mouse Morris water maze task on day 7 of testing. Results are shown as mean latency to NW zone entry ± SEM (A), mean number of NW zone entries ± SEM (B), mean time spent in NW zone ± SEM (C) and mean time spent at the target zone (D) (i.e., the former platform location) ± SEM for *n* = 8–10. Statistical analysis: one-way ANOVA followed by Dunnett’s (A, C, D) or Sidak’s (B) post hoc comparison. Significance vs LPS-treated control group: * *p* < 0.05; VEH = vehicle; LPS = lipopolysaccharide.

Next, the same experiment (MWM task; days 1–6 and 7) was carried out for neflamapimod administered to LPS-treated mice. Similar to rivastigmine, this p38α MAPK inhibitor at doses of 10 and 30 mg/kg turned out to be effective in improving spatial learning only on day 3 of the acquisition phase of the MWM task (Figure 12).

**Figure 12:**
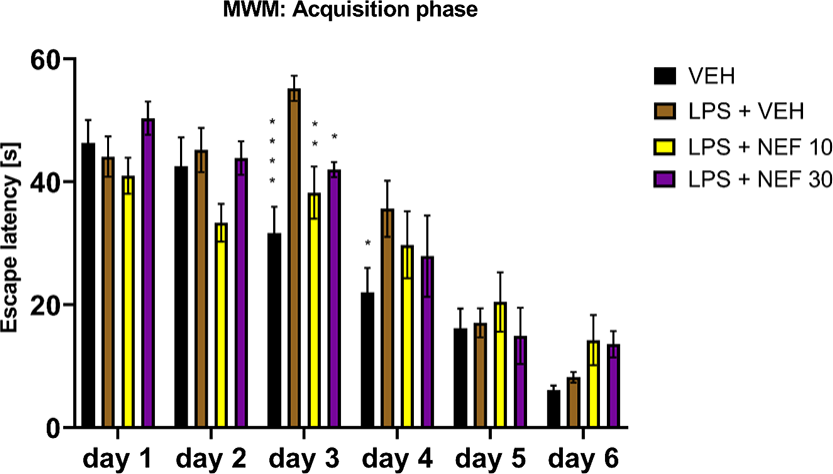
Effect of neflamapimod (NEF; doses: 10, 30 mg/kg, i.p.) on spatial learning measured as escape latency and assessed in LPS-treated mice during the acquisition phase (days 1–6) of the MWM task. Results are shown as mean latency [s] to reach the hidden platform ± SEM for *n* = 8–10. Statistical analysis: repeated measures ANOVA followed by Dunnett’s post hoc comparison. Significance vs LPS-treated control on the respective test day: * *p* < 0.05, ** *p* < 0.01, **** *p* < 0.0001; VEH = vehicle; LPS = lipopolysaccharide.

In the retention trial (day 7 of the MWM task), neflamapimod at the dose of 10 mg/kg increased both time spent in the NW zone (*p* < 0.05 vs LPS-treated control, Figure 13C) and time spent at the target zone (*p* < 0.05 vs LPS-treated control, Figure 13D). Thus, it can be concluded that this dose of neflamapimod positively influenced memory retention assessed in LPS-exposed mice.

**Figure 13:**
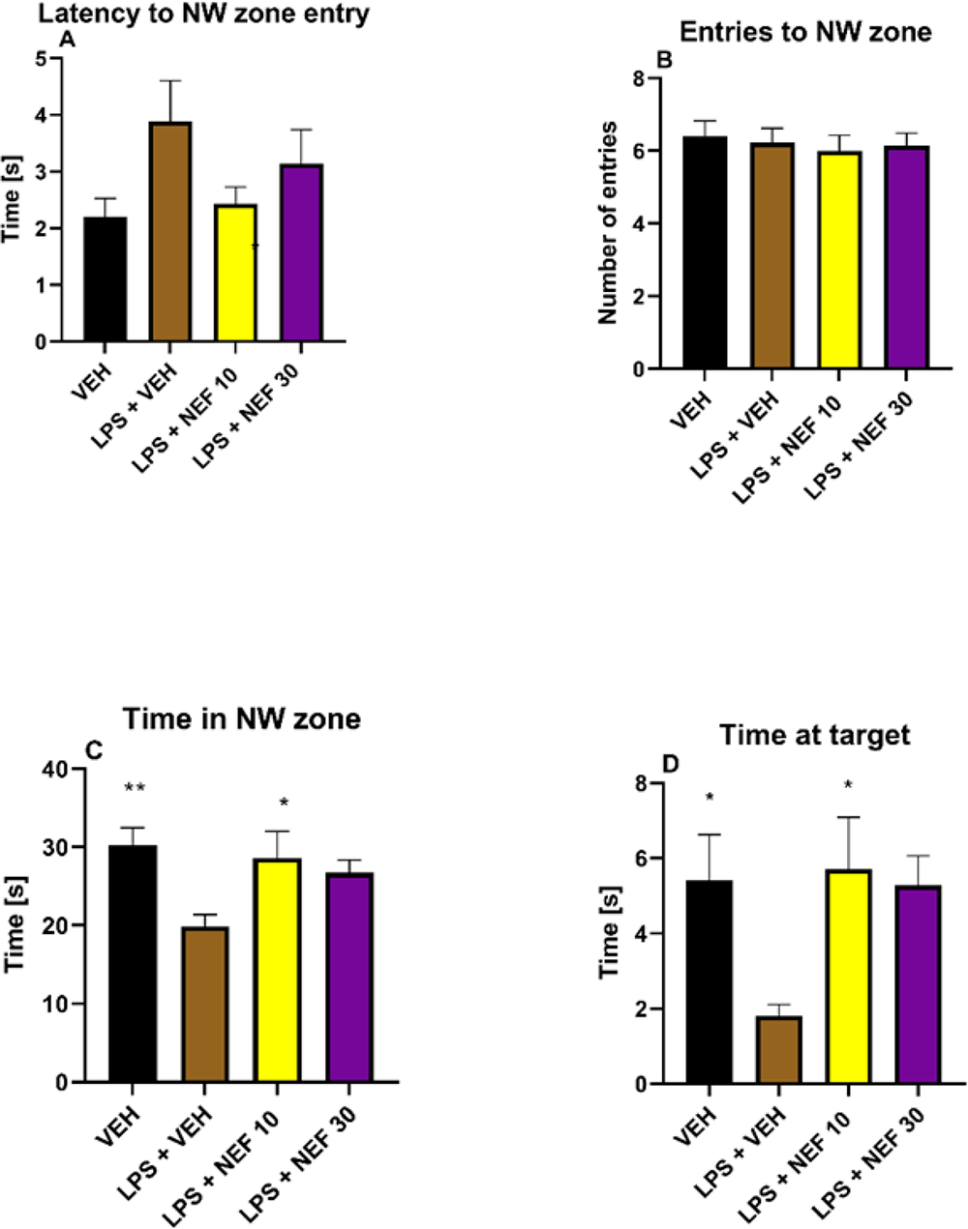
Effect of neflamapimod (NEF; doses: 10, 30 mg/kg, i.p.) on memory retention measured in the mouse MWM task on day 7 of testing. Results are shown as mean latency to NW zone entry ± SEM (A), mean number of NW zone entries ± SEM (B), mean time spent in NW zone ± SEM (C) and mean time spent at the target zone (D) (i.e., the former platform location) ± SEM for *n* = 7–10. Statistical analysis: one-way ANOVA followed by Dunnett’s (A, C, D), or Sidak’s (B) post hoc comparison. Significance vs LPS-treated control group: * *p* < 0.05, ** *p* < 0.01.

After completion of the MWM task, the mice were sacrificed, their brains removed, and analyzed for the expression of inflammatory state-related proteins, namely, TLR4, cyclooxygenase 2 (COX-2), cytosolic phospholipase A_2_ (cPLA2), and NLR family 3 pyrin domain-containing (NLRP3) inflammasome. The administration of LPS activates the TLR4 signaling pathway,^75^ which in turn triggers the activation of cPLA2 ^76^ and the release of arachidonic acid, which is converted into proinflammatory mediators via COX-2, which is also overexpressed by LPS stimulation.^77^ In addition, NLRP3 activation leads to increased IL-1β and caspase-1 production, which exacerbates cognitive impairment.^78,79^ Modulation of neuroinflammatory processes with a brain-permeable p38α MAPK inhibitor after LPS administration should lead to a significant decrease in the expression of these four proteins.

Brains of mice treated with both compound **94** or **95** and rivastigmine had significantly lower levels of TLR4, COX-2, and NLRP3, but no difference was seen in cPLA2 expression (Figure 14). These results provided clear evidence for the neuroprotective role of these ligands.

**Figure 14:**
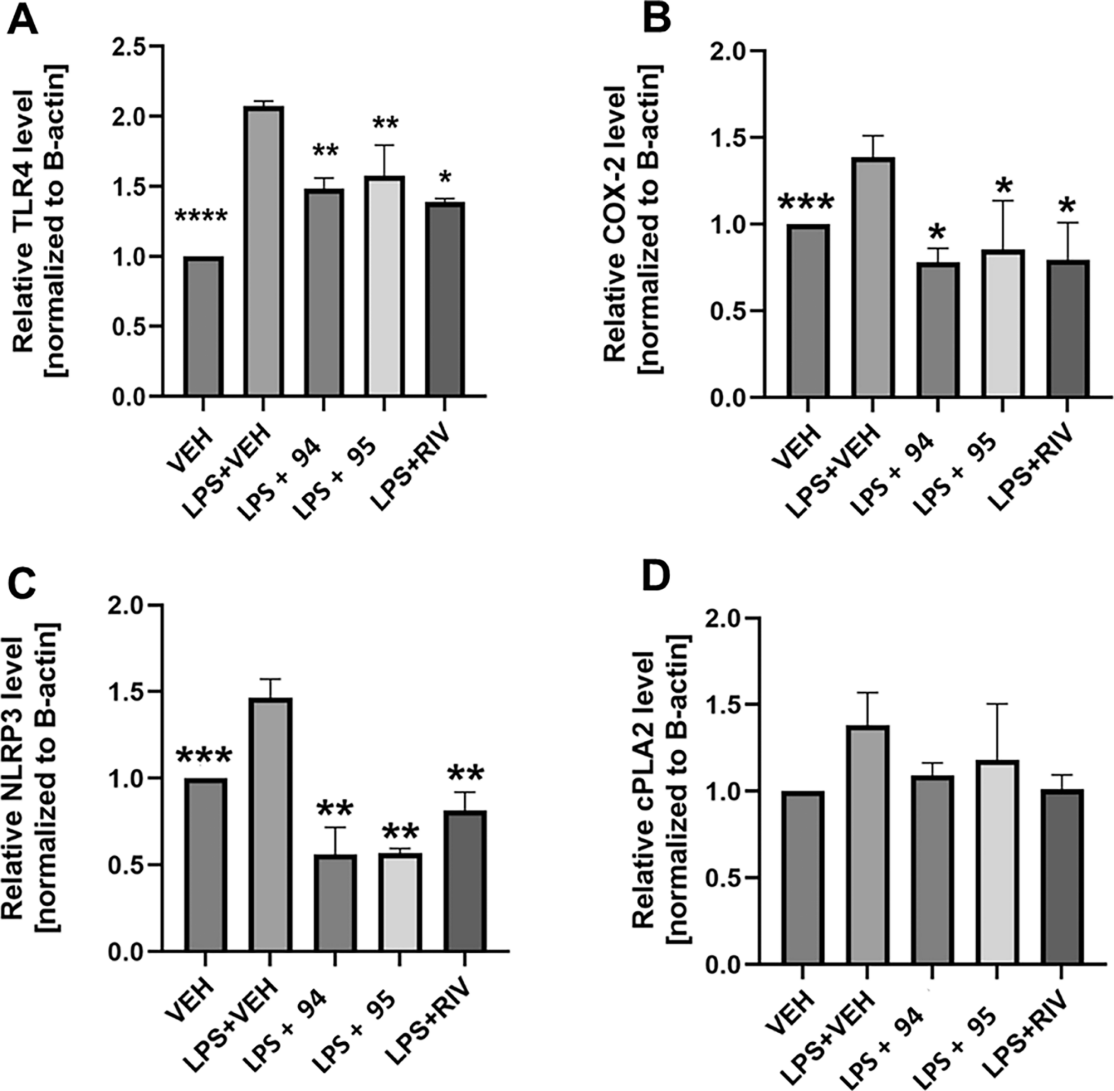
Effect of compounds 94, 95 (dose: 10 mg/kg, i.p.), and rivastigmine (RIV, dose 1 mg/kg, i.p.) on TLR4 (A), COX-2 (B), NLRP3 (C), and cPLA2 (D) levels in brains of mice stimulated by LPS administration. Statistical analysis: one-way ANOVA followed by Dunnett’s post hoc comparison. Significance vs LPS-treated control group: * *p* < 0.05, ** *p* < 0.01, *** *p* < 0.001, **** *p* < 0.0001; VEH = vehicle; LPS = lipopolysaccharide; 94 = compound 94; 95 = compound 95.

### 2.6 Pharmacokinetic Study

Concentration versus time profiles of compounds **94** and **95** in serum and brain tissue following administration of a dose of 10 mg/kg i.p. to mice are presented in Figure 15.

**Figure 15:**
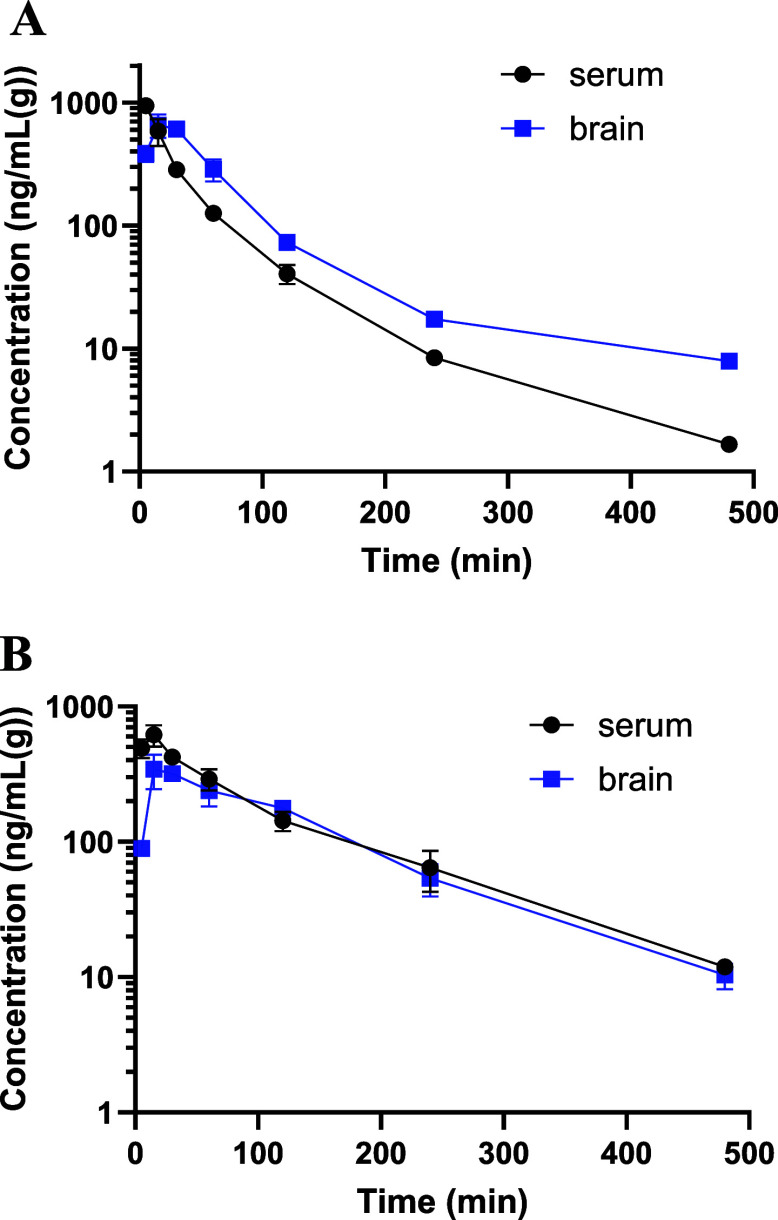
Mean (±SD) serum and brain concentrations of compound **94** (A) and compound **95** (B) following i.p. administration of a dose of 10 mg/kg to mice (*n* = 4). Pharmacokinetic parameters of these compounds calculated using the noncompartmental analysis are listed in Table 2.

The data presented in Figure 15 and Table 2 indicate that both compounds were rapidly absorbed into the blood after i.p. dosing to mice. The time to reach maximum concentration (*t*_max_) in serum was observed at the first sampling time, i.e., 5 min for compound **94** and at 15 min for compound **95**. Both compounds also reached their maximum concentration in the brain very fast, that is, at 15 min postdosing. The elimination half-lives of tested compounds were similar in serum and brain, and they were relatively long (about 100 min). In the brain tissue, compound **94** exhibited even slower elimination, as the value of this parameter was over 2-fold higher compared to that in serum. Serum and brain concentrations of both compounds were measured up to 480 min, and they were still above the quantification limit. The *C*_max_ of compound **94** to *C*_max_ of compound **95** was 1.53, whereas analogues AUC_0–*t*_ ratio was only 0.53. The apparent volumes of distribution (*V*_*z*_/*F*) of both compounds were very large in comparison to the mouse body water, indicating an extensive distribution of the tested compounds to mouse tissues. The brain-to-serum AUC ratio was 1.58 for compound **94** and 0.83 for compound **95**, indicating a higher brain penetration of compound **94**. However, both AUC and MRT of compounds **94** and **95** were similar in this tissue. Interestingly, *V*_*z*_/*F* and CL/*F* of compound **94** were twice as large as those of **95**. These differences may be the result of a different concentration versus time profile (a faster drop in serum concentrations observed after *C*_max_) and, in consequence, the lower value of AUC of compound **94** observed in serum despite the same dose administered (AUC occurs in the nominative case of the expression that allows for calculating these parameters). The observed concentrations of tested compounds in serum and brain tissue were maintained above in vitro hBChE IC_50_ values of compounds **94** and **95** (270 and 350 ng/mL, respectively) for at least 30 min from the time of dosing (for compound **94**, up to 60 min in brain). In the case of p38α MAPK IC_50_, effective concentrations of compound **94** and **95** (above 156 and 87 ng/mL) were maintained for at least 60 and 120 min, respectively. It should be kept in mind that IC_50_ values obtained in in vitro assays are frequently higher than those determined in vivo or therapeutic plasma concentrations.

**Table 2:** Pharmacokinetic Parameters of Compounds **94** and **95** Estimated Using the Model Independent Approach in Serum and Brain Following i.p. Administration of a Dose of 10 mg/kg to Mice^a^

	compound 94		compound 95	
pharmacokinetic parameter (unit)	serum	brain	serum	brain
*t*_max_ (min)	5	15	15	15
*C*_max_ (ng/mL(g))	946.00	658.38	616.67	342.83
λ_*z*_ [1/min]	0.0067	0.0033	0.0069	0.0077
t_0.5λ*z*_ [min]	102.50	210.94	100.15	89.51
AUC_0–*t*_ [ng·min/mL(g)]	31932.60	48459.19	59937.83	49811.92
AUC_0‑∞_ [ng·min/mL(g)]	32178.07	50863.30	61672.13	51082.15
*V*_*z*_/*F* [L/kg]	45.96		23.43	
CL/*F* [L/min/kg]	0.31		0.16	
MRT [min]	53.81	102.39	118.43	128.13

a Abbreviations: *C*_max_—maximum concentration, *t*_max_—time to reach maximum concentration, λ_*z*_—terminal elimination rate constant, *t*_0.5λ*z*_—terminal half-life, AUC_0–*t*_—area under the serum concentration–time curve from the time of dosing to the last measured point, AUC_0–∞_—area under the serum concentration–time curve extrapolated to infinity, CL/F—apparent serum clearance after extravascular administration, *V*_*z*_/*F*—volume of distribution based on the terminal phase, MRT—mean residence time.

To compare pharmacokinetic profiles in serum and the extent of brain distribution, pharmacokinetic studies were also performed for rivastigmine and neflamapimod used in this study as reference compounds. The results of these studies are presented in Figure S9 that shows concentration versus time profiles of rivastigmine and neflamapimod in serum and brain tissue following administration of the highest doses tested in vivo (2.5 and 30 mg/kg i.p., respectively) to mice. Pharmacokinetic parameters calculated using the noncompartmental analysis are listed in Table S9.

As shown in Figure S9 and Table S9, the reference compounds revealed different pharmacokinetic profiles. They were both very rapidly absorbed from the intraperitoneal cavity after i.p. dosing; however, their brain penetration was different. The brain-to-serum AUC ratio was 4.16 for rivastigmine and only 0.68 for neflamapimod. In contrast, rivastigmine almost disappeared from the blood within 120 min, whereas neflamapimod was present in serum and brain tissue up to 24 h. This effect was not only the result of different doses used, but also was caused by the huge difference in the values of terminal half-lives of these compounds (which were about 30 and 1000 min). The apparent volume of distribution (*V*_*z*_/*F*) was similar and very high for both enzyme inhibitors, whereas the clearance of rivastigmine was 32 times higher than that of neflamapimod. Interestingly, concentrations of rivastigmine in serum and brain tissue were higher than the hBChE IC_50_ (360 ng/mL) only at the first sampling time, whereas effective levels (above IC_50_) of neflamapimod were present in the serum and brain of mice for the whole observation period. This observation confirms that rivastigmine is a pseudoirreversible inhibitor of AChE and BChE and does not require sustained levels at the site of action to maintain its inhibitory effect.

## 3 Conclusions

Structure-based design of MTDLs that simultaneously inhibit p38α MAPK and hBChE led to the identification and subsequent optimization of the first dual inhibitors of these two AD-related target enzymes. Dual hBChE/p38α MAPK inhibitory activity was confirmed in vitro by enzyme assays and crystallographic studies, as well as in PBMC cells, in which our most promising compounds, **94** and **95**, reduced PHA-induced upregulation of proinflammatory cytokines. Moreover, these compounds were also effective in vivo, where they improved the cognitive function of mice with scopolamine- or LPS-induced cognitive deficits and reduced the expression of inflammatory proteins in their brains. Remarkably, these compounds were more effective in cognitive tasks than the reference compounds (rivastigmine and neflamapimod), suggesting that in AD, incorporating two mechanisms in one molecule with the MTDL strategy might be more efficacious than using drugs with only one mechanism of antiamnesic action. Thus, compounds **94** and **95** are promising lead compounds that target neuroinflammatory processes and alleviate the symptoms of AD associated with cholinergic hypofunction.

## 4 Experimental Section

### 4.1 Chemistry

#### 4.1.1 General Chemistry

Solvents and reagents were purchased from commercial suppliers and were used without further purification, unless otherwise stated. Purifications of crude intermediates and final compounds were performed with the indicated solvent mixtures as eluents for flash column chromatography (Merck, silica gel 60 for column chromatography, particle size 0.040–0.063 mm, 230–400 mesh), IsoleraOne flash purification system from Biotage or puriFlash 5.250 LC system from Advion Interchim Scientific (column and mobile phase used: SNAP Biotage KP-C18-HS column, 30 g; 0.1% TFA in deionized water/MeCN; gradient 0% MeCN for 2 column volumes; 10–100% MeCN in 15 column volumes; 100% MeOH for 5 column volumes). Analytical thin-layer chromatography was performed on silica gel aluminum sheets (0.20 mm; 60 F254; Merck), with visualization using ultraviolet light and/or visualization reagents. Some reactions were performed with temperature control mode in sealed microwave process vials, where the temperature was measured by an IR sensor outside the reaction vessel. Evaporation of the solvents was performed under reduced pressure. ^1^H NMR and ^13^C NMR data were recorded at 400.130 and 100.613 MHz, respectively, on an NMR spectrophotometer (Bruker Avance III). The chemical shifts (δ) are reported in parts per million (ppm) and are referenced to the deuterated solvent used. The coupling constants (*J*) are reported in Hz, and the signal multiplicities are indicated as s, singlet; bs, broad singlet; d, doublet; dd, doublet of doublets; td, triplet of doublets; m, multiplet; t, triplet; bt, broad triplet; dt, doublet of triplets; tt, triplet of triplets; q, quartet; qd, quartet of doublets. Infrared (IR) spectra were recorded on a FT-IR spectrometer (System Spectrum BX; PerkinElmer). Mass spectra were recorded on Agilent 6224 Accurate Mass TOF LC/MS and Thermo Scientific Q Exactive Plus LC-MS/MS spectrometers. UPLC analyses of the final compound purity were performed on a Thermo Scientific Dionex UltiMate 3000 modular system (Thermo Fisher Scientific Inc.). The general method used a Waters Acquity UPLC HSS C18 SB column (2.1 × 50 mm, 1.8 μm) thermostated at 40 °C, with injection volume, 4 μL; sample, 0.1–0.2 mg/mL in MeOH; flow rate, 0.4 mL/min; detector λ, 220 and 254 nm; mobile phase A: 0.1% TFA (v/v) in water; mobile phase B: MeCN. Gradient: 0–2 min, 20% B; 2–5 min, 20–90% B; 5–8 min, 90% B. All final compounds (**72–109**) are ≥95% pure as determined by UHPLC analysis.

#### 4.1.2 2-Fluoro-4-methyl-5-nitrobenzoic Acid (2)

To a 500 mL round-bottomed flask equipped with a stirring bar, 2-fluoro-4-methylbenzoic acid (**1**) (10 g, 64.88 mmol, 1.0 equiv) was added and dissolved in 100 mL 96% H_2_SO_4_. The solution was cooled to 0 °C, and the round-bottomed flask was equipped with a dropping funnel through which a mixture of HNO_3_ (6.7 mL, 147.80 mmol, 2.3 equiv) in 96% H_2_SO_4_ (4.3 mL) was added dropwise over 5 min. The dropping funnel was removed, and the reaction mixture was allowed to stir in an ice bath for 2 h. After that, ice-cold water (250 mL) was gradually added to the mixture, resulting in a formation of a precipitate, which was filtered, dissolved in 150 mL of CH_2_Cl_2_–MeOH = 3:1, v/v, washed with brine (100 + 50 mL), and dried with Na_2_SO_4_. Organic phases were evaporated on a rotary evaporator, and the solid product was further dried in vacuo at room temperature in the presence of NaOH and SICAPENT to constant mass to produce a light brown solid (11.922 g, 92% yield). *R*_f_ = 0.22 (CH_2_Cl_2_/MeOH 5:1); ^1^H NMR (400 MHz, CDCl_3_, ppm) δ 8.67 (s, 1H), 7.77 (s, 1H), 2.67 (s, 3H). Signal for −COOH is not visible.

#### 4.1.3 Methyl-2-fluoro-4-methyl-5-nitrobenzoate (3)

In a 250 mL round-bottom flask equipped with a stirring bar, 2-fluoro-4-methyl-5-nitrobenzoic acid (**2**) (11.92 g, 59.85 mmol, 1.0 equiv) was dissolved in 50 mL anhydrous *N*,*N*-dimethylformamide under an argon atmosphere. K_2_CO_3_ (19.02 g, 137.58 mmol, 2.3 equiv) was then added in 4 portions. After 20 min of stirring at room temperature, methyl iodide (11.17 mL, 179.43 mmol, 3.0 equiv) was added dropwise. The reaction mixture was left to stir at room temperature under an argon atmosphere overnight. After 18 h, water (30 mL) was added, and the mixture was transferred to a separatory funnel. The product was extracted with EtOAc (2 × 50 mL), and the organic phases were then further washed with 50 mL brine, dried with Na_2_SO_4_, and evaporated under reduced pressure using an oil pump. The product was then purified by recrystallization from the mixture of EtOAc and hexane to obtain dark yellow crystals (8.46 g, 66% yield). *R*_f_ = 0.78 (EtOAc/MeOH 2:1); ^1^H NMR (400 MHz, CDCl_3_, ppm) δ 8.67 (d, *J* = 6.7 Hz, 1H), 7.14 (d, *J* = 10.6 Hz, 1H), 3.97 (s, 3H), 2.68 (s, 3H).

#### 4.1.4 Representative Synthetic Procedure for Compounds 4–9

2-(2,4-Difluorophenoxy)-4-methyl-5-nitrobenzoate (**4**), methyl 2-((2,4-difluorophenyl)­thio)-4-methyl-5-nitrobenzoate (**5**), methyl 2-(4-fluorophenoxy)-4-methyl-5-nitrobenzoate (**6**), methyl 4-methyl-2-(naphthalen-1-yloxy)-5-nitrobenzoate (**7**), methyl 4-methyl-2-(naphthalen-2-yloxy)-5-nitrobenzoate (**8**) and methyl 2-(4-methoxyphenoxy)-4-methyl-5-nitrobenzoate (**9**).

To a 100 mL round-bottom flask with a stirring bar, methyl 2-fluoro-4-methyl-5-nitrobenzoate (**3**) (0.978 g, 4.59 mmol, 1.0 equiv) was added and subsequently dissolved in 10 mL of anhydrous DMF under an argon atmosphere. Cs_2_CO_3_ (2.24 g, 6.90 mmol, 1.5 equiv) was added in 2 portions, followed by dropwise addition of 2,4-difluorophenol (444 μL, 4.65 mmol, 1.0 equiv). The reaction mixture was then heated to 60 °C and left to stir overnight under an argon atmosphere. After 18 h, the reaction was cooled to room temperature and 30 mL of water was added before transferring the contents of the round-bottom flask to a separatory funnel, where the product was extracted with EtOAc (2 × 30 mL). Combined organic phases were washed with 30 mL of 10% (m/v) citric acid, 30 mL of 1 M NaOH solution, and 30 mL of brine and dried with Na_2_SO_4_. Evaporating the solvent and drying the product on an oil pump gave methyl 2-(2,4-difluorophenoxy)-4-methyl-5-nitrobenzoate (**4**) as a light brown solid (1.328 g, 90% yield). *R*_f_ = 0.26 (EtOAc/hexane 1:2); ^1^H NMR (400 MHz, CDCl_3_, ppm) δ 8.69 (s, 1H), 7.17 (td, *J* = 8.9, 5.4 Hz, 1H), 7.02 (ddd, *J* = 10.2, 8.2, 2.9 Hz, 1H), 6.95 (m, 1H), 6.61 (t, *J* = 0.9 Hz, 1H), 3.94 (s, 3H), 2.57 (s, 3H).

##### 4.1.4.1 Methyl-2-((2,4-difluorophenyl)­thio)-4-methyl-5-nitrobenzoate (5)

The compound was prepared according to the representative synthetic procedure described above. Compound **5** was synthesized with a yield of 85%. *R*_f_ = 0.79 (EtOAc/hexane 1:1). ^1^H NMR (400 MHz, CDCl_3_, ppm) δ 8.74 (s, 1H), 7.66–7.56 (m, 1H), 7.10–6.96 (m, 2H), 6.57 (t, *J* = 1.1 Hz, 1H), 4.00 (s, 3H), 1.55 (s, 3H).

##### 4.1.4.2 Methyl-2-(4-fluorophenoxy)-4-methyl-5-nitrobenzoate (**6**)

The compound was prepared according to the representative synthetic procedure described above. Compound **6** was synthesized with a yield of 70%. *R*_f_ = 0.79 (DCM/MeOH 30:1). ^1^H NMR (400 MHz, CDCl_3_, ppm) δ 1.26 (s, 1H), 2.56 (d, *J* = 0.7 Hz, 3H), 3.92 (s, 3H), 6.68 (d, *J* = 0.8 Hz, 1H), 7.00–7.18 (m, 4H), 8.67 (s, 1H).

##### 4.1.4.3 Methyl-4-methyl-2-(naphthalen-1-yloxy)-5-nitrobenzoate (**7**)

The compound was prepared according to the representative synthetic procedure described above. Compound **7** was synthesized with a yield of 78%. *R*_f_ = 0.45 (EtOAc/hexane 1:3). ^1^H NMR (400 MHz, CDCl_3_, ppm) δ 2.47 (d, *J* = 0.7 Hz, 3H), 3.90 (s, 3H), 6.62 (d, *J* = 0.8 Hz, 1H), 7.11 (dd, *J* = 7.5, 1.0 Hz, 1H), 7.44–7.61 (m, 3H), 7.74–7.81 (m, 1H), 7.93 (dd, *J* = 7.7, 1.4 Hz, 1H), 7.99–8.07 (m, 1H), 8.73 (s, 1H).

##### 4.1.4.4 Methyl-4-methyl-2-(naphthalen-2-yloxy)-5-nitrobenzoate (**8**)

The compound was prepared according to the representative synthetic procedure described above. Compound **8** was synthesized with a yield of 69%. *R*_f_ = 0.77 (EtOAc/hexane 1:3). ^1^H NMR (400 MHz, CDCl_3_, ppm) ^1^H NMR (400 MHz, CDCl_3_): δ 2.56–2.51 (m, 3H), 3.91 (s, 3H), 6.80–6.75 (m, 1H), 7.26 (dd, *J* = 8.8, 2.4 Hz, 1H), 7.57–7.42 (m, 3H), 7.83–7.74 (m, 1H), 7.96–7.83 (m, 2H), 8.71 (s, 1H).

##### 4.1.4.5 Methyl-2-(4-methoxyphenoxy)-4-methyl-5-nitrobenzoate (**9**)

The compound was prepared according to the representative synthetic procedure described above. Compound **9** was synthesized with a yield of 68%. *R*_f_ = 0.31 (EtOAc/hexane 1:3). ^1^H NMR (400 MHz, CDCl_3_, ppm) δ 2.54 (d, *J* = 0.7 Hz, 3H), 3.84 (s, 3H), 3.93 (s, 3H), 6.64 (d, *J* = 0.9 Hz, 1H), 6.91–6.99 (m, 2H), 6.99–7.01 (m, 2H), 8.67 (s, 1H).

#### 4.1.5 Representative Synthetic Procedure for Compounds 10–15

Methyl 5-amino-2-(2,4-difluorophenoxy)-4-methylbenzoate (**10**), methyl 5-amino-2-((2,4-difluorophenyl)­thio)-4-methylbenzoate (**11**), methyl 5-amino-2-(4-fluorophenoxy)-4-methylbenzoate (**12**), methyl 5-amino-2-(naphthalen-1-yloxy)-5-nitrobenzoate (**13**), methyl 5-amino-2-(naphthalen-2-yloxy)-5-nitrobenzoate (**14**) and methyl 5-amino-2-(4-methoxyphenoxy)-4-methylbenzoate (**15**).

Methyl-2-(2,4-difluorophenoxy)-4-methyl-5-nitrobenzoate (**4**) (1.325 g, 4.10 mmol, 1.0 equiv) was dissolved in 60 mL EtOAc in a 100 mL round-bottom flask. The contents of the flask were flushed with argon for 10 min. After that, 10% Pd/C (0.130 g, 1.22 mmol, 0.3 equiv) was added carefully in two portions. The flask was flushed with hydrogen, and the reaction mixture was left to stir overnight at room temperature under a hydrogen atmosphere. After 16 h, the flask and its contents were again flushed with argon and left to stir under an argon atmosphere for 15 min. A pad of Celite 545 was prepared, and the reaction mixture was slowly filtered through it. The mother liquor was evaporated under reduced pressure to obtain methyl 5-amino-2-(2,4-difluorophenoxy)-4-methylbenzoate (**10**) as a viscous brown oil (1.203 g, 100% yield). *R*_f_ = 0.26 (EtOAc/hexane 1:2); ^1^H NMR (400 MHz, CDCl_3_, ppm) δ 7.26 (s, 1H), 6.92 (m, 1H), 6.75 (s, 1H), 6.73–6.69 (m, 2H), 3.77 (s, 3H), 3.64 (s, 2H), 2.17 (s, 3H); LC-MS (ESI^+^) *m*/*z*: calcd for C_15_H_13_F_2_NO_3_ [M + H]^+^: 293.3; found: 294.1.

##### 4.1.5.1 Methyl-5-amino-2-((2,4-difluorophenyl)­thio)-4-methylbenzoate (**11**)

The compound was prepared according to the representative synthetic procedure described above. Compound **11** was synthesized with a yield of 80%. *R*_f_ = 0.45 (EtOAc/hexane 1:3). ^1^H NMR (400 MHz, CDCl_3_, ppm) δ 7.44–7.36 (m, 1H), 7.27 (s, 1H), 6.93–6.86 (m, 2H), 6.62 (s, 1H), 3.89 (s, 3H), 3.67 (s, 2H), 2.05 (s, 3H).

##### 4.1.5.2 Methyl-5-amino-2-(4-fluorophenoxy)-4-methylbenzoate (**12**)

The compound was prepared according to the representative synthetic procedure described above. Compound **12** was synthesized with a yield of 84%. *R*_f_ = 0.34 (EtOAc/hexane 1:1). ^1^H NMR (400 MHz, CDCl_3_, ppm) δ 3.83 (s, 3H), 6.84–6.93 (m, 2H), 6.93–7.03 (m, 2H), 7.34 (d, *J* = 0.6 Hz, 1H), 8.07 (d, *J* = 1.1 Hz, 1H), 8.12 (t, *J* = 0.8 Hz, 1H), 11.94 (s, 1H).

##### 4.1.5.3 Methyl-5-amino-2-(naphthalen-1-yloxy)-5-nitrobenzoate (**13**)

The compound was prepared according to the representative synthetic procedure described above. Compound **13** was synthesized with a yield of 78%. *R*_f_ = 0.32 (EtOAc/hexane 1:2). LC-MS (ESI^+^) *m*/*z*: calcd for C_19_H_17_NO_3_ [M + H]^+^: 308.1; found: 309.0.

##### 4.1.5.4 Methyl-5-amino-2-(naphthalen-2-yloxy)-5-nitrobenzoate (**14**)

The compound was prepared according to the representative synthetic procedure described above. Compound **14** was synthesized with a yield of 76%. *R*_f_ = 0.37 (EtOAc/hexane 1:2). LC-MS (ESI^+^) *m*/*z*: calcd for C_19_H_17_NO_3_ [M + H]^+^: 308.1; found: 308.9.

##### 4.1.5.5 Methyl-5-amino-2-(4-methoxyphenoxy)-4-methylbenzoate (**15**)

The compound was prepared according to the representative synthetic procedure described above. Compound **15** was synthesized with a yield of 70%. *R*_f_ = 0.22 (EtOAc/hexane 1:2). LC-MS (ESI^+^) *m*/*z*: calcd for C_16_H_17_NO_4_ [M + H]^+^: 288.1; found: 288.9.

#### 4.1.6 Representative Synthetic Procedure for Compounds 16–21

Methyl-5-(2,4-difluorophenoxy)-1*H*-indazole-6-carboxylate (**16**), methyl 5-((2,4-difluorophenyl)­thio)-1*H*-indazole-6-carboxylate (**17**), methyl 5-(4-fluorophenoxy)-1*H*-indazole-6-carboxylate (**18**), methyl 5-(naphthalen-1-yloxy)-1*H*-indazole-6-carboxylate (**19**), methyl 5-(naphthalen-2-yloxy)-1*H*-indazole-6-carboxylate (**20**), and methyl 5-(4-methoxyphenoxy)-1*H*-indazole-6-carboxylate (**21**).

In a 100 mL round-bottom flask equipped with a stirring bar, methyl 5-amino-2-(2,4-difluorophenoxy)-4-methylbenzoate (**10**) (1.257 g, 4.29 mmol, 1.0 equiv) was dissolved in 60 mL of chloroform. The solution was cooled to 0 ° in an ice bath, after which sodium acetate (56 mg, 0.68 mmol, 0.16 equiv) and acetic acid (4 mL, 69.94 mmol, 16.3 equiv) were added to the flask. After 15 min of stirring on an ice bath, *tert*-butyl nitrite (1.27 mL, 10.68 mmol, 2.5 equiv) was added dropwise. After an additional 15 min, the ice bath was removed and the reaction mixture was left to warm to room temperature. Once all the starting compound was consumed (monitored by TLC), the solvents were evaporated on a rotary evaporator, and the residue was dissolved in 50 mL of EtOAc, which was then carefully washed with a saturated NaHCO_3_ solution (2 × 30 mL). The organic phase was dried with Na_2_SO_4_ and evaporated under reduced pressure. The crude was purified by flash column chromatography using EtOAc–hexane (1:2, v/v) as the eluent to produce methyl 5-(2,4-difluorophenoxy)-1*H*-indazole-6-carboxylate (**16**) as a viscous orange oil (0.811 g, 62% yield). *R*_f_ = 0.35 (EtOAc/hexane 1:2); ^1^H NMR (400 MHz, CDCl_3_, ppm) δ 11.53 (s, 1H), 8.14 (t, *J* = 0.8 Hz, 1H), 8.05 (d, *J* = 1.1 Hz, 1H), 7.27 (d, *J* = 0.6 Hz, 1H), 6.96 (ddd, *J* = 11.0, 8.4, 2.9 Hz, 1H), 6.87 (td, *J* = 9.0, 5.4 Hz, 1H), 6.78 (m, 1H), 3.88 (s, 3H); ^13^C NMR (101 MHz, CDCl_3_, ppm) δ 165.05, 160.08 (d, *J* = 10.3 Hz), 157.63 (d, *J* = 10.1 Hz), 155.01 (d, *J* = 12.2 Hz), 152.51 (d, *J* = 12.2 Hz), 149.29, 139.86 (dd, *J* = 11.6, 3.8 Hz), 136.69, 133.96, 124.40, 121.95 (dd, *J* = 9.6, 1.9 Hz), 114.11, 111.53 (dd, *J* = 22.9, 3.9 Hz), 107.25, 105.57 (dd, *J* = 26.8, 21.7 Hz), 57.40, 44.80, 37.58, 29.70; LC-MS (ESI^+^) *m*/*z*: calcd for C_15_H_10_F_2_N_2_O_3_ [M + H]^+^: 304.3; found: 304.9.

##### 4.1.6.1 Methyl-5-((2,4-difluorophenyl)­thio)-1*H*-indazole-6-carboxylate (17)

The compound was prepared according to the representative synthetic procedure described above. Compound **17** was synthesized with a yield of 89%. *R*_f_ = 0.34 (EtOAc/hexane 1:1). ^1^H NMR (400 MHz, CDCl_3_, ppm) δ 8.20 (s, 1H), 7.94 (s, 1H), 7.58–7.49 (m, 1H), 7.23 (s, 1H), 7.01–6.90 (m, 2H), 3.99 (s, 3H).

##### 4.1.6.2 Methyl-5-(4-fluorophenoxy)-1*H*-indazole-6-carboxylate (18)

The compound was prepared according to the representative synthetic procedure described above. Compound **18** was synthesized with a yield of 71%. *R*_f_ = 0.34 (EtOAc/hexane 1:2). ^1^H NMR (400 MHz, CDCl_3_, ppm) δ δ 3.83 (s, 3H), 6.84–6.93 (m, 2H), 6.93–7.03 (m, 2H), 7.34 (d, *J* = 0.6 Hz, 1H), 8.07 (d, *J* = 1.1 Hz, 1H), 8.12 (t, *J* = 0.8 Hz, 1H), 11.94 (s, 1H).

##### 4.1.6.3 Methyl-5-(naphthalen-1-yloxy)-1*H*-indazole-6-carboxylate (19)

The compound was prepared according to the representative synthetic procedure described above. Compound **19** was synthesized with a yield of 53%. *R*_f_ = 0.13 (EtOAc/hexane 1:2). ^1^H NMR (400 MHz, CDCl_3_, ppm) δ 3.65 (s, 3H), 6.69 (dd, *J* = 7.7, 1.0 Hz, 1H), 7.31 (t, *J* = 8.0 Hz, 1H), 7.41 (d, *J* = 0.6 Hz, 1H), 7.60–7.49 (m, 3H), 7.92–7.82 (m, 1H), 8.03 (d, *J* = 1.1 Hz, 1H), 8.17 (t, *J* = 0.8 Hz, 1H), 8.42–8.33 (m, 1H), 10.43 (s, 1H).

##### 4.1.6.4 Methyl-5-(naphthalen-2-yloxy)-1*H*-indazole-6-carboxylate (20)

The compound was prepared according to the representative synthetic procedure described above. Compound **20** was synthesized with a yield of 50%. *R*_f_ = 0.37 (EtOAc/hexane 1:2). ^1^H NMR (400 MHz, CDCl_3_, ppm): δ 3.78 (s, 3H), 7.08 (d, *J* = 2.5 Hz, 1H), 7.30 (dd, *J* = 8.9, 2.5 Hz, 1H), 7.39 (dddd, *J* = 17.3, 8.2, 6.8, 1.4 Hz, 2H), 7.49 (d, *J* = 0.6 Hz, 1H), 7.61 (dd, *J* = 8.1, 1.5 Hz, 1H), 7.77–7.87 (m, 2H), 8.08 (d, *J* = 1.1 Hz, 1H), 8.19 (d, *J* = 0.9 Hz, 1H), 10.53 (s, 1H).

##### 4.1.6.5 Methyl-5-(4-methoxyphenoxy)-1*H*-indazole-6-carboxylate (**21**)

The compound was prepared according to the representative synthetic procedure described above. Compound **21** was synthesized with a yield of 41%. *R*_f_ = 0.15 (EtOAc/hexane 1:2). ^1^H NMR (400 MHz, CDCl_3_, ppm) δ 3.80 (s, 3H), 3.87 (s, 3H), 6.84–6.89 (m, 2H), 6.90–6.95 (m, 2H), 7.28 (d, *J* = 0.6 Hz, 1H), 8.01 (d, *J* = 1.1 Hz, 1H), 8.07 (t, *J* = 0.8 Hz, 1H), 10.40 (s, 1H).

#### 4.1.7 5-(2,4-Difluorophenoxy)-1*H*-indazole-6-carboxylic acid (56)

To a 25 mL round-bottom flask with a stirring bar methyl 5-(2,4-difluorophenoxy)-1*H*-indazole-6-carboxylate (**16**) (0.100 g, 0.37 mmol, 1.0 equiv) was added and dissolved in 6 mL of THF. 1 M aqueous LiOH (2.0 mL, 2.00 mmol, 3.0 equiv) was added dropwise during vigorous stirring of the solution at room temperature. The reaction mixture was then left to stir under reflux and monitored by TLC. After 5 h, the mixture was left to cool, and THF was evaporated. The remaining aqueous phase was acidified by dropwise addition of 1 M HCl to pH = 0. The precipitate that formed was filtered under suction and dried on a rotary evaporator to yield a light brown solid (0.081 g, 84% yield). *R*_f_ = 0.08 (CH_2_Cl_2_/MeOH 10:1); ^1^H NMR (400 MHz, DMSO-*d*_6_, ppm) δ 13.42 (s, 1H), 8.10 (s, 1H), 8.05 (s, 1H), 7.47–7.40 (m, 2H), 6.99 (m, 1H), 6.88 (td, *J* = 9.3, 5.5 Hz, 1H). The signal for 1*H* of indazole is not visible. LC-MS (ESI^–^) *m*/*z*: calculated for C_14_H_8_F_2_N_2_O_3_ [M]^−^: 290.2; found: 288.8.

#### 4.1.8 5-(2,4-Difluorophenoxy)-*N*-(2-(dimethylamino)­ethyl)-1*H*-indazole-6-carboxamide (87)

To a 25 mL round-bottom flask equipped with a stirring bar, 5-(2,4-difluorophenoxy)-1*H*-indazole-6-carboxylic acid (**56**) (0.074 g, 0.26 mmol, 1.0 equiv) was added. CH_2_Cl_2_ (15 mL) was added under argon, and a suspension was formed, which was cooled to 0 °C on an ice bath. Subsequently, Et_3_N (71 μL, 0.51 mmol, 2.0 equiv) was added dropwise, resulting in dissolution of **56**. After 10 min, TBTU (0.082 g, 0.26 mmol, 1.0 equiv) was added in two portions, and the reaction mixture was left to stir on an ice bath under argon for 1 h before *N*′,*N*′-dimethylethane-1,2-diamine (56 μL, 0.510 mmol, 2.0 equiv) was added. Finally, the reaction mixture was left to warm to room temperature and stir for 2 days. After 46 h, the reaction mixture was washed with saturated NaHCO_3_ solution (2 × 10 mL) and brine (2 × 10 mL), dried with Na_2_SO_4_, and CH_2_Cl_2_ evaporated under reduced pressure. The crude obtained was then purified by flash column chromatography using CH_2_Cl_2_–MeOH (1:1, v/v) and reverse-phase preparative chromatography (Biotage Sfär, C18 D 30 g column) using 0.1% TFA­(aq)/MeCN as a mobile phase to obtain a white solid (0.040 g, 43% yield). *R*_f_ = 0.29 (CH2Cl2/MeOH 1:1); ^1^H NMR (400 MHz, DMSO-*d*_6_) δ 13.31 (s, 1H), 8.33 (t, *J* = 5.4 Hz, 1H), 8.04 (s, 1H), 7.84 (s, 1H), 7.54–7.43 (m, 1H), 7.28 (s, 1H), 7.15–6.97 (m, 2H), 3.31–3.28 (m, 2H), 2.33 (d, *J* = 6.9 Hz, 2H), 2.13 (s, 6H). Signal for 1*H*-indazole is not visible; 13C NMR (101 MHz, CDCl3, ppm) δ 165.05, 160.08 (d, *J* = 10.3 Hz), 157.63 (d, *J* = 10.1 Hz), 155.01 (d, *J* = 12.2 Hz), 152.51 (d, *J* = 12.2 Hz), 149.29, 139.86 (dd, *J* = 11.6, 3.8 Hz), 136.69, 133.96, 124.40, 121.95 (dd, *J* = 9.6, 1.9 Hz), 114.11, 111.53 (dd, *J* = 22.9, 3.9 Hz), 107.25, 105.57 (dd, *J* = 26.8, 21.7 Hz), 57.40, 44.80, 37.58; HRMS (ESI^+^) *m*/*z*: calcd for C_18_H_18_F_2_N_4_O_2_ [M + H]^+^: 360.1398; found: 361.1460; UPLC purity: 95%.

#### 4.1.9 Representative Synthetic Procedure for Compounds 26–32, 34–38, 42–46

Methyl 5-(2,4-difluorophenoxy)-1-isobutyl-1*H*-indazole-6-carboxylate (**26**), methyl 1-(cyclopropylmethyl)-5-(2,4-difluorophenoxy)-1*H*-indazole-6-carboxylate (**27**), methyl 1-(cyclohexylmethyl)-5-(2,4-difluorophenoxy)-1*H*-indazole-6-carboxylate (**28**), methyl 1-(cyclobutylmethyl)-5-(2,4-difluorophenoxy)-1*H*-indazole-6-carboxylate (**30**), methyl 5-(2,4-difluorophenoxy)-1-isopentyl-1*H*-indazole-6-carboxylate (**31**), methyl 1-benzyl-5-(2,4-difluorophenoxy)-1*H*-indazole-6-carboxylate (**32**), methyl 5-(4-fluorophenoxy)-1-isobutyl-1*H*-indazole-6-carboxylate (**34**), methyl 1-isobutyl-5-(naphthalen-1-yloxy)-1*H*-indazole-6-carboxylate (**35**), methyl 1-isobutyl-5-(naphthalen-2-yloxy)-1*H*-indazole-6-carboxylate (**36**), methyl 1-isobutyl-5-(4-methoxyphenoxy)-1*H*-indazole-6-carboxylate (**37**), methyl 1-isobutyl-5-((2,4-difluorophenyl)­thio)-1*H*-indazole-6-carboxylate (**38**), methyl 5-(2,4-difluorophenoxy)-2-isobutyl-2*H*-indazole-6-carboxylate (**42**), methyl 2-(cyclopropylmethyl)-5-(2,4-difluorophenoxy)-2*H*-indazole-6-carboxylate (**43**), methyl 2-(cyclohexylmethyl)-5-(2,4-difluorophenoxy)-2*H*-indazole-6-carboxylate (**44**), methyl 2-benzyl-5-(2,4-difluorophenoxy)-2*H*-indazole-6-carboxylate (**45**), and methyl 5-(4-fluorophenoxy)-2-isobutyl-2*H*-indazole-6-carboxylate (**46**).

To a 25 mL round-bottom flask with a stirring bar, methyl 5-(2,4-difluorophenoxy)-1*H*-indazole-6-carboxylate (**16**) (0.200 g, 0.66 mmol, 1.0 equiv) was added. Anhydrous DMF (20 mL) was added under argon and the contents stirred until **16** was completely dissolved. K_2_CO_3_ (0.376 g, 2.72 mmol, 4.1 equiv) was added in two portions, followed by 5 min stirring at room temperature and dropwise addition of 1-bromo-2-methylpropane (98 μL, 0.91 mmol, 1.38 equiv). The reaction mixture was heated to 60 °C and left to stir overnight. After 18 h, 10 mL of H_2_O was added, and the contents of the flask were transferred to a separatory funnel and extracted with EtOAc (3 × 20 mL). Combined organic phases were washed with brine (10 mL), dried with Na_2_SO_4_, and evaporated under reduced pressure. The crude was purified by flash column chromatography using EtOAc-hexane (1:2, v/v) to obtain methyl 5-(2,4-difluorophenoxy)-1-isobutyl-1*H*-indazole-6-carboxylate (**26**) as a white viscous oil (0.109 g, 53% yield). *R*_f_ = 0.58 (EtOAc/hexane 1:2); ^1^H NMR (400 MHz, CDCl_3_, ppm) δ 8.03 (s, 1H), 7.94 (d, *J* = 1.0 Hz, 1H), 7.27 (s, 1H), 6.97 (ddd, *J* = 10.9, 8.3, 2.9 Hz, 1H), 6.85 (td, *J* = 9.0, 5.5 Hz, 1H), 6.77 (m, 1H), 4.22 (d, *J* = 7.4 Hz, 2H), 3.88 (s, 3H), 2.37 (hept, *J* = 6.9 Hz, 1H), 0.95 (d, *J* = 6.7 Hz, 6H); LC-MS (ESI^+^) *m*/*z*: calcd for C_19_H_18_F_2_N_2_O_3_ [M + H]^+^: 360.4; found: 361.9.

##### 4.1.9.1 Methyl-1-(cyclopropylmethyl)-5-(2,4-difluorophenoxy)-1*H*-indazole-6-carboxylate (27)

The compound was prepared according to the representative synthetic procedure described above. Compound **27** was synthesized with a yield of 46%. *R*_f_ = 0.72 (EtOAc/hexane 1:1). ^1^H NMR (400 MHz, CDCl_3_, ppm) δ 8.07 (s, 1H), 7.94 (d, *J* = 1.0 Hz, 1H), 7.27 (s, 1H), 6.96 (ddd, *J* = 10.9, 8.4, 2.9 Hz, 1H), 6.84 (td, *J* = 9.0, 5.5 Hz, 1H), 6.80–6.73 (m, 1H), 4.31 (d, *J* = 6.9 Hz, 2H), 3.88 (s, 3H), 1.44–1.31 (m, 1H), 0.69–0.58 (m, 2H), 0.51–0.38 (m, 2H).

##### 4.1.9.2 Methyl-1-(cyclohexylmethyl)-5-(2,4-difluorophenoxy)-1*H*-indazole-6-carboxylate (28)

The compound was prepared according to the representative synthetic procedure described above. Compound **28** was synthesized with a yield of 51%. *R*_f_ = 0.63 (EtOAc/hexane 1:1). ^1^H NMR (400 MHz, CDCl_3_, ppm) δ 8.02 (s, 1H), 7.93 (d, *J* = 1.0 Hz, 1H), 7.25 (s, 1H), 6.96 (ddd, *J* = 10.9, 8.4, 2.9 Hz, 1H), 6.88–6.81 (m, 1H), 6.81–6.74 (m, 1H), 4.23 (d, *J* = 7.3 Hz, 2H), 3.88 (s, 3H), 2.06–1.96 (m, 1H), 1.75–1.65 (m, 3H), 1.56 (s, 2H), 1.27–1.14 (m, 3H), 1.09–0.99 (m, 2H).

##### 4.1.9.3 Methyl-1-(cyclobutylmethyl)-5-(2,4-difluorophenoxy)-1*H*-indazole-6-carboxylate (30)

The compound was prepared according to the representative synthetic procedure described above. Compound **30** was synthesized with a yield of 27%. *R*_f_ = 0.57 (EtOAc/hexane 1:1). ^1^H NMR (400 MHz, CDCl_3_, ppm) δ 8.05 (s, 1H), 7.92 (d, *J* = 1.0 Hz, 1H), 7.25 (s, 1H), 6.96 (ddd, *J* = 10.9, 8.4, 2.9 Hz, 1H), 6.84 (td, *J* = 9.0, 5.5 Hz, 1H), 6.77 (dddd, *J* = 9.1, 7.7, 2.9, 1.5 Hz, 1H), 4.43 (d, *J* = 7.2 Hz), 3.88 (s, 3H), 2.95–2.90 (m, 1H), 2.13–2.00 (m, 2H), 1.97–1.81 (m, 4H).

##### 4.1.9.4 Methyl-5-(2,4-difluorophenoxy)-1-isopentyl-1*H*-indazole-6-carboxylate (31)

The compound was prepared according to the representative synthetic procedure described above. Compound **31** was synthesized with a yield of 46%. *R*_f_ = 0.09 (DCM/MeOH 10:1). ^1^H NMR (400 MHz, CDCl_3_, ppm) δ 8.04 (s, 1H), 7.93 (d, *J* = 1.0 Hz, 1H), 7.26 (s, 1H), 6.97 (ddd, *J* = 10.9, 8.4, 2.9 Hz, 1H), 6.84 (td, *J* = 9.0, 5.5 Hz, 1H), 6.77 (dddd, *J* = 9.1, 7.7, 2.9, 1.5 Hz, 1H), 4.44 (t, 2H), 3.88 (s, 3H), 1.84 (q, *J* = 7.6, 7.1 Hz, 1H), 1,63 (h, *J* = 13.4, 6.7 Hz, 1H), 0.99 (d, *J* = 6.6 Hz, 6H).

##### 4.1.9.5 Methyl-1-benzyl-5-(2,4-difluorophenoxy)-1*H*-indazole-6-carboxylate (32)

The compound was prepared according to the representative synthetic procedure described above. Compound **32** was synthesized with a yield of 46%. *R*_f_ = 0.09 (DCM/MeOH 10:1). ^1^H NMR (400 MHz, CDCl_3_, ppm) δ 7.99 (s, 2H), 7.39–7.29 (m, 4H), 7.25–7.21 (m, 2H), 6.96 (ddd, *J* = 10.9, 8.4, 2.9 Hz, 1H), 6.89–6.81 (m, 1H), 6.81–6.74 (m, 1H), 5.63 (s, 2H), 3.85 (s, 3H).

##### 4.1.9.6 Methyl-5-(4-fluorophenoxy)-1-isobutyl-1*H*-indazole-6-carboxylate (34)

The compound was prepared according to the representative synthetic procedure described above. Compound **34** was synthesized with a yield of 42%. *R*_f_ = 0.55 (EtOAc/hexane 1:2). ^1^H NMR (400 MHz, CDCl_3_, ppm) δ 0,96 (d, *J* = 6.6 Hz, 6H), 2.38 (hept, *J* = 6.8 Hz, 1H), 3,84 (s, 3H), 4.22 (d, *J* = 7.4 Hz, 2H), 6.84–6.93 (m, 2H), 6.93–7.05 (m, 2H), 7.32 (d, *J* = 0.6 Hz, 1H), 7.95 (d, *J* = 1.0 Hz, 1H), 8.01 (t, *J* = 0.8 Hz, 1H).

##### 4.1.9.7 Methyl-1-isobutyl-5-(naphthalen-1-yloxy)-1*H*-indazole-6-carboxylate (35)

The compound was prepared according to the representative synthetic procedure described above. Compound **35** was synthesized with a yield of 10%. *R*_f_ = 0.45 (EtOAc/hexane 1:2). ^1^H NMR (400 MHz, CDCl_3_, ppm) δ 0.97 (d, *J* = 6.6 Hz, 6H), 2.39 (hept, *J* = 6.9 Hz, 1H), 3.63 (s, 3H), 4.24 (d, *J* = 7.4 Hz, 2H), 6.67 (dd, *J* = 7.6, 1.0 Hz, 1H), 7.26–7.34 (m, 1H), 7.38 (d, *J* = 0.6 Hz, 1H), 7.58–7.49 (m, 3H), 7.91–7.83 (m, 1H), 7.94 (d, *J* = 1.0 Hz, 1H), 8.07 (t, *J* = 0.8 Hz, 1H), 8.35–8.43 (m, 1H).

##### 4.1.9.8 Methyl-1-isobutyl-5-(naphthalen-2-yloxy)-1*H*-indazole-6-carboxylate (36)

The compound was prepared according to the representative synthetic procedure described above. Compound **36** was synthesized with a yield of 50%. *R*_f_ = 0.60 (EtOAc/hexane 1:2). ^1^H NMR (400 MHz, CDCl_3_, ppm) δ 0.98 (d, *J* = 6.7 Hz, 6H), 2.40 (hept, *J* = 6.8 Hz, 1H), 3.78 (s, 3H), 4.25 (d, *J* = 7.4 Hz, 2H), 7.07 (d, *J* = 2.5 Hz, 1H), 7.30 (dd, *J* = 8.9, 2.5 Hz, 1H), 7.39 (dddd, *J* = 18.0, 8.1, 6.8, 1.4 Hz, 2H), 7.45 (d, *J* = 0.6 Hz, 1H), 7.58–7.65 (m, 1H), 7.77–7.86 (m, 2H), 7.97 (d, *J* = 1.0 Hz, 1H), 8.08 (t, *J* = 0.8 Hz, 1H).

##### 4.1.9.9 Methyl-1-isobutyl-5-(4-methoxyphenoxy)-1*H*-indazole-6-carboxylate (37)

The compound was prepared according to the representative synthetic procedure described above. Compound **37** was synthesized with a yield of 23%. *R*_f_ = 0.79 (EtOAc/hexane 1:2). ^1^H NMR (400 MHz, CDCl_3_, ppm) δ 0.95 (d, *J* = 6.7 Hz, 6H), 2.37 (hept, *J* = 6.8 Hz, 1H), 3.79 (s, 3H), 3.86 (s, 3H), 4.21 (d, *J* = 7.4 Hz, 2H), 6.83–6.88 (m, 2H), 6.90–6.94 (m, 2H), 7.25 (d, *J* = 8.0 Hz, 1H), 7.90 (d, *J* = 1.0 Hz, 1H), 7.97 (t, *J* = 0.8 Hz, 1H).

##### 4.1.9.10 Methyl-1-isobutyl-5-((2,4-difluorophenyl)­thio)-1*H*-indazole-6-carboxylate (38)

The compound was prepared according to the representative synthetic procedure described above. Compound **38** was synthesized with a yield of 44%. *R*_f_ = 0.63 (EtOAc/hexane 1:1). ^1^H NMR (400 MHz, CDCl_3_, ppm) δ 8.07 (s, 1H), 7.85 (d, *J* = 1.0 Hz, 1H), 7.56–7.47 (m, 1H), 7.20 (s, 1H), 6.99–6.91 (m, 2H), 4.18 (d, *J* = 7.3 Hz, 2H), 4.00 (s, 3H), 2.34 (hept, *J* = 6.8 Hz, 1H), 1.26 (t, *J* = 7.1 Hz, 2H), 0.93 (d, *J* = 6.7 Hz, 6H).

##### 4.1.9.11 Methyl-5-(2,4-difluorophenoxy)-2-isobutyl-2*H*-indazole-6-carboxylate (42)

The compound was prepared according to the representative synthetic procedure described above. Compound **42** was synthesized with a yield of 32%. *R*_f_ = 0.48 (EtOAc/hexane 1:2). ^1^H NMR (400 MHz, CDCl_3_, ppm) δ 8.36 (s, 1H), 7.81 (d, *J* = 1.0 Hz, 1H), 7.12 (s, 1H), 6.96 (ddd, *J* = 10.5, 8.4, 2.9 Hz, 1H), 6.89 (td, *J* = 9.0, 5.4 Hz, 1H), 6.77 (dddd, *J* = 9.2, 7.8, 3.0, 0.7 Hz, 1H), 4.22 (d, *J* = 7.3 Hz, 2H), 3.86 (s, 3H), 2.39 (hept, *J* = 6.9 Hz, 1H), 0.96 (d, *J* = 6.7 Hz, 6H).

##### 4.1.9.12 Methyl-2-(cyclopropylmethyl)-5-(2,4-difluorophenoxy)-2*H*-indazole-6-carboxylate (43)

The compound was prepared according to the representative synthetic procedure described above. Compound **43** was synthesized with a yield of 38%. *R*_f_ = 0.20 (EtOAc/hexane 1:2). ^1^H NMR (400 MHz, CDCl_3_, ppm) δ 8.54 (s, 1H), 7.92 (s, 1H), 7.88 (t, *J* = 6.2 Hz, 1H), 7.06–6.99 (m, 1H), 6.96–6.93 (m, 2H), 6.87–6.78 (m, 1H), 4.27 (d, *J* = 7.2 Hz, 2H), 3.53 (d, *J* = 6.1 Hz, 2H), 2.48 (t, *J* = 6.1 Hz, 2H), 2.18 (s, 6H), 1.46–1.36 (m, 1H), 0.74–0.62 (m, 2H), 0.48–0.38 (m, 2H).

##### 4.1.9.13 Methyl-2-(cyclohexylmethyl)-5-(2,4-difluorophenoxy)-2*H*-indazole-6-carboxylate (44)

The compound was prepared according to the representative synthetic procedure described above. Compound **44** was synthesized with a yield of 38%. *R*_f_ = 0.65 (EtOAc/hexane 1:2). ^1^H NMR (400 MHz, CDCl_3_, ppm) δ 8.35 (s, 1H), 7.79 (d, *J* = 1.0 Hz, 1H), 7.11 (s, 1H), 6.95 (ddd, *J* = 10.7, 8.4, 3.0 Hz, 1H), 6.90–6.84 (m, 1H), 6.81–6.70 (m, 1H), 4.23 (d, *J* = 7.3 Hz, 2H), 3.85 (s, 3H), 2.10–1.98 (m, 1H), 1.77–1.49 (m, 5H), 1.30–1.13 (m, 3H), 1.06–0.95 (m, 2H).

##### 4.1.9.14 Methyl-2-benzyl-5-(2,4-difluorophenoxy)-2*H*-indazole-6-carboxylate (45)

The compound was prepared according to the representative synthetic procedure described above. Compound **45** was synthesized with a yield of 42%. *R*_f_ = 0.06 (DCM/MeOH 10:1). ^1^H NMR (400 MHz, CDCl_3_, ppm) δ 8.03 (s, 2H), 7.37–7.27 (m, 4H), 7.24–7.21 (m, 2H), 6.96 (ddd, *J* = 10.9, 8.4, 2.9 Hz, 1H), 6.89–6.81 (m, 1H), 6.81–6.74 (m, 1H), 5.83 (s, 2H), 3.65 (s, 3H).

##### 4.1.9.15 Methyl-5-(4-fluorophenoxy)-2-isobutyl-2*H*-indazole-6-carboxylate (46)

The compound was prepared according to the representative synthetic procedure described above. Compound **46** was synthesized with a yield of 31%. *R*_f_ = 0.35 (EtOAc/hexane 1:2). ^1^H NMR (400 MHz, CDCl_3_, ppm) δ 0.96 (d, *J* = 6.7 Hz, 6H), 2.39 (hept, *J* = 6.8 Hz, 1H), 3.81 (s, 3H), 4.22 (d, *J* = 7.3 Hz, 2H), 6.87–6.95 (m, 2H), 6.95–7.04 (m, 2H), 7.19 (d, *J* = 0.7 Hz, 1H), 7.82 (d, *J* = 1.0 Hz, 1H), 8.34 (t, *J* = 0.8 Hz, 1H).

#### 4.1.10 Representative Synthetic Procedure for Compounds 49–55, 57–71

5-(2,4-Difluorophenoxy)-1-isobutyl-1*H*-indazole-6-carboxylic acid (**49**), 1-(cyclopropylmethyl)-5-(2,4-difluorophenoxy)-1*H*-indazole-6-carboxylic acid (**50**), 1-(cyclohexylmethyl)-5-(2,4-difluorophenoxy)-1*H*-indazole-6-carboxylic acid (**51**), 1-(cyclobutylmethyl)-5-(2,4-difluorophenoxy)-1*H*-indazole-6-carboxylic acid (**53**), 5-(2,4-difluorophenoxy)-1-isopentyl-1*H*-indazole-6-carboxylic acid (**54**), 1-benzyl-5-(2,4-difluorophenoxy)-1*H*-indazole-6-carboxylic acid (**55**), 5-(4-fluorophenoxy)-1-isobutyl-1*H*-indazole-6-carboxylic acid (**57**), 1-isobutyl-5-(naphthalen-1-yloxy)-1*H*-indazole-6-carboxylic acid (**58**), 1-isobutyl-5-(naphthalen-2-yloxy)-1*H*-indazole-6-carboxylic acid (**59**), 1-isobutyl-5-(4-methoxyphenoxy)-1*H*-indazole-6-carboxylic acid (**60**), 1-isobutyl-5-((2,4-difluorophenyl)­thio)-1*H*-indazole-6-carboxylic acid (**61**), 5-(2,4-difluorobenzyl)-1-isobutyl-1*H*-indazole-6-carboxylic acid (**62**), 1-(cyclohexylmethyl)-5-(2,4-difluorobenzyl)-1*H*-indazole-6-carboxylic acid (**63**), 5-(2,4-difluorobenzyl)-1-methyl-1*H*-indazole-6-carboxylic acid (**64**), 5-(2,4-difluorophenoxy)-2-isobutyl-2*H*-indazole-6-carboxylic acid (**65**), 2-(cyclopropylmethyl)-5-(2,4-difluorophenoxy)-2*H*-indazole-6-carboxylic acid (**66**), 2-(cyclohexylmethyl)-5-(2,4-difluorophenoxy)-2*H*-indazole-6-carboxylic acid (**67**), 2-benzyl-5-(2,4-difluorophenoxy)-2*H*-indazole-6-carboxylic acid (**68**), 5-(4-fluorophenoxy)-2-isobutyl-2*H*-indazole-6-carboxylic acid (**69**), 2-(cyclohexylmethyl)-5-(2,4-difluorobenzyl)-2*H*-indazole-6-carboxylic acid (**70**), and 5-(2,4-difluorobenzyl)-2-methyl-2*H*-indazole-6-carboxylic acid (**71**).

In a 50 mL round-bottom flask equipped with a stirring bar, methyl 5-(2,4-difluorophenoxy)-1-isobutyl-1*H*-indazole-6-carboxylate (**26**) (0.107 g, 0.28 mmol, 1.0 equiv) was dissolved in 12 mL of THF. One M aqueous LiOH (890 μL, 0.89 mmol, 3.0 equiv) was added dropwise during vigorous stirring of the solution at room temperature. The reaction mixture was then heated, left to stir under a reflux condenser, and monitored by TLC. After 16 h, the mixture was left to cool, and THF was evaporated. The remaining aqueous phase was acidified by dropwise addition of 1 M HCl to pH = 0, transferred to a separatory funnel, and the product extracted with EtOAc (3 × 20 mL). Combined organic phases were washed with 10 mL of brine, dried with Na_2_SO_4_, and evaporated on a rotary evaporator to yield 5-(2,4-difluorophenoxy)-1-isobutyl-1*H*-indazole-6-carboxylic acid (**49**) as an off-white viscous oil (0.086 g, 82% yield), which was used in the next step without further purification. *R*_f_ = 0.43 (CH_2_Cl_2_/MeOH 10:1). LC-MS (ESI^+^) *m*/*z*: calcd for C_18_H_16_F_2_N_2_O_3_ [M + H]^+^: 346.3; found: 347.9.

#### 4.1.11 5-(2,4-Difluorophenoxy)-*N*-(2-(dimethylamino)­ethyl)-1-isobutyl-1*H*-indazole-6-carboxamide (72)

Compound **72** was prepared from 5-(2,4-difluorophenoxy)-1-isobutyl-1*H*-indazole-6-carboxylic acid (0.084 g, 0.24 mmol, 1.0 equiv), Et_3_N (68 μL, 0.46 mmol, 2.0 equiv), TBTU (0.078 g, 0.24 mmol, 1.0 equiv), and *N*,*N*-dimethylethylenediamine (53.0 μL, 0.46 mmol, 2.0 equiv) by the procedure used for the synthesis of **87**. The crude product was purified with flash column chromatography using CH_2_Cl_2_-MeOH (1:2, v/v) to obtain 0.050 g of a white solid (50% yield). *R*_*f*_ = 0.39 (CH_2_Cl_2_/MeOH:1); ^1^H NMR (400 MHz, CDCl_3_, ppm) δ 8.33 (s, 1H), 8.20 (s, 1H), 7.88 (d, *J* = 1.1 Hz, 1H), 7.09 (s, 1H), 7.06–6.95 (m, 2H), 6.86 (m, 1H), 4.23 (d, *J* = 7.4 Hz, 2H), 3.56 (td, *J* = 6.1, 4.8 Hz, 2H), 2.50 (t, *J* = 6.1 Hz, 2H), 2.37 (hept, *J* = 6.9 Hz, 1H), 2.18 (s, 6H), 0.93 (d, *J* = 6.7 Hz, 6H); ^13^C NMR (101 MHz, CDCl_3_, ppm) δ 164.79, 160.02 (d, *J* = 10.3 Hz), 157.58 (d, *J* = 10.1 Hz), 155.00 (d, *J* = 12.0 Hz), 152.49 (d, *J* = 12.1 Hz), 149.21, 140.05 (d, *J* = 7.9 Hz), 136.54, 131.96, 125.05, 124.19, 121.74 (d, *J* = 8.9 Hz), 113.48, 111.50 (dd, *J* = 22.9, 3.9 Hz), 107.78, 105.56 (dd, *J* = 26.9, 21.8 Hz), 57.32, 56.69, 44.88, 37.65, 29.57, 20.14; HRMS (ESI^+^): *m*/*z* calcd for C_22_H_26_F_2_N_4_O_2_ [M + H]^+^: 417.2097; found: 417.2090; UPLC purity: 99%.

#### 4.1.12 1-(Cyclopropylmethyl)-5-(2,4-difluorophenoxy)-*N*-(2-(dimethylamino)­ethyl)-1*H*-indazole-6-carboxamide (73)

Compound **73** was prepared from 5-(2,4-difluorophenoxy)-1-cyclopropylmethyl-1*H*-indazole-6-carboxylic acid (0.054 g, 0.16 mmol, 1.0 equiv), Et_3_N (44 μL, 0.32 mmol, 2.0 equiv), TBTU (0.051 g, 0.16 mmol, 1.0 equiv), and *N*,*N*-dimethylethylenediamine (34 μL, 0.32 mmol, 2.0 equiv) by the procedure used for the synthesis of **87**. The crude product was used in reverse-phase preparative chromatography (Biotage Sfär, C18 D 30 g column) using 0.1% TFA­(aq)/MeCN as a mobile phase to obtain a white solid (41% yield). *R*_f_ = 0.20 (EtOAc/MeOH 2:1); ^1^H NMR (400 MHz, CDCl_3_) δ 8.36 (s, 1H), 8.21 (s, 1H), 7.88 (d, *J* = 1.1 Hz, 1H), 7.10 (s, 1H), 7.05–6.96 (m, 2H), 6.88–6.82 (m, 1H), 4.31 (d, *J* = 6.9 Hz, 2H), 3.55 (td, *J* = 6.2, 4.8 Hz, 2H), 2.48 (t, *J* = 6.1 Hz, 2H), 2.16 (s, 6H), 1.44–1.32 (m, 1H), 0.65–0.54 (m, 2H), 0.43 (dt, *J* = 6.2, 4.8 Hz, 2H); ^13^C NMR (101 MHz, CDCl_3_, ppm) δ 164.74, 159.99 (d, *J* = 10.1 Hz), 157.54 (d, *J* = 10.1 Hz), 154.96 (d, *J* = 12.1 Hz), 152.46 (d, *J* = 12.2 Hz), 149.21, 140.11 (dd, *J* = 11.5, 3.8 Hz), 136.15, 132.09, 125.26, 124.23, 121.66 (dd, *J* = 9.6, 2.0 Hz), 113.43, 111.49 (dd, *J* = 22.9, 3.9 Hz), 107.92, 105.56 (dd, *J* = 26.9, 21.8 Hz), 57.31, 53.95, 44.88, 37.67; HRMS (ESI^+^): *m*/*z* calcd for C_22_H_24_F_2_N_4_O_2_ [M + H]^+^: 415.1867; found: 415.1929; UPLC purity: 99%.

#### 4.1.13 1-(Cyclopropylmethyl)-5-(2,4-difluorophenoxy)-*N*-(3-(dimethylamino)­propyl)-1*H*-indazole-6-carboxamide (74)

Compound **74** was prepared from 5-(2,4-difluorophenoxy)-1-cyclopropylmethyl-1*H*-indazole-6-carboxylic acid (0.166 g, 0.48 mmol, 1.0 equiv), Et_3_N (135 μL, 0.96 mmol, 2.0 equiv), TBTU (0.157 g, 0.48 mmol, 1.0 equiv), and *N*,*N*-dimethylpropylene diamine (122 μL, 1.44 mmol, 3.0 equiv) by the procedure used for the synthesis of **87**. The crude product was obtained using reverse-phase preparative chromatography (Biotage Sfär, C18 D 30 g column) using 0.1% TFA­(aq)/MeCN as a mobile phase to obtain a bright yellow solid (32% yield). *R*_f_ = 0.08 (EtOAc/MeOH 2:1); ^1^H NMR (400 MHz, CDCl_3_, ppm) δ 8.38 (s, 1H), 8.18 (t, *J* = 5.6 Hz, 1H), 7.87 (d, *J* = 1.0 Hz, 1H), 7.09 (td, *J* = 9.0, 5.4 Hz, 1H), 7.03 (s, 1H), 7.03–6.99 (m, 1H), 6.91 (m, 1H), 4.31 (d, *J* = 6.9 Hz, 2H), 3.58 (q, *J* = 6.7 Hz, 2H), 2.37 (t, *J* = 6.8 Hz, 2H), 2.14 (s, 6H), 1.77 (p, *J* = 6.7 Hz, 2H), 1.42–1.31 (m, 1H), 0.64–0.54 (m, 2H), 0.44 (dt, *J* = 6.1, 4.7 Hz, 2H); ^13^C NMR (101 MHz, CDCl_3_, ppm) δ 164.75, 160.37 (d, *J* = 10.3 Hz), 157.91 (d, *J* = 10.2 Hz), 155.28 (d, *J* = 12.3 Hz), 152.78 (d, *J* = 12.2 Hz), 149.70, 139.59 (dd, *J* = 11.7, 4.0 Hz), 136.03, 132.03, 125.18, 123.89, 122.69 (dd, *J* = 9.7, 2.0 Hz), 113.60, 111.76 (dd, *J* = 22.9, 3.9 Hz), 106.95, 105.77 (dd, *J* = 26.9, 21.8 Hz), 57.99, 53.93, 45.37, 39.24, 26.73; HRMS (ESI^+^): *m*/*z* calcd for C_23_H_26_F_2_N_4_O_2_ [M + H]^+^: 429.2024; found: 429.2086; UPLC purity: 99%.

#### 4.1.14 1-(Cyclohexylmethyl)-5-(2,4-difluorophenoxy)-*N*-(2-(dimethylamino)­ethyl)-1*H*-indazole-6-carboxamide (75)

Compound **75** was prepared from 5-(2,4-difluorophenoxy)-1-cyclohexylmethyl-1*H*-indazole-6-carboxylic acid (0.075 g, 0.19 mmol, 1.0 equiv), Et_3_N (54 μL, 0.38 mmol, 2.0 equiv), TBTU (0.062 g, 0.19 mmol, 1.0 equiv), and *N*,*N*-dimethylethylenediamine (43 μL, 0.38 mmol, 2.0 equiv) by the procedure used for the synthesis of **87**. The crude product was obtained using reverse-phase preparative chromatography (Biotage Sfär, C18 D 30 g column) using 0.1% TFA­(aq)/MeCN as a mobile phase to obtain a white solid (67% yield). *R*_f_ = 0.39 (EtOAc/MeOH 2:1); ^1^H NMR (400 MHz, CDCl_3_, ppm) δ 8.33 (s, 1H), 8.21 (s, 1H), 7.87 (d, *J* = 1.0 Hz, 1H), 7.09 (s, 1H), 7.05–6.97 (m, 2H), 6.86 (m, 1H), 4.25 (d, *J* = 7.3 Hz, 2H), 3.56 (q, *J* = 6.2, 4.8 Hz, 2H), 2.49 (t, *J* = 6.1 Hz, 2H), 2.17 (s, 6H), 2.06–1.97 (m, 1H), 1.74–1.67 (m, 3H), 1.57 (d, *J* = 12.8 Hz, 2H), 1.23–1.14 (m, 3H), 1.10–0.98 (m, 2H); ^13^C NMR (101 MHz, CDCl_3_, ppm) δ 165.23, 160.44 (d, *J* = 10.2 Hz), 158.00 (d, *J* = 10.2 Hz), 155.43 (d, *J* = 12.2 Hz), 152.92 (d, *J* = 12.2 Hz), 149.62, 140.52 (dd, *J* = 11.6, 3.9 Hz), 137.07, 132.34, 125.42, 124.61, 122.14 (dd, *J* = 9.6, 2.0 Hz), 113.97, 111.94 (dd, *J* = 22.8, 4.0 Hz), 108.23, 106.00 (dd, *J* = 26.9, 21.8 Hz), 57.76, 56.03, 45.33, 39.18, 38.12, 31.28, 26.68, 26.07; HRMS (ESI^+^): *m*/*z* calcd for C_25_H_30_F_2_N_4_O_2_ [M + H]^+^: 457.2337; found: 457.2397; UPLC purity: 100%.

#### 4.1.15 1-(Cyclohexylmethyl)-5-(2,4-difluorophenoxy)-*N*-(3-(dimethylamino)­propyl)-1*H*-indazole-6-carboxamide (76)

Compound **76** was prepared from 5-(2,4-difluorophenoxy)-1-cyclohexylmethyl-1*H*-indazole-6-carboxylic acid (0.100 g, 0.26 mmol, 1.0 equiv), Et_3_N (72 μL, 0.52 mmol, 2.0 equiv), TBTU (0.083 g, 0.26 mmol, 1.0 equiv), and *N*,*N*-dimethylpropylene diamine (64 μL, 0.52 mmol, 2.0 equiv) by the procedure used for the synthesis of **87**. The crude product was obtained using reverse-phase preparative chromatography (Biotage Sfär, C18 D 30 g column) using 0.1% TFA­(aq)/MeCN as a mobile phase to obtain a white solid (30% yield). *R*_f_ = 0.28 (CH_2_Cl_2_/MeOH 9:1); ^1^H NMR (400 MHz, CDCl_3_, ppm) δ 8.34 (s, 1H), 8.16 (s, 1H), 7.85 (d, *J* = 1.0 Hz, 1H), 7.09 (td, *J* = 9.0, 5.4 Hz, 1H), 7.05–7.02 (m, 1H), 7.01 (s, 1H), 6.90 (m, 1H), 4.25 (d, *J* = 7.3 Hz, 2H), 3.58 (q, *J* = 6.6 Hz, 2H), 2.37 (t, *J* = 6.8 Hz, 2H), 2.14 (s, 6H), 2.06–1.96 (m, 1H), 1.78 (p, *J* = 6.7 Hz, 2H), 1.72–1.63 (m, 3H), 1.59–1.53 (m, 2H), 1.26–1.13 (m, 3H), 1.10–0.98 (m, 2H); ^13^C NMR (101 MHz, CDCl_3_, ppm) δ 164.81, 160.43 (d, *J* = 11.2 Hz), 157.88 (d, *J* = 10.0 Hz), 155.25 (d, *J* = 11.2 Hz), 152.80 (d, *J* = 12.5 Hz), 149.67, 139.56 (d, *J* = 11.8 Hz), 136.51, 131.83, 124.91, 123.79, 122.73 (d, *J* = 9.0 Hz), 113.71, 111.77 (dd, *J* = 22.9, 4.0 Hz), 106.82, 105.78 (dd, *J* = 27.0, 21.8 Hz), 57.96, 55.58, 45.36, 39.21, 38.73, 30.84, 26.74, 26.24, 25.63; HRMS (ESI^+^): *m*/*z* calcd for C_26_H_32_F_2_N_4_O_2_ [M + H]^+^: 471.2571; found: 471.2560; UPLC purity: 99%.

#### 4.1.16 *tert*-Butyl 3-hydroxypiperidine-1-carboxylate (33b)

To a 100 mL round-bottom flask equipped with a stirring bar, piperidin-3-ol (**33a**) (1.000 g, 9.89 mmol, 1.0 equiv) was added and dissolved in 60 mL of CH_3_CN. Di-*tert*-butyl dicarbonate (2.156 g, 9.89 mmol, 1.0 equiv) was added in two portions, and the reaction mixture was left to stir overnight at room temperature. After 20 h, CH_3_CN was evaporated, and the crude was filtered through a 30 mL pad of silica using CH_2_Cl_2_–MeOH (10:1, v/v) as an eluent, which was then evaporated to yield a viscous colorless oil (1.262 g, 63% yield). *R*_f_ = 0.67 (EtOAc/MeOH 2:1); ^1^H NMR (400 MHz, CDCl_3_, ppm) δ 3.78–3.68 (m, 2H), 3.52 (s, 1H), 3.10 (dd, *J* = 17.6, 9.2 Hz, 2H), 1.93–1.83 (m, 1H), 1.81–1.70 (m, 1H), 1.62–1.61 (m, 1H), 1.57–1.48 (m, 2H), 1.46 (s, 9H).

#### 4.1.17 *tert*-Butyl 3-methoxypiperidine-1-carboxylate (33c)

To an oven-dried 50 mL round-bottom flask equipped with a stirring bar, *tert*-butyl 3-hydroxypiperidine-1-carboxylate (**33b**) (1.255 g, 6.24 mmol, 1.0 equiv) was added, dissolved in 30 mL of anhydrous THF under argon, and cooled to 0 °C on an ice bath. Subsequently, NaH (60% dispersion in mineral oil, 0.090 mg, 6.24 mmol, 1.0 equiv) was added, and the reaction mixture was left to stir at 0 °C for 30 min. After that, methyl iodide (457 μL, 7.34 mmol, 1.2 equiv) was added dropwise. The ice bath was removed, and the reaction was left to stir overnight. After 16 h, THF was evaporated the crude was dissolved in 30 mL of EtOAc, washed with 20 mL of brine, dried with Na_2_SO_4_, and the organic phases evaporated to produce a viscous brown oil (1.043 g, 78% yield). *R*_f_ = 0.65 (CH_2_Cl_2_/MeOH 30:1); ^1^H NMR (400 MHz, CDCl_3_, ppm) δ 3.98–3.68 (m, 1H), 3.64–3.56 (m, 1H), 3.38 (s, 3H), 3.20 (dq, *J* = 7.7, 3.8 Hz, 1H), 3.14–2.98 (m, 2H), 1.98–1.88 (m, 1H), 1.79–1.70 (m, 1H), 1.46 (s, 9H), 1.44–1.37 (m, 2H).

#### 4.1.18 3-Methoxypiperidin-1-ium Chloride (33d)

To a 50 mL round-bottom flask with a stirring bar was added *tert*-butyl 3-methoxypiperidine-1-carboxylate (**33c**) (1.040 g, 5.17 mmol, 1.0 equiv) was added and dissolved in 20 mL of CH_2_Cl_2_. The flask was flushed with argon, and 4 M HCl in dioxane (3.0 mL, 12.92 mmol, 2.5 equiv) was added dropwise. The reaction mixture was left to stir at room temperature overnight. After 16 h, volatiles were evaporated on a water pump, and the crude was further dried on an oil pump for 1 h to yield a viscous yellow oil (0.780 g, 100% yield), which was used in the next step without further purification. *R*_f_ = 0.18 (CH_2_Cl_2_/MeOH 10:1); ^1^H NMR (400 MHz, CDCl_3_, ppm) δ 9.79 (s, 1H), 9.25 (s, 1H), 3.66 (dq, *J* = 7.2, 3.6 Hz, 1H), 3.41 (s, 3H), 3.38–3.27 (m, 1H), 3.25–3.15 (m, 1H), 3.10–2.92 (m, 2H), 2.11–2.00 (m, 1H), 1.98–1.90 (m, 1H), 1.89–1.80 (m, 1H), 1.66 (m, 1H).

#### 4.1.19 *tert*-Butyl­(2-(3-methoxypiperidin-1-yl)­ethyl)­carbamate (33e)

To a 50 mL round-bottom flask equipped with a stirring bar, 3-methoxypiperidin-1-ium chloride (**33d**) (0.500 g, 3.30 mmol, 1.0 equiv) was added and dissolved in 12 mL of DMSO. DIPEA (1242 μL, 7.13 mmol, 2.2 equiv) was added dropwise, followed by the addition of *tert*-butyl (2-chloroethyl)­carbamate (0.592 g, 3.30 mmol, 1.0 equiv) in three portions. The reaction mixture was warmed to 60 °C and left to stir overnight. After 18 h, DMSO and DIPEA were evaporated on an oil pump, and the crude was purified by flash column chromatography using CH_2_Cl_2_–MeOH (10:1, v/v) as an eluent to produce a viscous brown oil (0.223 g, 26% yield). *R*_f_ = 0.25 (CH_2_Cl_2_/MeOH 10:1); ^1^H NMR (400 MHz, DMSO-*d*_6_, ppm) δ 6.62 (t, *J* = 5.8 Hz, 1H), 3.23 (s, 3H), 3.20–3.11 (m, 1H), 3.02 (q, *J* = 6.6 Hz, 2H), 2.91 (dd, *J* = 11.0, 3.7 Hz, 1H), 2.66–2.57 (m, 1H), 2.35–2.27 (m, 2H), 1.94–1.84 (m, 2H), 1.79 + (t, *J* = 9.7 Hz, 1H), 1.65–1.57 (m, 1H), 1.43–1.41 (m, 1H), 1.37 (s, 9H), 1.05 (tdd, *J* = 12.0, 9.7, 4.2 Hz, 1H).

#### 4.1.20 2-(3-Methoxypiperidin-1-yl)­ethan-1-aminium Chloride (33f)

The synthetic procedure is identical to that for compound **33e**. After evaporation of HCl in dioxane and CH_2_Cl_2_, the dark brown viscous oil was dried on an oil pump for 1 h and used in the next step without further purification. *R*_f_ = 0.05 (CH_2_Cl_2_/MeOH 10:1); ^1^H NMR (400 MHz, CDCl_3_, ppm) δ 8.59–8.41 (m, 3H), 4.33–4.25 (m, 1H), 3.71–3.54 (m, 3H), 3.48–3.42 (m, 1H), 3.39–3.36 (m, 2H), 3.17 (s, 3H), 3.04–2.87 (m, 1H), 2.75 (d, *J* = 9.6 Hz, 1H), 2.10 (d, *J* = 12.3 Hz, 1H), 1.93–1.76 (m, 2H), 1.70–1.48 (m, 1H).

#### 4.1.21 1-(Cyclohexylmethyl)-5-(2,4-difluorophenoxy)-*N*-(2-(3-methoxypiperidin-1-yl)­ethyl)-1*H*-indazole-6-carboxamide (78)

To a 25 mL round-bottom flask equipped with a stirring bar, 2-(3-methoxypiperidin-1-yl)­ethan-1-aminium chloride (**33f**) (0.082 g, 0.52 mmol, 2.0 equiv) was added. CH_2_Cl_2_ (15 mL) was added under argon, and a suspension was formed, which was cooled to 0 °C on an ice bath. Subsequently, Et_3_N (72 μL, 0.52 mmol, 2.0 equiv) was added dropwise, resulting in dissolution of **33f**. After 10 min, TBTU (0.083 g, 0.26 mmol, 1.0 equiv) was added in two portions and the reaction mixture was left to stir on an ice bath under argon for 1 h before 1-(cyclohexylmethyl)-5-(2,4-difluorophenoxy)-1*H*-indazole-6-carboxylic acid (**51**) (0.100 g, 0.31 mmol, 1.2 equiv) was added. Finally, the reaction mixture was left to warm to room temperature and stir for 2 days. After 46 h, the reaction mixture was washed with saturated NaHCO_3_ solution (2 × 10 mL) and brine (2 × 10 mL), dried with Na_2_SO_4_, and CH_2_Cl_2_ evaporated under reduced pressure. The crude obtained was then purified by reverse-phase preparative chromatography (Biotage Sfär, C18 D 30 g column) using 0,1% TFA­(aq)/MeCN as a mobile phase to obtain a light brown solid (0.057 g, 42% yield). *R*_f_ = 0.60 (CH_2_Cl_2_/MeOH 10:1); ^1^H NMR (400 MHz, CDCl_3_, ppm) δ 8.38 (s, 1H), 8.32 (s, 1H), 7.85 (d, *J* = 1.1 Hz, 1H), 7.14 (td, *J* = 9.0, 5.4 Hz, 1H), 7.03 (ddd, *J* = 10.3, 8.2, 3.0 Hz, 1H), 6.98 (s, 1H), 6.93 (m, 1H), 4.25 (d, *J* = 7.4 Hz, 2H), 3.62 (p, *J* = 5.5 Hz, 2H), 3.25 (d, *J* = 1.4 Hz, 3H), 2.96–2.88 (m, 2H), 2.67 (d, *J* = 11.2 Hz, 1H), 2.63–2.54 (m, 2H), 2.06–1.95 (m, 3H), 1.93–1.82 (m, 2H), 1.73–1.54 (m, 6H), 1.27–0.98 (m, 6H); ^13^C NMR (101 MHz, CDCl_3_, ppm) δ 164.61, 160.51 (d, *J* = 10.0 Hz), 158.05 (d, *J* = 10.2 Hz), 155.53 (d, *J* = 12.1 Hz), 153.02 (d, *J* = 12.2 Hz), 150.19, 139.40 (dd, *J* = 11.9, 3.9 Hz), 136.45, 131.80, 124.93, 123.52–123.09 (m), 113.71, 111.82 (dd, *J* = 22.9, 3.9 Hz), 106.37, 105.76 (dd, *J* = 26.8, 21.7 Hz), 76.21, 57.69, 56.35, 55.96, 55.58, 53.08, 38.75, 37.02, 30.83, 29.96, 26.23, 25.62, 23.15; HRMS (ESI^+^): *m*/*z* calcd for C_29_H_36_F_2_N_4_O_3_ [M + H]^+^: 527.2755; found: 527.2816; UPLC purity: 99%.

#### 4.1.22 2-(3-(Methoxymethyl)­piperidin-1-yl)­ethan-1-aminium Chloride (33g)

The synthetic procedure is identical to that used to obtain compound **33f**. *R*_f_ = 0.06 (CH_2_Cl_2_/MeOH 10:1); ^1^H NMR (400 MHz, DMSO-*d*_6_, ppm) δ 8.38 (s, 3H), 4.33–4.25 (m, 1H), 3.58–3.39 (m, 5H), 3.33–3.30 (m, 3H), 3.17 (s, 3H), 2.90–2.82 (m, 1H), 2.75 (q, *J* = 11.4 Hz, 1H), 2.30–2.20 (m, 1H), 1.90–1.80 (m, 2H), 1.71 (d, *J* = 13.4 Hz, 1H).

#### 4.1.23 (±)-1-(Cyclohexylmethyl)-5-(2,4-difluorophenoxy)-*N*-(2-(3-(methoxymethyl)­piperidin-1-yl)­ethyl)-1*H*-indazole-6-carboxamide (79)

To a 25 mL round-bottom flask equipped with a stirring bar was added 2-(3-methoxypiperidin-1-yl)­ethan-1-aminium chloride (**33f**) (0.089 g, 0.52 mmol, 2.0 equiv). CH_2_Cl_2_ (15 mL) was added under argon, and a suspension was formed, which was cooled to 0 °C on an ice bath. Subsequently, Et_3_N (72 μL, 0.52 mmol, 2.0 equiv) was added dropwise, resulting in dissolution of **33f**. After 10 min, TBTU (0.083 g, 0.26 mmol, 1.0 equiv) was added in two portions and the reaction mixture was left to stir on an ice bath under argon for 1 h before 1-(cyclohexylmethyl)-5-(2,4-difluorophenoxy)-1*H*-indazole-6-carboxylic acid (**51**) (0.100 g, 0.31 mmol, 1.2 equiv) was added. Finally, the reaction mixture was left to warm to room temperature and stir for 2 days. After 46 h, the reaction mixture was washed with saturated NaHCO_3_ solution (2 × 10 mL) and brine (2 × 10 mL), dried with Na_2_SO_4_, and CH_2_Cl_2_ evaporated under reduced pressure. The crude obtained was then purified by reverse-phase preparative chromatography (Biotage Sfär, C18 D 30 g column) using 0,1% TFA­(aq)/MeCN as a mobile phase to obtain a light brown solid (0.032 g, 24% yield). *R*_f_ = 0.56 (CH_2_Cl_2_/MeOH 10:1); ^1^H NMR (400 MHz, CDCl_3_, ppm) δ 8.35 (s, 1H), 8.31 (t, *J* = 4.7 Hz, 1H), 7.82 (d, *J* = 1.0 Hz, 1H), 7.11 (td, *J* = 9.0, 5.4 Hz, 1H), 7.00 (dt, *J* = 8.4, 2.9, 2.6 Hz, 1H), 6.97 (s, 1H), 6.89 (m, 1H), 4.23 (d, *J* = 7.3 Hz, 2H), 3.58 (dt, *J* = 7.4, 5.7 Hz, 2H), 3.24 (s, 3H), 3.11 (qd, *J* = 9.4, 6.2 Hz, 2H), 2.83 (d, *J* = 10.0 Hz, 1H), 2.73 (d, *J* = 11.1 Hz, 1H), 2.50 (t, *J* = 6.0 Hz, 2H), 2.03–1.95 (m, 1H), 1.89 (td, *J* = 11.3, 2.6 Hz, 1H), 1.75–1.61 (m, 4H), 1.53 (dd, *J* = 14.8, 10.8 Hz, 2H), 1.24–1.12 (m, 6H), 1.07–0.97 (m, 2H), 0.92–0.81 (m, 2H); ^13^C NMR (101 MHz, CDCl_3_, ppm) δ 162.71, 158.59 (d, *J* = 10.2 Hz), 156.13 (d, *J* = 10.2 Hz), 153.63 (d, *J* = 12.3 Hz), 151.12 (d, *J* = 12.3 Hz), 148.30, 137.52 (dd, *J* = 11.8, 3.9 Hz), 134.54, 129.90, 123.02, 121.67, 121.32 (dd, *J* = 9.7, 1.9 Hz), 111.73, 109.83 (dd, *J* = 22.9, 3.9 Hz), 104.50, 103.84 (dd, *J* = 26.8, 21.7 Hz), 74.24, 56.86, 55.24, 54.87, 53.66, 52.14, 36.85, 35.12, 34.56, 28.93, 25.55, 24.34, 23.73, 22.89; HRMS (ESI^+^): *m*/*z* calcd for C_30_H_38_F_2_N_4_O_3_ [M + H]^+^: 541.2912; found: 541.2971; UPLC purity: 98%.

#### 4.1.24 1-(Cyclohexylmethyl)-5-(2,4-difluorophenoxy)-*N*-(2-morpholinoethyl)-1*H*-indazole-6-carboxamide (80)

Compound **80** was prepared from 5-(2,4-difluorophenoxy)-1-cyclohexylmethyl-1*H*-indazole-6-carboxylic acid (0.100 g, 0.26 mmol, 1.0 equiv), Et_3_N (72 μL, 0.52 mmol, 2.0 equiv), TBTU (0.083 g, 0.26 mmol, 1.0 equiv), and 2-morpholinoethan-1-amine (68 μL, 0.52 mmol, 2.0 equiv) by the procedure used for the synthesis of **87**. The crude product was obtained using reverse-phase preparative chromatography (Biotage Sfär, C18 D 30 g column) using 0.1% TFA­(aq)/MeCN as a mobile phase to obtain a bright yellow solid (32% yield). ^1^H NMR (400 MHz, CDCl_3_, ppm) δ 8.40–8.36 (m, 2H), 7.85 (d, *J* = 1.0 Hz, 1H), 7.14 (td, *J* = 9.0, 5.4 Hz, 1H), 7.04 (ddd, *J* = 10.4, 8.2, 3.0 Hz, 1H), 7.00 (s, 1H), 6.97–6.90 (m, 1H), 4.25 (d, *J* = 7.3 Hz, 2H), 3.62 (d, *J* = 6.2 Hz, 2H), 3.52–3.42 (m, 4H), 2.57 (t, *J* = 6.0 Hz, 2H), 2.42 (t, *J* = 4.6 Hz, 4H), 2.06–1.94 (m, 1H), 1.75–1.63 (m, 4H), 1.63–1.49 (m, 2H), 1.24–1.12 (m, 2H), 1.10–0.97 (m, 2H); ^13^C NMR (101 MHz, CDCl_3_, ppm) δ 164.63, 160.44, 158.03 (d, *J* = 10.2 Hz), 155.43 (d, *J* = 12.1 Hz), 152.93 (d, *J* = 12.3 Hz), 150.13, 139.38 (dd, *J* = 11.8, 3.9 Hz), 136.46, 131.82, 124.97, 123.30, 123.09 (dd, *J* = 9.7, 1.8 Hz), 113.72, 111.90 (dd, *J* = 23.0, 3.9 Hz), 106.47, 105.84 (dd, *J* = 26.8, 21.7 Hz), 66.77, 56.66, 55.57, 53.22, 38.75, 36.46, 30.82, 26.22, 25.62. HRMS (ESI^+^): *m*/*z* calcd for C_27_H_32_F_2_N_4_O_3_ [M + H]^+^: 498.2442; found: 499.2510; UPLC purity: 98%.

#### 4.1.25 *N*-(2-(1*H*-Pyrrol-1-yl)­ethyl)-1-(cyclohexylmethyl)-5-(2,4-difluorophenoxy)-1*H*-indazole-6-carboxamide (81)

Compound **81** was prepared from 5-(2,4-difluorophenoxy)-1-cyclohexylmethyl-1*H*-indazole-6-carboxylic acid (0.100 g, 0.26 mmol, 1.0 equiv), Et_3_N (72 μL, 0.52 mmol, 2.0 equiv), TBTU (0.083 g, 0.26 mmol, 1.0 equiv), and 2-(1*H*-pyrrol-1-yl)­ethan-1-amine (0.057 mg, 0.52 mmol, 2.0 equiv) by the procedure used for the synthesis of **87**. The crude product was obtained using reverse-phase preparative chromatography (Biotage Sfär, C18 D 30 g column) using 0.1% TFA­(aq)/MeCN as a mobile phase to obtain a bright yellow solid (33% yield). ^1^H NMR (400 MHz, CDCl_3_, ppm) δ 8.36 (s, 1H), 7.84 (d, *J* = 1.0 Hz, 1H), 7.81 (t, *J* = 6.3 Hz, 1H), 7.07 (dd, *J* = 9.0, 5.4 Hz, 1H), 7.01 (ddd, *J* = 10.4, 6.8, 2.0 Hz, 1H), 6.95–6.89 (m, 2H), 6.64 (t, *J* = 2.1 Hz, 2H), 5.99 (t, *J* = 2.1 Hz, 2H), 4.25 (d, *J* = 7.3 Hz, 2H), 4.15 (d, *J* = 6.6 Hz, 2H), 3.84 (d, *J* = 5.5 Hz, 2H), 2.05–1.97 (m, 1H), 1.75–1.64 (m, 3H), 1.59 (s, 1H), 1.55 (d, *J* = 12.6 Hz, 2H), 1.22–1.14 (m, 2H), 1.09–0.98 (m, 2H); ^13^C NMR (101 MHz, CDCl_3_, ppm) δ 165.19, 160.79 (d, *J* = 10.2 Hz), 158.33 (d, *J* = 10.3 Hz), 155.61 (d, *J* = 12.4 Hz), 153.10 (d, *J* = 12.3 Hz), 150.25, 138.73 (dd, *J* = 11.8, 3.9 Hz), 136.40, 131.93, 125.20, 123.58 (d, *J* = 9.7 Hz), 122.57, 120.56, 113.89, 111.89 (dd, *J* = 22.9, 3.9 Hz), 108.73, 106.13, 106.10, 105.89 (d, *J* = 5.2 Hz), 105.65, 55.67, 48.86, 41.69, 38.85, 30.92, 26.32, 25.71. HRMS (ESI^+^): *m*/*z* calcd for C_27_H_28_F_2_N_4_O_2_ [M + H]^+^: 478.2180; found: 480.2203; UPLC purity: 99%.

#### 4.1.26 *N*-(2-(1*H*-Imidazol-1-yl)­ethyl)-1-(cyclohexylmethyl)-5-(2,4-difluorophenoxy)-1*H*-indazole-6-carboxamide (82)

Compound **82** was prepared from 5-(2,4-difluorophenoxy)-1-cyclohexylmethyl-1*H*-indazole-6-carboxylic acid (0.100 g, 0.26 mmol, 1.0 equiv), Et_3_N (72 μL, 0.52 mmol, 2.0 equiv), TBTU (0.083 g, 0.26 mmol, 1.0 equiv), and 2-(1*H*-imidazol-1-yl)­ethan-1-amine (0.058 mg, 0.52 mmol, 2.0 equiv) by the procedure used for the synthesis of **87** with DMF replacing CH_2_Cl_2_. The crude product was obtained using reverse-phase preparative chromatography (Biotage Sfär, C18 D 30 g column) using 0.1% TFA­(aq)/MeCN as a mobile phase to obtain a white solid (30% yield). *R*_f_ = 0.21 (CH_2_Cl_2_/MeOH 15:1); ^1^H NMR (400 MHz, CDCl_3_, ppm) δ 8.31 (s, 1H), 7.90 (t, *J* = 5.9 Hz, 1H), 7.82 (d, *J* = 1.0 Hz, 1H), 7.44 (s, 1H), 7.06 (td, *J* = 9.0, 5.4 Hz, 1H), 6.98 (ddd, *J* = 10.7, 8.2, 2.9 Hz, 1H), 6.93 (s, 1H), 6.94–6.88 (m, 3H), 4.22 (d, *J* = 7.2 Hz, 2H), 4.20 (d, *J* = 6.0 Hz, 2H), 3.81 (q, *J* = 5.9 Hz, 2H), 2.00–1.93 (m, 1H), 1.71–1.59 (m, 3H), 1.54 (d, *J* = 12.6 Hz, 2H), 1.19–1.10 (m, 3H), 1.06–0.94 (m, 2H); ^13^C NMR (101 MHz, CDCl_3_) δ 165.41, 160.75 (d, *J* = 10.2 Hz), 158.29 (d, *J* = 10.3 Hz), 155.40 (d, *J* = 12.3 Hz), 152.90 (d, *J* = 12.3 Hz), 150.00, 138.48 (dd, *J* = 11.8, 4.0 Hz), 137.20, 136.30, 131.91, 129.66, 125.21, 123.38 (dd, *J* = 9.6, 1.8 Hz), 122.14, 118.83, 113.77, 111.99 (dd, *J* = 23.0, 3.9 Hz), 106.18, 105.83 (dd, *J* = 26.8, 21.9 Hz), 55.59, 46.11, 41.27, 38.75, 30.82, 26.21, 25.61; HRMS (ESI^+^): *m*/*z* calcd for C_26_H_27_F_2_N_5_O_2_ [M + H]^+^: 480.2133; found: 480.2203; UPLC purity: 99%.

#### 4.1.27 Methyl-1-(cyclohex-2-en-1-yl)-5-(2,4-difluorophenoxy)-1*H*-indazole-6-carboxylate (29)

To a 50 mL round-bottom flask with a stirring bar, compound **16** (0.400 g, 1.32 mmol, 1.0 equiv) was added. Anhydrous DMF (20 mL) was added under argon, and the contents were stirred until compound **16** was completely dissolved. K_2_CO_3_ (0.364 g, 2.63 mmol, 2.0 equiv) was added in two portions, followed by 5 min stirring at room temperature and dropwise addition of 3-bromocyclohex-1-ene (209 μL, 1.81 mmol, 1.4 equiv). The reaction mixture was heated to 60 °C, left to stir under a reflux condenser, and monitored by TLC. After 24 h, 15 mL of H_2_O was added, and the contents of the flask were transferred to a separatory funnel and extracted with EtOAc (3 × 20 mL). Combined organic phases were washed with brine (10 mL), dried with Na_2_SO_4_, and evaporated under reduced pressure. The crude was purified by flash column chromatography using EtOAc-hexane (1:1, v/v) to obtain a light-yellow oil (0.114 g, 23% yield). *R*_f_ = 0.63 (EtOAc/hexane = 1:1); ^1^H NMR (400 MHz, CDCl_3_, ppm) δ 8.13 (t, *J* = 0.8 Hz, 1H), 7.95 (d, *J* = 1.1 Hz, 1H), 7.27 (s, 1H), 6.96 (ddd, *J* = 10.9, 8.4, 2.9 Hz, 1H), 6.84 (td, *J* = 9.0, 5.5 Hz, 1H), 6.77 (m, 1H), 6.15 (dq, *J* = 9.9, 3.3 Hz, 1H), 5.86 (dd, *J* = 10.1, 2.4 Hz, 1H), 5.31 (ddt, *J* = 8.5, 6.1, 2.9 Hz, 1H), 3.87 (s, 3H), 2.30 (dtt, *J* = 17.8, 5.8, 2.8 Hz, 1H), 2.23–2.13 (m, 2H), 2.13–2.04 (m, 1H), 1.97 (dt, *J* = 16.1, 6.2 Hz, 1H), 1.80 (m, 1H); LC-MS (ESI^+^): *m*/*z* calcd for C_21_H_18_F_2_N_2_O_3_ [M + H]^+^: 385.1; found: 385.8.

#### 4.1.28 1-(Cyclohex-2-en-1-yl)-5-(2,4-difluorophenoxy)-1*H*-indazole-6-carboxylic Acid (52)

In a 50 mL round-bottom flask equipped with a stirring bar, methyl 5-(2,4-difluorophenoxy)-1-(cyclohex-2-en-1-yl)-1*H*-indazole-6-carboxylate (**29**) (0.114 g, 0.30 mmol, 1.0 equiv) was dissolved in 12 mL of THF. 1 M aqueous LiOH (886 μL, 0.89 mmol, 3.0 equiv) was added dropwise during vigorous stirring of the solution at room temperature. The reaction mixture was then heated, left to stir under a reflux condenser, and monitored by TLC. After 16 h, the mixture was left to cool, and THF was evaporated. The remaining aqueous phase was acidified by dropwise addition of 1 M HCl to pH = 0, transferred to a separatory funnel, and the product extracted with EtOAc (3 × 20 mL). Combined organic phases were washed with 10 mL of brine, dried with Na_2_SO_4_, and evaporated on a rotary evaporator to yield a light-yellow solid (0.072 g, 65% yield). *R*_f_ = 0.36 (CH_2_Cl_2_/MeOH 10:1); ^1^H NMR (400 MHz, DMSO-*d*_6_, ppm) δ 13.10 (s, 1H), 8.25 (s, 1H), 8.07 (d, *J* = 0.9 Hz, 1H), 7.45 (dd, *J* = 8.8, 3.0 Hz, 1H), 7.41 (s, 1H), 6.99 (m, 1H), 6.91 (td, *J* = 9.2, 5.5 Hz, 1H), 6.02 (ddd, *J* = 9.8, 5.0, 2.7 Hz, 1H), 5.76 (dd, *J* = 10.0, 1.7 Hz, 1H), 5.52 (ddt, *J* = 8.4, 5.6, 2.7 Hz, 1H), 2.23–2.02 (m, 3H), 1.99–1.82 (m, 2H), 1.83–1.67 (m, 1H); LC-MS (ESI^+^): *m*/*z* calcd for C_20_H_16_F_2_N_2_O_3_ [M + H]^+^: 371.1; found: 371.9.

#### 4.1.29 1-(Cyclohex-2-en-1-yl)-5-(2,4-difluorophenoxy)-*N*-(2-(dimethylamino)­ethyl)-1*H*-indazole-6-carboxamide (83)

Compound **83** was prepared from 1-(cyclohex-2-en-1-yl)-5-(2,4-difluorophenoxy-1*H*-indazole-6-carboxylic acid (0.072 g, 0.20 mmol, 1.0 equiv), Et_3_N (82 μL, 0.56 mmol, 2.0 equiv), TBTU (0.075 g, 0.23 mmol, 1.1 equiv), and *N*,*N*-dimethylethylenediamine (43 μL, 0.40 mmol, 2.0 equiv) by the procedure used for the synthesis of **87**. The crude product was used in reverse-phase preparative chromatography (Biotage Sfär, C18 D 30 g column) using 0.1% TFA­(aq)/MeCN as a mobile phase to obtain a bright yellow solid (70% yield). *R*_f_ = 0.30 (EtOAc/MeOH 2:1); ^1^H NMR (400 MHz, CDCl_3_, ppm) δ 8.43 (s, 1H), 8.19 (t, *J* = 4.8 Hz, 1H), 7.90 (d, *J* = 1.0 Hz, 1H), 7.11 (s, 1H), 7.04–6.95 (m, 2H), 6.86 (m, 1H), 6.13 (ddt, *J* = 9.9, 5.0, 2.8 Hz, 1H), 5.85 (dd, *J* = 10.3, 2.3 Hz, 1H), 5.34 (ddt, *J* = 8.5, 5.6, 2.8 Hz, 1H), 3.55 (td, *J* = 6.1, 4.8 Hz, 2H), 2.48 (t, *J* = 6.1 Hz, 2H), 2.35–2.23 (m, 1H), 2.16 (s, 6H), 2.15–2.12 (m, 2H), 2.09–2.05 (m, 1H), 1.98–1.89 (m, 1H), 1.84–1.72 (m, 1H); ^13^C NMR (101 MHz, CDCl_3_, ppm) δ 164.77, 159.96 (d, *J* = 10.0 Hz), 157.52 (d, *J* = 10.2 Hz), 154.93 (d, *J* = 12.2 Hz), 152.43 (d, *J* = 12.2 Hz), 149.16, 140.17 (dd, *J* = 11.6, 3.9 Hz), 135.63, 132.04 (d, *J* = 21.0 Hz), 126.18, 125.51, 124.08, 121.58 (dd, *J* = 9.6, 2.0 Hz), 113.77, 111.47 (dd, *J* = 22.9, 3.9 Hz), 108.01, 105.54 (dd, *J* = 26.9, 21.8 Hz), 57.32, 55.78, 44.90, 37.68, 29.83, 24.62, 20.61; HRMS (ESI^+^): *m*/*z* calcd for C_24_H_26_F_2_N_4_O_2_ [M + H]^+^: 441.2097; found: 441.2090; UPLC purity: 99%.

#### 4.1.30 1-(Cyclobutylmethyl)-5-(2,4-difluorophenoxy)-N-(2-(dimethylamino)­ethyl)-1H-indazole-6-carboxamide (84)

Compound **84** was prepared from 1-(cyclobutylmethyl)-5-(2,4-difluorophenoxy-1*H*-indazole-6-carboxylic acid (0.054 g, 0.15 mmol, 1.0 equiv), Et_3_N (42 μL, 0.30 mmol, 2.0 equiv), TBTU (0.049 g, 0.15 mmol, 1.0 equiv), and *N*,*N*-dimethylethylenediamine (33 μL, 0.30 mmol, 2.0 equiv) by the procedure used for the synthesis of **87**. The crude product was obtained using reverse-phase preparative chromatography (Biotage Sfär, C18 D 30 g column) using 0.1% TFA­(aq)/MeCN as a mobile phase to obtain a white solid (84% yield). *R*_f_ = 0.32 (CH_2_Cl_2_/MeOH = 15:1); ^1^H NMR (400 MHz, CDCl_3_, ppm) δ 8.36 (s, 1H), 8.21 (s, 1H), 7.86 (d, *J* = 1.0 Hz, 1H), 7.09 (s, 1H), 7.04–6.96 (m, 2H), 6.86 (m, 1H), 4.44 (d, *J* = 7.3 Hz, 2H), 3.55 (q, *J* = 6.1, 4.8 Hz, 2H), 2.94 (p, *J* = 7.6 Hz, 1H), 2.47 (t, *J* = 6.1 Hz, 2H), 2.16 (s, 6H), 2.08–1.99 (m, 2H), 1.94–1.80 (m, 4H); ^13^C NMR (101 MHz, CDCl_3_, ppm) δ 164.74, 159.98 (d, *J* = 10.1 Hz), 157.54 (d, *J* = 10.1 Hz), 154.97 (d, *J* = 12.2 Hz), 152.46 (d, *J* = 12.2 Hz), 149.17, 140.12 (dd, *J* = 11.6, 3.9 Hz), 136.33, 131.96, 125.06, 124.18, 121.66 (dd, *J* = 9.7, 2.0 Hz), 113.39, 111.48 (dd, *J* = 22.9, 3.9 Hz), 107.86, 105.54 (dd, *J* = 26.9, 21.8 Hz), 57.33, 54.32, 44.90, 37.69, 35.81, 26.11, 18.22; HRMS (ESI^+^): *m*/*z* calcd for C_23_H_26_F_2_N_4_O_2_ [M + H]^+^: 429.2097; found: 429.2090; UPLC purity: 98%.

#### 4.1.31 5-(2,4-Difluorophenoxy)-*N*-(2-(dimethylamino)­ethyl)-1-isopentyl-1*H*-indazole-6-carboxamide (85)

Compound **85** was prepared from 1-(isopentyl)-5-(2,4-difluorophenoxy-1*H*-indazole-6-carboxylic acid (0.088 g, 0.24 mmol, 1.0 equiv), Et_3_N (68 μL, 0.49 mmol, 2.0 equiv), TBTU (0.078 g, 0.24 mmol, 1.0 equiv), and *N*,*N*-dimethylethylenediamine (53 μL, 0.49 mmol, 2.0 equiv) by the procedure used for the synthesis of **87**. The crude product was obtained using reverse-phase preparative chromatography (Biotage Sfär, C18 D 30 g column) using 0.1% TFA­(aq)/MeCN as a mobile phase to obtain a white solid (35% yield). *R*_f_ = 0.35 (CH_2_Cl_2_/MeOH 15:1); ^1^H NMR (400 MHz, CDCl_3_, ppm) δ 8.35 (s, 1H), 8.20 (s, 1H), 7.87 (d, *J* = 1.0 Hz, 1H), 7.10 (s, 1H), 7.05–6.94 (m, 2H), 6.86 (m, 1H), 4.45 (t, *J* = 7.4 Hz, 2H), 3.55 (td, *J* = 6.1, 4.8 Hz, 2H), 2.47 (t, *J* = 6.1 Hz, 2H), 2.16 (s, 6H), 1.83 (dt, *J* = 8.9, 7.0 Hz, 2H), 1.70–1.55 (m, 1H), 0.98 (d, *J* = 6.6 Hz, 6H); ^13^C NMR (101 MHz, CDCl_3_, ppm) δ 164.76, 159.98 (d, *J* = 10.3 Hz), 157.53 (d, *J* = 10.2 Hz), 154.96 (d, *J* = 12.2 Hz), 152.45 (d, *J* = 12.2 Hz), 149.17, 140.15 (dd, *J* = 11.6, 3.9 Hz), 136.04, 131.94, 125.17, 124.24, 121.62 (dd, *J* = 9.7, 1.9 Hz), 113.27, 111.48 (dd, *J* = 22.9, 3.9 Hz), 107.97, 105.55 (dd, *J* = 26.8, 21.8 Hz), 57.32, 47.78, 44.90, 38.59, 37.70, 25.81, 22.39; HRMS (ESI^+^): *m*/*z* calcd for C_23_H_28_F_2_N_4_O_2_ [M + H]^+^: 431.2253; found: 431.2247; UPLC purity: 99%.

#### 4.1.32 1-Benzyl-5-(2,4-difluorophenoxy)-*N*-(2-(dimethylamino)­ethyl)-1*H*-indazole-6-carboxamide (86)

Compound **86** was prepared from 1-benzyl-5-(2,4-difluorophenoxy-1*H*-indazole-6-carboxylic acid (0.200 g, 0.53 mmol, 1.0 equiv), Et_3_N (146 μL, 1.06 mmol, 2.0 equiv), TBTU (0.169 g, 0.53 mmol, 1.0 equiv), and *N*,*N*-dimethylethylenediamine (115 μL, 1.06 mmol, 2.0 equiv) by the procedure used for the synthesis of **87**. The crude product was obtained using reverse-phase preparative chromatography (Biotage Sfär, C18 D 30 g column) using 0.1% TFA­(aq)/MeCN as a mobile phase to obtain a light yellow solid (54% yield). *R*_f_ = 0.28 (CH_2_Cl_2_/MeOH 10:1); ^1^H NMR (400 MHz, CDCl_3_) δ 8.32 (s, 1H), 8.17 (s, 1H), 7.93 (d, *J* = 1.0 Hz, 1H), 7.33–7.27 (m, 3H), 7.25–7.22 (m, 2H), 7.09 (s, 1H), 7.05–6.93 (m, 2H), 6.86 (m, 1H), 5.63 (s, 2H), 3.53 (td, *J* = 6.1, 4.8 Hz, 2H), 2.47 (q, *J* = 6.0 Hz, 2H), 2.16 (s, 6H); ^13^C NMR (101 MHz, CDCl_3_) δ 166.81, 164.63, 160.05 (d, *J* = 10.2 Hz), 157.60 (d, *J* = 10.2 Hz), 155.00 (d, *J* = 12.2 Hz), 152.49 (d, *J* = 12.2 Hz), 149.48, 139.93 (dd, *J* = 11.6, 3.8 Hz), 136.31 (d, *J* = 16.0 Hz), 132.69, 128.79, 127.94, 127.34, 125.53, 124.54, 121.82 (dd, *J* = 9.7, 1.9 Hz), 113.39, 111.52 (dd, *J* = 22.9, 3.9 Hz), 107.80, 105.56 (dd, *J* = 26.9, 21.8 Hz), 57.25, 53.22, 44.82, 37.58; HRMS (ESI^+^): *m*/*z* calcd for C_25_H_24_F_2_N_4_O_2_ [M + H]^+^: 451.1867; found: 451.1935, UPLC purity: 98%.

#### 4.1.33 *N*-(2-(Dimethylamino)­ethyl)-5-(4-fluorophenoxy)-1-isobutyl-1*H*-indazole-6-carboxamide (88)

Compound **88** was prepared from 1-(isobutyl)-5-(4-fluorophenoxy)-1*H*-indazole-6-carboxylic acid (0.167 g, 0.51 mmol, 1.0 equiv), Et_3_N (142 μL, 1.02 mmol, 3.0 equiv), TBTU (0.163 g, 0.51 mmol, 1.0 equiv), and *N*,*N*-dimethylethylenediamine (110 μL, 1.02 mmol, 2.0 equiv) by the procedure used for the synthesis of **87**. The crude product was obtained using reverse-phase preparative chromatography (Biotage Sfär, C18 D 30 g column) using 0.1% TFA­(aq)/MeCN as a mobile phase to obtain a white solid (0.037 g, 41% yield). *R*_f_ = 0.11 (CH_2_Cl_2_/MeOH 9:1); ^1^H NMR (400 MHz, CDCl_3_, ppm) δ 8.37 (s, 1H), 8.31 (t, *J* = 4.1 Hz, 1H), 7.89 (d, *J* = 1.0 Hz, 1H), 7.19 (s, 1H), 7.08–7.01 (m, 2H), 7.00–6.95 (m, 2H), 4.24 (d, *J* = 7.4 Hz, 2H), 3.51 (td, *J* = 6.1, 4.7 Hz, 2H), 2.43 (t, *J* = 6.1 Hz, 2H), 2.36 (h, *J* = 6.9 Hz, 1H), 2.15 (s, 6H), 0.94 (d, *J* = 6.7 Hz, 6H); ^13^C NMR (101 MHz, CDCl_3_, ppm) δ 164.77, 160.09, 157.68, 153.02 (d, *J* = 2.5 Hz), 149.03, 136.65, 132.00, 125.29, 124.73, 119.57 (d, *J* = 8.2 Hz), 116.40 (d, *J* = 23.4 Hz), 113.39, 110.05, 57.30, 56.67, 44.96, 37.59, 29.58, 20.14; HRMS (ESI^+^): *m*/*z* calcd for C_22_H_27_FN_4_O_2_ [M + H]^+^: 399.2118; found: 399.2186; UPLC purity: 99%.

#### 4.1.34 *N*-(2-(Dimethylamino)­ethyl)-1-isobutyl-5-(naphthalen-1-yloxy)-1*H*-indazole-6-carboxamide (89)

Compound **89** was prepared from 1-(isobutyl)-5-(naphthalen-1-yloxy)-1*H*-indazole-6-carboxylic acid (0.056 g, 0.16 mmol, 1.0 equiv), Et_3_N (65 μL, 0.47 mmol, 3.0 equiv), TBTU (0.060 g, 0.19 mmol, 1.2 equiv), and *N*,*N*-dimethylethylenediamine (34 μL, 0.31 mmol, 2.0 equiv) by the procedure used for the synthesis of **87**. The crude product was obtained using reverse-phase preparative chromatography (Biotage Sfär, C18 D 30 g column) using 0.1% TFA­(aq)/MeCN as a mobile phase to obtain a yellow viscous oil (0.038 g, 57% yield). *R*_f_ = 0.59 (CH_2_Cl_2_/MeOH 9:1); ^1^H NMR (400 MHz, CDCl_3_. ppm) δ 8.42 (s, 1H), 8.41 (t, *J* = 4.2 Hz, 1H), 8.32–8.29 (m, 1H), 7.92–7.89 (m, 1H), 7.85 (d, *J* = 1.0 Hz, 1H), 7.64 (dd, *J* = 8.3, 1.1 Hz, 1H), 7.59–7.51 (m, 2H), 7.36 (t, *J* = 7.9 Hz, 1H), 7.21 (s, 1H), 6.82 (dd, *J* = 7.6, 0.9 Hz, 1H), 4.26 (d, *J* = 7.4 Hz, 2H), 3.46 (td, *J* = 6.2, 4.8 Hz, 2H), 2.39 (h, *J* = 7.2 Hz, 1H), 2.27 (t, *J* = 6.1 Hz, 2H), 1.86 (s, 6H), 0.95 (d, *J* = 6.5 Hz, 6H); ^13^C NMR (101 MHz, CDCl_3_, ppm) δ 164.96, 153.30, 149.16, 136.74, 134.89, 132.03, 127.83, 126.77, 126.31, 126.15, 125.81, 125.47, 124.90, 123.54, 121.75, 113.32, 112.46, 110.35, 57.12, 56.69, 44.58, 37.57, 29.59, 20.17; HRMS (ESI^+^): *m*/*z* calcd for C_26_H_30_N_4_O_2_ [M + H]^+^: 431.2369; found: 431.2438; UPLC purity: 99%.

#### 4.1.35 *N*-(2-(Dimethylamino)­ethyl)-1-isobutyl-5-(naphthalen-2-yloxy)-1*H*-indazole-6-carboxamide (90)

Compound **90** was prepared from 1-(isobutyl)-5-(naphthalen-2-yloxy)-1*H*-indazole-6-carboxylic acid (0.134 g, 0.37 mmol, 1.0 equiv), Et_3_N (155 μL, 1.12 mmol, 3.0 equiv), TBTU (0.143 g, 0.45 mmol, 1.2 equiv), and *N*,*N*-dimethylethylenediamine (81 μL, 0.74 mmol, 2.0 equiv) by the procedure used for the synthesis of **87**. The crude product was obtained using reverse-phase preparative chromatography (Biotage Sfär, C18 D 30 g column) using 0.1% TFA­(aq)/MeCN as a mobile phase to obtain a brown viscous oil (0.069 g, 43% yield). *R*_f_ = 0.50 (CH_2_Cl_2_/MeOH 9:1); ^1^H NMR (400 MHz, CDCl_3_, ppm) δ 8.42 (s, 1H), 8.37 (t, *J* = 5.2 Hz, 1H), 7.90 (d, *J* = 1.0 Hz, 1H), 7.87–7.80 (m, 2H), 7.67 (dd, *J* = 7.9, 1.5 Hz, 1H), 7.50–7.38 (m, 2H), 7.32 (s, 1H), 7.30 (dd, *J* = 8.9, 2.5 Hz, 1H), 7.24 (d, *J* = 2.5 Hz, 1H), 4.26 (d, *J* = 7.4 Hz, 2H), 3.49 (td, *J* = 6.1, 4.7 Hz, 2H), 2.43 (h, *J* = 6.9 Hz, 1H), 2.39 (t, *J* = 6.3 Hz, 2H), 2.11 (s, 6H), 0.96 (d, *J* = 6.7 Hz, 6H); ^13^C NMR (101 MHz, CDCl_3_, ppm) δ 164.83, 155.21, 148.40, 136.85, 134.19, 132.08, 130.20, 129.97, 127.72, 127.17, 126.75, 125.38, 125.09, 124.93, 119.09, 113.39, 113.18, 111.13, 57.26, 56.69, 44.95, 37.60, 29.59, 20.18; HRMS (ESI^+^): *m*/*z* calcd for C_26_H_30_N_4_O_2_ [M + H]^+^: 431.2369; found: 431.2437; UPLC purity: 98%.

#### 4.1.36 *N*-(2-(Dimethylamino)­ethyl)-1-isobutyl-5-(4-methoxyphenoxy)-1*H*-indazole-6-carboxamide (91)

Compound **91** was prepared from 1-(isobutyl)-5-(4-methoxyphenoxy)-1*H*-indazole-6-carboxylic acid (0.209 g, 0.61 mmol, 1.0 equiv), Et_3_N (257 μL, 1.84 mmol, 3.0 equiv), TBTU (0.237 g, 0.74 mmol, 1.2 equiv), and *N*,*N*-dimethylethylenediamine (134 μL, 1.23 mmol, 2.0 equiv) by the procedure used for the synthesis of **87**. The crude product was obtained using reverse-phase preparative chromatography (Biotage Sfär, C18 D 30 g column) using 0.1% TFA­(aq)/MeCN as a mobile phase to obtain a yellow viscous oil (0.088 g, 35% yield). *R*_f_ = 0.49 (CH_2_Cl_2_/MeOH 9:1); ^1^H NMR (400 MHz, CDCl_3_) δ 8.44 (s, 1H), 8.36 (s, 1H), 7.84 (s, 1H), 7.11 (s, 1H), 7.00–6.94 (m, 2H), 6.92–6.83 (m, 2H), 4.22 (d, *J* = 7.4 Hz, 2H), 3.80 (s, 3H), 3.53 (d, *J* = 6.1 Hz, 2H), 2.45 (t, *J* = 6.1 Hz, 2H), 2.35 (p, *J* = 7.1 Hz, 1H), 2.16 (s, 6H), 0.92 (d, *J* = 6.6 Hz, 6H); 13C NMR (101 MHz, CDCl3, ppm) ^13^C NMR (101 MHz, CDCl_3_) δ 165.11, 156.21, 150.32, 150.27, 136.47, 132.00, 125.42, 124.36, 120.15 (2x), 115.06 (2x), 113.40, 108.88, 57.56, 56.76, 55.81, 45.15 (2x), 37.81, 29.69, 20.26 (2x); HRMS (ESI^+^): *m*/*z* calcd for C_23_H_30_N_4_O_3_ [M + H]^+^: 411.2318; found: 411.2385; UPLC purity: 98%.

#### 4.1.37 5-((2,4-Difluorophenyl)­thio)-*N*-(2-(dimethylamino)­ethyl)-1-isobutyl-1*H*-indazole-6-carboxamide (92)

Compound **92** was prepared from 1-(isobutyl)-5-((2,4-difluorophenyl)­thio)-1*H*-indazole-6-carboxylic acid (0.189 g, 0.50 mmol, 1.0 equiv), Et_3_N (145 μL, 1.00 mmol, 2.0 equiv), TBTU (0.168 g, 0.50 mmol, 1.0 equiv), and *N*,*N*-dimethylethylenediamine (112 μL, 1.00 mmol, 2.0 equiv) by the procedure used for the synthesis of **87**. The crude product was obtained using reverse-phase preparative chromatography (Biotage Sfär, C18 D 30 g column) using 0.1% TFA­(aq)/MeCN as a mobile phase to obtain a light brown powder (0.117 g, 52% yield). *R*_f_ = 0.22 (EtOAc/MeOH 2:1); ^1^H NMR (400 MHz, CDCl_3_) δ 7.92 (s, 1H), 7.75 (d, *J* = 3.3 Hz, 2H), 7.17–7.09 (m, 2H), 6.87–6.73 (m, 2H), 4.15 (d, *J* = 7.3 Hz, 2H), 3.48 (q, *J* = 5.6 Hz, 2H), 2.47 (t, *J* = 6.0 Hz, 2H), 2.37–2.27 (m, 1H), 2.21 (s, 6H), 0.90 (d, *J* = 6.7 Hz, 6H); ^13^C NMR (101 MHz, CDCl_3_, ppm) δ 167.93, 163.66 (d, *J* = 11.2 Hz), 161.86 (d, *J* = 12.1 Hz), 161.17 (d, *J* = 11.3 Hz), 159.39 (d, *J* = 12.1 Hz), 138.85, 136.74, 133.42 (dd, *J* = 9.4, 2.8 Hz), 132.51, 127.69, 125.09, 121.43, 119.44 (dd, *J* = 17.8, 4.0 Hz), 112.22 (dd, *J* = 21.6, 3.7 Hz), 110.72, 104.47 (t, *J* = 25.9 Hz), 57.43, 56.61, 45.02, 37.55, 29.48, 20.15; HRMS (ESI^+^): *m*/*z* calcd for C_22_H_26_F_2_N_4_OS [M + H]^+^: 433.1795; found: 433.1857; UPLC purity: 96%.

#### 4.1.38 5-((2,4-Difluorophenyl)­sulfonyl)-*N*-(2-(dimethylamino)­ethyl)-1-isobutyl-1*H*-indazole-6-carboxamide (93)

To a 25 mL round-bottom flask with a stirring bar, 5-((2,4-difluorophenyl)­thio)-*N*-(2-(dimethylamino)­ethyl)-1-isobutyl-1*H*-indazole-6-carboxamide (**92**) (0.059 mg, 0.136 mmol, 1.0 equiv) was added and dissolved in methanol (12 mL). Magnesium monoperoxyphthalate hexahydrate (0.069 g, 0.140 mmol, 1.0 equiv) was added in two portions, and the reaction mixture was left to stir at room temperature for 3 h. After that, MeOH was evaporated, the crude dissolved in 20 mL of EtOAc, washed with 10 mL of saturated NaHCO_3_ solution and 10 mL of brine, dried over Na_2_SO_4_, and EtOAc evaporated under reduced pressure. The product was then purified by reverse-phase preparative chromatography (Biotage Sfär, C18 D 30 g column) using 0,1% TFA­(aq)/MeCN as a mobile phase as an eluent to yield a brown solid (0.004 g, 6% yield). *R*_f_ = 0.17 (EtOAc/MeOH 2:1); ^1^H NMR (400 MHz, CDCl_3_, ppm) δ 10.07 (s, 1H), 8.81 (s, 1H), 8.18 (s, 1H), 7.80 (s, 1H), 7.23–7.15 (m, 1H), 6.89–6.73 (m, 2H), 4.23 (d, *J* = 7.3 Hz, 2H), 3.94–3.69 (m, 2H), 3.57–3.35 (m, 2H), 3.26 (d, *J* = 18.6 Hz, 6H), 2.36 (hept, *J* = 6.7 Hz, 1H), 0.92 (d, *J* = 6.6 Hz, 6H). ^13^C NMR (101 MHz, CDCl_3_, ppm) δ 166.22, 140.04, 135.29, 134.01, 129.95, 129.54, 129.40, 125.02, 121.35, 112.41, 112.23, 109.40, 104.63 (t, *J* = 25.5 Hz), 67.43, 59.56, 56.75, 36.56, 29.84 (2x), 29.70, 20.28 (2x); HRMS (ESI^+^): *m*/*z* calcd for C_22_H_26_F_2_N_4_O_3_S [M + H]^+^: 464.1694; found:.464.2611; UPLC purity: 95%.

#### 4.1.39 Methyl-5-amino-2-bromo-4-methylbenzoate (22)

To a 50 mL round-bottom flask equipped with a stirring bar, methyl 3-amino-4-methylbenzoate (10 g, 60.536 mmol, 1.0 equiv) was added and dissolved in 40 mL of DMF. The solution was cooled to 0 °C on an ice bath, and *N*-bromosuccinimide (10.78 g, 60.536 mmol, 1.0 equiv) was added in four portions. The reaction mixture was left to stir at 0 °C while being monitored by TLC. After 1 h, DMF was evaporated on an oil pump, the product dissolved in 100 mL of EtOAc, washed with brine (2 × 50 mL), dried over Na_2_SO_4_, and the solvent evaporated. The crude was then purified by recrystallization using EtOAc-hexane (1:10, v/v) as a solvent system to yield a white solid (7.857 g, 53% yield). *R*_f_ = 0.40 (EtOAc/hexane 1:1); ^1^H NMR (400 MHz, CDCl_3_, ppm) δ 7.31 (s, 1H), 7.14 (s, 1H), 3.89 (s, 3H), 3.70 (s, 2H), 2.15 (s, 3H); HRMS (ESI^+^): *m*/*z* calcd for C_9_H_10_BrNO_2_ [M + H]^+^: 243,9968; found: 243,9968.

#### 4.1.40 Methyl-5-bromo-1*H*-indazole-6-carboxylate (23)

In a 500 mL round-bottom flask equipped with a stirring bar, methyl 5-amino-2-bromo-4-methylbenzoate (**22**) (26.157 g, 107.16 mmol, 1.0 equiv) was dissolved in 150 mL of chloroform. The solution was cooled to 0° in an ice bath, after which sodium acetate (1.046 g, 17.15 mmol, 0.16 equiv) and acetic acid (100 mL, 1746.73 mmol, 16.3 equiv) were added to the flask. After 15 min of stirring on an ice bath, *tert*-butyl nitrite (32 mL, 267.90 mmol, 1.6 equiv) was added dropwise. After an additional 15 min, the ice bath was removed and the reaction mixture was left to warm to room temperature. Once all the starting compound was consumed (monitored by TLC), the solvents were evaporated on a rotary evaporator, and the residue was dissolved in 100 mL of EtOAc, which was then carefully washed with a saturated NaHCO_3_ solution (2 × 50 mL). The organic phase was dried with Na_2_SO_4_ and evaporated under reduced pressure. The crude was purified by flash column chromatography using EtOAc–hexane (1:2, v/v) as the eluent to produce a viscous yellow oil (16.412 g, 60% yield). *R*_f_ = 0.32 (EtOAc/hexane 1:1); ^1^H NMR (400 MHz, CDCl_3_, ppm) δ 11.04 (s, 1H), 8.08 (d, *J* = 1.1 Hz, 1H), 8.06 (d, *J* = 0.6 Hz, 1H), 7.97 (t, *J* = 0.8 Hz, 1H), 3.98 (s, 3H); HRMS (ESI^+^): *m*/*z* calcd for C_9_H_7_BrN_2_O_2_ [M + H]^+^: 254.9764; found: 254.9765.

#### 4.1.41 Methyl-5-bromo-1-isobutyl-1*H*-indazole-6-carboxylate (24)

To a 25 mL round-bottom flask with a stirring bar, methyl 5-bromo-1*H*-indazole-6-carboxylate (**23**) (0.200 g, 0.78 mmol, 1.0 equiv) was added. Anhydrous DMF (20 mL) was added under argon and the contents stirred until **23** was completely dissolved. K_2_CO_3_ (0.434 g, 3.14 mmol, 4.1 equiv) was added in two portions, followed by 5 min stirring at room temperature and dropwise addition of 1-bromo-2-methylpropane (116 μL, 1.08 mmol, 1.38 equiv). The reaction mixture was heated to 60 °C and left to stir overnight. After 18 h, 10 mL of H_2_O was added, the contents of the flask were transferred to a separatory funnel, and extracted with EtOAc (3 × 20 mL). Combined organic phases were washed with brine (10 mL), dried with Na_2_SO_4_, and evaporated under reduced pressure. The crude was purified by flash column chromatography using EtOAc-hexane (1:2, v/v) to obtain a white viscous oil (0.102 g, 42% yield). *R*_f_ = 0.58 (EtOAc/hexane 1:2); ^1^H NMR (400 MHz, CDCl_3_, ppm) δ 8.02 (s, 1H), 7.98 (d, *J* = 1.0 Hz, 1H), 7.86 (s, 1H), 4.19 (d, *J* = 7.4 Hz, 2H), 3.99 (s, 3H), 2.34 (h, *J* = 6.9 Hz, 1H), 0.92 (d, *J* = 6.7 Hz, 6H); LC-MS (ESI^+^): *m*/*z* calcd for C_13_H_15_ BrN_2_O_2_ [M + H]^+^: 310.0; found: 312.9.

#### 4.1.42 2,4-Difluorobenzylzinc Bromide (25)

Zinc dust (0.525 mg, 8.034 mmol, 5.0 eq—relative to 5-bromo-1-isobutyl-1*H*-indazole-6-carboxylate (**24**) added in the next step) was added to a 50 mL round-bottom flask and suspended in 10 mL of anhydrous THF under argon. 1,2-dibromoethane (0.017 mL, 0.201 mmol, 5 mol %) was added dropwise, and the suspension was heated to reflux for 10 min. Chlorotrimethylsilane (0.010 mL, 0.080 mmol, 2 mol %) was then added dropwise to the hot suspension, which was stirred vigorously at 60 °C for an additional 15 min before it was cooled on an ice bath. 2,4-Difluorobenzyl bromide (1.031 mL, 1.607 mmol, 5.0 equiv) dissolved in ice-cold anhydrous THF (5 mL) was then added via cannula to the cooled suspension, and the reaction mixture left to warm up to room temperature and stir overnight. After 19 h, the reaction mixture proceeded to the next step without any isolation procedures.

#### 4.1.43 Representative Synthetic Procedure for Compounds 39–41, 47–48

Methyl 5-(2,4-difluorobenzyl)-1-isobutyl-1*H*-indazole-6-carboxylate (**39**), methyl 1-(cyclohexylmethyl)-5-(2,4-difluorobenzyl)-1*H*-indazole-6-carboxylate (**40**), methyl 5-(2,4-difluorobenzyl)-1-methyl-1*H*-indazole-6-carboxylate (**41**), methyl 1-(cyclohexylmethyl)-5-(2,4-difluorobenzyl)-2*H*-indazole-6-carboxylate (**47**), and methyl 5-(2,4-difluorobenzyl)-1-methyl-2*H*-indazole-6-carboxylate (**48**).

To the reaction mixture with **25** was added tetrakis­(triphenylphosphine)­palladium(0) (0.046 g, 0.040 mmol, 0.025 equiv) dissolved in 10 mL of anhydrous THF. Subsequently, 5-bromo-1-isobutyl-1*H*-indazole-6-carboxylate (**24**) (0.500 g, 1.61 mmol, 1.0 equiv) was added, and the reaction mixture was heated to 60 °C and monitored by LC-MS until all the 5-bromo-1-isobutyl-1*H*-indazole-6-carboxylate was consumed. After 4.5 h, the flask was cooled to room temperature and the reaction mixture filtered under suction through a pad of Celite 545, followed by filtration through a syringe filter (pore size: 0.45 μm, PTFE). THF was evaporated, and the crude was purified by flash column chromatography using EtOAc–hexane (1:3, v/v) as an eluent. After drying the product on an oil pump for 2 h, it was purified by reverse-phase preparative chromatography (Biotage Sfär, C18 D 30 g column) using 0.1% TFA­(aq)/MeCN as a mobile phase to yield methyl 5-(2,4-difluorobenzyl)-1-isobutyl-1*H*-indazole-6-carboxylate (**39**) as a light brown viscous oil (0.120 g, 21% yield). *R*_f_ = 0.60 (EtOAc/hexane 1:3); ^1^H NMR (400 MHz, CDCl_3_, ppm) δ 8.03 (s, 1H), 7.95 (d, *J* = 1.0 Hz, 1H), 7.49 (s, 1H), 6.94 (td, *J* = 8.6, 6.5 Hz, 1H), 6.85–6.71 (m, 2H), 4.41 (s, 2H), 4.21 (d, *J* = 7.4 Hz, 2H), 3.87 (s, 3H), 2.36 (hept, *J* = 6.9 Hz, 1H), 0.94 (d, *J* = 6.7 Hz, 6H); LC-MS (ESI^+^): *m*/*z* calcd for C_20_H_20_F_2_N_2_O_2_ [M + H]^+^: 358.2; found: 359.9.

##### 4.1.43.1 Methyl-1-(cyclohexylmethyl)-5-(2,4-difluorobenzyl)-1*H*-indazole-6-carboxylate (40)

The compound was prepared according to the representative synthetic procedure described above. Compound **40** was synthesized with a yield of 25%. *R*_f_ = 0.73 (EtOAc/hexane 1:3). ^1^H NMR (400 MHz, CDCl_3_, ppm) δ 8.02 (s, 1H), 7.95 (d, *J* = 1.0 Hz, 1H), 7.49 (s, 1H), 6.94 (td, *J* = 8.6, 6.6 Hz, 1H), 6.85–6.71 (m, 2H), 4.40 (s, 2H), 4.22 (d, *J* = 7.3 Hz, 2H), 3.87 (s, 3H), 2.06–1.96 (m, 1H), 1.73–1.65 (m, 3H), 1.61–1.57 (m, 2H), 1.26–1.14 (m, 3H), 1.10–0.97 (m, 2H).

##### 4.1.43.2 Methyl-5-(2,4-difluorobenzyl)-1-methyl-1*H*-indazole-6-carboxylate (41)

The compound was prepared according to the representative synthetic procedure described above. Compound **41** was synthesized with a yield of 29%. *R*_f_ = 0.45 (EtOAc/hexane 1:3). ^1^H NMR (400 MHz, CDCl_3_, ppm) δ 8.05 (s, 1H), 7.94 (d, *J* = 1.1 Hz, 1H), 7.50 (s, 1H), 6.92 (td, *J* = 8.6, 6.5 Hz, 1H), 6.85–6.77 (m, 1H), 6.77–6.69 (m, 1H), 4.41 (s, 2H), 4.12 (s, 3H), 3.87 (s, 3H).

##### 4.1.43.3 Methyl-1-(cyclohexylmethyl)-5-(2,4-difluorobenzyl)-2*H*-indazole-6-carboxylate (47)

The compound was prepared according to the representative synthetic procedure described above. Compound **47** was synthesized with a yield of 27%. *R*_f_ = 0.52 (EtOAc/hexane 1:3). ^1^H NMR (400 MHz, CDCl_3_, ppm) δ 8.37 (s, 1H), 7.80 (s, 1H), 7.37 (s, 1H), 6.97 (td, *J* = 8.6, 6.5 Hz, 1H), 6.87–6.67 (m, 2H), 4.38 (s, 2H), 4.24 (d, *J* = 7.3 Hz, 2H), 3.84 (s, 3H), 2.10–2.03 (m, 1H), 1.74–1.65 (m, 3H), 1.63–1.57 (m, 2H), 1.28–1.13 (m, 3H), 1.05–0.95 (m, 2H).

##### 4.1.43.4 Methyl-5-(2,4-difluorobenzyl)-1-methyl-2*H*-indazole-6-carboxylate (48)

The compound was prepared according to the representative synthetic procedure described above. Compound **48** was synthesized with a yield of 23%. *R*_f_ = 0.38 (EtOAc/hexane 1:3). ^1^H NMR (400 MHz, CDCl_3_, ppm) δ 8.35 (s, 1H), 7.83 (s, 1H), 7.37 (s, 1H), 6.95 (td, *J* = 8.6, 6.5 Hz, 1H), 6.84–6.69 (m, 2H), 4.37 (s, 2H), 4.24 (s, 3H), 3.84 (s, 3H).

#### 4.1.44 5-(2,4-Difluorobenzyl)-*N*-(2-(dimethylamino)­ethyl)-1-isobutyl-1*H*-indazole-6-carboxamide (94)

Compound **94** was prepared from 1-(isobutyl)-5-((2,4-difluorobenzyl)-1*H*-indazole-6-carboxylic acid (0.112 g, 0.33 mmol, 1.0 equiv), Et_3_N (91 μL, 0.66 mmol, 2.0 equiv), TBTU (0.105 g, 0.33 mmol, 1.0 equiv), and *N*,*N*-dimethylethylenediamine (71 μL, 0.66 mmol, 2.0 equiv) by the procedure used for the synthesis of **87**. The crude product was used in reverse-phase preparative chromatography (Biotage Sfär, C18 D 30 g column) using 0.1% TFA­(aq)/MeCN as a mobile phase to obtain a green viscous oil (0.059 g, 44% yield). *R*_f_ = 0.26 (EtOAc/MeOH 2:1); ^1^H NMR (400 MHz, CDCl_3_, ppm) δ 7.92 (d, *J* = 1.0 Hz, 1H), 7.49 (s, 1H), 7.44 (s, 1H), 7.11 (td, *J* = 8.8, 6.5 Hz, 1H), 6.81–6.68 (m, 2H), 6.40 (s, 1H), 4.24 (s, 2H), 4.15 (d, *J* = 7.3 Hz, 2H), 3.46 (q, *J* = 5.4 Hz, 2H), 2.43 (t, *J* = 5.9 Hz, 2H), 2.34 (hept, *J* = 6.9 Hz, 1H), 2.20 (s, 6H), 0.92 (d, *J* = 6.7 Hz, 6H); ^13^C NMR (101 MHz, CDCl_3_, ppm) δ 170.12, 162.79 (d, *J* = 11.4 Hz), 162.11 (d, *J* = 12.1 Hz), 160.34 (d, *J* = 11.4 Hz), 159.64 (d, *J* = 12.1 Hz), 138.01, 135.55, 132.42, 131.81 (dd, *J* = 9.3, 6.2 Hz), 129.17, 124.49, 123.97 (dd, *J* = 15.7, 3.8 Hz), 122.51 (d, *J* = 1.2 Hz), 110.91 (dd, *J* = 20.8, 3.7 Hz), 108.36, 103.58 (t, *J* = 25.6 Hz), 57.53, 56.46, 44.99, 37.17, 31.43 (d, *J* = 2.6 Hz), 29.49, 20.19; HRMS (ESI^+^): *m*/*z* calcd for C_23_H_28_F_2_N_4_O [M + H]^+^: 415.2231; found: 415.2292; UPLC purity: 98.6%.

#### 4.1.45 1-(Cyclohexylmethyl)-5-(2,4-difluorobenzyl)-*N*-(2-(dimethylamino)­ethyl)-1*H*-indazole-6-carboxamide (95)

Compound **95** was prepared from 1-(cyclohexylmethyl)-5-(2,4-difluorobenzyl)-1*H*-indazole-6-carboxylic acid (0.118 g, 0.26 mmol, 1.0 equiv), Et_3_N (86 μL, 0.52 mmol, 2.0 equiv), TBTU (0.099 g, 0.26 mmol, 1.0 equiv), and *N*,*N*-dimethylethylenediamine (67 μL, 0.52 mmol, 2.0 equiv) by the procedure used for the synthesis of **87**. The crude product was obtained using reverse-phase preparative chromatography (Biotage Sfär, C18 D 30 g column) using 0.1% TFA­(aq)/MeCN as a mobile phase to obtain a light green viscous oil (0.063 g, 53% yield). *R*_f_ = 0.35 (CH_2_Cl_2_/MeOH 10:1); ^1^H NMR (400 MHz, CDCl_3_, ppm) δ 7.89 (s, 1H), 7.46 (s, 1H), 7.41 (s, 1H), 7.10 (td, *J* = 8.8, 6.5 Hz, 1H), 6.79–6.71 (m, 2H), 6.43 (t, *J* = 5.0 Hz, 1H), 4.21 (s, 2H), 4.14 (d, *J* = 7.2 Hz, 2H), 3.42 (q, *J* = 5.6 Hz, 2H), 2.40 (t, *J* = 6.0 Hz, 2H), 2.16 (s, 6H), 1.97 (h, *J* = 3.6 Hz, 1H), 1.70–1.60 (m, 3H), 1.57–1.51 (m, 2H), 1.20–1.09 (m, 3H), 1.03–0.92 (m, 2H); ^13^C NMR (101 MHz, CDCl_3_, ppm) δ 170.13, 162.79 (d, *J* = 11.5 Hz), 162.12 (d, *J* = 12.2 Hz), 160.34 (d, *J* = 11.5 Hz), 159.65 (d, *J* = 12.2 Hz), 138.09, 135.55, 132.37, 131.81 (dd, *J* = 9.3, 6.1 Hz), 129.11, 124.44, 123.97 (dd, *J* = 15.7, 3.8 Hz), 122.66–122.37 (m), 110.91 (dd, *J* = 20.8, 3.8 Hz), 108.40, 103.59 (t, *J* = 25.6 Hz), 57.47, 55.38, 45.02, 38.72, 37.20, 31.44 (d, *J* = 2.6 Hz), 30.93, 26.24, 25.64; HRMS (ESI^+^): *m*/*z* calcd for C_26_H_32_F_2_N_4_O [M + H]^+^: 455.2544; found: 455.2602; UPLC purity: 100%.

#### 4.1.46 5-(2,4-Difluorobenzyl)-*N*-(2-(dimethylamino)­ethyl)-1-methyl-1*H*-indazole-6-carboxamide (96)

Compound **96** was prepared from 5-(2,4-difluorobenzyl)-1-methyl-1*H*-indazole-6-carboxylic acid (0.140 g, 0.46 mmol, 1.0 equiv), Et_3_N (152 μL, 0.92 mmol, 2.0 equiv), TBTU (0.175 g, 0.46 mmol, 1.0 equiv), and *N*,*N*-dimethylethylenediamine (118 μL, 0.92 mmol, 2.0 equiv) by the procedure used for the synthesis of **87**. The crude product was obtained using reverse-phase preparative chromatography (Biotage Sfär, C18 D 30 g column) using 0.1% TFA_(aq)_/MeCN as a mobile phase to obtain a light green viscous oil (0.071 g, 41% yield). ^1^H NMR (400 MHz, CDCl_3_, ppm) δ 7.81 (s, 1H), 7.74 (s, 1H), 7.38 (s, 1H), 7.13 (td, *J* = 9.2, 8.8, 6.5 Hz, 1H), 6.80–6.71 (m, 2H), 6.43 (d, *J* = 4.3 Hz, 1H), 4.24 (s, 2H), 4.21 (s, 3H), 3.41 (d, *J* = 6.2 Hz, 2H), 2.40 (t, *J* = 5.9 Hz, 2H), 2.18 (s, 6H); ^13^C NMR (101 MHz, CDCl_3_, ppm) δ 169.99, 162.82 (d, *J* = 11.9 Hz), 162.14 (d, *J* = 13.0 Hz), 160.36 (d, *J* = 11.7 Hz), 159.66 (d, *J* = 13.0 Hz), 138.14, 135.71, 132.49, 131.78 (dd, *J* = 9.4, 6.1 Hz), 129.36, 124.63, 124.00 (dd, *J* = 15.7, 3.8 Hz), 122.60 (d, *J* = 1.2 Hz), 110.89 (dd, *J* = 20.8, 3.7 Hz), 103.61 (t, *J* = 25.6 Hz), 57.47, 45.01, 37.16, 35.72, 31.54 (d, *J* = 2.6 Hz); UPLC purity: 96%.

#### 4.1.47 5-(2,4-Difluorophenoxy)-*N*-(2-(dimethylamino)­ethyl)-2-isobutyl-2*H*-indazole-6-carboxamide (97)

Compound **97** was prepared from 5-(2,4-difluorophenoxy)-2-isobutyl-2*H*-indazole-6-carboxylic acid (0.056 g, 0.16 mmol, 1.0 equiv), Et_3_N (45 μL, 0.32 mmol, 2.0 equiv), TBTU (0.052 g, 0.16 mmol, 1.0 equiv), and *N*,*N*-dimethylethylenediamine (35 μL, 0.32 mmol, 2.0 equiv) by the procedure used for the synthesis of **87**. The crude product was obtained using reverse-phase preparative chromatography (Biotage Sfär, C18 D 30 g column) using 0.1% TFA_(aq)_/MeCN as a mobile phase to obtain a bright yellow solid (0.015 g, 24% yield). *R*_f_ = 0.46 (CH_2_Cl_2_/MeOH 9:1); ^1^H NMR (400 MHz, CDCl_3_, ppm) δ 8.54 (s, 1H), 7.86 (t, *J* = 4.8 Hz, 1H), 7.77 (d, *J* = 1.0 Hz, 1H), 7.07–6.98 (m, 2H), 6.96 (s, 1H), 6.85 (m, 1H), 4.20 (d, *J* = 7.3 Hz, 2H), 3.53 (td, *J* = 6.1, 4.8 Hz, 2H), 2.46 (t, *J* = 6.1 Hz, 2H), 2.38 (h, *J* = 6.8 Hz, 1H), 2.16 (s, 6H), 0.94 (d, *J* = 6.8 Hz, 6H); ^13^C NMR (101 MHz, CDCl_3_, ppm) δ 165.23, 159.89 (d, *J* = 10.2 Hz), 157.44 (d, *J* = 10.2 Hz), 154.97 (d, *J* = 12.3 Hz), 152.47 (d, *J* = 12.2 Hz), 149.70, 145.68, 140.22 (dd, *J* = 11.6, 4.0 Hz), 126.00, 122.74 (d, *J* = 6.4 Hz), 122.15, 121.82 (dd, *J* = 9.6, 2.1 Hz), 111.38 (dd, *J* = 22.8, 3.9 Hz), 105.81, 105.41 (dd, *J* = 26.8, 21.9 Hz), 61.48, 57.43, 44.92, 37.55, 29.95, 19.92; HRMS (ESI^+^): *m*/*z* calcd for C_22_H_26_F_2_N_4_O_2_ [M + H]^+^: 417.2097; found: 417.2090; UPLC purity: 99%.

#### 4.1.48 2-(Cyclopropylmethyl)-5-(2,4-difluorophenoxy)-*N*-(2-(dimethylamino)­ethyl)-2*H*-indazole-6-carboxamide (98)

Compound **98** was prepared from 5-(2,4-difluorophenoxy)-2-cyclopropylmethyl-2*H*-indazole-6-carboxylic acid (0.054 g, 0.16 mmol, 1.0 equiv), Et_3_N (44 μL, 0.32 mmol, 2.0 equiv), TBTU (0.051 g, 0.16 mmol, 1.0 equiv), and *N*,*N*-dimethylethylenediamine (34 μL, 0.32 mmol, 2.0 equiv) by the procedure used for the synthesis of **87**. The crude product was obtained using reverse-phase preparative chromatography (Biotage Sfär, C18 D 30 g column) using 0.1% TFA_(aq)_/MeCN as a mobile phase to obtain a white solid (45% yield). *R*_f_ = 0.17 (EtOAc/MeOH 2:1); ^1^H NMR (400 MHz, CDCl_3_, ppm) δ 8.54 (s, 1H), 7.92 (s, 1H), 7.88 (t, *J* = 6.2 Hz, 1H), 7.06–6.99 (m, 1H), 6.96–6.93 (m, 2H), 6.87–6.78 (m, 1H), 4.27 (d, *J* = 7.2 Hz, 2H), 3.53 (d, *J* = 6.1 Hz, 2H), 2.48 (t, *J* = 6.1 Hz, 2H), 2.18 (s, 6H), 1.46–1.36 (m, 1H), 0.74–0.62 (m, 2H), 0.48–0.38 (m, 2H); ^13^C NMR (101 MHz, CDCl_3_, ppm) δ 164.74, 159.99 (d, *J* = 10.1 Hz), 157.54 (d, *J* = 10.1 Hz), 154.96 (d, *J* = 12.1 Hz), 152.46 (d, *J* = 12.2 Hz), 149.21, 140.11 (dd, *J* = 11.5, 3.8 Hz), 136.15, 132.09, 125.26, 124.23, 121.66 (dd, *J* = 9.6, 2.0 Hz), 113.43, 111.49 (dd, *J* = 22.9, 3.9 Hz), 107.92, 105.56 (dd, *J* = 26.9, 21.8 Hz), 57.31, 53.95, 44.88, 37.67; HRMS (ESI^+^): *m*/*z* calcd for C_22_H_24_F_2_N_4_O_2_ [M + H]^+^: 414.1867; found: 415.1930; UPLC purity: 99%.

#### 4.1.49 2-(Cyclohexylmethyl)-5-(2,4-difluorophenoxy)-*N*-(2-(dimethylamino)­ethyl)-2*H*-indazole-6-carboxamide (99)

Compound **99** was prepared from 2-(cyclohexylmethyl)-5-(2,4-difluorophenoxy)-2*H*-indazole-6-carboxylic acid (0.153 g, 0.39 mmol, 1.0 equiv), Et_3_N (110 μL, 0.79 mmol, 2.0 equiv), TBTU (0.127 g, 0.39 mmol, 1.0 equiv), and *N*,*N*-dimethylethylenediamine (86 μL, 0.79 mmol, 2.0 equiv) by the procedure used for the synthesis of **87**. The crude product was obtained using reverse-phase preparative chromatography (Biotage Sfär, C18 D 30 g column) using 0.1% TFA­(aq)/MeCN as a mobile phase to obtain a white solid (0.058 g, 32% yield). *R*_f_ = 0.31 (CH_2_Cl_2_/MeOH 9:1); ^1^H NMR (400 MHz, CDCl_3_, ppm) δ 8.52 (s, 1H), 7.84 (t, *J* = 4.9 Hz, 1H), 7.74 (d, *J* = 0.9 Hz, 1H), 7.02 (dt, *J* = 9.1, 5.2 Hz, 1H), 6.98 (ddd, *J* = 5.4, 2.6, 1.4 Hz, 1H), 6.94 (s, 1H), 6.83 (m, 1H), 4.21 (d, *J* = 7.2 Hz, 2H), 3.51 (q, *J* = 6.0 Hz, 2H), 2.45 (t, *J* = 6.1 Hz, 2H), 2.15 (s, 6H), 2.08–1.97 (m, 1H), 1.73–1.64 (m, 3H), 1.61–1.55 (m, 2H), 1.28–1.14 (m, 3H), 0.99 (qd, *J* = 11.9, 3.4 Hz, 2H); ^13^C NMR (101 MHz, CDCl_3_, ppm) δ 165.25, 159.88 (d, *J* = 10.2 Hz), 157.44 (d, *J* = 10.2 Hz), 154.96 (d, *J* = 12.2 Hz), 152.46 (d, *J* = 12.2 Hz), 149.67, 145.68, 140.25 (dd, *J* = 11.6, 3.9 Hz), 125.95, 122.92, 122.70, 121.80 (dd, *J* = 9.6, 2.0 Hz), 111.38 (dd, *J* = 22.8, 3.9 Hz), 105.83, 105.42 (dd, *J* = 26.8, 21.9 Hz), 60.43, 57.42, 44.93, 39.01, 37.54, 30.63, 26.17, 25.56; HRMS (ESI^+^): *m*/*z* calcd for C_25_H_30_F_2_N_4_O_2_: 457.2415; found: 457.2400; UPLC purity: 98%.

#### 4.1.50 2-(Cyclohexylmethyl)-5-(2,4-difluorophenoxy)-*N*-(3-(dimethylamino)­propyl)-2*H*-indazole-6-carboxamide (100)

Compound **100** was prepared from 2-(cyclohexylmethyl)-5-(2,4-difluorophenoxy)-2*H*-indazole-6-carboxylic acid (0.100 g, 0.26 mmol, 1.0 equiv), Et_3_N (72 μL, 0.52 mmol, 2.0 equiv), TBTU (0.083 g, 0.26 mmol, 1.0 equiv), and *N*,*N*-dimethylpropylenediamine (64 μL, 0.52 mmol, 2.0 equiv) by the procedure used for the synthesis of **87**. The crude product was obtained using reverse-phase preparative chromatography (Biotage Sfär, C18 D 30 g column) using 0.1% TFA_(aq)_/MeCN as a mobile phase to obtain a bright yellow solid (0.036 g, 30% yield). *R*_f_ = 0.20 (CH_2_Cl_2_/MeOH 9:1); ^1^H NMR (400 MHz, CDCl_3_, ppm) δ 8.53 (t, *J* = 0.7 Hz, 1H), 7.87 (d, *J* = 5.6 Hz, 1H), 7.72 (d, *J* = 1.0 Hz, 1H), 7.07 (td, *J* = 9.0, 5.4 Hz, 1H), 6.98 (ddd, *J* = 10.4, 8.3, 3.0 Hz, 1H), 6.87 (d, *J* = 0.6 Hz, 1H), 6.89–6.81 (m, 1H), 4.20 (d, *J* = 7.3 Hz, 2H), 3.53 (q, *J* = 6.3 Hz, 2H), 2.32 (t, *J* = 6.8 Hz, 2H), 2.11 (s, 6H), 2.09–1.94 (m, 1H), 1.75 (p, *J* = 6.6 Hz, 2H), 1.73–1.60 (m, 3H), 1.61–1.52 (m, 2H), 1.29–1.12 (m, 3H), 1.05–0.90 (m, 2H); ^13^C NMR (101 MHz, CDCl_3_, ppm) δ 165.20, 160.19 (d, *J* = 10.3 Hz), 157.74 (d, *J* = 10.2 Hz), 155.21 (d, *J* = 12.2 Hz), 152.71 (d, *J* = 12.3 Hz), 150.05, 145.60, 139.78 (dd, *J* = 11.7, 3.8 Hz), 125.71, 122.85 (d, *J* = 3.0 Hz), 122.62 (dd, *J* = 9.6, 1.9 Hz), 122.06, 111.61 (dd, *J* = 22.9, 3.9 Hz), 105.60 (dd, *J* = 26.8, 21.8 Hz), 105.01, 60.41, 58.05, 45.45, 39.08 (d, *J* = 14.1 Hz), 30.62, 26.83, 26.16, 25.55; HRMS (ESI^+^): *m*/*z* calcd for C_26_H_32_F_2_N_4_O_2_ [M + H]^+^: 471.2571; found: 471.2560; UPLC purity: 95%.

#### 4.1.51 1-(Cyclohexylmethyl)-5-(2,4-difluorophenoxy)-*N*-(2-(3-methoxypiperidin-1-yl)­ethyl)-1*H*-indazole-6-carboxamide (101)

To a 25 mL round-bottom flask equipped with a stirring bar, 2-(3-methoxypiperidin-1-yl)­ethan-1-aminium chloride (**33f**) (0.082 g, 0.52 mmol, 2.0 equiv) was added. CH_2_Cl_2_ (15 mL) was added under argon, and a suspension was formed, which was cooled to 0 °C on an ice bath. Subsequently, Et_3_N (72 μL, 0.52 mmol, 2.0 equiv) was added dropwise, resulting in dissolution of **33f**. After 10 min, TBTU (0.083 g, 0.26 mmol, 1.0 equiv) was added in two portions and the reaction mixture was left to stir on an ice bath under argon for 1 h before 1-(cyclohexylmethyl)-5-(2,4-difluorophenoxy)-1*H*-indazole-6-carboxylic acid (**51**) (0.100 g, 0.31 mmol, 1.2 equiv) was added. Finally, the reaction mixture was left to warm to room temperature and stir for 2 days. After 46 h, the reaction mixture was washed with saturated NaHCO_3_ solution (2 × 10 mL) and brine (2 × 10 mL), dried with Na_2_SO_4_, and CH_2_Cl_2_ evaporated under reduced pressure. The crude obtained was then purified by reverse-phase preparative chromatography (Biotage Sfär, C18 D 30 g column) using 0,1% TFA­(aq)/MeCN as a mobile phase to obtain an off-white solid (0.060 g, 44% yield). *R*_f_ = 0.33 (CH_2_Cl_2_/MeOH 10:1); ^1^H NMR (400 MHz, CDCl_3_, ppm) δ 8.59 (s, 1H), 7.97 (t, *J* = 4.9 Hz, 1H), 7.71 (d, *J* = 1.0 Hz, 1H), 7.13 (td, *J* = 8.9, 5.4 Hz, 1H), 7.00 (ddd, *J* = 10.3, 8.2, 3.0 Hz, 1H), 6.90 (m, 1H), 6.84 (s, 1H), 4.20 (d, *J* = 7.2 Hz, 2H), 3.63–3.51 (m, 2H), 3.24 (s, 3H), 2.98–2.88 (m, 2H), 2.70–2.61 (m, 1H), 2.55 (td, *J* = 6.0, 1.4 Hz, 2H), 2.06–2.01 (m, 1H), 1.98–1.90 (m, 1H), 1.88–1.82 (m, 2H), 1.73–1.66 (m, 2H), 1.64–1.62 (m, 1H), 1.60–1.54 (m, 3H), 1.26–1.22 (m, 2H), 1.21–1.16 (m, 2H), 1.11 (ddd, *J* = 12.6, 9.5, 3.7 Hz, 1H), 0.98 (qd, *J* = 12.1, 3.3 Hz, 2H); ^13^C NMR (101 MHz, CDCl_3_, ppm) δ 163.09, 158.44 (d, *J* = 10.2 Hz), 155.99 (d, *J* = 10.1 Hz), 153.55 (d, *J* = 12.3 Hz), 151.04 (d, *J* = 12.3 Hz), 148.69, 143.65, 137.64 (dd, *J* = 11.8, 3.9 Hz), 123.24, 121.29 (dd, *J* = 9.7, 2.0 Hz), 121.15, 120.90, 120.20, 109.77 (dd, *J* = 22.9, 3.9 Hz), 103.70 (dd, *J* = 26.8, 21.7 Hz), 102.51, 58.50, 55.74, 54.55, 54.06, 51.17, 37.10, 34.97, 28.71, 28.10, 24.25, 23.64, 21.24; HRMS (ESI^+^): *m*/*z* calcd for C_29_H_36_F_2_N_4_O_3_ [M + H]^+^: 527.2775; found: 527.2812; UPLC purity: 99%.

#### 4.1.52 2-(Cyclohexylmethyl)-5-(2,4-difluorophenoxy)-*N*-(2-(3-(methoxymethyl)­piperidin-1-yl)­ethyl)-2*H*-indazole-6-carboxamide (102)

To a 25 mL round-bottom flask equipped with a stirring bar, 2-(3-methoxypiperidin-1-yl)­ethan-1-aminium chloride (**33g**) (0.089 g, 0.52 mmol, 2.0 equiv) was added. CH_2_Cl_2_ (15 mL) was added under argon, and a suspension was formed, which was cooled to 0 °C on an ice bath. Subsequently, Et_3_N (72 μL, 0.52 mmol, 2.0 equiv) was added dropwise, resulting in dissolution of **33f**. After 10 min, TBTU (0.083 g, 0.26 mmol, 1.0 equiv) was added in two portions and the reaction mixture was left to stir on an ice bath under argon for 1 h before 1-(cyclohexylmethyl)-5-(2,4-difluorophenoxy)-1*H*-indazole-6-carboxylic acid (**51**) (0.100 g, 0.31 mmol, 1.2 equiv) was added. Finally, the reaction mixture was left to warm to room temperature and stir for 2 days. After 46 h, the reaction mixture was washed with saturated NaHCO_3_ solution (2 × 10 mL) and brine (2 × 10 mL), dried with Na_2_SO_4_, and CH_2_Cl_2_ evaporated under reduced pressure. The crude obtained was then purified by reverse-phase preparative chromatography (Biotage Sfär, C18 D 30 g column) using 0,1% TFA­(aq)/MeCN as a mobile phase to obtain an off-white solid (0.026 g, 19% yield). *R*_f_ = 0.40 (CH_2_Cl_2_/MeOH 10:1); ^1^H NMR (400 MHz, DMSO-*d*_6_, ppm) δ 8.28 (t, *J* = 5.2 Hz, 1H), 8.24 (d, *J* = 1.0 Hz, 1H), 7.96 (s, 1H), 7.47 (ddd, *J* = 11.8, 8.9, 3.0 Hz, 1H), 7.22 (td, *J* = 9.2, 5.6 Hz, 1H), 7.12–7.07 (m, 1H), 7.06 (s, 1H), 4.26 (d, *J* = 7.2 Hz, 2H), 3.18 (s, 3H), 3.13–3.06 (m, 2H), 2.79–2.61 (m, 2H), 2.36 (t, *J* = 6.6 Hz, 2H), 1.98–1.84 (m, 2H), 1.71–1.58 (m, 5H), 1.53–1.41 (m, 5H), 1.31–1.18 (m, 2H), 1.17–1.08 (m, 3H), 1.02–0.83 (m, 3H); ^13^C NMR (101 MHz, DMSO-*d*_6_, ppm) δ 164.90, 159.24 (d, *J* = 11.8 Hz), 156.77 (d, *J* = 10.8 Hz), 154.34, 151.99 (d, *J* = 13.4 Hz), 148.87, 144.45, 140.25 (d, *J* = 11.3 Hz), 127.24, 124.45, 122.58 (d, *J* = 9.7 Hz), 121.29, 119.53, 111.84 (d, *J* = 22.7 Hz), 106.07, 105.43 (dd, *J* = 22.4, 5.9 Hz), 75.30, 58.83, 58.06, 56.85 (d, *J* = 3.9 Hz), 53.66, 38.41, 36.66, 35.94, 29.82, 27.03, 25.79, 25.05, 24.37; HRMS (ESI^+^): *m*/*z* calcd for C_30_H_38_F_2_N_4_O_3_ [M + H]^+^: 541.2912; found: 541.2972; UPLC purity: 99%.

#### 4.1.53 1-(Cyclohexylmethyl)-5-(2,4-difluorophenoxy)-*N*-(2-morpholinoethyl)-2*H*-indazole-6-carboxamide (103)

Compound **103** was prepared from 5-(2,4-difluorophenoxy)-2-cyclohexylmethyl-2*H*-indazole-6-carboxylic acid (0.100 g, 0.26 mmol, 1.0 equiv), Et_3_N (72 μL, 0.52 mmol, 2.0 equiv), TBTU (0.083 g, 0.26 mmol, 1.0 equiv), and 2-morpholinoethan-1-amine (68 μL, 0.52 mmol, 2.0 equiv) by the procedure used for the synthesis of **87**. The crude product was obtained using reverse-phase preparative chromatography (Biotage Sfär, C18 D 30 g column) using 0.1% TFA­(aq)/MeCN as a mobile phase to obtain a bright yellow solid (36% yield). ^1^H NMR (400 MHz, CDCl_3_, ppm) δ 8.61 (s, 1H), 8.04 (t, *J* = 4.7 Hz, 1H), 7.72 (d, *J* = 0.9 Hz, 1H), 7.13 (td, *J* = 9.0, 5.4 Hz, 1H), 7.01 (ddd, *J* = 10.3, 8.2, 2.9 Hz, 1H), 6.90 (tdd, *J* = 7.7, 3.0, 1.5 Hz, 1H), 6.86 (s, 1H), 4.21 (d, *J* = 7.2 Hz, 2H), 3.59 (d, *J* = 5.7 Hz, 2H), 3.48 (t, *J* = 4.6 Hz, 4H), 2.54 (t, *J* = 5.9 Hz, 2H), 2.41 (t, *J* = 4.6 Hz, 4H), 2.03 (m 1H), 1.73–1.64 (m, 3H), 1.61–1.53 (m, 2H), 1.29–1.11 (m, 3H), 1.03–0.93 (m, 2H); ^13^C NMR (101 MHz, CDCl_3_, ppm) δ 165.15, 160.49 (d, *J* = 10.2 Hz), 158.03 (d, *J* = 10.1 Hz), 155.52 (d, *J* = 12.3 Hz), 153.01 (d, *J* = 12.2 Hz), 150.69, 145.71, 139.67 (dd, *J* = 11.7, 3.9 Hz), 125.18, 123.29, 123.25–123.12 (m), 122.96, 122.27, 111.89 (dd, *J* = 22.9, 3.9 Hz), 105.83 (dd, *J* = 26.8, 21.7 Hz), 104.64, 66.94, 60.58, 56.90, 53.36, 39.15, 36.47, 30.76, 26.30, 25.69; HRMS (ESI^+^): *m*/*z* calcd for C_27_H_32_F_2_N_4_O_3_ [M + H]^+^: 498.2442; found: 499.2510; UPLC purity: 99%.

#### 4.1.54 *N*-(2-(1*H*-Pyrrol-1-yl)­ethyl)-1-(cyclohexylmethyl)-5-(2,4-difluorophenoxy)-2*H*-indazole-6-carboxamide (104)

Compound **104** was prepared from 5-(2,4-difluorophenoxy)-2-cyclohexylmethyl-2*H*-indazole-6-carboxylic acid (0.100 g, 0.26 mmol, 1.0 equiv), Et_3_N (72 μL, 0.52 mmol, 2.0 equiv), TBTU (0.083 g, 0.26 mmol, 1.0 equiv), and 2-(1*H*-pyrrol-1-yl)­ethan-1-amine (0.057 mg, 0.52 mmol, 2.0 equiv) by the procedure used for the synthesis of **87**. The crude product was obtained using reverse-phase preparative chromatography (Biotage Sfär, C18 D 30 g column) using 0.1% TFA­(aq)/MeCN as a mobile phase to obtain a bright yellow solid (33% yield). ^1^H NMR (400 MHz, CDCl_3_, ppm) δ 8.61 (s, 1H), 7.71 (s, 1H), 7.53 (t, *J* = 5.8 Hz, 1H), 7.06 (td, *J* = 9.0, 5.5 Hz, 1H), 6.99 (ddd, *J* = 10.3, 8.3, 2.9 Hz, 1H), 6.90 (tdd, *J* = 7.7, 2.9, 1.5 Hz, 1H), 6.80 (s, 1H), 6.64 (t, *J* = 2.1 Hz, 2H), 5.99 (t, *J* = 2.1 Hz, 2H), 4.21 (d, *J* = 7.3 Hz, 2H), 4.16–4.09 (m, 2H), 3.81 (q, *J* = 5.8 Hz, 2H), 2.07–2.02 (m, 1H), 1.76–1.64 (m, 3H), 1.63–1.54 (m, 1H), 1.28–1.22 (m, 2H), 1.21–1.13 (m, 2H), 1.04–0.94 (m, 2H); ^13^C NMR (101 MHz, CDCl_3_, ppm) δ 165.50, 160.66 (d, *J* = 10.3 Hz), 158.20 (d, *J* = 10.2 Hz), 155.58 (d, *J* = 12.3 Hz), 153.07 (d, *J* = 12.3 Hz), 150.62, 145.50, 138.93 (dd, *J* = 11.8, 3.9 Hz), 124.33, 123.55 (dd, *J* = 9.8, 1.9 Hz), 123.43, 122.97, 122.35, 120.62, 111.77 (dd, *J* = 22.9, 3.9 Hz), 108.65, 105.76 (dd, *J* = 26.9, 21.8 Hz), 104.17, 60.54, 48.94, 41.65, 39.11, 30.71, 26.25, 25.65; HRMS (ESI^+^): *m*/*z* calcd for C_27_H_28_F_2_N_4_O_2_ [M + H]^+^: 478.2180; found: 480.2201; UPLC purity: 98%.

#### 4.1.55 *N*-(2-(1*H*-Imidazol-1-yl)­ethyl)-2-(cyclohexylmethyl)-5-(2,4-difluorophenoxy)-2*H*-indazole-6-carboxamide (105)

Compound **105** was prepared from 5-(2,4-difluorophenoxy)-2-cyclohexylmethyl-2*H*-indazole-6-carboxylic acid (0.100 g, 0.26 mmol, 1.0 equiv), Et_3_N (72 μL, 0.52 mmol, 2.0 equiv), TBTU (0.083 g, 0.26 mmol, 1.0 equiv), and 2-(1*H*-imidazol-1-yl)­ethan-1-amine (0.058 mg, 0.52 mmol, 2.0 equiv) by the procedure used for the synthesis of **87** with DMF replacing CH_2_Cl_2_. The crude product was obtained using reverse-phase preparative chromatography (Biotage Sfär, C18 D 30 g column) using 0.1% TFA­(aq)/MeCN as a mobile phase to obtain a white solid (33% yield). *R*_f_ = 0.11 (CH_2_Cl_2_/MeOH 15:1); ^1^H NMR (400 MHz, CDCl_3_, ppm) δ 8.60 (s, 1H), 7.72 (d, *J* = 1.0 Hz, 1H), 7.63 (t, *J* = 5.9 Hz, 1H), 7.46 (s, 1H), 7.08 (td, *J* = 9.0, 5.5 Hz, 1H), 6.99 (ddd, *J* = 10.4, 8.2, 2.9 Hz, 1H), 6.96–6.90 (m, 2H), 6.89 (ddd, *J* = 9.2, 3.0, 1.6 Hz, 1H), 6.81 (s, 1H), 4.24–4.17 (m, 4H), 3.79 (q, *J* = 5.9 Hz, 2H), 2.10–1.95 (m, 1H), 1.73–1.63 (m, 3H), 1.59–1.53 (m, 2H), 1.21–1.11 (m, 3H), 1.04–0.92 (m, 2H); ^13^C NMR (101 MHz, CDCl_3_, ppm) δ 165.79, 160.75 (d, *J* = 10.2 Hz), 158.29 (d, *J* = 10.3 Hz), 155.50 (d, *J* = 12.3 Hz), 153.00 (d, *J* = 12.5 Hz), 150.49, 145.46, 138.74 (dd, *J* = 11.8, 4.0 Hz), 137.33, 129.71, 123.90, 123.59, 123.64–123.34 (m), 123.07, 122.45, 119.00, 111.97 (dd, *J* = 22.9, 3.9 Hz), 105.90 (dd, *J* = 26.9, 21.8 Hz), 104.32, 60.60, 46.27, 41.37, 39.14, 30.72, 26.26, 25.66; HRMS (ESI^+^): *m*/*z* calcd for C_26_H_27_F_2_N_5_O_2_ [M + H]^+^: 480.2133; found: 480.2201; UPLC purity: 98%.

#### 4.1.56 2-Benzyl-5-(2,4-difluorophenoxy)-*N*-(2-(dimethylamino)­ethyl)-2*H*-indazole-6-carboxamide (106)

Compound **106** was prepared from 2-benzyl-5-(2,4-difluorophenoxy-2*H*-indazole-6-carboxylic acid (0.200 g, 0.53 mmol, 1.0 equiv), Et_3_N (146 μL, 1.06 mmol, 2.0 equiv), TBTU (0.169 g, 0.53 mmol, 1.0 equiv), and *N*,*N*-dimethylethylenediamine (115 μL, 1.06 mmol, 2.0 equiv) by the procedure used for the synthesis of **87**. The crude product was obtained using reverse-phase preparative chromatography (Biotage Sfär, C18 D 30 g column) using 0.1% TFA­(aq)/MeCN as a mobile phase to obtain a light yellow solid (50% yield). *R*_f_ = 0.20 (CH_2_Cl_2_/MeOH 10:1); ^1^H NMR (400 MHz, CDCl_3_, ppm) δ 8.56 (s, 1H), 7.86 (s, 1H), 7.74 (d, *J* = 0.9 Hz, 1H), 7.38–7.32 (m, 3H), 7.29–7.27 (m, 2H), 7.02 (dt, *J* = 9.2, 5.6 Hz, 1H), 6.99–6.93 (m, 1H), 6.91 (s, 1H), 6.83 (m, 1H), 5.58 (s, 2H), 3.53 (q, *J* = 5.8 Hz, 2H), 2.47 (t, *J* = 6.1 Hz, 2H), 2.17 (s, 6H); ^13^C NMR (101 MHz, CDCl_3_, ppm) δ 165.31, 160.09 (d, *J* = 10.2 Hz), 157.64 (d, *J* = 10.2 Hz), 155.12 (d, *J* = 12.1 Hz), 152.62 (d, *J* = 12.3 Hz), 150.06, 145.88, 140.17 (dd, *J* = 11.6, 4.0 Hz), 135.42, 129.13, 128.71, 128.20, 126.32, 123.03, 122.76, 122.63, 122.07 (dd, *J* = 9.7, 2.0 Hz), 111.54 (dd, *J* = 22.9, 3.9 Hz), 105.81, 105.49 (dd, *J* = 21.8, 5.0 Hz), 58.02, 57.52, 45.03, 37.64; HRMS (ESI^+^): *m*/*z* calcd for C_25_H_24_F_2_N_4_O_2_ [M + H]^+^: 451.1867; found: 451.1934; UPLC purity: 98%.

#### 4.1.57 *N*-(2-(Dimethylamino)­ethyl)-5-(4-fluorophenoxy)-2-isobutyl-2*H*-indazole-6-carboxamide (107)

Compound **107** was prepared from 5-(4-fluorophenoxy)-2-isobutyl-2*H*-indazole-6-carboxylic acid (0.167 g, 0.51 mmol, 1.0 equiv), Et_3_N (142 μL, 1.02 mmol, 2.0 equiv), TBTU (0.163 g, 0.51 mmol, 1.0 equiv), and *N*,*N*-dimethylethylenediamine (111 μL, 1.02 mmol, 2.0 equiv) by the procedure used for the synthesis of **87**. The crude product was obtained using reverse-phase preparative chromatography (Biotage Sfär, C18 D 30 g column) using 0.1% TFA­(aq)/MeCN as a mobile phase to obtain a bright yellow solid (0.037 g, 4% yield). *R*_f_ = 0.11 (CH_2_Cl_2_/MeOH 9:1); ^1^H NMR (400 MHz, CDCl_3_, ppm) δ 8.58 (s, 1H), 7.97 (s, 1H), 7.77 (d, *J* = 1.0 Hz, 1H), 7.05 (s, 1H), 7.04–6.96 (m, 4H), 4.20 (d, *J* = 7.4 Hz, 2H), 3.49 (q, *J* = 6.1 Hz, 2H), 2.42 (t, *J* = 6.1 Hz, 2H), 2.36 (h, *J* = 7.0 Hz, 1H), 2.16 (s, 6H), 0.94 (d, *J* = 6.7 Hz, 6H); ^13^C NMR (101 MHz, CDCl_3_, ppm) δ 165.25, 160.02, 157.61, 153.10 (d, *J* = 2.6 Hz), 149.66, 145.79, 126.32, 122.79 (d, *J* = 3.7 Hz), 122.42, 119.75 (d, *J* = 8.3 Hz), 116.42, 116.19, 107.93, 61.51, 57.41, 44.97, 37.43, 29.97, 19.95; HRMS (ESI^+^): *m*/*z* calcd for C_22_H_27_FN_4_O_2_ [M + H]^+^: 399.2118; found: 399.2187; UPLC purity: 99%.

#### 4.1.58 2-(Cyclohexylmethyl)-5-(2,4-difluorobenzyl)-*N*-(2-(dimethylamino)­ethyl)-2*H*-indazole-6-carboxamide (108)

Compound **108** was prepared from 2-(cyclohexylmethyl)-5-(2,4-difluorobenzyl)-2*H*-indazole-6-carboxylic acid (0.118 g, 0.26 mmol, 1.0 equiv), Et_3_N (86 μL, 0.52 mmol, 2.0 equiv), TBTU (0.099 g, 0.26 mmol, 1.0 equiv), and *N*,*N*-dimethylethylenediamine (67 μL, 0.52 mmol, 2.0 equiv) by the procedure used for the synthesis of **87**. The crude product was obtained using reverse-phase preparative chromatography (Biotage Sfär, C18 D 30 g column) using 0.1% TFA­(aq)/MeCN as a mobile phase to obtain an off-white viscous oil (0.052 g, 44% yield). *R*_f_ = 0.12 (CH_2_Cl_2_/MeOH 10:1); ^1^H NMR (400 MHz, CDCl_3_, ppm) δ 7.73 (s, 1H), 7.71 (s, 1H), 7.32 (s, 1H), 7.07 (td, *J* = 8.8, 6.5 Hz, 1H), 6.73–6.64 (m, 2H), 6.52 (t, *J* = 4.9 Hz, 1H), 4.18 (s, 2H), 4.12 (d, *J* = 7.2 Hz, 2H), 3.34 (q, *J* = 5.6 Hz, 2H), 2.31 (t, *J* = 6.0 Hz, 2H), 2.10 (s, 6H), 2.01–1.91 (m, 1H), 1.67–1.56 (m, 3H), 1.54–1.46 (m, 2H), 1.21–1.04 (m, 3H), 0.92 (qd, *J* = 12.0, 3.4 Hz, 2H); ^13^C NMR (101 MHz, CDCl_3_, ppm) δ 168.41, 160.91 (d, *J* = 11.7 Hz), 160.44 (d, *J* = 11.7 Hz), 158.47 (d, *J* = 11.8 Hz), 157.97 (d, *J* = 11.7 Hz), 145.19, 133.76, 130.20 (dd, *J* = 9.4, 6.2 Hz), 128.66, 122.32 (dd, *J* = 15.6, 3.7 Hz), 121.47, 120.25, 119.43, 114.96, 108.94 (dd, *J* = 20.7, 3.7 Hz), 101.67 (t, *J* = 25.6 Hz), 58.39, 55.65, 43.18, 37.21, 35.27, 29.93 (d, *J* = 2.6 Hz), 28.82, 24.38, 23.78; HRMS (ESI^+^): *m*/*z* calcd for C_26_H_32_F_2_N_4_O [M + H]^+^: 455.2544; found: 455.2606; UPLC purity: 98%.

#### 4.1.59 5-(2,4-Difluorobenzyl)-*N*-(2-(dimethylamino)­ethyl)-1-methyl-2*H*-indazole-6-carboxamide (109)

Compound **109** was prepared from 2-(cyclohexylmethyl)-5-(2,4-difluorobenzyl)-2*H*-indazole-6-carboxylic acid (0.118 g, 0.26 mmol, 1.0 equiv), Et_3_N (86 μL, 0.52 mmol, 2.0 equiv), TBTU (0.099 g, 0.26 mmol, 1.0 equiv), and *N*,*N*-dimethylethylenediamine (67 μL, 0.52 mmol, 2.0 equiv) by the procedure used for the synthesis of **87**. The crude product was obtained using reverse-phase preparative chromatography (Biotage Sfär, C18 D 30 g column) using 0.1% TFA_(aq)_/MeCN as a mobile phase to obtain an off-white viscous oil (0.044 g, 37% yield). ^1^H NMR (400 MHz, CDCl_3_, ppm) δ 7.92 (s, 1H), 7.51 (s, 1H), 7.45 (s, 1H), 7.10 (td, *J* = 9.2, 8.8, 6.4 Hz, 1H), 6.81–6.72 (m, 2H), 6.37 (s, 1H), 4.24 (s, 2H), 4.07 (s, 3H), 3.48–3.41 (m, 2H), 2.42 (t, *J* = 5.9 Hz, 2H), 2.18 (s, 6H); ^13^C NMR (101 MHz, CDCl_3_, ppm) δ 170.17, 162.75 (d, *J* = 11.8 Hz), 162.27 (d, *J* = 11.8 Hz), 160.31 (d, *J* = 11.8 Hz), 159.80 (d, *J* = 11.7 Hz), 147.22, 135.71, 131.98 (dd, *J* = 9.3, 6.2 Hz), 130.65, 124.03 (dd, *J* = 15.7, 3.8 Hz), 123.48, 122.57, 121.08 (d, *J* = 1.2 Hz), 110.75 (dd, *J* = 20.7, 3.7 Hz), 104.79–101.12 (m), 57.44, 45.00, 40.50, 37.04, 31.79 (d, *J* = 2.5 Hz); UPLC purity: 96%.

### 4.2 Computational Studies

Computational studies were performed on workstations at the Department of Pharmaceutical Chemistry, Faculty of Pharmacy, University of Ljubljana, using Schrödinger Small Molecule Discovery Suite Release 2021-1 (Schrödinger, LLC, New York, USA, 2021). hBChE (PDB code 6QAA) and p38α MAPK (PDB structure 1A9U) crystal structures were preprocessed using the Protein Preparation Wizard (bond orders were assigned using CCD database, missing hydrogens were added, termini were capped, the missing side chains and loops were modeled with Prime, whereas the het protonation states (pH 7.0 ± 2.0) were modeled with Epik.^80^ All hets (except cocrystallized ligands), cosolvent molecules, and waters were removed. Hydrogen bonds were automatically assigned and optimized using PROPKA (pH 7.0). Receptor grids were generated (van der Waals radii scaling: 1, partial charge cutoff: 0.25). The active site was defined as the centroid of the cocrystallized ligand (inner box: 10 Å^3^). For p38α MAPK, an additional constraint had to be satisfied–a hydrogen bond with Met109 amide nitrogen.

A library of known p38α MAPK inhibitors was generated from ChEMBL (p38αMAPK-CHEMBL260, activity threshold *K*_d_/IC_50_ < 50 μM) and PBD (MAPK14: Accession Code(s)—UniProt: Q16539) on 25th April, 2021. Compounds were first examined for known classes of assay interference compounds using the Molecular Catalog Filter node in KNIME (KNIME Analytics Platform Release 4.3.3., AG, Zurich, Switzerland). This yielded 5490 compounds, of which 172 were commercially available in the Molport database. These 172 ligands were prepared using the LigPrep tool and docked into the hBChE active site using Glide XP^81^ and OPLS_2005 force field. After docking, ligands with MW > 500 Da were removed, and the rest were clustered into 15 clusters by k-means clustering in KNIME Analytics Platform Release 4.3.3. From each cluster, 0, 1, or 2 ligands were selected based on the two criteria: a cation−π interaction with Trp82 of the hBChE choline-binding pocket and the presence of an aromatic moiety in the acyl-binding pocket. The final 8 compounds were purchased and evaluated on both enzymes in vitro.

Molecular dynamics (MD) simulations were used to evaluate the dynamic stability of the docked poses. The docked poses were merged with their respective receptors (i.e., hBChE and p38α MAPK, respectively) and prepared by System Builder tool: protein was solvated by TIP4P^82^ water molecules in an orthorhombic box (10 Å buffer zone from the protein surface), sodium and chloride ions were added both neutralize the system and produce the final 0.15 M concentration, and OPLS_2005 force field^83,84^ was used for parametrization. The default Desmond *NPT* relaxation protocol was used for the equilibration, which was followed by the production stage with the following settings: 1.2 ps interval for energy, RESPA integrator with 2 fs time step, cutoff scheme at 9.0 Å, random seed, isothermal–isobaric *NPT* ensemble at 300 K and 1.013 bar pressure with Nose–Hoover chain thermostat and Martyna-Tobias-Klein barostat (1 and 2 ps relaxation time, respectively, isotropic coupling). The simulation time was 100 ns with 1000 frames saved per trajectory, and the results were analyzed using the built-in Desmond tools.

### 4.3 In Vitro Enzymatic Activity Studies

#### 4.3.1 hAChE, hBChE, and Human p38α MAPK Inhibition Assays

The inhibitory potencies of the compounds against the ChEs were determined using the method of Ellman.^41^ Briefly, compound stock solutions in DMSO were incubated with Ellman’s reagent and the ChEs (final concentrations: 370 μM Ellman’s reagent, approximately 1 nM or 50 pM recombinant hBChE or recombinant hAChE, respectively) in 0.1 M sodium phosphate pH 8.0 for 5 min at 20 °C. The reactions were started by the addition of the substrate (final concentration, 500 μM butyrylthiocholine iodide or acetylthiocholine iodide for hBChE and hAChE, respectively). The final content of DMSO was always 1% [v/v]. The increase in absorbance at 412 nm was monitored for 2 min using a 96-well microplate reader (Synergy HT, BioTek Instruments, VT, USA). The initial velocities in the presence (*v*_*i*_) and absence (*v*_0_) of the test compounds were calculated. The inhibitory potencies were expressed as the residual activities, according to RA = (*v*_i_ – *b*)/(*v*_0_ – *b*), where *b* is the blank value using phosphate buffer without ChEs. For IC_50_ determinations, at least seven different concentrations for each compound were used. The IC_50_ values were obtained by plotting the residual ChE activities against the applied inhibitor concentrations, with the experimental data fitted to a four-parameter logistic function (GraphPad Prism 10.0). Tacrine and donepezil were used as positive controls.

The inhibitory activities against human p38α MAPK were evaluated using the commercially available ADP-Glo Kinase Assay (Promega, Inc., USA). The assay kit included ADP-Glo reagent, Kinase Detection Buffer, Kinase Detection Substrate, Ultra Pure ATP (10 mM), and ADP (10 mM). The reaction was monitored using a white 384-well microtiter plate (REF: 784075, Greiner Bio-One, Austria) and a plate reader (Synergy HT, BioTek Instruments Inc., USA).

For the reference sample, 1% [v/v] DMSO replaced the inhibitor solution. The enzyme solution was prepared by diluting the enzyme stock (human recombinant p38α MAPK, expressed in Sf9, an insect cell line, stock concentration: 0.1 μg/μL) in 1× Kinase Buffer (pH = 7.5). The 1× Kinase Buffer was prepared from Milli-Q water and contained 40 mM Tris × HCl, 20 mM MgCl_2_, 0.1% BSA, 5% DMSO and 50 μM DTT (pH = 7.5) The substrate/ATP mix was prepared by diluting substrate peptide IPTTPITTTYFFFKKK stock solution (c_0_ = 1 mg/mL) and ATP stock solution (c_0_ = 1 mM) in 1× Kinase Buffer to reach the final concentration in the well of 0.2 μg/μL and 100 μM, respectively. The enzyme reaction was started with the addition of the enzyme solution. The DMSO concentration in the test solution was maintained at 1% [v/v]. The plate was incubated for 60 min. Then 5 μL of ADP-Glo reagent was added, followed by a 40 min incubation at room temperature. Next, 10 μL of Kinase Detection Substrate was added, followed by an additional 30 min incubation, and the luminescence was measured at 570 nm using the microtiter plate reader (Synergy HT, BioTek Instruments Inc., USA, measurement parameters: measurement from top, gain: 150, integration time: 1 s). All incubations were performed at room temperature. The RA and IC_50_ values were calculated as described above (ChEs inhibitory potency).

#### 4.3.2 hBChE Inhibition Assay in SH-SY5Y Cells

hBChE activity in live whole SH-SY5Y cells was measured by the modified procedure of Onder and colleagues.^45^ Briefly, after centrifugation, the complete medium was removed and cells were suspended in PBS warmed to 37 °C. 50 μL of this suspension was added to a 96-well plate so that each well contained approximately 360,000 cells. 81 μL of 0.5 mM 5,5′-dithiobis (2-nitrobenzoic acid) in 0.1 M potassium phosphate buffer (pH 7.0) was added, followed by 2 μL of DMSO solutions of compounds **94**, **95**, or ethopropazine (positive control). The reaction was initiated by the addition of 67 μL of 1 mM acetylthiocholine. The total reagent volume in each well was 200 μL. The microplate was covered with a lid to minimize evaporation while absorbance at 412 nm was recorded over a period of 120 min with a 30 s interval on a Tecan Spark microplate reader (Tecan Austria GmbH, Salzburg, Austria) set to 24 °C. Autohydrolysis rates of butyrylthiocholine were measured in wells that contained all reagents + PBS with no cells. The residual activity of hBChE was then calculated by the protocol described above.

#### 4.3.3 Kinase Selectivity Profiling

The kinase inhibition profile of compounds **94** and **95** was assessed against 103 protein kinases by Eurofins DiscoverX, LLC., using the KINOMEscan assay. Kinase-tagged T7 phage strains were grown in parallel in 24-well blocks in an *E. coli* host derived from the BL21 strain. *E. coli* were grown to log-phase and infected with T7 phage from a frozen stock (multiplicity of infection = 0.4) and incubated with shaking at 32 °C until lysis (90–150 min). The lysates were centrifuged (6000*g*) and filtered (0.2 μm) to remove cell debris. The remaining kinases were produced in HEK-293 cells and subsequently tagged with DNA for qPCR detection. Streptavidin-coated magnetic beads were treated with biotinylated small molecule ligands for 30 min at room temperature to generate affinity resins for kinase assays. The liganded beads were blocked with excess biotin and washed with blocking buffer (SeaBlock (Pierce), 1% BSA, 0.05% Tween 20, 1 mM DTT) to remove unbound ligand and to reduce nonspecific phage binding. Binding reactions were assembled by combining kinases, liganded affinity beads, and test compounds in 1x binding buffer (20% SeaBlock, 0.17× PBS, 0.05% Tween 20, 6 mM DTT). Test compounds were prepared as 100× stocks in 100% DMSO and directly diluted into the assay. All reactions were performed in polypropylene 384-well plates in a final volume of 0.02 mL. The assay plates were incubated at room temperature with shaking for 1 h, and the affinity beads were washed with wash buffer (1× PBS, 0.05% Tween 20). The beads were then resuspended in elution buffer (1× PBS, 0.05% Tween 20, 0.5 μM nonbiotinylated affinity ligand) and incubated at room temperature with shaking for 30 min. The kinase concentration in the eluates was measured by qPCR.

##### Residual Activity (RA) Calculation

The compound(s) were screened at the concentration(s) requested, and results for primary screen binding interactions are reported as RA (%), where lower numbers indicate stronger hits in the matrix.

$$\mathrm{RA}=\left(\frac{\mathrm{test}\;\mathrm{compound}\;\mathrm{signal}-\mathrm{PC}\;\mathrm{signal}}{\mathrm{NC}\;\mathrm{signal}-\mathrm{PC}\;\mathrm{signal}}\right)\times 100$$

Test compound = Cpd. **94** or **95**; NC signal = negative control (DMSO; 100% RA); PC signal = positive control (control compound; 0% RA).

#### 4.3.4 X-ray Crystallography

##### 4.3.4.1 hBChE Crystallographic Study

For crystallization, hBChE was recombinantly produced in CHO cells as a C-terminus truncated form with lower N-glycosylation sites, as previously described.^85^ The secreted enzyme was purified by an initial BChE-specific affinity chromatographic step (Hupresin, Chemforase, Rouen, France), followed by size exclusion chromatography (Superdex 200, Cytiva, France), as previously described.^86^ Crystals were obtained by vapor diffusion and the hanging drop method at 293 K using MES (100 mM, pH 6.5), 2.15 M (NH_4_)_2_SO_4_ as crystallization buffer. Compounds **94**, **95**, and **102** were dissolved in MeOH (0.1 M stock solution), and complexes with hBChE were obtained by soaking crystals in crystallization buffer containing about 2 mM of respective compounds. Crystals were cryo-protected in a solution of MES (100 mM, pH 6.5), 2.15 M (NH_4_)_2_SO_4_, 20% glycerol, before flash cooling into liquid nitrogen.

X-ray data were collected at BM07-FIP2, ID30A-3, and ID30B beamlines of the European Synchrotron Radiation Facility (Grenoble, France) at 100 K. Images were autoprocessed with the EDNA pipeline,^87^ based on XDS/XSCALE for indexing, reduction, and scaling. The initial models were obtained by molecular replacement using the hBChE X-ray structure (PDB 1P0I) and the Phaser program of the Phenix software suite.^88^ hBChE models were further refined by iterative cycles of Phenix.refine and model building (*Coot*). Extra electron density within the hBChE active site allowed for unambiguously fitting the different ligands, whose geometry restraints were generated with Phenix eLBOW^89^ using the semiempirical quantum mechanical AM1 method. The coordinates and structure factors of hBChE complexed with **95**, **94**, and **102** were deposited to the Protein Data Bank under accession IDs 9I5O, 9I5P, and 9I5Q, respectively. Authors will release the atomic coordinates and experimental data upon article publication.

##### 4.3.4.2 p38α MAPK Expression and Purification

The human p38αMAPK used in these studies was produced in *E. coli* and purified. Briefly, bacterial cultures were grown in Terrific Broth medium (BD Difco) at 30 °C, protein expression was induced at OD_600_ = 1.4 by the addition of 1 mM isopropyl-β-D-1-thiogalactopyranoside (Research Products International), and incubation continued at 22 °C for 16 h. Cells were harvested by centrifugation and resuspended at 4 °C in lysis buffer composed of 10 mM Tris–HCl (pH 8.3), 1 M NaCl, 1 mM MgCl_2_, 10% glycerol, 0.01% IGEPAL CA-630 (Sigma), 1 mM tris­(2-carboxyethyl)­phosphine (TCEP) and the Pierce EDTA-free protease inhibitors tablet (Thermo Scientific). Resuspended pellets were placed at −30 °C until purification. Frozen cell suspensions were thawed under the cold running water, sonicated using a Branson SFX250 sonifier with 1/2″ disruptor horn for 15 min (5 s “on”, 10 s “off”), 42% amplitude, 4 °C, and the resultant lysate clarified by centrifugation at 39,000 × *g* for 45 min, 4 °C. The supernatant was collected and subjected to Ni-NTA-affinity column chromatography on HisTrap FF (5 mL) column equilibrated with loading buffer (20 mM Tris–HCl (pH 8.3), 1 M NaCl, 10 mM imidazole (pH 8.3), 2 mM TCEP), washed with 5 cv of loading buffer and 10 cv of loading buffer containing 20 mM imidazole, and the protein was collected via step-elution with loading buffer containing 500 mM Imidazole. Collected protein was dialyzed vs low salt buffer (20 mM Tris-HCl (pH 8.3), 50 mM NaCl, 2 mM TCEP) using D-Tube Dialyzer Mega, MWCO 6–8 kDa (Millipore) and was subjected to ion-exchange chromatography on HiTrap Q HP (5 mL) column (GE Healthcare) using the 30 cv gradient 0.050–1 M NaCl in buffer containing 20 mM Tris–HCl (pH 8.3) and 2 mM TCEP. Multiple fractions showing absorption at A_280_ were SDS-PAGE verified, and the ones containing the p38αMAPK were combined and dialyzed using D-Tube Dialyzer Mega, MWCO 6–8 kDa (Millipore) vs crystallization buffer composed of 10 mM Tris (pH 8.3), 150 mM NaCl, 1 mM TCEP. The equilibrated protein sample was concentrated to 20 mg/mL via multiple 20 min centrifugations at 5000*g*, 4 °C in Vivaspin 15R, 10,000 MWCO (Sartorius) concentrator.

##### 4.3.4.3 Human p38α MAPK Crystallographic Study

Protein samples for crystallization were prepared immediately prior to use by addition of 4 mM 4-[3-(4-fluorophenyl)-1*H*-pyrazol-4-yl] pyridine (4-FPP) (ZW-4-252A) in 8% (v/v) DMSO (Sigma) and kept in an ice bath for 45 min. Crystals of human p38αMAPK were obtained using the sitting drop vapor diffusion method in CombiClover crystallization plates (Rigaku). Drop solutions of protein (5 μL) were composed of 3 μL protein solution: 2 μL reservoir solution. The reservoir solution was composed of 15–20% (w/v) PEG 10,000, containing 100 mM ammonium acetate and 100 mM Bis–Tris (pH 5.5) at 20 °C. Protein:ligand complexes were generated using the crystal soak approach. Solutions of each ligand, 50 mM in neat HPLC-grade MeOH, were diluted to a final concentration of 2.5 mM by addition to the protein crystals in the reservoir solution with 20% (w/v) PEG 10,000 at 20 °C. Selected crystals were flash-cooled with liquid nitrogen directly from the soaking solution and taken for data collection. Diffraction data were collected on beamline ID30B at the European Synchrotron Radiation Facility^90^ at 100 K from orthorhombic crystals using a Eiger2 9 M detector. All data were processed in the XDS software package^91^ via autoPROC.^92^ Initial data fitting used the DIMPLE pipeline within CCP4 program suite (CCP4, 1994) and a ligand/water free human p38α MAPK model (PDB entry 4EWQ).^93^ Ligands were identified and placed into sigma-A weighted fo-fc maps with Coot^94^ using real space refinement. Ligand restraint dictionaries were generated with the Grade2 server. Iterative rounds of refinement and model building were performed with phenix.refine^95^ and Coot. Polder maps^96^ were created for every ligand to ensure the correct placement of its atoms. The model quality was analyzed by MolProbity^97^ within Phenix. The coordinates and structure factors have been deposited in the Protein Data Bank (PDB) with accession codes 9D7N and 9D75. Authors will release the atomic coordinates and experimental data upon article publication.

### 4.4 Biological Studies

#### 4.4.1 Cell Cultures

The human hepatocellular carcinoma HepG2 and human neuroblastoma SH-SY5Y cell lines were purchased from American Type Culture Collection (Manassas, VA, USA). The murine microglia BV2 cells were a generous gift from Dr. Biljana Božić Nedeljković (University of Belgrade, Belgrade, Serbia). Cells were cultured in Advanced Dulbecco’s modified Eagle’s medium (Gibco, Thermo Fisher Scientific, Waltham, MA, USA) supplemented with 10% fetal bovine serum (FBS, Gibco), 2 mM l-glutamine, 50 U/mL penicillin and 50 μg/mL streptomycin (Sigma, St. Louis, MO, USA) in a humidified atmosphere of 95% air and 5% CO_2_ at 37 °C, and grown to 80% confluence. Prior to cell treatment, complete medium was replaced with serum-free medium. Compounds **94** and **95** were prepared as a stock solution of 30 mM in DMSO and were used at concentrations of 1–100 μM.

#### 4.4.2 Metabolic Activity

BV2 cells (5 × 10^3^/well) and HepG2 and SH-SY5Y cells (1 × 10^4^/well) were seeded in 96-well plates and assessed by MTS ([3-(4,5-dimethylthiazol-2-yl)-5-(3-carboxymethoxyphenyl)-2-(4-sulfophenyl)-2*H* tetrazolium, inner salt) assay for their response to compounds **94** and **95**. Cells were treated with increasing concentrations of compounds (1–100 μM) in serum-free medium, and metabolic activity was assessed after 24 h using the CellTiter 96 Aqueous One Solution Cell Proliferation Assay (Promega, Madison, WI, USA), in accordance with the manufacturer’s instructions. Absorbance was measured with an automatic microplate reader (Tecan Safire^2^, Switzerland) at a wavelength of 492 nm. Results are presented as a percentage of the control (DMSO).

#### 4.4.3 Neuroprotection Assay

The neuroprotective effect of compounds **94** and **95** on the cytotoxic effect of Aβ_1–42_ was determined with the fluorescent intercalator 7-AAD, assessed by flow cytometry. The peptide Aβ_1–42_ (Merck Millipore, Darmstadt, Germany) was dissolved in DMSO at a concentration of 1 mM and was used at a final concentration of 5 μM. Prior to cell treatment, the peptide Aβ_1–42_ was aggregated at a concentration of 100 μM for 24 h at 37 °C to obtain fibrils. SH-Y5Y cells were seeded in 24-well culture plates (2 × 10^4^/well) and the next day treated with preaggregated Aβ_1–42_ in the absence or presence of compounds at concentrations 1, 2.5, and 5 μM. After 48 h treatment, cells were harvested and washed with cold PBS, and labeled with 7AAD (2 μg/mL; Sigma-Aldrich) for 10 min at room temperature. Cells were then analyzed for cytotoxicity by flow cytometry on an Attune NxT flow cytometer (Thermo Fisher Scientific). The percentage of 7AAD positive (7AAD^pos^) cells was evaluated using FlowJo software (FlowJo, LLC, Ashland, OR, USA) and recorded as relative to control cells treated with DMSO alone.

#### 4.4.4 Quantification of Nitrite

The gliaprotective effect of compounds **94** and **95** on the pro-inflammatory effect of LPS was determined by measuring nitrite in the culture supernatants of BV2 cells with Griess reagent kits. BV2 cells were seeded in 12-well culture plates (5 × 10^4^ /well) and the next day stimulated with 1 μg/mL LPS (L6529; *Escherichia coli* 055:B5; Sigma-Aldrich, St. Louis, MO, USA) in serum-free medium in the absence or presence of compounds at concentrations 1, 2.5, and 5 μM. After 24 h stimulation, aliquots of the cell supernatants were collected and obtained by centrifugation at 1200 rpm for 5 min and were then either stored at −80 °C or used immediately to determine the nitrite content, an indicator of nitric oxide (NO) production. The accumulation of NO in culture supernatants was determined using Griess reagent kits (Promega), according to the manufacturer’s instructions. Absorbance was measured at 550 nm using an automatic microplate reader (Tecan Safire^2^). NaNO_2_ was used as the standard to calculate nitrite concentrations (in μM).

#### 4.4.5 Peripheral Blood Mononuclear Cells (PBMCs)

Human PBMCs from healthy donors were isolated from heparinized blood by density gradient centrifugation with Ficoll-Paque. The isolated cells were washed twice with PBS, resuspended in RPMI 1640 medium (Sigma-Aldrich, St. Louis, MO, USA) supplemented with 10% heat-inactivated fetal bovine serum (Gibco), 2 mM l-glutamine (Sigma-Aldrich), 100 U/mL penicillin (Sigma-Aldrich), and 100 μg/mL streptomycin (Sigma-Aldrich), and used in the assays.

#### 4.4.6 Cytokine Release from PBMCs

Peripheral blood mononuclear cells were seeded (500,000 cells/well) in 24-well plates in 500 μL of growth medium and treated with compounds **94**, **95**, and neflamapimod (1, 2.5, and 5 μM) in the presence or absence of PHA (1 μg/mL) or the corresponding vehicle (0.1% DMSO). Cell-free supernatants were collected after 18 h of incubation (37 °C, 5% CO_2_) and stored at −80 °C until tested. Cytokine production was determined with BD Cytometric Bead Array human inflammatory cytokines kit (BD Bioscience) on an Attune NxT flow cytometer (Thermo Fisher Scientific, Waltham, MA, USA). Standard curves were generated using recombinant cytokines contained in the kit. The data were analyzed using the FlowJo (Tree Star, Inc., Ashland, OR, USA) and Prism (GraphPad Prism 6.0, GraphPad Software) software.

### 4.5 In Vitro Physicochemical Property Studies

#### 4.5.1 Log *D* Determination

Calibration curves for phosphate-buffered saline (PBS) (pH = 7.4) and octanol were prepared (0.05–5 μM). The buffer and *n*-octanol phases were shaken, using phosphate buffer (pH = 7.4). The phase ratios of buffer to *n*-octanol were 1:1 (0.8 mL octanol:0.8 mL PBS) and 1:3 (0.4 mL octanol:1.2 mL PBS). Octanol was then pipetted into a HPLC vial, followed by the addition of 1.6 μL of a 10 mM stock DMSO solution and the buffer. The vial was mixed with a vortex mixer and shaken for 3 h in the dark at 23 °C. Subsequently, the aqueous and organic phases were sampled with a syringe into a new HPLC vial, and the samples were analyzed using UHPLC/DAD.

#### 4.5.2 PAMPA Assay

The parallel artificial membrane permeability assay (PAMPA)^53,98^ was carried out in a coated 96-well membrane filter. The filter membrane of the donor plate was coated with PBL (Polar Brain Lipid, Avanti, USA) in dodecane (4 μL of 20 mg/mL PBL in dodecane), and the acceptor well was filled with 300 μL of PBS (pH 7.4; *V*_A_). The tested compounds were dissolved first in DMSO and subsequently diluted with PBS (pH 7.4) to final concentrations of 100 μM in the donor wells. Concentration of DMSO did not exceed 0.5% [v/v] in the donor solution. The final concentration of DMSO did not exceed 0.5% [v/v] in the donor solution. About 300 μL of the donor solution was added to the donor wells (*V*_D_), and the donor filter plate was carefully put on the acceptor plate so that the coated membrane was “in touch” with both the donor solution and acceptor buffer. In principle, the test compound diffused from the donor well through the lipid membrane (Area = 0.28 cm^2^) to the acceptor well. The concentration of the drug in both donor and the acceptor wells was assessed after 3, 4, 5, and 6 h of incubation in quadruplicate using the UV plate reader Spark (Tecan Group Ltd., Switzerland) at the maximum absorption wavelength of each compound. Besides that, a solution of theoretical compound concentration, simulating the equilibrium state established if the membrane were ideally permeable, was prepared and assessed as well. Concentration of the compounds in the donor and acceptor wells and equilibrium concentration were calculated from the standard curve and expressed as the permeability (*Pe*) according to the equation:

$$Pe=C\times -\ln\left(1-\frac{[\mathrm{drug}{]}_{\mathrm{acceptor}}}{[\mathrm{drug}{]}_{\mathrm{equilibrium}}}\right)$$

where

$$C=\left(\frac{V_{D}\times V_{A}}{(V_{D}+V_{A})\times \mathrm{Area}\times \mathrm{Time}}\right)$$

#### 4.5.3 Microsomal Stability Assay

Mixture in total volume 100 μL consisted of 5 μL of human liver microsomes (Sekisui Xenotech Xtreme 200, PN H2620), 10 μL of NADPH generating system, 10 μL of 100 μM solution of compound **94** (or **95**) in 10 mM sodium phosphate buffer, pH 7.4. Blank samples excluded either microsomes or compounds. After 5 h incubation at 37 °C, the reaction was stopped by adding MeOH (100 μL) and MeCN (100 μL), vortexed for 15 min, and filtered through a 0.22 μm PTFE syringe filter into an HPLC vial. Metabolites were detected using an Agilent Infinity II 1290 UHPLC system coupled to an Agilent 6470 QqQ mass spectrometer. Chromatographic conditions were maintained at gradient elution of 0.4 mL/min by 0.1% formic acid in water and MeCN (0–0.5 min 95:5, 0.5–3.0 gradient to 5:95, 3.0–4.0 5:95, 4.0–5.0 95:5), thermostated autosampler set to 15 °C and column thermostat equipped with Zorbax Eclipse plus RRHD C18 2.1 × 50 mm, 1.8 μm (PN 959757-902) column kept to 30 °C. MS analysis was performed in MS2 scan in the range 100–800, 500 ms scan time, 100 V fragmentor, 5 V cell acceleration voltage, positive mode. MS source parameters were set as follows: drying gas temperature 300 °C, drying gas flow 8 L/min, nebulizer pressure 35 psi, sheath gas temperature 400 °C, sheath gas flow 11 L/min, capillary voltage 2500 V.

### 4.6 In Vivo Assays

#### 4.6.1 Behavioral Testing Protocol

In the two mouse models used (the scopolamine model and the LPS model)^61,62^ selected behavioral assays were applied to assess the effect of compounds **94**, **95** and rivastigmine on distinct types of learning and memory, namely, the passive avoidance task (PA), the novel object recognition task (NOR) and the Morris water maze task (MWM). In order to assess the compounds’ effect on animals’ motor coordination, two assays, i.e., the locomotor activity test and the rotarod test, were additionally used. In the scopolamine model, the PA and NOR tasks were used for dose selection for further testing. The latter comprised the assessment of the effect of test compounds on locomotor activity and motor coordination measured in the locomotor activity and rotarod tests, and the effect of subchronically administered compounds **94**, **95**, rivastigmine, and neflamapimod in the LPS model: MWM task. The compounds were administered for 10 consecutive days, and on the last 6 days of their administration, LPS was also administered intraperitoneally 5 h before the test compounds. On these 6 days, the acquisition phase of the MWM task was performed. One day later, the retention, drug-off trial of the MWM task was carried out.

#### 4.6.2 Animals and Housing Conditions

In this in vivo study, adult male Albino Swiss (CD-1) mice weighing between 18 and 22 g, purchased from the Animal Breeding Farm of the Faculty of Pharmacy, Jagiellonian University Medical College, were used in the scopolamine model. In the LPS model adult male C57BL/6J mice (Animal Breeding Farm of the Faculty of Pharmacy, Jagiellonian University Medical College) of similar weight and age were used. The animals were housed in cages (10 mice per cage) at a constant temperature of 22 ± 2 °C, humidity of 55 ± 10% and a light/dark (12:12) cycle. The mice had unlimited access to food and water prior to the experiment. Specified conditions for the maintenance of mice were ensured throughout the experiment, including tree bedding (Transwior, Poland) and cage enrichment (tunnels, nesting material, wooden igloos, etc.). For behavioral tests, the mice were selected randomly; each group consisted of 7–10 mice. The experiments were performed between 9 AM and 5 PM. After in vivo tests, the animals were immediately euthanized, and their tissues were collected and stored for further ex vivo testing. The procedures for maintenance and treatment of laboratory animals were approved by the first Local Ethics Committee in Krakow (Approval No. 645/2022, 760/2023, and 829/2024 and the treatment of animals was in full accordance with ethical standards laid down in respective Polish and EU regulations (Directive 2010/63/EU).

#### 4.6.3 Drugs and Dose Selection for the in Vivo Tests

Scopolamine hydrobromide (Sigma-Aldrich, Poland) at the dose of 1 mg/kg was used to induce learning and memory deficits. For the in vivo tests, scopolamine was prepared in distilled water (Polfa Kutno, Poland) and was administered subcutaneously (s.c.) 30 min before behavioral tests during the acquisition (training) trial of PA and NOR tasks. LPS (Lipopolysaccharide from *Escherichia coli* O111:B4, Sigma-Aldrich, Poland) was used to induce neuroinflammation in mice.^62^ For this purpose, it was freshly prepared in distilled water (Polfa Kutno, Poland) and was administered to mice at doses of 2.5 mg/kg (2 days), 2 mg/kg (2 days), and 1.5 mg/kg (2 days). Compounds **94**, **95**, rivastigmine, and neflamapimod were suspended in 1% Tween 80 (Sigma-Aldrich, Poland). They were administered intraperitoneally (i.p.) 60 min before the acquisition trial of the PA, NOR, and MWM tasks. In the rotarod and locomotor activity tests, compounds **94** and **95**, rivastigmine and neflamapimod, were also injected i.p. 60 min before testing. As mentioned above, in the scopolamine model, the PA and NOR tasks were used as screening assays for the evaluation of antiamnesic properties of **94** and **95** and for dose selection for further experiments. Hence, in these assays, the pharmacological activity of 2 doses of **94** and **95**, namely 5 and 10 mg/kg, was assessed. In the rotarod, locomotor activity, and MWM tests, only one dose effective in the scopolamine model of amnesia was evaluated.

#### 4.6.4 Passive Avoidance Task

The PA apparatus (Panlab Harvard Apparatus, Spain) consists of a large white-colored illuminated compartment (26 cm × 26 cm × 34 cm) and a small black-colored compartment (13 cm × 7.5 cm × 7.5 cm), which are separated from each other by a guillotine gate (5 cm × 5 cm). The PA task is divided into two trials (i.e., the acquisition trial and the retention trial), each conducted 24 h apart.^70^ On the first day, during the acquisition trial (a conditioning phase), the test compounds (**94** and **95**, neflamapimod and vehicle) were injected. Also, before the acquisition trial, scopolamine or LPS was administered at selected time points. Then, each mouse was placed into the white compartment, and a 30 s habituation period began (guillotine gate is closed). After that, the guillotine gate was opened, and the mice were tested during the 180 s testing period. As soon as the mouse entered the black compartment of the PA device, the guillotine gate was closed and an electrical shock (intensity: 0.2 mA, duration: 2 s) was automatically applied through the grid floor. For each mouse, the latency between the opening of the guillotine gate and the mouse’s entering the black compartment was measured. On the next day, during the retention (drug-off) trial, the mice were placed again into the white compartment for a 30 s habituation period, and the latency to enter the black compartment was measured for each mouse. Increased latency to enter the black compartment in the retention trial in scopolamine/LPS + **94**/**95**/neflamapimod-treated groups compared to that measured in scopolamine/LPS-treated control was considered as an indicator of the compound’s antiamnesic (procognitive) properties.

#### 4.6.5 Novel Object Recognition Task

The experiment was conducted in opaque black boxes with dimensions of 50 × 50 × 50 cm.^99^ The 3 day procedure consists of habituation to the test arena (without any objects) for 10 min on the first day (T0), a training trial (T1), and a testing trial (T2) separated by a 24 h intertrial interval.^100^ During T1 (familiarization trial), two identical objects (A and B) were presented in the opposite corners of the arena, approximately 5 cm from its walls. During T2 (recognition trial), one of the objects (B) was replaced by a novel object (C), so that the animals were presented with A (familiar) and C (novel) objects. Both T1 and T2 trials lasted for 10 min, and after each trial, the animals returned to their home cages. White plastic round containers (height 5 cm, diameter 5 cm) attached to the ground with double-sided tape were used as the A, B objects, and green cuboids of similar height were used as object C. The objects were stable enough so that the animals could not displace them. The sequence of presentations and the location of the objects were randomly assigned to each mouse. The animals explored the objects by looking, licking, sniffing, or touching them while sniffing, but not when leaning against, standing, or sitting on the objects. Any mouse exploring the two objects for less than 5 s within the 10 min duration of T1 or T2 was eliminated from the study. An experimenter, blind to the drug treatment, measured the exploration time of the objects. Based on exploration time (E) of the two objects during T2, the discrimination index (DI) was calculated according to the formula: DI = (EC – EA)/(EA + EC) × 100%. In this task, scores approaching zero reflect no preference, while positive values reflect preference for the novel object and negative numbers reflect preference for the familiar object.

#### 4.6.6 Morris Water Maze Task

The Morris water maze is a circular, plastic, and gray-painted pool (120 cm in diameter and 60 cm in height), filled with water (up to about 48 cm below the edge to prevent an animal from jumping out), maintained at 23 ± 1 °C. The pool was divided into four equal quadrants (compass locations: NE, NW, SE, SW) by a computerized video tracking system (SMART, ver. 3.0; Panlab, Spain). An escape platform (11 cm in diameter and 47 cm in height) at a fixed location (the center of the NW quadrant, i.e., the target quadrant) was made of transparent Plexiglas, being invisible to the swimming animal, and was immersed 1 cm under the surface of water. The maze was lighted with an intensity of 45 lx. During the spatial acquisition trial (6 consecutive days) mice were assigned to training sessions (four training sessions a day; sessions were held 4 h apart) in which the mice were trained to escape from water by reaching a hidden platform whose location could be identified using distal extra-maze cues (A4-size sheets of black laminated paper with color geometric symbols) attached to the room walls and constituted navigation points. Visual cues had different colors and dimensions and were kept constant during the whole experiment. The whole experiment was conducted by an experimenter who remained always stationary in a constant location, being an additional, distal cue for swimming animals. For each trial, the animal was placed in the water starting from a different randomly chosen quadrant that did not contain the platform, whereas the platform was always positioned in the same place. If an animal did not find the hidden platform within 60 s, it was gently placed on the platform for 15 s. The time taken to reach the hidden platform (escape latency) on days 1–6 was recorded for each mouse and analyzed. Each animal was released from a different starting point and was allowed to swim for 60 s. If a mouse did not find the platform’s place within 60 s, it was given a latency score of 60 s. On the seventh day (a drug-off trial conducted 24 h after the last training trial), the platform was removed from the pool, and a probe trial was performed (without drug treatment—assessment of memory retention). On day 7, for each mouse, the following parameters: (1) latency to NW zone entrance, (2) time in NW zone, (3) time spent at target zone (i.e., the former platform location), and (4) the number of NW zone entries were recorded and then analyzed.

#### 4.6.7 Rotarod Test

Three days before the rotarod test, the mice were trained on the rotarod apparatus (Rotarod apparatus, May Commat RR0711, Turkey; rod diameter: 2 cm) that was rotated at a fixed speed of 18 rpm. In each training session, the mice were placed on the rotating rod for 3 min with an unlimited number of trials.^101^ The proper experiment was performed 24 h after the last training trial. Sixty minutes after the administration of compound **94**, **95**, rivastigmine, neflamapimod, or vehicle, the mice were tested on the rod that revolved at 6, 18, and 24 rpm. Motor impairments in mice were defined as the inability to remain on the rotarod apparatus for 1 min. The results are expressed as the mean time spent on the rotarod.

#### 4.6.8 Locomotor Activity Test

The locomotor activity test was performed using activity cages (40 cm × 40 cm × 30 cm, supplied with IR beam emitters) (Activity Cage 7441, Ugo Basile, Italy) connected to a counter for the recording of light-beam interrupts. Before the experiment, the mice were intraperitoneally pretreated with compound **94**, **95**, rivastigmine, neflamapimod, or vehicle, and then, they were individually placed in the activity cages in a sound-attenuated room. The animals’ movements (i.e., the number of light-beam crossings) were counted during the next 60 min of the test. Before the experiment, the mice were habituated to activity cages for 15 min.

### 4.7 Ex Vivo Assays

#### 4.7.1 Western Blot Analysis

After the completion of cognitive assays carried out in the LPS model, the mice were sacrificed, and their brains were collected and stored for further analysis. In the *ex vivo* part of the study, murine brains were homogenized using T-PER mammalian protein extraction reagent (Thermo Fisher Scientific, Waltham, MA, USA) with protease (Merck Millipore, Burlington, MA, USA) and phosphatase inhibitors (Cayman Chemical, Ann Arbor, MI, USA). The total protein concentrations were determined using the Bradford reaction. Forty μg of the total proteins were solubilized in Laemmli buffer with 2% 2-mercaptoethanol and were subject to 10% SDS-polyacrylamide gel electrophoresis. The anticyclooxygenase 2 (COX-2, diluted 1:1000), antitoll-like receptor 4 (TLR4, diluted 1:1000), anticytosolic phospholipase A2 (cPLA2, 1:1000), and anti-inflammasome (NLPR3, diluted 1:500) were used (Thermo Fisher Scientific, Waltham, MA, USA). The endogenous control was β-actin (1:1000) (Thermo Fisher Scientific, Waltham, MA, USA). The secondary antibody was antirabbit IgG (HRP) diluted 1:2000 (Thermo Fisher Scientific, Waltham, MA, USA). Proteins were detected using the Clarity Western ECL Luminol Substrate (Bio-Rad, Hercules, CA, USA). Chemi Doc Camera with Image Lab 6.1 software (Bio-Rad) was used to quantify the integrated optical density of the bands. The results were normalized to the data obtained for β-actin after stripping the PVDF membrane using Restore Western Blot Stripping Buffer (Thermo Fisher Scientific, Waltham, MA, USA) and expressed as relative to the control. This thorough process ensures the validity and reliability of the results.

### 4.8 Data Analysis

For the analysis of data obtained in the in vivo, ex vivo tests, GraphPad Prism software (v. 9.0, CA, USA) was used. Numerical results from the experiments were expressed as the mean ± standard error of the mean (SEM). Repeated measures of analysis of variance (ANOVA), one-way ANOVA, followed by Dunnett’s or Sidak’s post hoc test, were used for the statistical analysis of the results. In each case, *p* < 0.05 was considered significant.

### 4.9 Pharmacokinetic Study

In this study, male CD-1 mice weighing 27–34 g housed in conditions of constant temperature with a 12:12 h light-dark cycle with free access to food and water were used. Both tested and reference compounds were suspended in 1% Tween in water for injection (Polpharma, Poland) and administered intraperitoneally (i.p.). The mice were sacrificed by decapitation under isoflurane anesthesia at different time points following compound administration. Brains were harvested at the same time points. Blood was allowed to clot at room temperature for 20 min, and serum was separated by centrifugation at 8 000*g* (Eppendorf MiniSpin centrifuge, Germany) for 10 min. All samples were stored at −80 °C until analysis. The animal procedures were approved by the first Local Ethics Committee in Krakow (Approval No. 760/2023).

#### 4.9.1 Analytical Method

##### 4.9.1.1 Apparatus and Method Development

The quantification of rivastigmine, neflamapimod, and the investigational compounds **94** and **95** in murine serum and brain homogenates was performed using high-performance liquid chromatography coupled with tandem mass spectrometry (HPLC-MS/MS). The analytical workflow employed a Sciex QTRAP 4500 triple quadrupole mass spectrometer interfaced with an Exion LC AC HPLC system (Danaher Corporation, USA). Chromatographic separation was achieved on an XBridge C18 column (3 × 50 mm, 5 μm; Waters, USA), utilizing a mobile phase composed of acetonitrile and water both containing 0.1% formic acid. The column temperature was maintained at 40 °C, and a gradient elution protocol (Table S10) was implemented to ensure optimal analyte resolution and retention times. Mass spectrometric detection was conducted in unit resolution mode, with ion transitions specified in Table S11. For each analyte, the primary ion pair was used for quantification, while a secondary transition was monitored for confirmation of identity—except in the case of rivastigmine, for which only a single transition was tracked. Valsartan served as the internal standard and was monitored at *m*/*z* 436 → 207 (collision energy = 42 eV). Positive electrospray ionization (ESI+) was employed to enhance detection sensitivity. Ion path parameters (Table S11) were optimized via direct infusion of analyte solutions (7 μL/min) using a syringe pump. The optimized source conditions included an ion spray voltage of 5500 V, a source temperature of 450 °C, curtain gas pressure at 20 psi, and medium collision gas pressure. The method fulfilled the U.S. FDA guidelines for bioanalytical method validation, demonstrating acceptable accuracy and precision. No significant matrix effects or stability concerns were observed during routine sample analysis. Data acquisition and processing were carried out using Analyst software version 1.7.

##### 4.9.1.2 Preparation of Calibration Samples

Primary stock solutions of rivastigmine, neflamapimod, and the test compounds **94** and **95** were prepared in methanol at a concentration of 1 mg/mL. Serial dilutions were performed to generate working solutions ranging from 0.01 to 20 μg/mL, corresponding to final calibration concentrations of 0.001–2 μg/mL. For calibration curve construction, 5 μL of each working solution was spiked into 45 μL of the relevant biological matrix (serum or brain homogenate) and vortexed for 10 s. Sample preparation involved protein precipitation, in a 1:3 (v/v) ratio, using acetonitrile containing 0.1% formic acid and the internal standard. The mixtures were agitated for 10 min using an IKA Vibrax VXR shaker (Germany), followed by centrifugation at 8000 × *g* for 5 min (Eppendorf miniSpin, Germany). The resulting supernatants were transferred to chromatographic vials for analysis. Calibration curves were generated by plotting the ratio of analyte to internal standard peak areas against nominal concentrations, applying weighted (1/*x* ^2^) linear regression. Quantitative ranges were established as follows: 0.001–2 μg/mL for rivastigmine, **94**, and **95**; 0.01–2 μg/mL for neflamapimod in serum; and 0.005–10 μg/g (or 0.05–10 μg/g for neflamapimod) in tissue homogenates.

##### 4.9.1.3 Sample Preparation

Brain tissues were homogenized in distilled water at a 1:4 (w/v) ratio using an ULTRA-TURRAX T10 basic homogenizer (IKA, Germany). Aliquots of brain homogenate or serum (50 μL) were subjected to protein precipitation in a 1:3 (v/v) ratio with acetonitrile containing 0.1% formic acid and the internal standard. The mixtures were agitated for 10 min using an IKA Vibrax VXR shaker (Germany), followed by centrifugation at 8000 × *g* for 5 min (Eppendorf miniSpin, Germany). The resulting supernatants were directly transferred to chromatographic vials for analysis. In cases where analyte concentrations in serum exceeded 20 μg/mL, samples were diluted with blank matrix prior to the deproteinization step. The autosampler was maintained at 15 °C, and a 1 μL aliquot was injected onto the analytical column. Representative chromatograms are presented in Figure S10.

#### 4.9.2 Pharmacokinetic Data Analysis

Pharmacokinetic parameters were estimated using the noncompartmental analysis (NCA). The maximum concentration (*C*_max_) and the time to reach maximum concentration (*t*_max_) in serum and brain were obtained directly from the concentration versus time data. The terminal elimination rate constant (λ_*z*_) was estimated by linear regression, and the terminal half-life (*t*_0.5λz_) was calculated as ln 2/λ_*z*_. The area under the concentration versus time curve from the time of dosing to the last measured point (AUC_0–*t*_) and to infinity (AUC_0–∞_) was calculated by the linear trapezoidal rule. The clearance (CL/F) was estimated as Dose/AUC_0–∞_ and the volume of distribution based on the terminal phase (*V*_*z*_/*F*) was calculated according to the equation: *D*/(λ_*z*_·AUC_0–∞_), where *F* is the fraction absorbed. The mean residence time (MRT) was calculated as AUC_0–∞_/AUMC_0–∞_, where AMUC_0–∞_ is the area under the first moment curve from the time of dosing to infinity.

## Supplementary Material





## Notes

Eight docking hit compounds from Molport; complete list of synthesized ARRY-371797 derivatives with substitution on the *N* ^1^ and *N* ^2^ nitrogens, respectively, with their corresponding in vitro activities on hAChE, hBChE, and human p38α MAPK; profiling of compounds **94** and **95** on a panel of 103 kinases; data collection and refinement statistics of the crystal structures of compounds **94** and **95** with hBChE and compounds **94** and **95** with human p38α MAPK; prediction of BBB permeation of **94** and **95** by BBB-Pampa Assay; metabolites of **94** and **95** after a 5 h incubation with human liver microsomes; in vivo pharmacokinetic parameters of rivastigmine and neflamapimod following i.p. administration to mice; gradient of MF used to determine compound **94**, **95**, rivastigmine and neflamapimod in mouse serum and brain homogenates and precursor/product ion transitions and optimal ion path parameters for neflamapimod, rivastigmine, and compounds **94** and **95** determined in mouse serum and brain homogenates; general synthetic scheme of the synthesis of ARRY-371797 derivatives with substitution on the *N* ^1^ and *N* ^2^ nitrogens, respectively; inhibition of BChE from the SH-SY5Y cell line treated by compounds **94** and **95**; the crystal structure of compound **102** in the hBChE active site; the overlay of the docking and crystal structure poses of ARRY-371797 and compound **95** in hBChE; in vitro cytotoxicity; neuro- and gliaprotective properties of **94** and **95**; the results of the in vivo assay of **94** and **95** on the rotarod and locomotor activity test; and mean serum and brain concentrations of rivastigmine and neflamapimod after i.p. administration to mice and representative chromatograms of serum calibration samples for rivastigmine compounds **94**, **95,** and neflamapimod (PDF)
Molecular formula strings (CSV)

## Notes

The coordinates and structure factors of the complexes of compounds **94**, **95**, and **102** with human butyrylcholinesterase (9I5O, 9I5P, and 9I5Q, respectively) and compounds **94** and **95** with human p38α MAP kinase (9D7N and 9D75, respectively) have been deposited to the Protein Data Bank (PDB) Authors will release the atomic coordinates upon article publication.

## Abbreviations

Aβ: amyloid beta
Aβ_40_: amyloid beta 40
Aβ_42_: amyloid beta 42
ACh: acetylcholine
AChE: acetylcholinesterase
AD: Alzheimer’s disease
ADP-Glo: adenosine diphosphate glow assay
BACE-1: beta-secretase 1
BChE: butyrylcholinesterase
ChE: cholinesterase
ChEIs: cholinesterase inhibitors
DCM: dichloromethane
DIPEA: *N*,*N*-diisopropylethylamine
DMF: dimethylformamide
DMSO: dimethyl sulfoxide
DFG: aspartate-phenylalanine-glycine motif in kinases
Et_3_N: triethylamine
EtOH: ethanol
FDA: Food and Drug Administration
GSK-3β: glycogen synthase kinase-3 beta
HRI: hydrophobic region I in p38α MAPK active site
HRII: hydrophobic region II in p38α MAPK active site
IC_50_: half maximal inhibitory concentration
IL-1β: interleukin-1 beta
LiOH­(aq): lithium hydroxide aqueous solution
MAPK: mitogen-activated protein kinase
MD: molecular dynamics simulations
MeI: methyl iodide
MTDLs: multitarget directed ligands
MW: microwave irradiation used in synthesis
NaOH­(aq): sodium hydroxide aqueous solution
NDDs: neurodegenerative diseases
PAS: peripheral aromatic site of cholinesterase
PDE9A: phosphodiesterase 9A
PDB: Protein Data Bank
RA: residual enzymatic activity
SARs: structure–activity relationships
SN2: bimolecular nucleophilic substitution reaction mechanism
TBTU: *O*-(benzotriazol-1-yl)-*N*,*N*,*N′*,*N*′**-tetramethyluronium tetrafluoroborate
