# Supplementary material for: Targeting Neuroinflammation and Cognitive Decline: First-in-Class Dual Butyrylcholinesterase and p38α Mitogen-Activated Protein Kinase Inhibitors
Source: J Med Chem. 2025 Aug 8;68(16):17378–411. doi: 10.1021/acs.jmedchem.5c00933 (PMC12406203; doi:10.1021/acs.jmedchem.5c00933)
Supplement: Supplementary file 1 [file jm5c00933_si_001.pdf]

Supporting Information

Targeting Neuroinflammation and Cognitive Decline: First-in-Class Dual Butyrylcholinesterase and p38α Mitogen-Activated Protein Kinase Inhibitors

Svit Ferjančič Benetik[a], Matic Proj[a], Damijan Knez[a], Urban Košak[a], Anže Meden[a], Katja Krajšek[a], Anja Pišlar[a], Selena Horvat[a], Urban Švajger[b], Nataša Tešić[b], Lenka Pulkrabkova[c], Ondrej Soukup[c], Adam Skarka[d], Rudolf Andrys[d], Xavier Brazzolotto[e], Alexandre Igert[e], Florian Nachon[e], Jose Dias[e], Jan Detka[f], Joanna Gdula-Argasińska[g], Elżbieta Wyska[h], Małgorzata Szafarz[h] Aleksandra Manik[f], Natalia Płachtij[f], Kamil Musílek[c-d], Kinga Sałat[f], Aleš Obreza[a], Stanislav Gobec[a*]

[a] *University of Ljubljana, Faculty of Pharmacy, Department of Pharmaceutical Chemistry, 1000 Ljubljana, Slovenia*
[b] *Department for Therapeutic Services, Blood Transfusion Center of Slovenia, 1000 Ljubljana, Slovenia*
[c] *University Hospital in Hradec Kralove, Biomedical Research Centre, 500 05 Hradec Kralove, Czech Republic*
[d] *University of Hradec Kralove, Faculty of Science, Department of Chemistry, 500 03 Hradec Kralove, Czech Republic*
[e] *Département de Toxicologie et Risques Chimiques, Institut de Recherche Biomédicale des Armées, Paris 91220, France*
[f] *Department of Pharmacodynamics, Chair of Pharmacodynamics, Faculty of Pharmacy, Jagiellonian University Medical College, Krakow 30-688, Poland*
[g] *Department of Radioligands, Faculty of Pharmacy, Jagiellonian University Medical College, 9 Medyczna St., 30-688 Krakow, Poland*
[h] *Department of Pharmacokinetics and Physical Pharmacy, Jagiellonian University Medical College, 9 Medyczna St., 30-688, Kraków, Poland*

[*] *e-mail: stanislav.gobec@ffa.uni-lj.si*

Index

Supplementary Schemes, Figures and Tables....S3

Scheme S1. Synthesis of ARRY-371797 derivatives....S3

Table S1. Eight hBChE molecular docking hit compounds purchased from Molport (Riga, Latvia)....S4

Table S2. Inhibitory activity of ARRY-371797 derivatives with a substituent on the indazole N1 atom....S6

Table S3. Inhibitory activity of ARRY-371797 derivatives with a substituent on the indazole *N*[2] atom....S12

Figure S1. Residual BChE activity in SH-SY5Y human neuroblastoma cell line following treatment with compounds 94, 95 and ethopropazine....S15

Table S4. Profiling of compounds 94 and 95 at two concentrations (1000 and 10000 nM) against 103 kinases; single measurements....S15

Figure S2. Crystal structure of 102 in the hBChE active site (PDB code 9I5Q). S20
Figure S3. Overlay of the docking pose of ARRY-371797 and the crystal structure of compound 94 in hBChE. S21
Table S5. Data collection and refinement statistics of human BChE in complex with 95, 94, and 102. ....S22
Table S6. Data collection and refinement statistics for human p38α MAPK in complex with 94 and 95. ....S23
Table S7. Prediction of blood-brain barrier (BBB) penetration of compounds 94 and 95. ...S24
Table S8. Metabolites of compounds 94 and 95 after a five-hour incubation with human liver microsomes. S25
Figure S4. *In vitro* cytotoxicity profiles of compounds 94, 95 and rivastigmine. S26
Figure S5. The impact of compounds 94 and 95 on $A\beta_{1-42}$-induced toxicity. S27
Figure S6. The impact of compounds 94 and 95 on LPS-induced microglial activation. S27
Figure S7. Effect of compounds 94 and 95 (10 mg/kg, i.p. injection) and rivastigmine (1 and 2.5 mg/kg, i.p. injection) on motor coordination measured using the rotarod test at 6 rotations per minute (rpm) (A), 18 rpm (B) and 24 rpm (C). S28
Figure S8. Effect of compounds 94 and 95 (10 mg/kg, i.p. injection) on locomotor activity measured in mice. S28
Figure S9. Mean (±SD) serum and brain concentrations of rivastigmine (A) and neflamapimod (B). S29
Table S9. Pharmacokinetic parameters of rivastigmine and neflamapimod estimated using the model independent approach in serum and brain following i.p. administration of a dose of 2.5 and 30 mg/kg, respectively, to mice. S30
Table S10. Gradient of the mobile phase composition used for the determination of neflamapimod, rivastigmine, and compounds 94 and 95 in mouse serum and brain homogenates. S30
Table S11. Monitored precursor/product ion transitions and optimal ion path parameters for neflamapimod, rivastigmine, and compounds 94 and 95 determined in mouse serum and brain homogenates. S31
Figure S10. Representative chromatograms of serum calibration samples for rivastigmine (A), compound 94 (B), neflamapimod (C), and compound 95 (D) at a concentration of 10 ng/mL. S32
^{1}H and ^{13}C NMR spectra of final compounds S33
Supplementary References S72

Supplementary Schemes, Figures and Tables

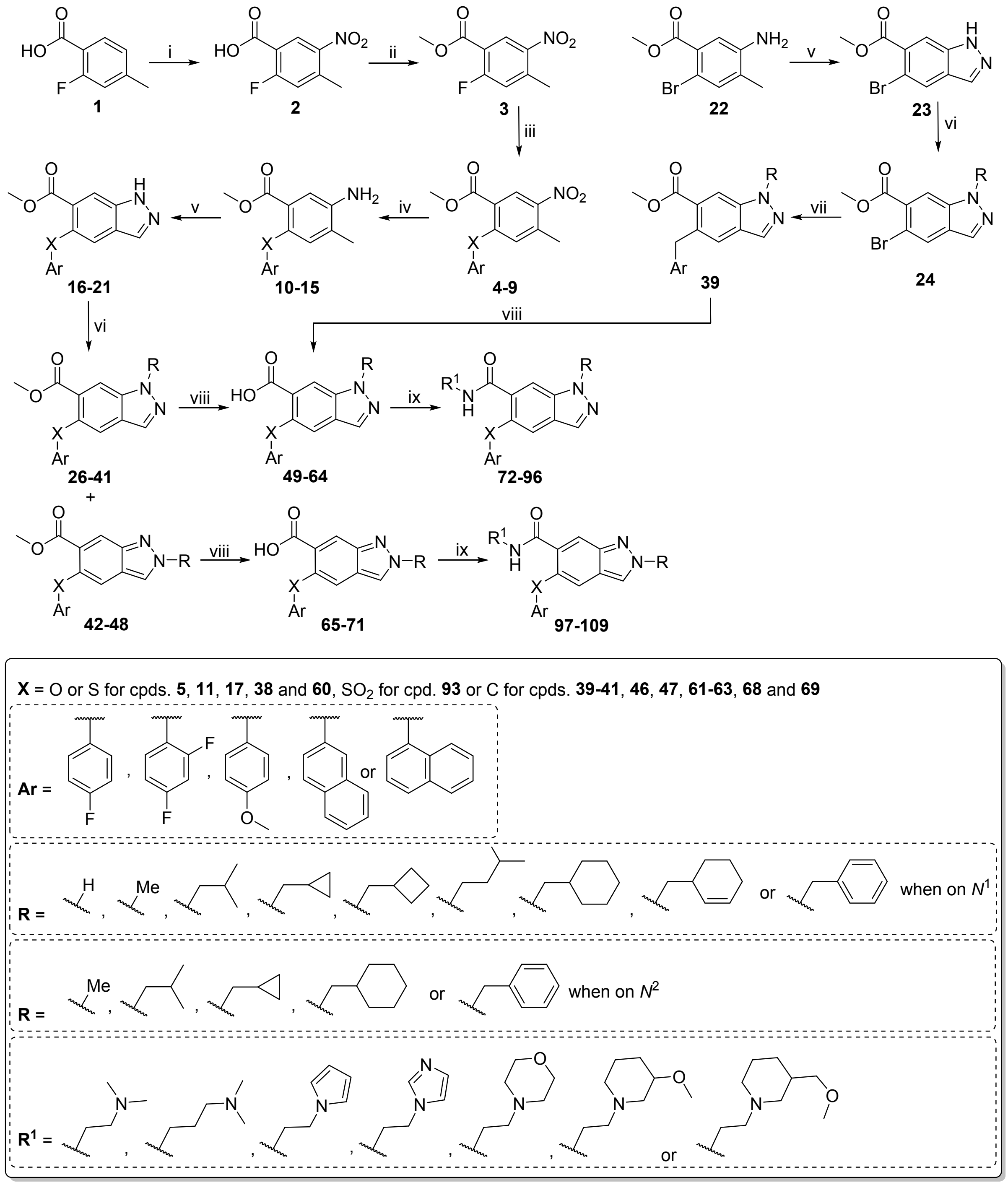

Scheme S1. Synthesis of ARRY-371797 derivatives.

Table S1. Eight hBChE molecular docking hit compounds purchased from Molport (Riga, Latvia).

Compound structure	Compound code	hBChE		p38α MAPK	
		RA at 100 μM [%]	**IC_{50} [μM]**	RA at 100 μM [%]	**IC_{50} [μM]**
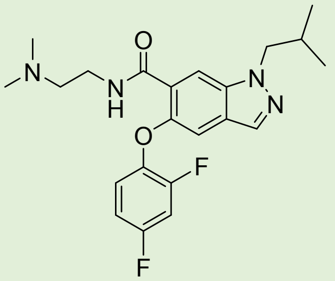	**MPB01 (Hit cpd) ARRY-371797**	9%	**12.01**	6%	**0.13**
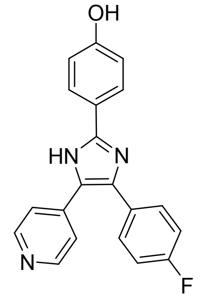	**MPB02**	84%	n.d.	2%	n.d.
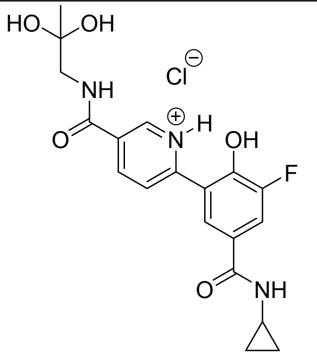	**MPB03**	85%	n.d.	2%	n.d.
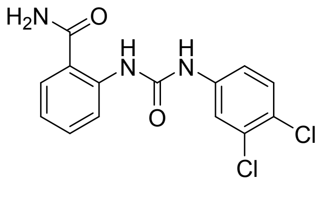	**MPB04**	17 %	**19.42**[a]	59 %	n.d.
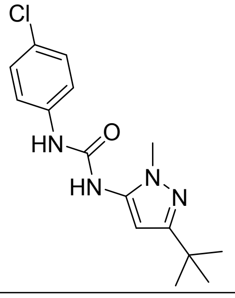	**MPB05**	70%	n.d.	10%	n.d.

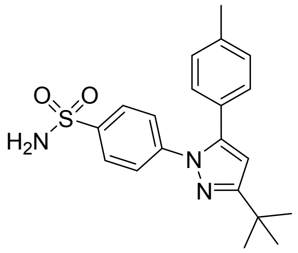	**MPB06**	89%	n.d.	34%	n.d.
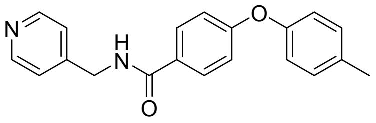	**MPB08**	95%	n.d.	n.d.	n.d.
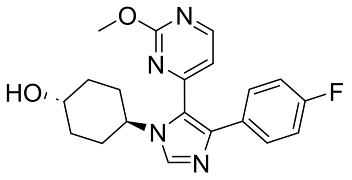	**MPB11**	91%	n.d.	1%	n.d.

All compounds were evaluated *in vitro* for hBChE and human p38α MAPK (hp38α MAPK) inhibition by the method of Ellman[1] and ADP-Glo method,[2] respectively. For RA values <50 % at 100 μM compound concentrations, the IC_{50} value was determined. n.d. = not determined; [a]Although **MPB04** is active against hBChE, this ligand is a non-specific ChE inhibitor and is poorly soluble in the kinase buffer. Compound **MPB01** is the previously published p38α MAPK inhibitor ARRY-371797[3].

Table S2. Inhibitory activity of ARRY-371797 derivatives with a substituent on the indazole N1 atom.

	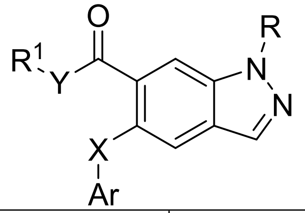					RA[a] at 100 µM [%] or **IC_{50} ± SEM[b] [µM]**		
**compound**	**R**	**X**	**Ar**	**R[1]**	**Y**	**hAChE**	**hBChE**	**p38α MAPK**
**27**	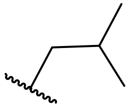	O	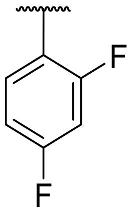	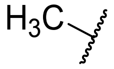	O	81.1 ± 2.3%	76.6 ± 11.7%	**0.330 ± 0.158**
**72 (hit)**	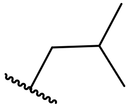	O	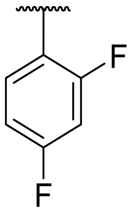	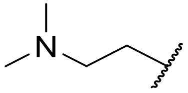	NH	53.8 ± 2.6%	**12.01 ± 1.95**	**0.132 ± 0.042**
**73**	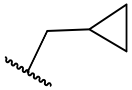	O	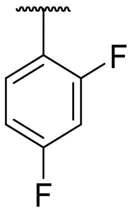	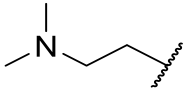	NH	**83.94 ± 28.93**	**10.46 ± 1.51**	**0.362 ± 0.139**

**74**	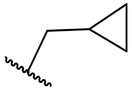	O	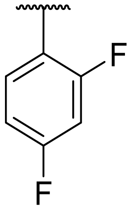	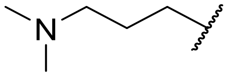	NH	**109.6 ± 38.4**	**8.68 ± 1.83**	**0.096 ± 0.011**
**75**	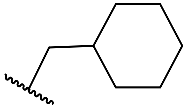	O	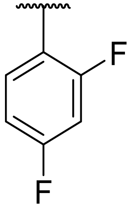	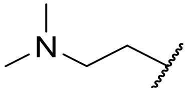	NH	**64.40 ± 24.49**	**2.895 ± 0.446**	**0.142 ± 0.079**
**76**	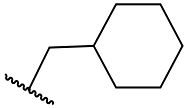	O	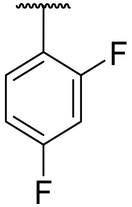	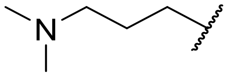	NH	51.4 ± 1.7%	**2.622 ± 0.749**	**0.108 ± 0.007**
**78**	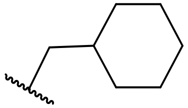	O	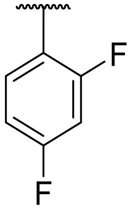	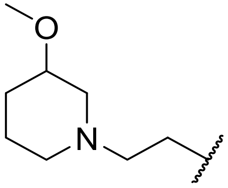	NH	*23.4 ± 1.9%	**5.219 ± 1.547**	**0.744 ± 0.191**
**79**	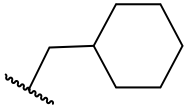	O	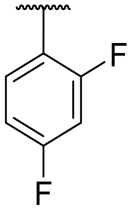	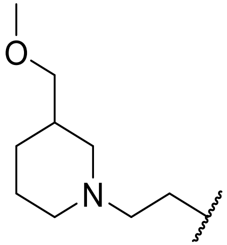	NH	*11.6 ± 1.3%	**3.599 ± 0.825**	**0.379 ± 0.126**

**80**	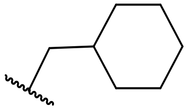	O	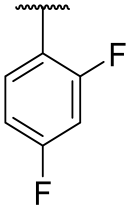	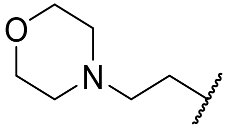	NH	51.9 ± 1.7%	**5.219 ± 1.547**	**0.744 ± 0.191**
**81**	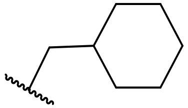	O	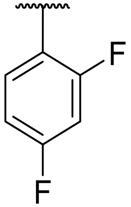	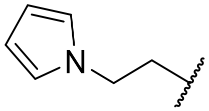	NH	58.7 ± 3.6%	**80.0 ± 4.0**	**75.8 ± 2.7**
**82**	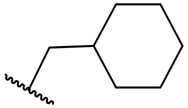	O	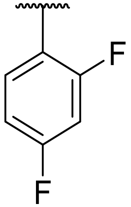	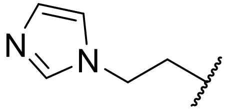	NH	65.1 ± 2.5%	**18.73 ± 6.10**	**0.218 ± 0.017**
**83**	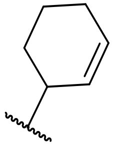	O	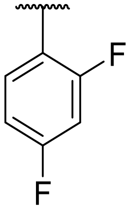	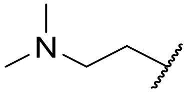	NH	52.9 ± 2.7%	**8.352 ± 1.671**	**0.088 ± 0.02**
**84**	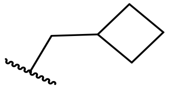	O	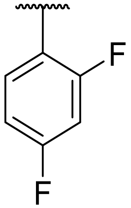	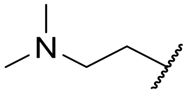	NH	51.9 ± 0.5%	**6.407 ± 0.565**	**0.142 ± 0.02**

**85**	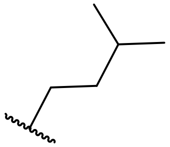	O	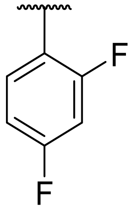	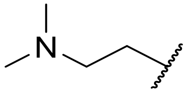	NH	51.1 ± 1.4%	**2.698 ± 0.558**	**0.189 ± 0.073**
**86**	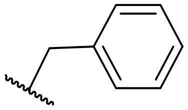	O	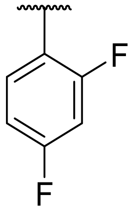	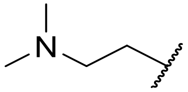	NH	[a]43.1 ± 1.1%	**3.947 ± 1.508**	**0.739 ± 0.400**
**87**	H	O	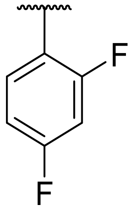	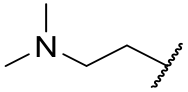	NH	80.4 ± 2.9%	**66.48 ± 21.75**	**6.03 ± 0.92**
**88**	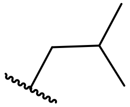	O	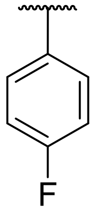	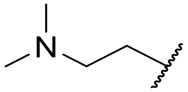	NH	**71.3 ± 4.7**	**6.382 ± 0.511**	**0.276 ± 0.015**
**89**	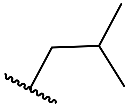	O	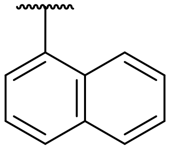	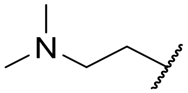	NH	**61.28 ± 22.46**	**10.56 ± 1.06**	0.9 ± 0.3%
**90**	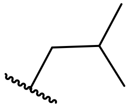	O	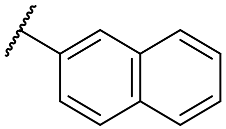	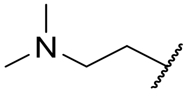	NH	**116.1 ± 27.8**	**11.48 ± 2.00**	9.5 ± 0.6%

**91**	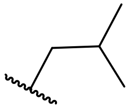	O	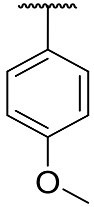	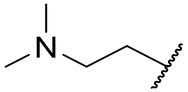	NH	75.2 ± 3.8%	54.1 ± 1.3%	0.9 ± 0.3%
**92**	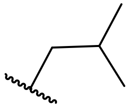	S	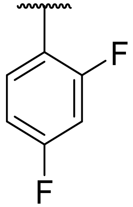	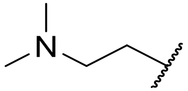	NH	62.8 ± 3.0%	**2.253 ± 0.332**	**0.053 ± 0.017**
**93**	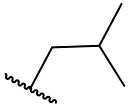	SO_2	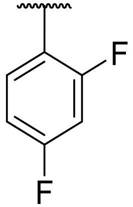	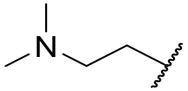	NH	98.0 ± 2.7%	77.6 ± 3.1%	5.9 ± 0.2%
**94**	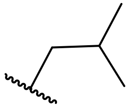	CH_2	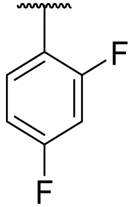	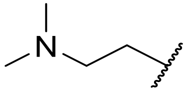	NH	69.0 ± 4.0%	**0.652 ± 0.112**	**0.377 ± 0.123**
**95**	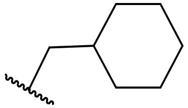	CH_2	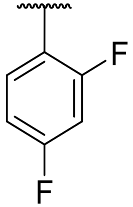	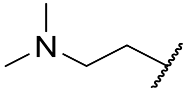	NH	53.4 ± 0.8%	**0.772 ± 0.057**	**0.191 ± 0.021**

**96**	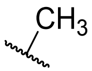	CH_2	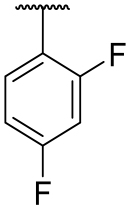	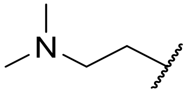	NH	78.8 ± 3.1%	**4.118 ± 0.969**	88.2 ± 5.0 %

SEM – standard error of the mean, data are the average of two independent experiments, each performed in triplicate; RA – residual activity (mean ± standard deviation of one independent experiment performed in triplicate); [a] – nonspecific inhibition at the concentration tested (100 µM) due to solubility issues, inhibition disappears upon dilution (RA at the 30 µM compound concentration above 50%); hAChE – human acetylcholinesterase; hBChE – human butyrylcholinesterase; p38α MAPK – p38α mitogen-activated protein kinase.

Table S3. Inhibitory activity of ARRY-371797 derivatives with a substituent on the indazole N^2 atom.

	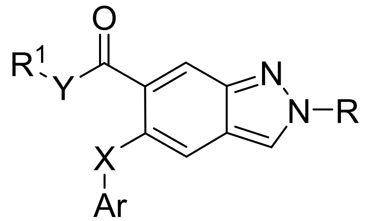					RA[a] at 100 µM [%]<br>or<br>**IC$_{50}$ ± SEM[b] [µM]**		
**Cpd number**	**R**	**X**	**Ar**	**R[1]**	**Y**	**hAChE**	**hBChE**	**p38α MAPK**
**97**	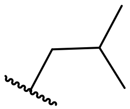	O	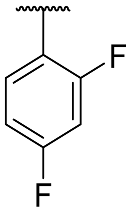	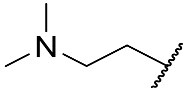	NH	51.8 ± 1.6%	**16.24 ± 4.87**	**5.79 ± 0.88**
**98**	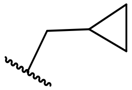	O	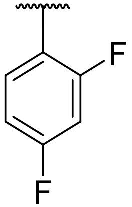	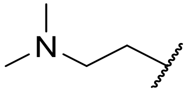	NH	56.2 ± 4.0%	**27.05 ± 8.06**	70.2 ± 4.6%
**99**	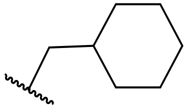	O	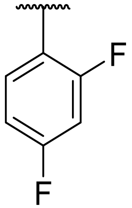	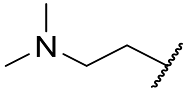	NH	**28.94 ± 8.16**	**0.167 ± 0.0203**	**6.776 ± 2.848**

**100**	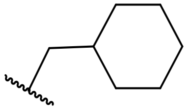	O	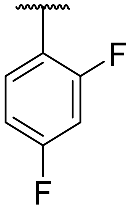	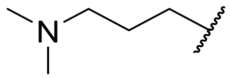	NH	**41.93 ± 13.85**	**0.499 ± 0.045**	**16.24 ± 2.97**
**101**	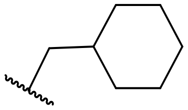	O	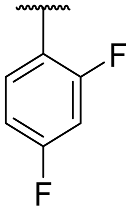	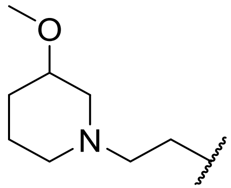	NH	51.8 ± 1.6%	**0.184 ± 0.0310**	94.3 ± 2.4%
**102**	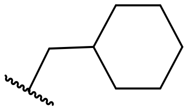	O	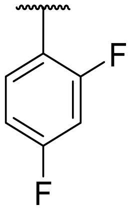	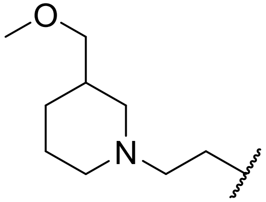	NH	50.7 ± 3.1%	**0.021 ± 0.0019**	92.7 ± 25.3%
**103**	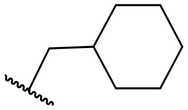	O	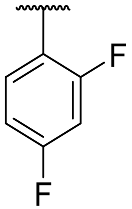	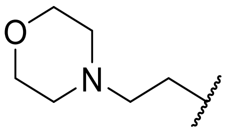	NH	78.3 ± 2.1%	**10.76 ± 2.072**	66.8 ± 5.9%
**104**	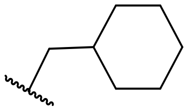	O	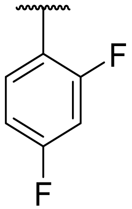	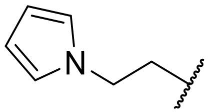	NH	67.6 ± 2.3%	[a]34.4±1.5 %	81.2 ± 6.6%

**105**	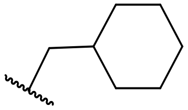	O	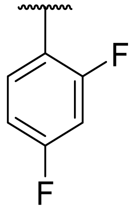	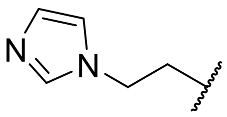	NH	55.4 ± 1.3%	**0.066 ± 0.0089**	**19.49 ± 2.87**
**106**	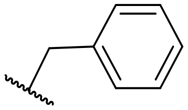	O	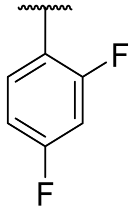	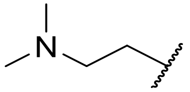	NH	102.6 ± 36.4	1.005 ± 0.133	59.8 ± 4.7%
**107**	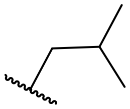	O	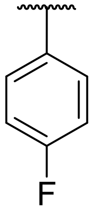	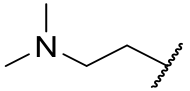	H	57.6 ± 2.2%	21.91 ± 5.74	89.3 ± 2.9%
**108**	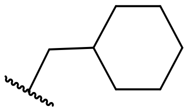	CH_2	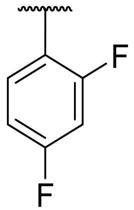	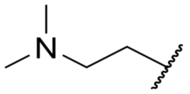	H	96.49±35.9%	**0.748 ± 0.0764**	91.0 ± 1.7%
**109**	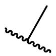	CH_2	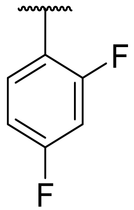	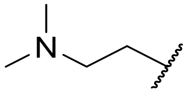	H	81.4 ± 7.5%	**1.062 ± 0.97**	89.6 ± 13.6%

SEM – standard error of the mean, data are the average of two independent experiments, each performed in triplicate; RA – residual activity (mean ± standard deviation of one independent experiment performed in triplicate); [a] – nonspecific inhibition at the concentration tested (100 µM) due to solubility issues, inhibition disappears upon dilution (RA at the 30 µM compound concentration above 50%); hAChE – human acetylcholinesterase; hBChE – human butyrylcholinesterase; p38α MAPK – p38α mitogen-activated protein kinase.

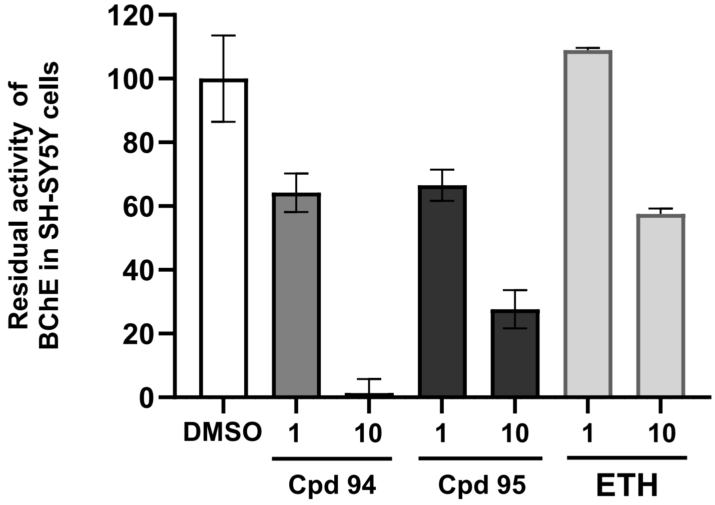

Figure S1. Residual BChE activity in SH-SY5Y human neuroblastoma cell line following treatment with compounds 94, 95 and ethopropazine. SH-SY5Y cells were suspended in PBS and treated with compounds **94**, **95** and ethopropazine (1 and 10 µM) and DMSO on 96-well plates. After the addition of Ellman's reagent and butyrylthiocholine residual activity (RA) of BChE was determined by measuring absorbance at 412 nm over a period of 120 min on a microplate reader We considered the control (DMSO; 0 µM of compound) as 100% RA and presented the data as means (± SEM) of three independent experiments, each carried out in triplicate. ETH = ethopropazine

Table S4. Profiling of compounds 94 and 95 at two concentrations (1000 and 10000 nM) against 103 kinases; single measurements.

Target	Cpd. **94**		Cpd. **95**	
	RA @ 1000nM	RA @ 10000nM	RA @ 1000nM	RA @ 10000nM
**ABL1(E255K)-phosphorylated**	96	55	96	93
**ABL1(T315I)-phosphorylated**	93	90	72	91
**ABL1-nonphosphorylated**	85	97	90	90
**ABL1-phosphorylated**	79	53	81	85
**ACVR1B**	100	100	100	100
**ADCK3**	100	100	100	81

**AKT1**	100	75	100	100
**AKT2**	89	76	100	88
**ALK**	93	83	62	92
**AURKA**	96	93	95	93
**AURKB**	84	68	99	71
**AXL**	97	89	84	83
**BMPR2**	86	95	89	92
**BRAF**	100	100	100	100
**BRAF(V600E)**	100	100	98	100
**BTK**	100	100	100	100
**CDK11**	100	89	92	100
**CDK2**	82	97	99	100
**CDK3**	100	1.3	100	100
**CDK7**	79	86	88	77
**CDK9**	100	100	100	93
**CHEK1**	100	100	100	100
**CSF1R**	100	86	100	79
**CSNK1D**	86	79	93	84
**CSNK1G2**	96	93	100	98
**DCAMKL1**	100	100	100	100
**DYRK1B**	100	85	95	100
**EGFR**	98	69	100	100
**EGFR(L858R)**	100	66	100	100
**EPHA2**	97	100	100	100
**ERBB2**	74	81	84	78
**ERBB4**	85	83	93	81
**ERK1**	87	87	96	90

**ERK 2**	100	98	100	100
**FAK**	96	89	92	94
**FGFR2**	95	100	100	100
**FGFR3**	100	100	100	92
**FLT3**	100	90	100	100
**GSK3B**	91	97	100	80
**IGF1R**	94	84	100	93
**IKKα**	82	87	92	75
**IKKβ**	89	89	93	80
**INSR**	81	90	84	81
**JAK2(JH1domain-catalytic)**	98	100	100	100
**JAK3(JH1domain-catalytic)**	100	100	100	100
**JNK1**	77	41	100	27
**JNK2**	52	8.3	44	3.8
**JNK3**	55	6.8	48	3.7
**KIT**	100	100	100	100
**KIT(D816V)**	94	100	96	94
**KIT(V559D,T670I)**	100	100	47	94
**LCK**	100	97	94	100
**LKB1**	100	96	100	86
**MAP3K4**	49	100	100	60
**MAPKAPK2**	100	84	100	100
**MAPKAPK5**	100	100	100	100
**MARK3**	100	100	79	86
**MEK1**	98	74	98	100
**MEK2**	86	80	95	100

**MET**	96	100	100	100
**MKNK1**	100	100	100	100
**MKNK2**	76	80	81	67
**MLK1**	100	100	100	90
**NLK**	100	98	100	100
**p38α**	2.3	0.4	6.7	0.1
**p38β**	72	15	84	18
**p38γ**	93	94	100	99
**p38δ**	97	100	100	100
**PAK1**	96	94	100	93
**PAK2**	100	97	100	95
**PAK4**	97	96	100	88
**PCTK1**	96	92	87	79
**PDGFRA**	96	76	92	92
**PDGFRB**	100	97	100	96
**PDPK1**	88	74	100	87
**PIK3C2B**	90	82	91	91
**PIK3CA**	77	95	100	100
**PIK3CG**	86	85	92	97
**PIM1**	95	100	100	94
**PIM2**	100	100	100	100
**PIM3**	100	100	100	100
**PKACα**	100	96	100	94
**PLK1**	100	100	100	100
**PLK3**	100	97	88	100
**PLK4**	100	100	100	100
**PRKCE**	80	80	79	76

**RAF1**	100	100	100	100
**RET**	100	100	100	100
**RIOK2**	85	92	88	81
**ROCK2**	87	96	83	100
**RSK2 (Kin.Dom.1-N-terminal)**	86	65	94	94
**SNARK**	100	93	93	92
**SRC**	100	94	100	93
**SRPK3**	77	100	92	99
**TGFBR1**	100	100	100	100
**TIE2**	100	100	90	100
**TRKA**	98	94	100	92
**TSSK1B**	77	89	100	79
**TYK2(JH1domain-catalytic)**	78	79	89	75
**ULK2**	71	88	77	75
**VEGFR2**	100	100	100	100
**YANK3**	100	100	100	100
**ZAP70**	100	79	100	100

RA – residual activity (one independent experiment performed in singlicate)

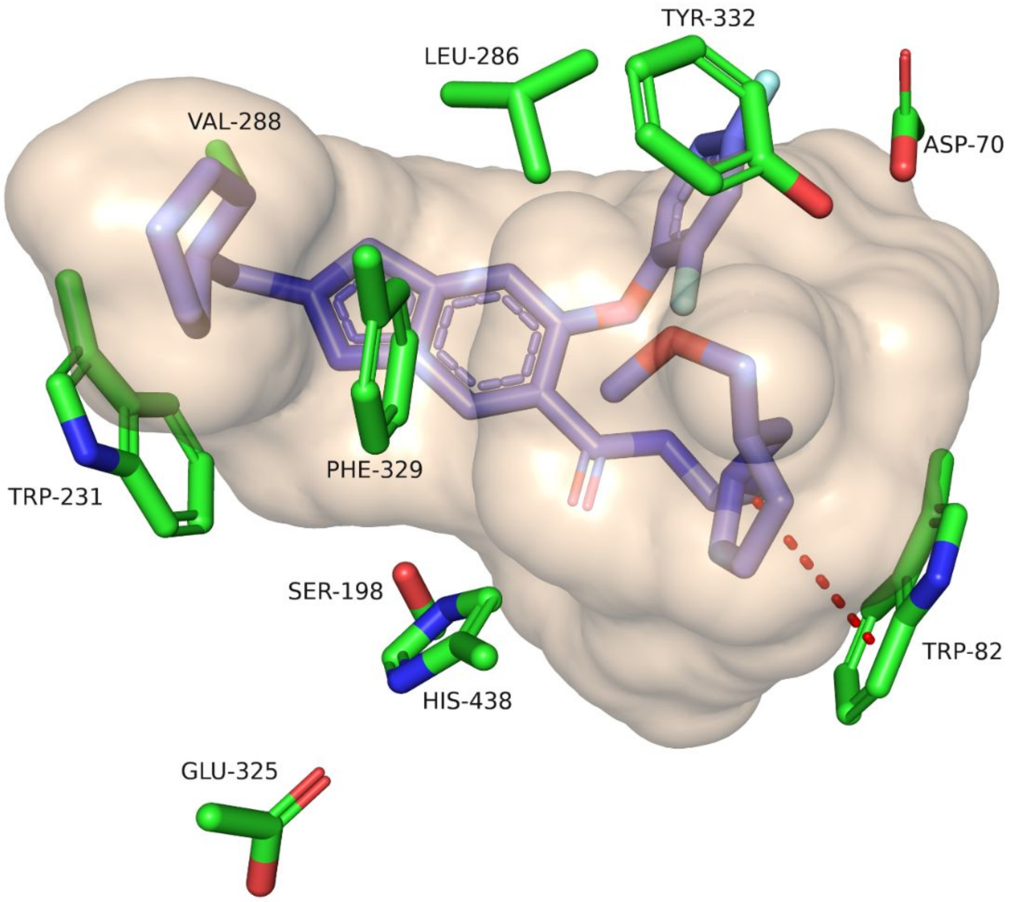

Figure S2. Crystal structure of 102 in the hBChE active site (PDB code 9I5Q). Compound **102** is shown as violet sticks; the enzyme active site gorge is displayed with wheat-coloured outer surface with the most important amino acid residues as green sticks and the acyl loop as magenta sticks. Hydrogen bond is presented with a red dashed line. The crystal structure represents how N^2-substitution of the indazole core with a bulky alkyl chain displaces the acyl loop (Val280–Val288), which positions above the methylene cyclohexyl moiety. This is energetically unfavourable. However, the 3-(methoxymethyl)-1-propylpiperidine of compound **102** fully occupies the choline-binding pocket compared, therefore, very potent inhibition of BChE is still achieved.

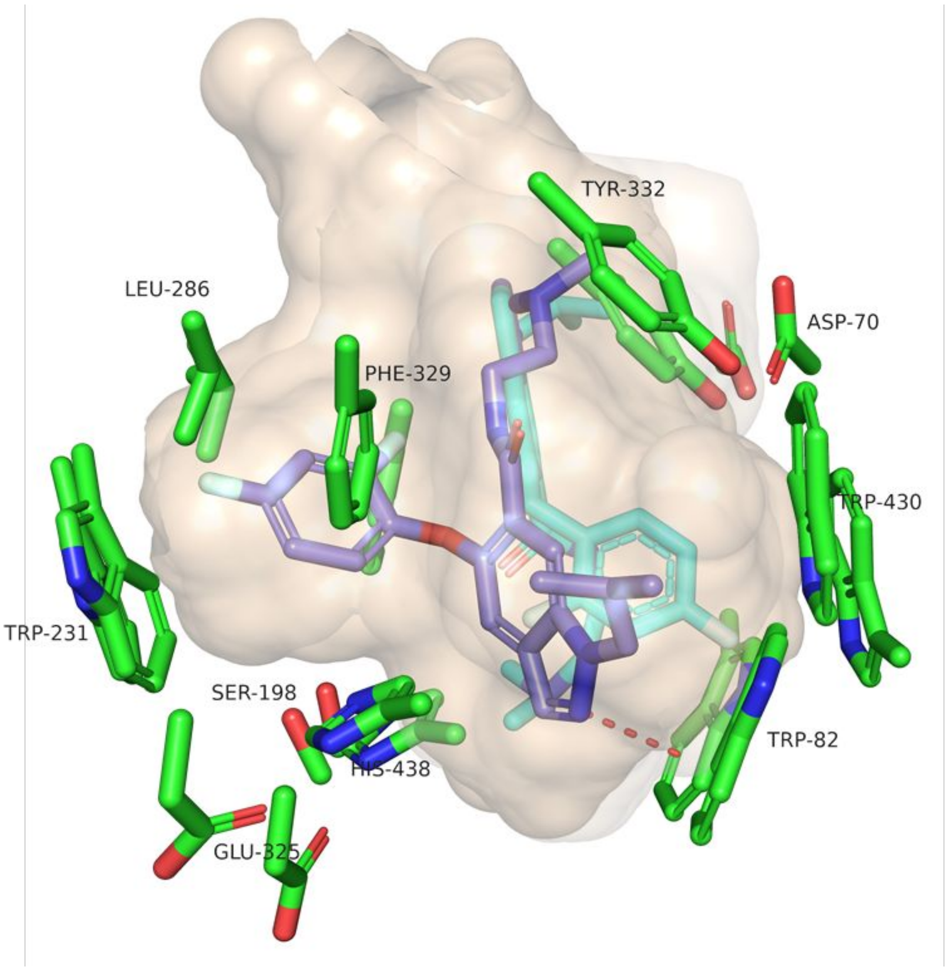

Figure S3. Overlay of the docking pose of ARRY-371797 and the crystal structure of compound 94 in hBChE. ARRY-371797 and compound **94** are presented as violet and cyan sticks, respectively. The enzyme is displayed as wheat-coloured outer surface with the most important amino acid residues as green sticks. A hydrogen bond between the tertiary amine of compound **94** and Trp82 is presented with a red dashed line. It is evident that the poses are markedly different – molecular docking placed the 2,4-difluorophenoxy moiety of ARRY-371797 in the acyl-binding pocket, the isobutyl group in the choline-binding pocket, and the tertiary amine pointing to the peripheral aromatic site. In the crystal structure of compound **94**, however, the acyl-binding pocket remains unoccupied and a strong cation-π interaction is observed between the tertiary amine moiety and Trp82 of the choline-binding pocket. The indazole core with the N^1-isobutyl group is oriented perpendicularly pointing out of the active site gorge.

Table S5. Data collection and refinement statistics of human BChE in complex with 95, 94, and 102.

	**PDB 9I5O** (Cpd. **95**)	**PDB 9I5P** (Cpd. **94**)	**PDB 9I5Q** (Cpd. **102**)
Data collection			
**X-ray source**	ESRF BM07 – FIP2	ESRF ID30A-3	ID30B
**Wavelength (Å)**	0.9795	0.9677	0.8731
**Resolution range (Å)**	49.17 - 2.66 (2.78 - 2.66)	47.63 - 2.75 (2.9 - 2.75)	41.51 - 2.36 (2.44 - 2.36)
**Space group**	I 4 2 2	I 4 2 2	I 4 2 2
**Unit cell (Å,°)**	154.484 154.484 127.527 90 90 90	154.08 154.08 127.253 90 90 90	155.391 155.391 126.766 90 90 90
**Total reflections**	182915 (23237)	183502 (27301)	263326 (23997)
**Unique reflections**	21986 (2739)	20122 (2848)	31344 (2803)
**Multiplicity**	8.3 (8.5)	9.1 (9.6)	8.4 (8.6)
**Completeness (%)**	97.23 (99.60)	99.54 (99.96)	97.92 (96.82)
**Mean I/sigma(I)**	8.02 (2.48)	9.23 (1.47)	10.98 (1.18)
**Wilson B-factor (Å^2)**	38.02	60.39	53.14
**R-merge**	0.2312 (0.8621)	0.1721 (1.422)	0.1105 (1.282)
**R-meas**	0.2466 (0.9156)	0.1825 (1.506)	0.1176 (1.366)
**R-pim**	0.08154 (0.2953)	0.0596 (0.4869)	0.03898 (0.4565)
**CC1/2**	0.992 (0.455)	0.996 (0.585)	0.999 (0.471)
**CC***	0.998 (0.791)	0.999 (0.859)	1 (0.8)
Refinement statistics			
**Reflections used in refinement**	21826 (2739)	20117 (2847)	31305 (2771)
**Reflections used for R-free**	1081 (137)	1034 (136)	1577 (132)
**R-work**	0.1917 (0.2723)	0.1848 (0.3080)	0.1821 (0.3134)
**R-free**	0.2393 (0.3502)	0.2284 (0.4256)	0.2218 (0.3486)
**Number of non-hydrogen atoms**	4597	4554	4571
**macromolecules**	4245	4237	4232
**ligands**	240	247	234
**solvent**	112	70	105
**Protein residues**	527	526	527
**RMS(bonds)**	0.009	0.008	0.008
**RMS(angles)**	1.06	1.02	1.00
**Ramachandran favoured (%)**	93.52	94.47	95.81
**Ramachandran allowed (%)**	6.29	5.34	4.00
**Ramachandran outliers (%)**	0.19	0.19	0.19
**Rotamer outliers (%)**	1.97	1.53	1.75
**Clashscore**	7.95	7.37	4.68
**Average B-factor**	43.29	68.48	62.84
**macromolecules**	41.83	66.78	61.70
**ligands**	72.18	100.90	85.29
**solvent**	36.82	57.04	58.60

Table was calculated using Phenix. R-work=Σ |Fo - |Fc|| / Σ |Fo|, where Fo and Fc are observed and calculated structure factors, respectively. The R-free set uses about 5% randomly chosen reflections. Statistics for the highest-resolution shell are shown in parentheses.

Table S6. Data collection and refinement statistics for human p38α MAPK in complex with 94 and 95.

	**PDB 9D7N** (Cpd. **94**)	**PDB 9D75** (Cpd. **95**)
**Data collection**		
Space group	P 21 21 21	P 21 21 21
Wavelength (Å)	0.8731	0.8731
Unit cell parameters *a,b,c* (Å), angles (°)	66.392, 75.381, 79.241, 90°, 90°, 90°	65.906, 75.48, 78.923, 90°, 90°, 90°
Resolution (Å)	54.62 - 2.13	34.97 - 2.13
Outer Resolution Shell (Å)	(2.206 - 2.13)	(2.206 - 2.13)
Completeness (%)	75.79 (18.45)	99.95 (99.91)
R-merge	0.112 (3.188)	0.112 (1.707)
Mean I/σ(I)	14.12 (0.72)	14.36 (1.31)
Redundancy	14.0 (14.8)	13.3 (13.8)
No. of Unique Reflections	21792 (418)	22632 (2200)
**Refinement statistics**		
R-work	0.2024 (0.2708)	0.2061 (0.2994)
R-free	0.2515 (0.3348)	0.2360 (0.3230)
Residues	337	335
**Atoms**		
Protein	2757	2754
Ligand	87	94
Solvent	86	68
**Mean B-value (Å)**		
Overall	46.72	58.21
Protein	46.83	58.42
Ligand	47.53	55.02
Solvent	42.90	52.20
RMSD bond lengths (Å)	0.003	0.004
RMSD bond angles (°)	0.62	0.67
**Ramachandran plot, %**		
Most Favored	97.60	96.98
Allowed	2.10	2.72
Disallowed	0.30	0.30

Table was calculated using Phenix. R-work = $\Sigma\ |Fo - |Fc|| / \Sigma\ |Fo|$, Fo and Fc are observed and calculated structure factors, R-free set uses >1000 randomly chosen reflections. Statistics for the highest-resolution shell are shown in parentheses.

Table S7. Prediction of blood-brain barrier (BBB) penetration of compounds 94 and 95.

**Compound**	***Pe* ± SEM ($\times 10^{-6}$ cm/s)**	**CNS (+/–)**
**94**	10.4 ± 0.01	CNS+
**95**	13.2 ± 1.1	CNS+
Donepezil	*9.8 ± 0.4*	*CNS+*
Rivastigmine	*12.6 ± 2.9*	*CNS+*
Obidoxime	*0.34 ± 0.081*	*CNS–*
Cefuroxime	*0.31 ± 0.09*	*CNS–*

BBB penetration is expressed as effective permeability (*Pe*) value ± SEM (n = 2 for novel compounds, n = 3 for reference drugs) in cm/s. CNS+ indicates high predicted BBB permeation [Pe ($\times 10^{-6}$ cm/s) > 4.0], whereas CNS– indicates low predicted BBB permeation [Pe ($\times 10^{-6}$ cm/s) < 2.0].

Table S8. Metabolites of compounds 94 and 95 after a five-hour incubation with human liver microsomes.

Cpd number	Structure	[M+H]$^+$	Metab. 5 h/Parent 0 h [% area]
**94**	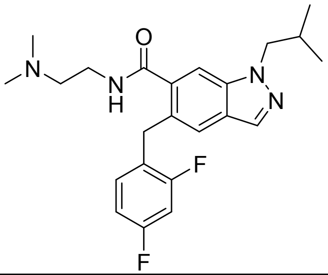	415	
/	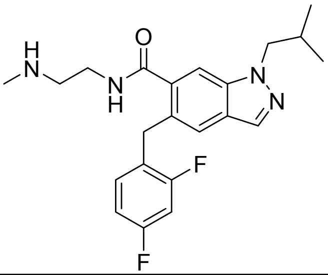	401	18.7 %
**Cpd number**	**Structure**	**[M+H]$^+$**	**Metab. 5 h/Parent 0 h [% area]**
**95**	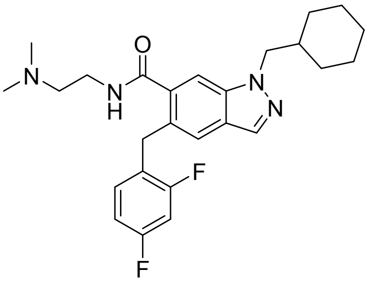	455	
/	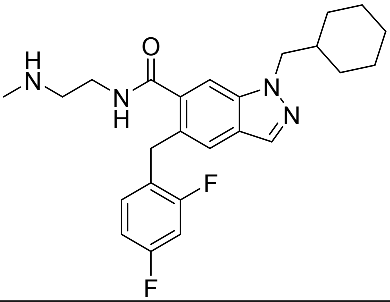	441	7.9 %

Percentages are defined as metabolite peak area compared to parent compound at the beginning of the incubation. Both compounds are subjected to mono *N*-demethylation (18.7% and 7.9%). No other significant metabolites were observed.

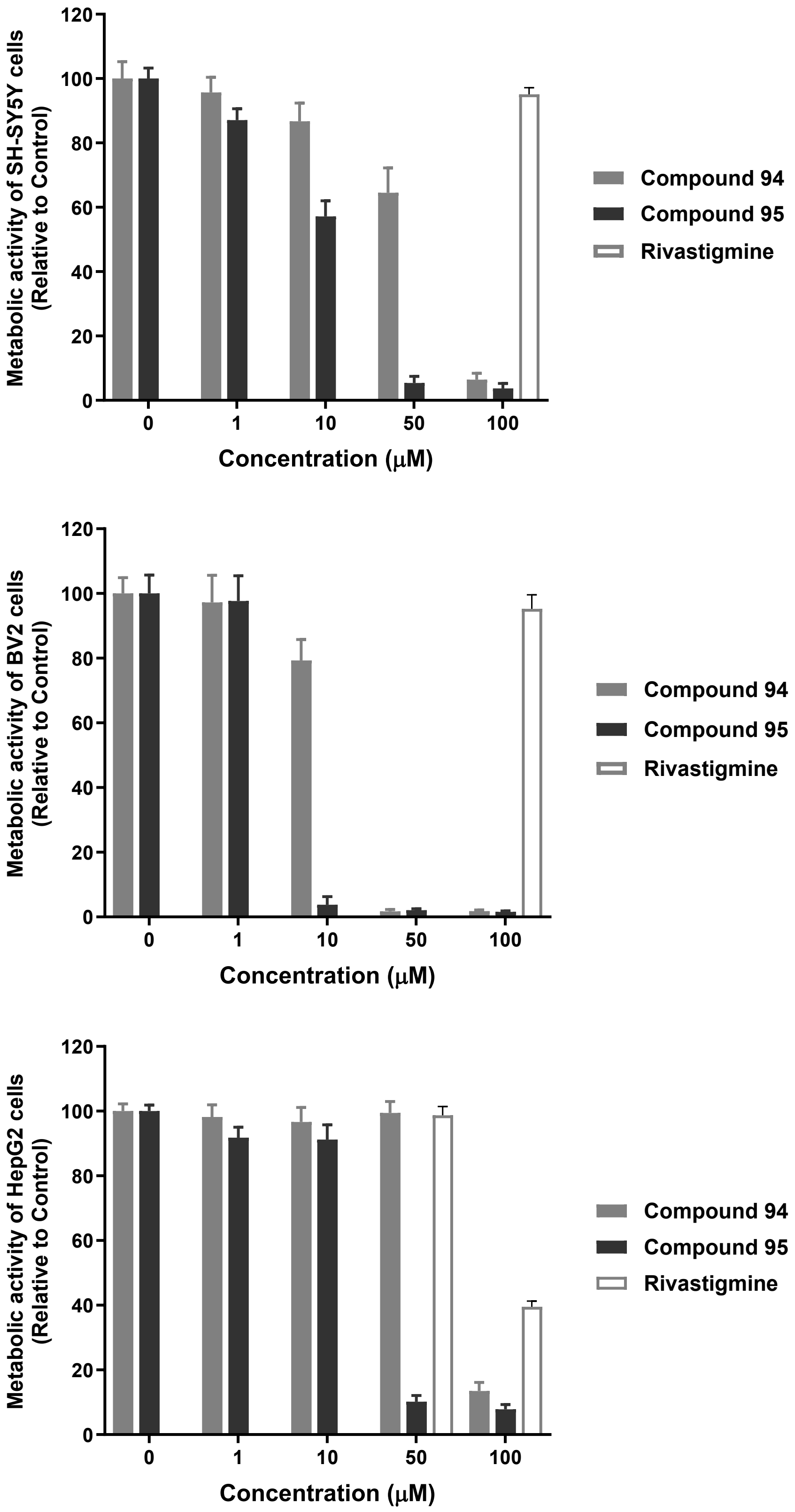

Figure S4. ***In vitro*** **cytotoxicity profiles of compounds 94, 95 and rivastigmine.** SH-SY5Y, BV2 and HepG2 cells were treated with the increasing concentrations of compounds **94** and **95** (1–100 μM) and rivastigmine (100 μM) in serum-free medium. After 24h, we evaluated the cell viability using the metabolic MTS assay. We

considered the control group (DMSO; 0 µM of compound) as 100% cell viability and present the data as means (± SEM) of at least three independent experiments, each carried out in quadruplicate.

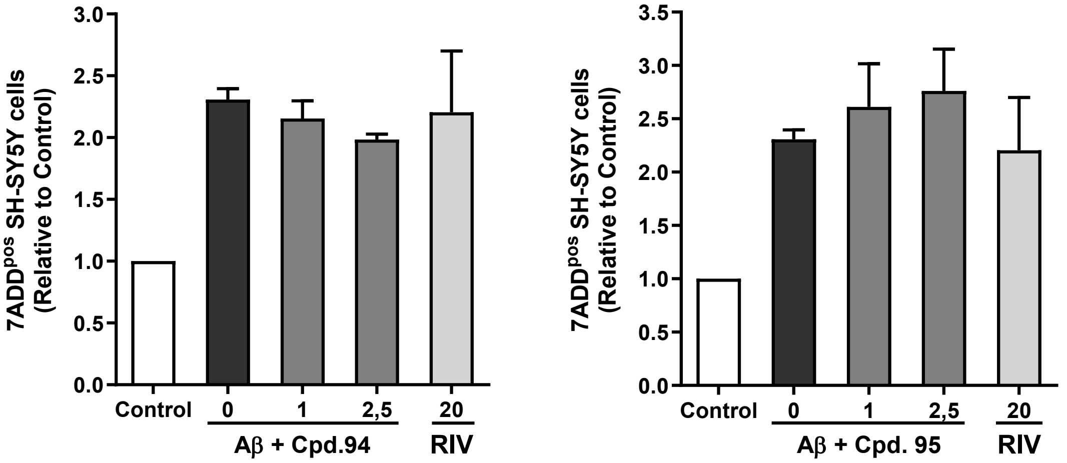

Figure S5. The impact of compounds 94 and 95 on A$\beta_{1\text{–}42}$-induced toxicity. SH-SY5Y cells were treated with pre-aggregated A$\beta_{1\text{–}42}$ (5 µM) in the absence or presence of compounds at concentrations 1 and 2.5 µM. After 48h treatment, neuroprotective effect was evaluated by flow cytometry analysis of 7AAD staining. The graph shows the results of quantitative analysis and indicates the percentage of dead cells, a fraction of 7AAD positive cells (7AAD^{pos}). DMSO treated cells were considered as control cells. Cells were treated in duplicate. The values are the mean ±SEM of two independent experiments.

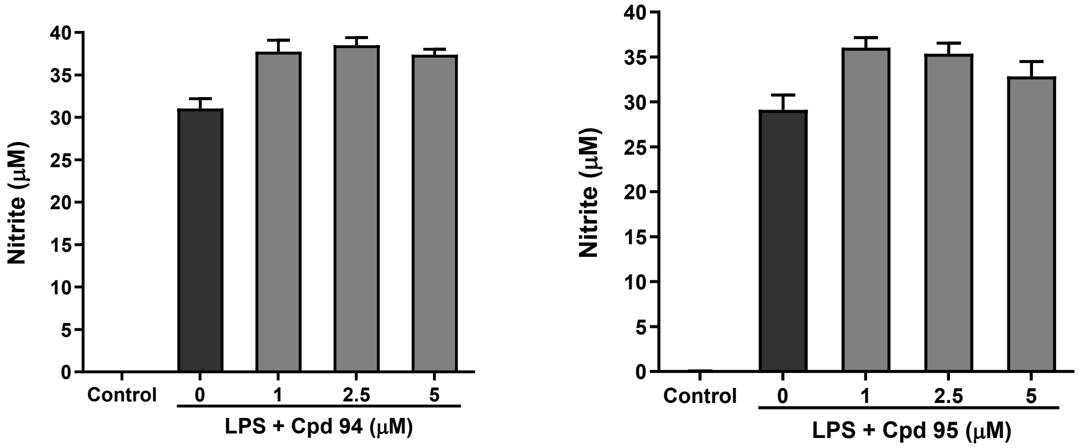

Figure S6. The impact of compounds 94 and 95 on LPS-induced microglial activation. BV2 cells were stimulated with LPS (1 µM) in the absence or presence of compounds at concentrations 1, 2.5 and 5 µM. After 24h stimulation, the supernatants of control and stimulated BV2 cells were collected and nitrite concentrations were determined using the Griess assay[4]. DMSO treated cells were considered as control cells. The values are the mean ±SEM of two independent experiments, each performed in duplicate.

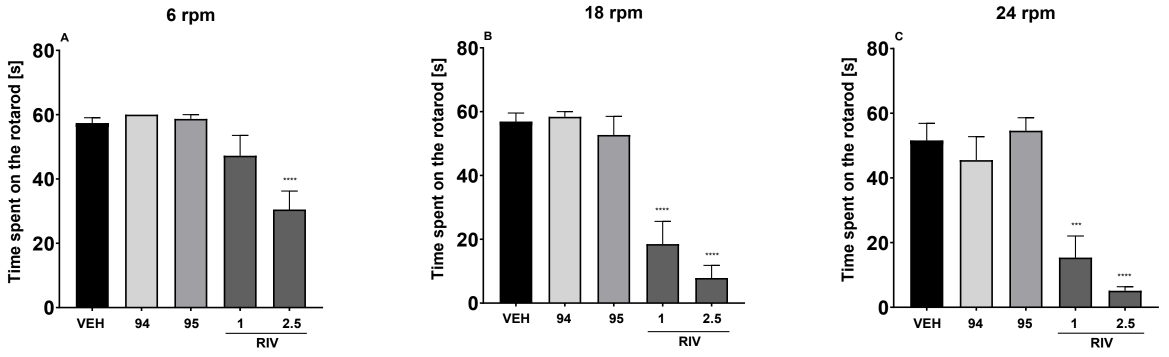

Figure S7. Effect of compounds 94 and 95 (10 mg/kg, i.p. injection) and rivastigmine (1 and 2.5 mg/kg, i.p. injection) on motor coordination measured using the rotarod test at 6 rotations per minute (rpm) (A), 18 rpm (B) and 24 rpm (C). Results for n = 8-10 are shown as the mean time spent on the rotarod apparatus ± SEM and were measured during the 1-min (for each speed) test. Statistical analysis: one-way ANOVA followed by Dunnett's post hoc comparison. Significance vs. control: *** p < 0.001, **** p < 0.0001.

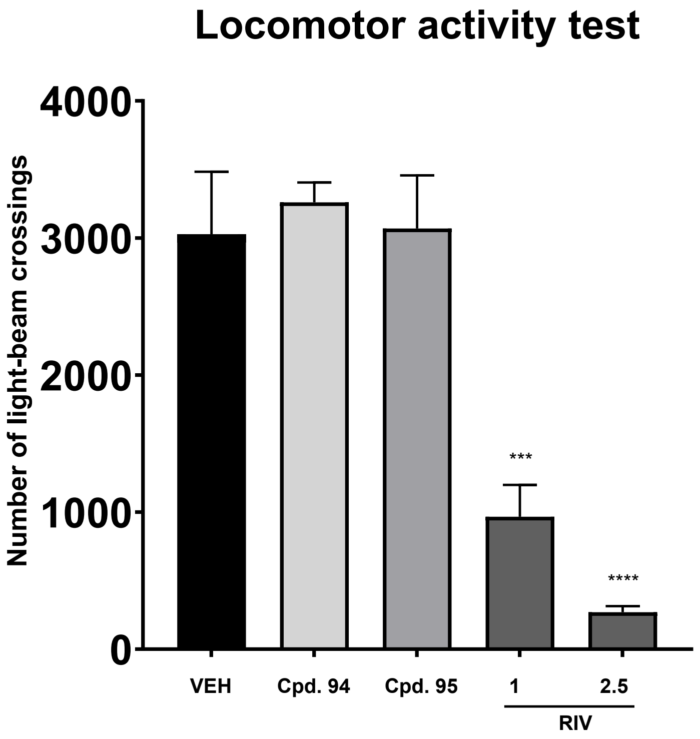

Figure S8. Effect of compounds 94 and 95 (10 mg/kg, i.p. injection) on locomotor activity measured in mice. Results are shown as the mean number of light-beam crossings ± SEM measured during the 60 min test for n = 8-10. Statistical analysis: one-way ANOVA followed by Dunnett's *post hoc* comparison. Significance vs. control: *** p < 0.001, **** p < 0.0001.

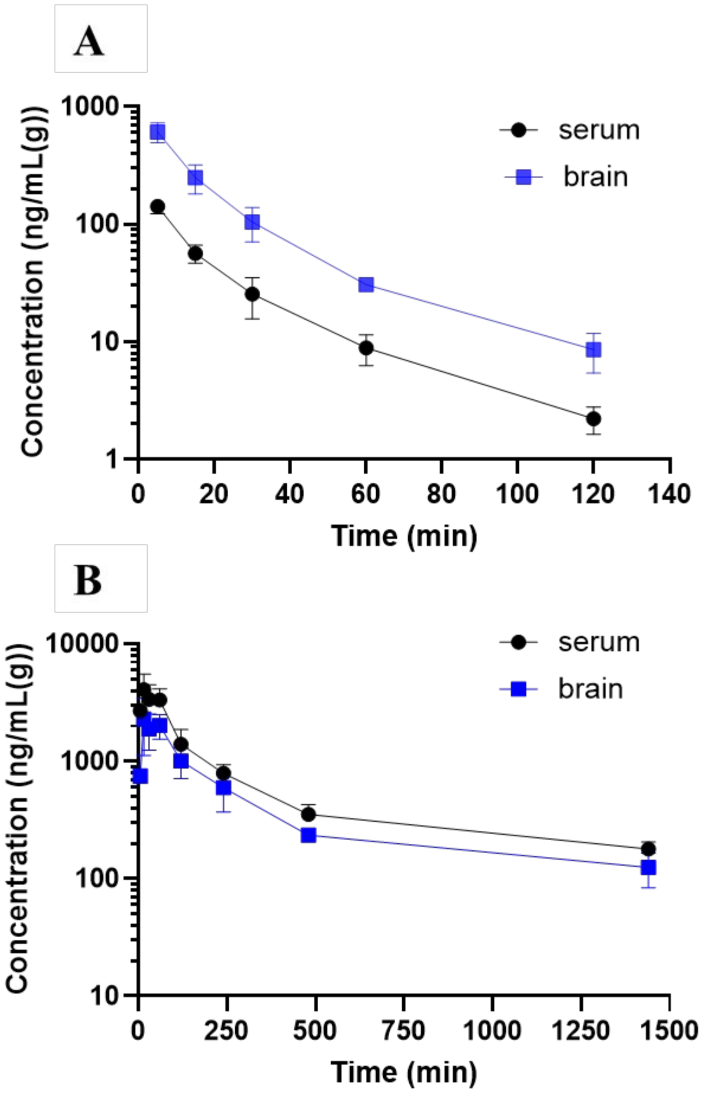

Figure S9. Mean (±SD) serum and brain concentrations of rivastigmine (A) and neflamapimod (B). Data was obtained following i.p. administration of a dose of 2.5 and 30 mg/kg, respectively, to mice (n=4).

Table S9. Pharmacokinetic parameters of rivastigmine and neflamapimod estimated using the model independent approach in serum and brain following i.p. administration of a dose of 2.5 and 30 mg/kg, respectively, to mice.

Pharmacokinetic parameter (unit)	rivastigmine		neflamapimod	
	serum	brain	serum	brain
t_{max} (min)	5	5	15	15
C_{max} (ng/mL(g))	142.00	609.33	4090.00	2293.33
λ_z [1/min]	0.023	0.021	0.00071	0.00066
$t_{0.5\lambda z}$ [min]	29.99	32.64	978.45	1049.79
AUC_{0-t} [ng·min/mL(g)]	2810.18	11678.40	865780.00	566796.70
$AUC_{0-\infty}$ (ng·min/mL(g)]	2906.24	12082.71	1119399.00	755355.60
V_z/F [L/kg]	37.22	-	37.83	-
CL/F [L/min/kg]	0.860	-	0.027	-
MRT [min]	27.79	26.61	903.28	999.01

Abbreviations: C_{max} - maximum concentration, t_{max} - time to reach maximum concentration, λ_z - terminal elimination rate constant, $t_{0.5\lambda z}$ - terminal half-life, AUC_{0-t} - area under the serum concentration-time curve from the time of dosing to the last measured point, $AUC_{0-\infty}$ - area under the serum concentration-time curve extrapolated to infinity, CL/F – apparent serum clearance after extravascular administration, V_z/F - volume of distribution based on the terminal phase, MRT - mean residence time.

Table S10. Gradient of the mobile phase composition used for the determination of neflamapimod, rivastigmine, and compounds 94 and 95 in mouse serum and brain homogenates. Phase A – 0.1% formic acid in acetonitrile; Phase B – 0.1% formic acid in water.

Time [min]	Phase A [%]	Phase B [%]	Flow rate [µL/min]
0	5	95	400
1	5	95	400
3	90	10	400
5	90	10	400
5.1	5	95	400
10	5	95	400

Table S11. Monitored precursor/product ion transitions and optimal ion path parameters for neflamapimod, rivastigmine, and compounds 94 and 95 determined in mouse serum and brain homogenates.

Compound	Neflamapimod		Rivastigmine	Compound 94		Compound 95	
Precursor/product ion transitions	437/101	437/145	251/206	415/370	415/271	455/410	455/367
Declustering potential (DP)	121	121	1	101	101	121	121
Entrance potential (EP)	10	10	10	10	10	10	10
Collision cell exit potential (CXP)	8	10	6	12	10	26	6
Collision Energy (CE)	81	35	19	29	45	33	37

Figure S10. Representative chromatograms of serum calibration samples for rivastigmine (A), compound 94 (B), neflamapimod (C), and compound 95 (D) at a concentration of 10 ng/mL.

A

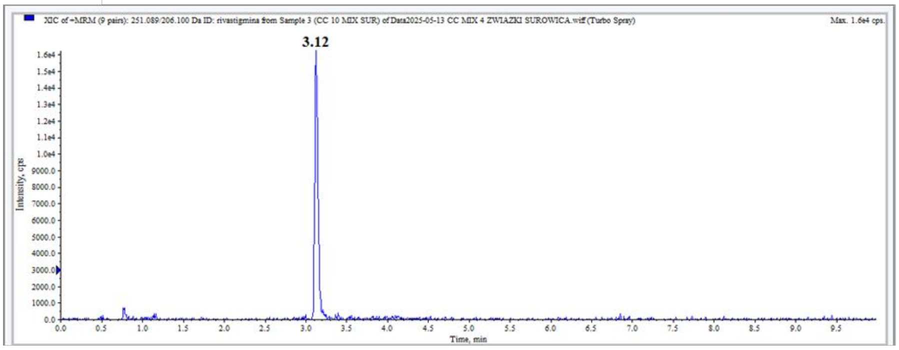

B

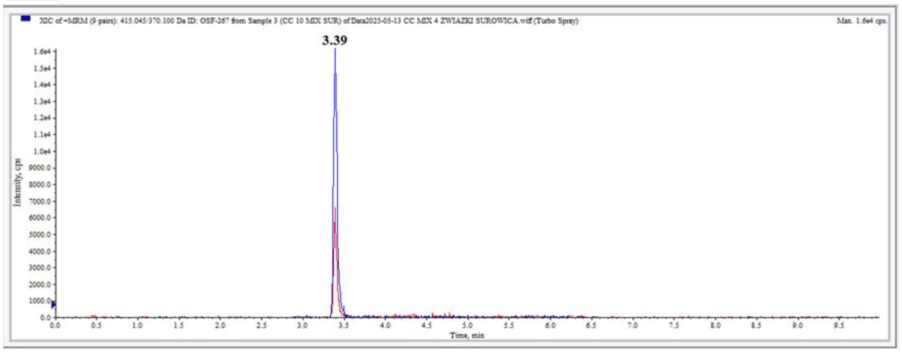

C

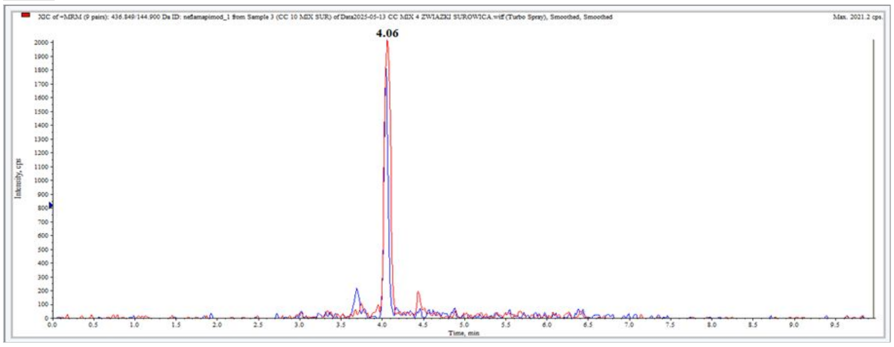

D

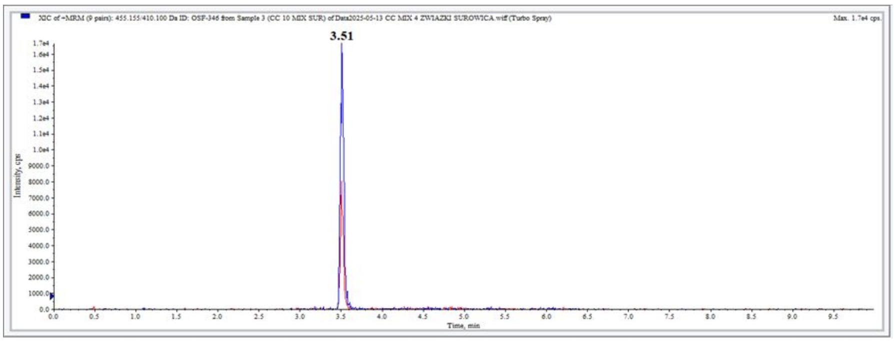

^{1}H and ^{13}C NMR spectra of final compounds

87

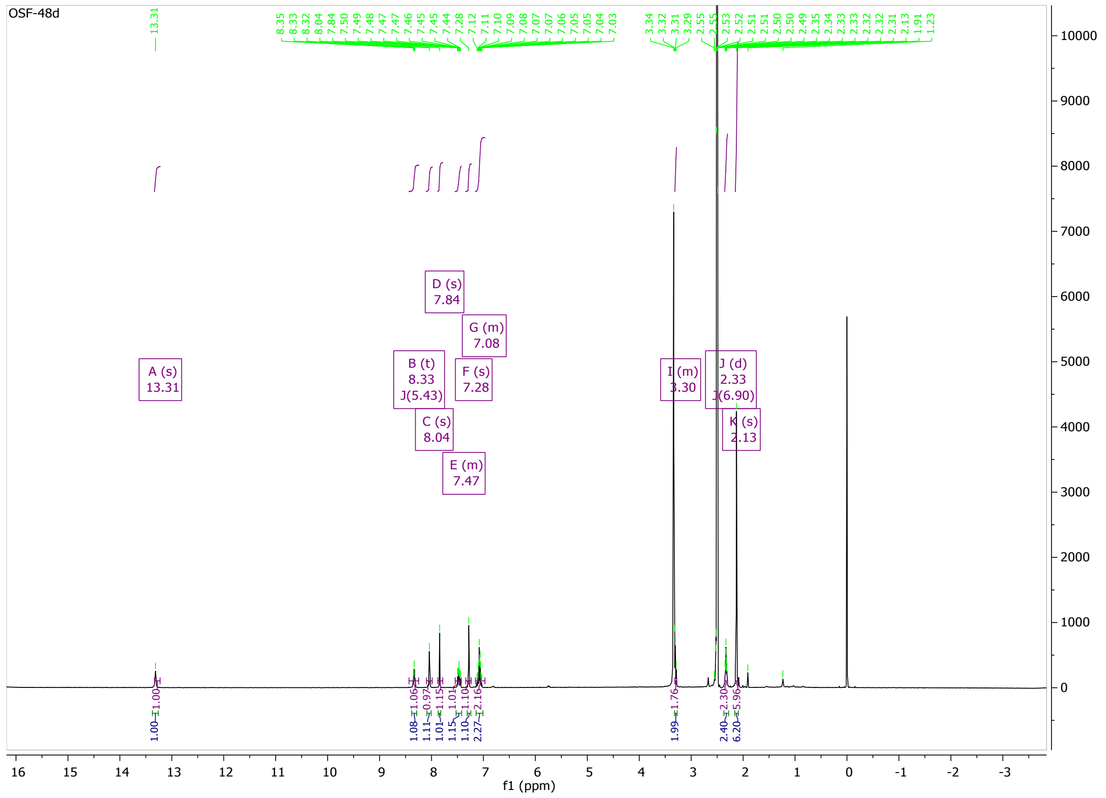

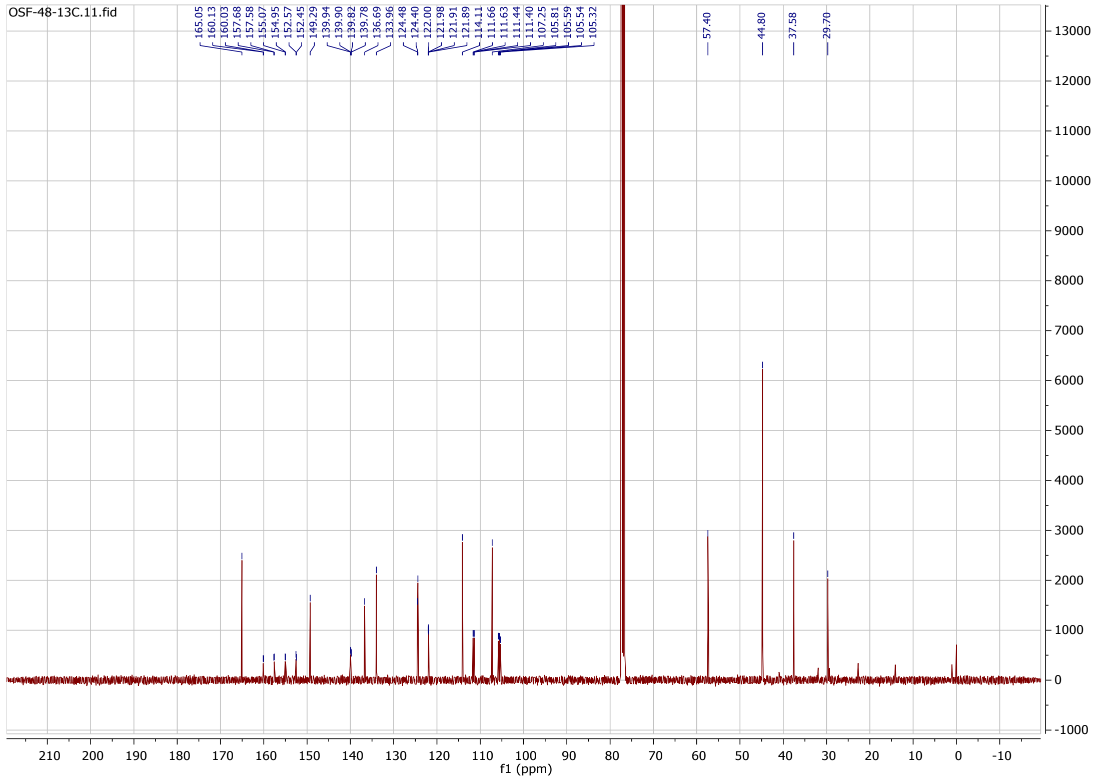

72

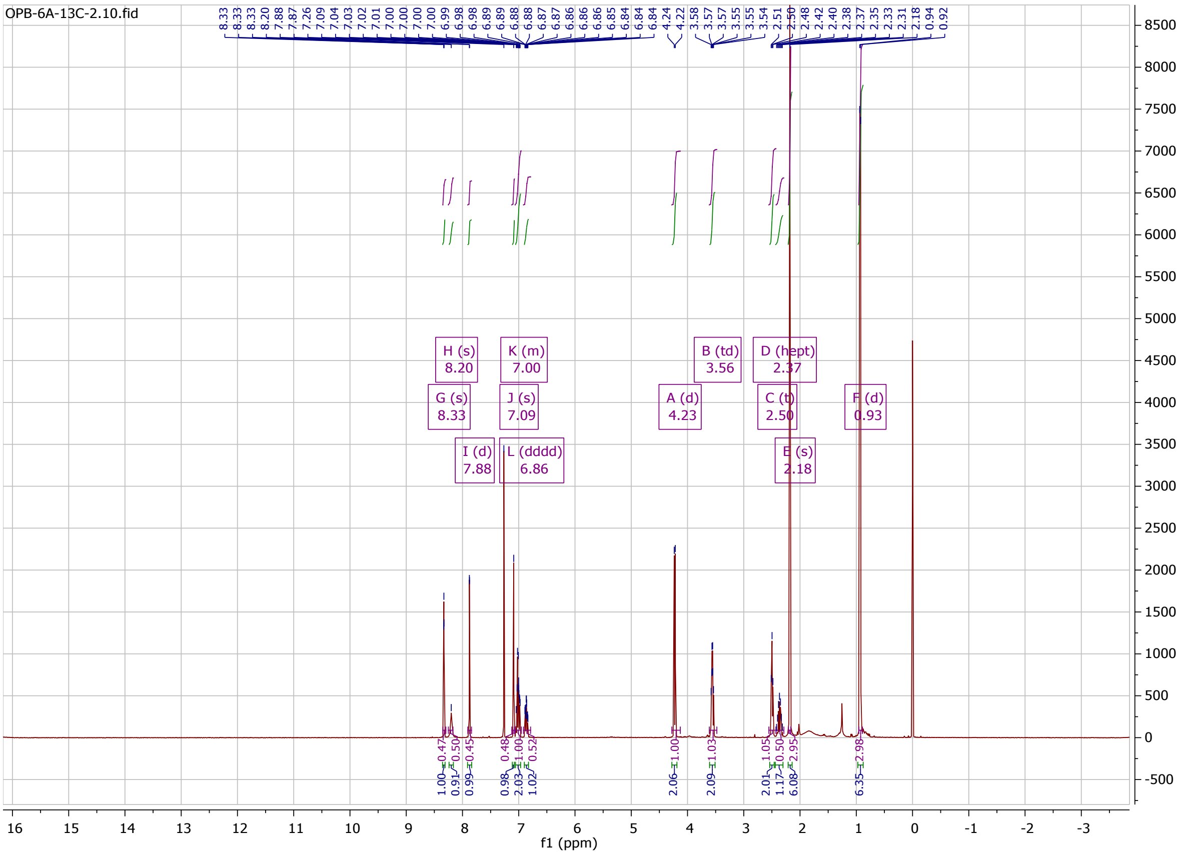

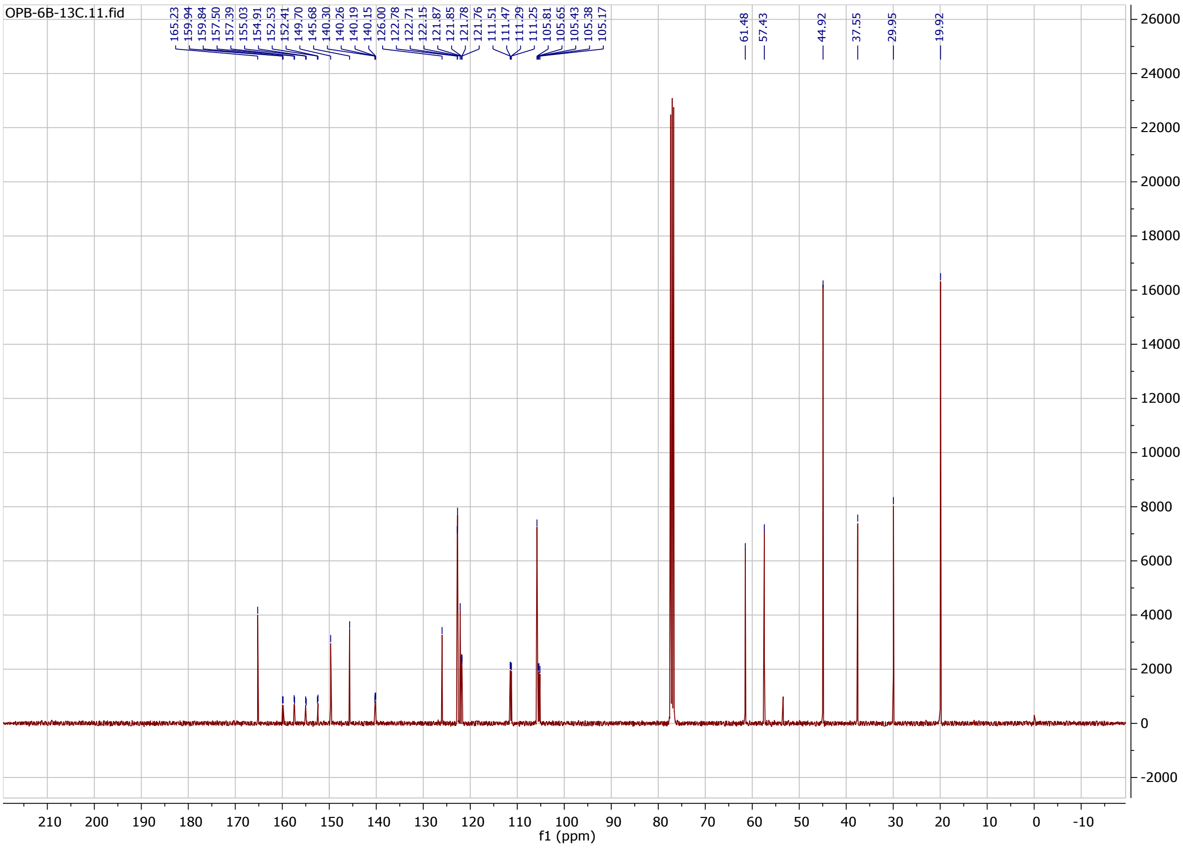

73

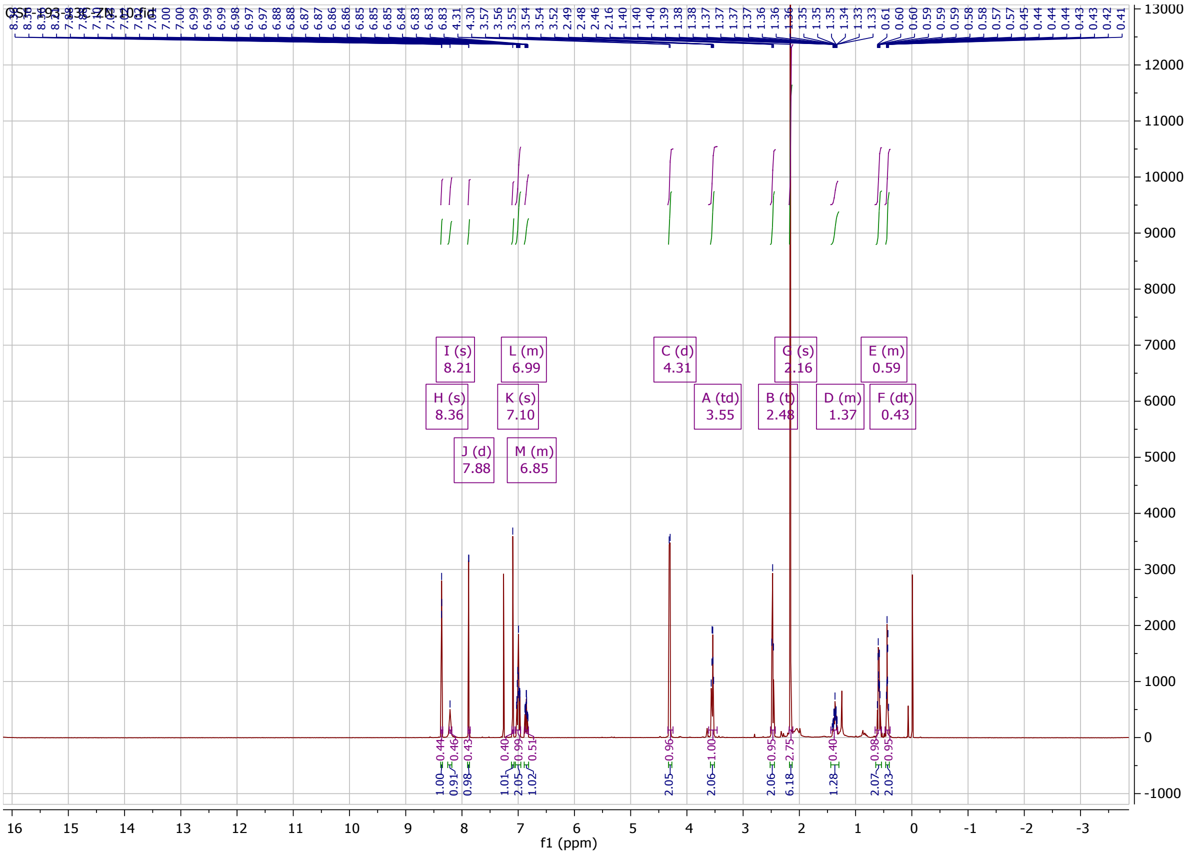

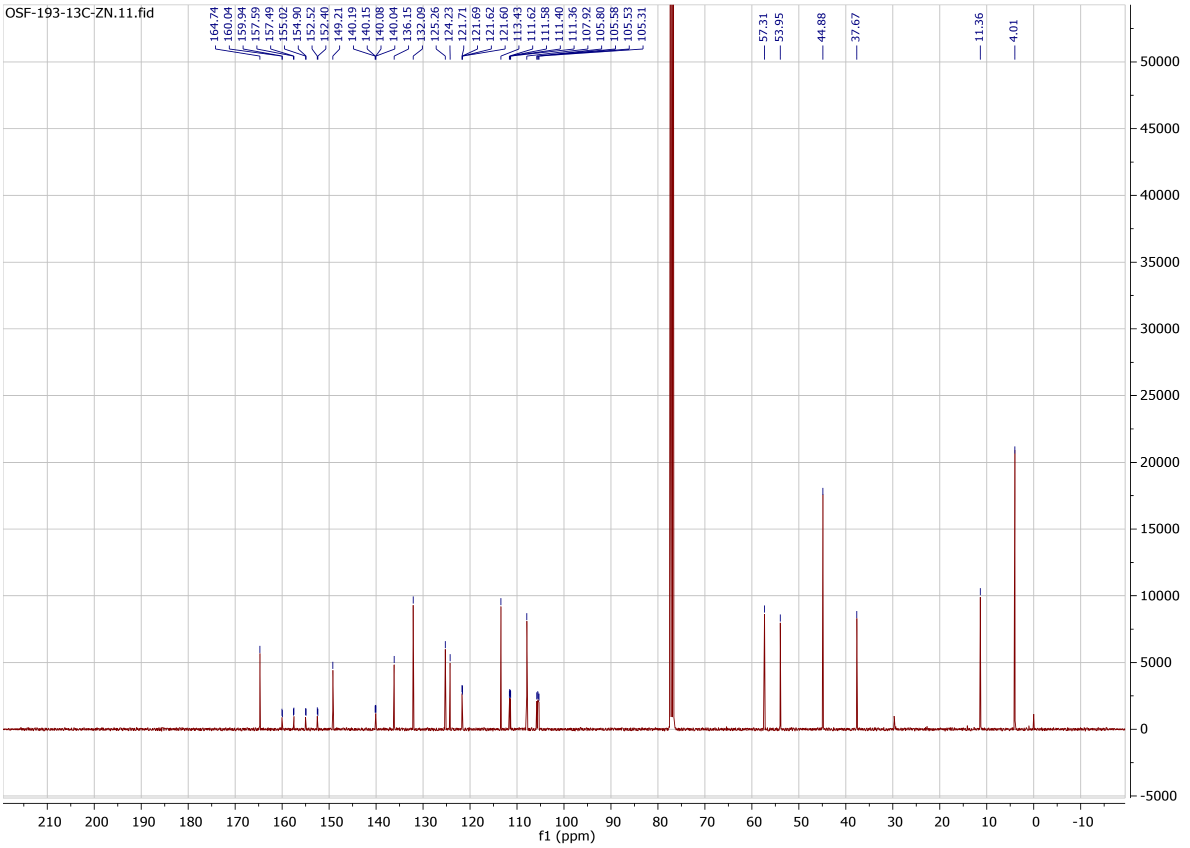

74

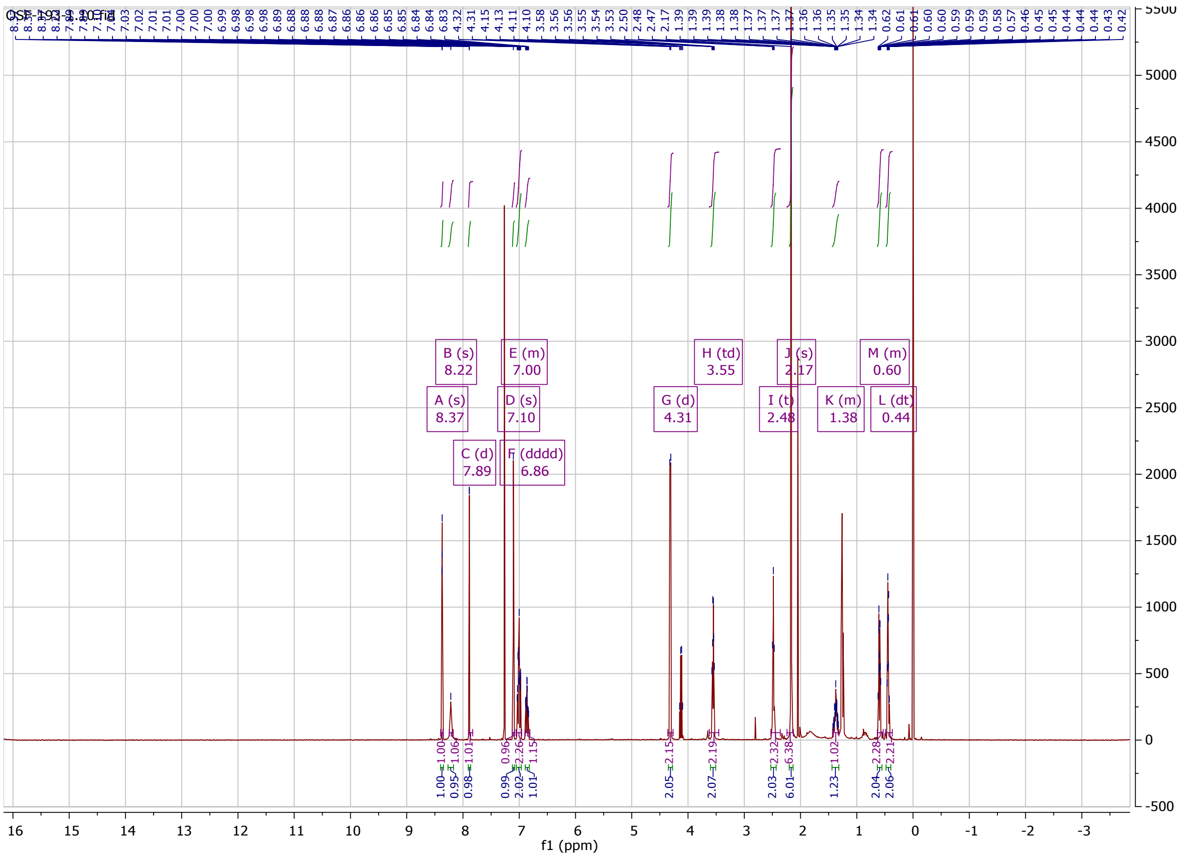

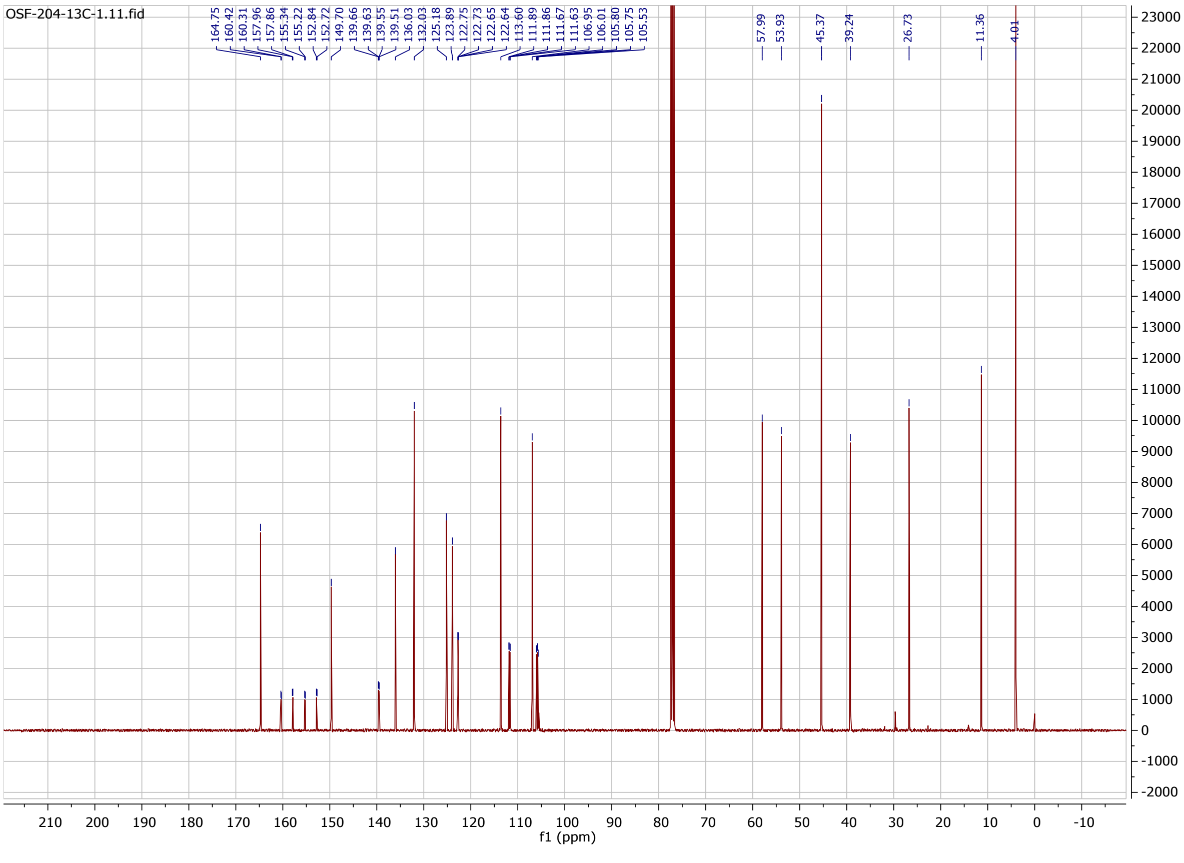

75

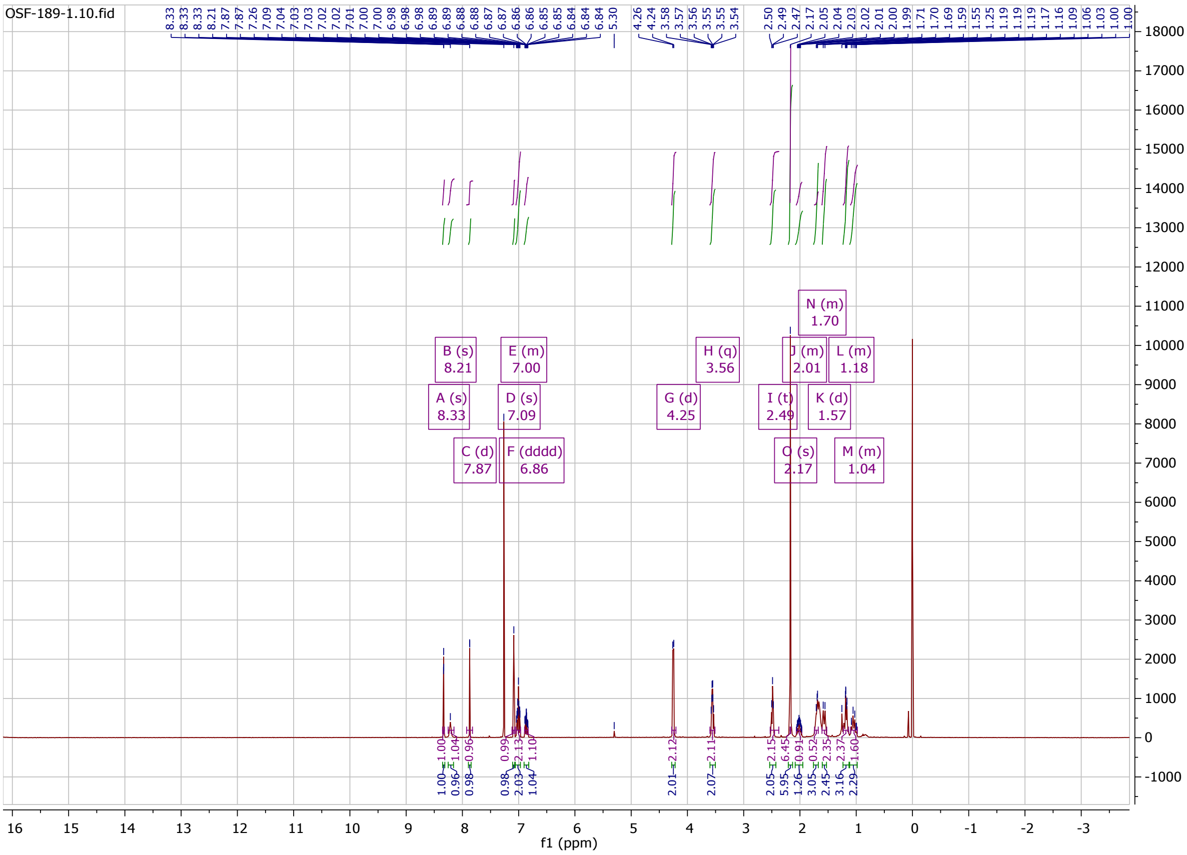

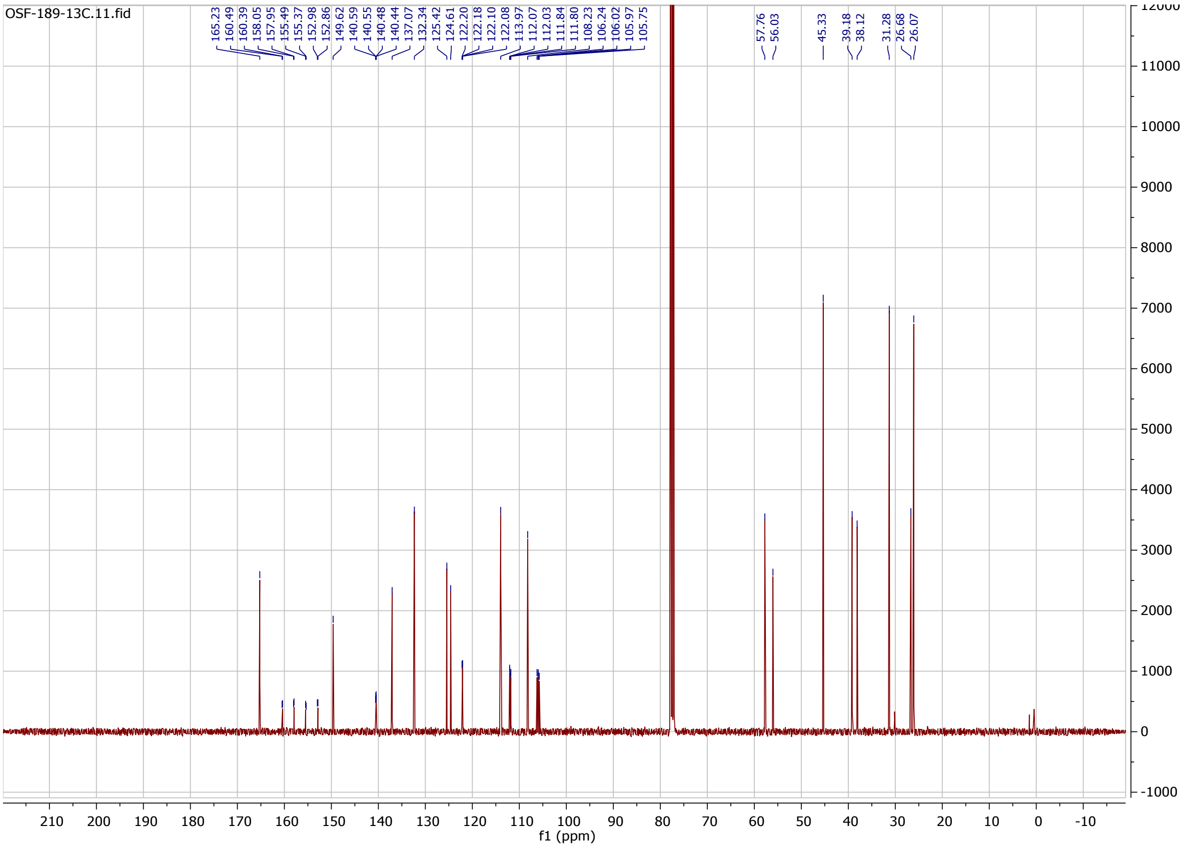

76

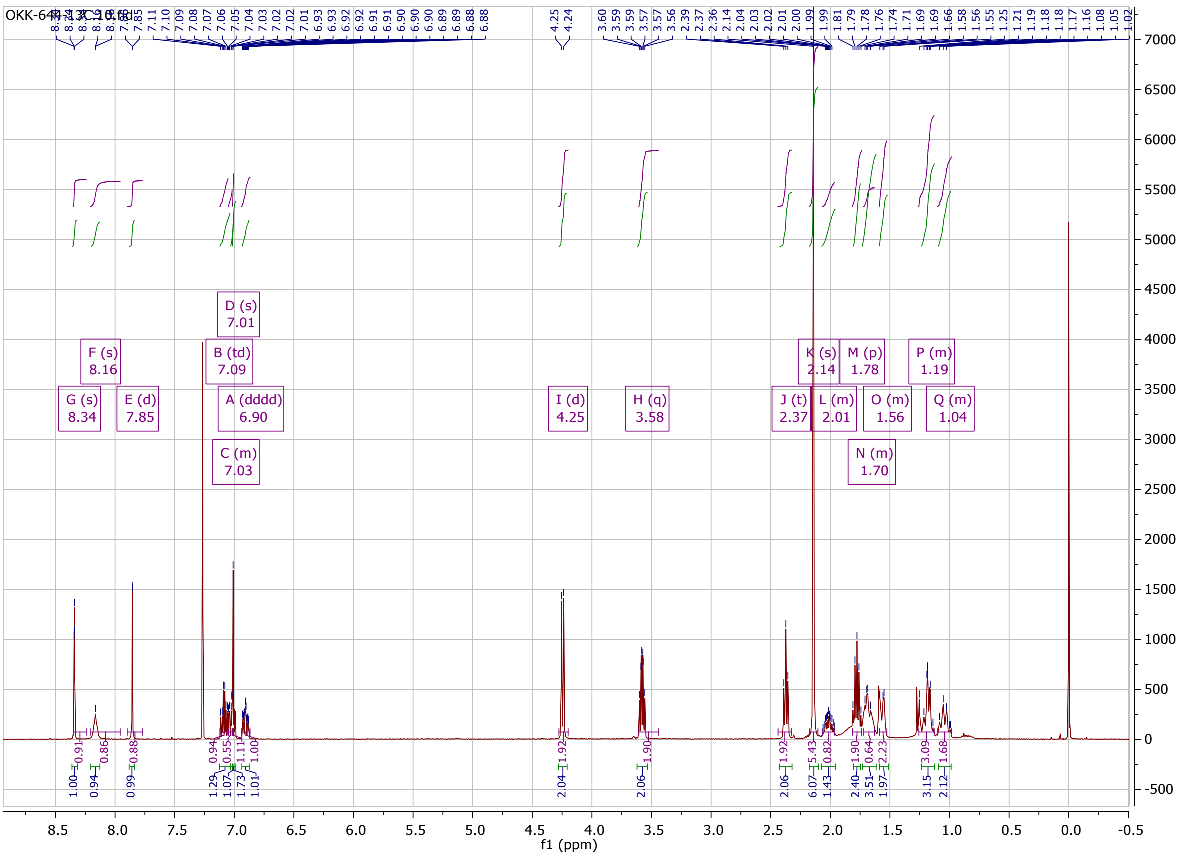

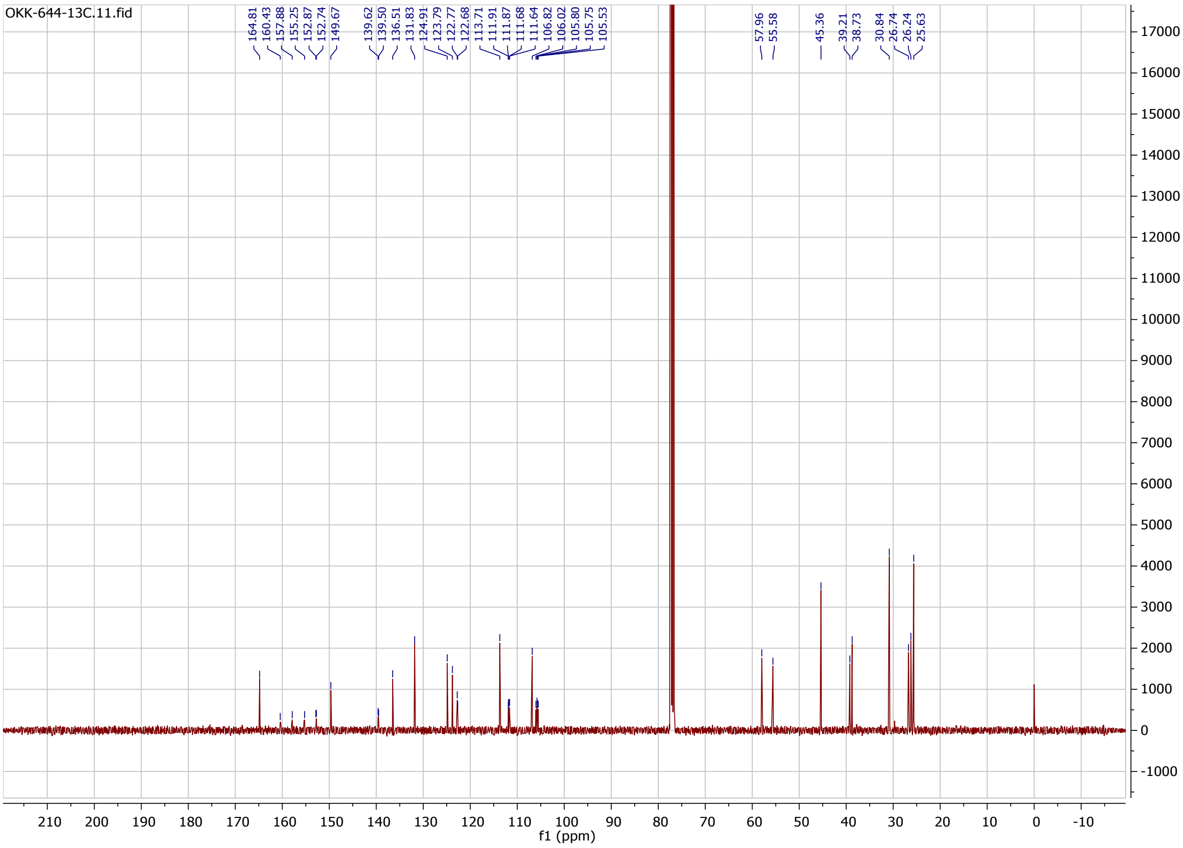

78

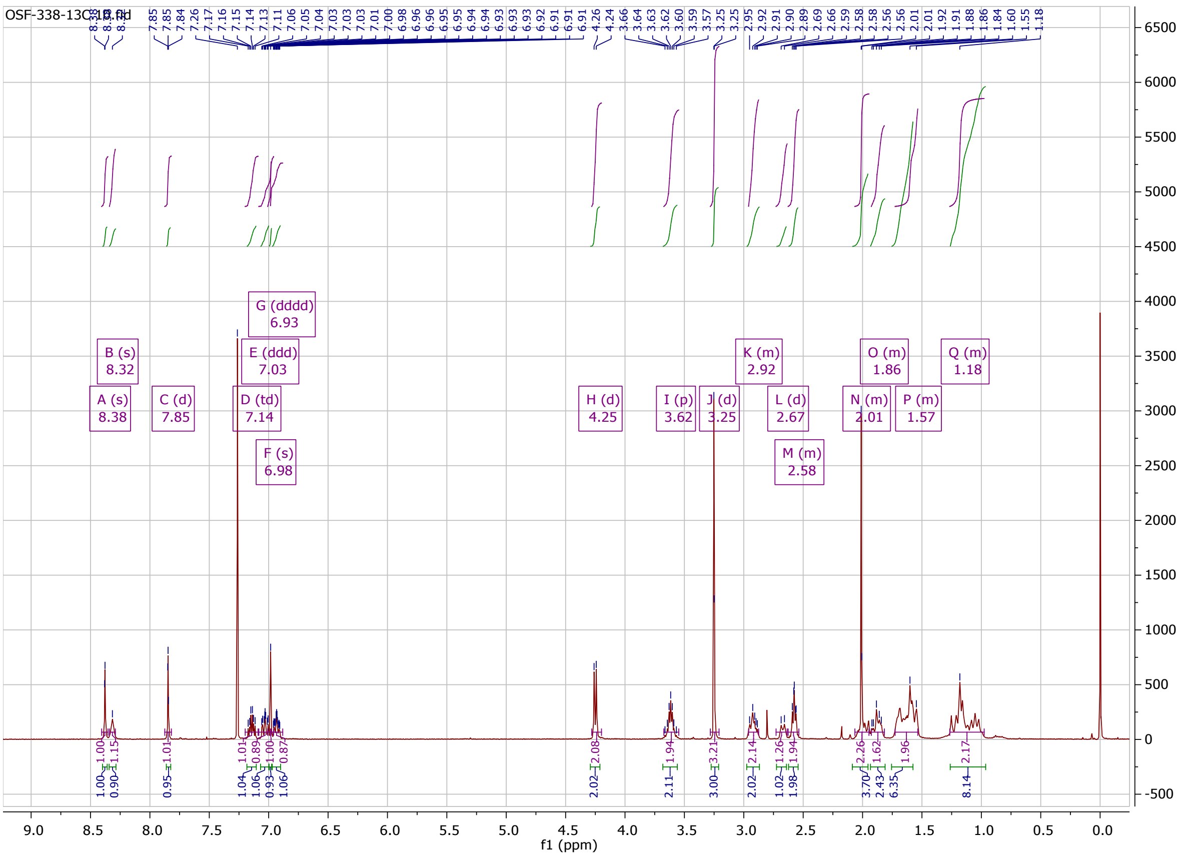

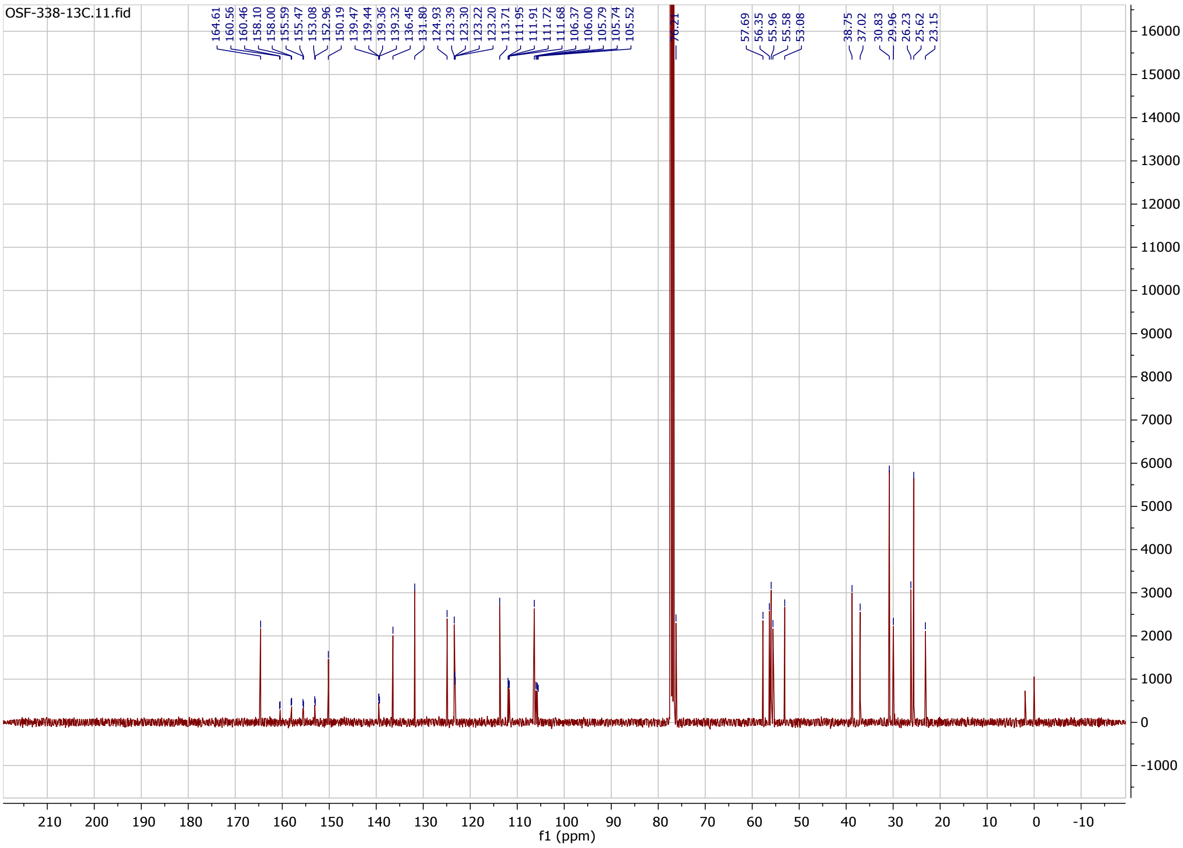

79

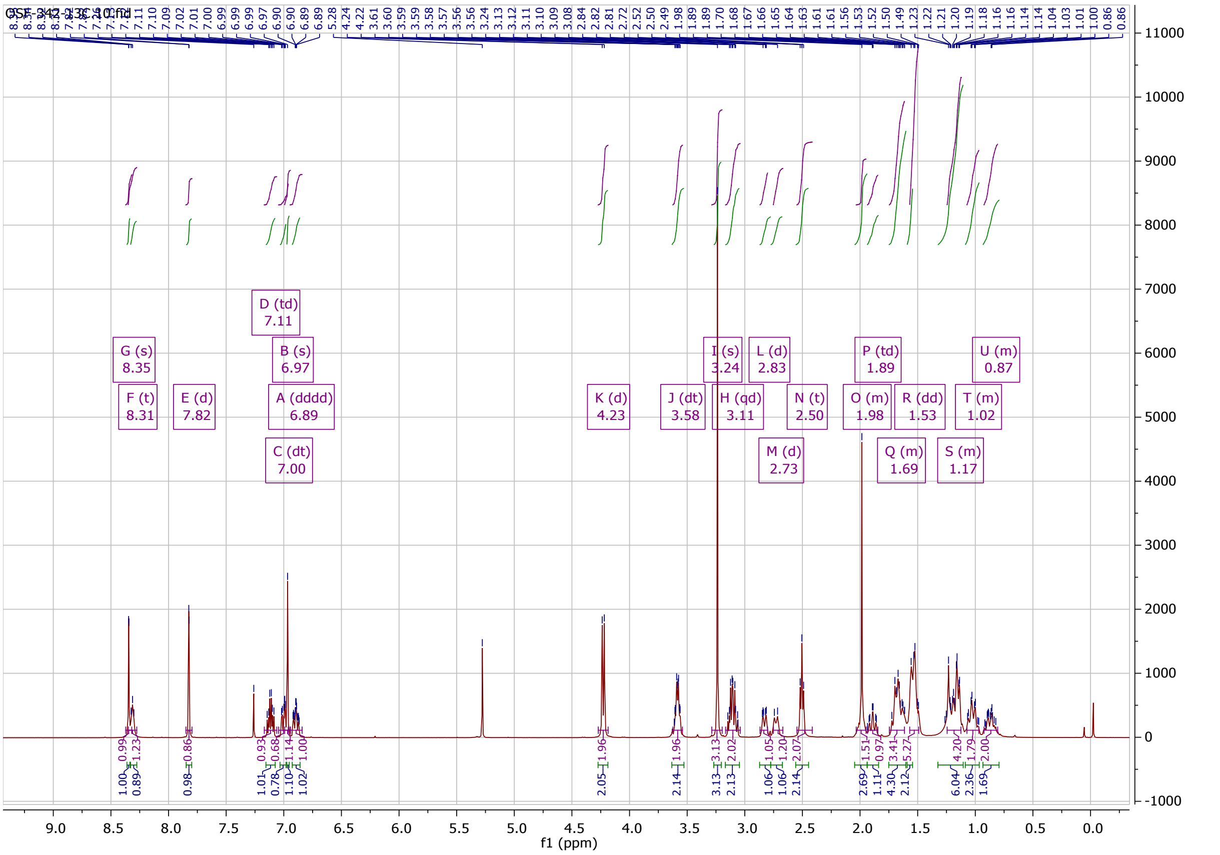

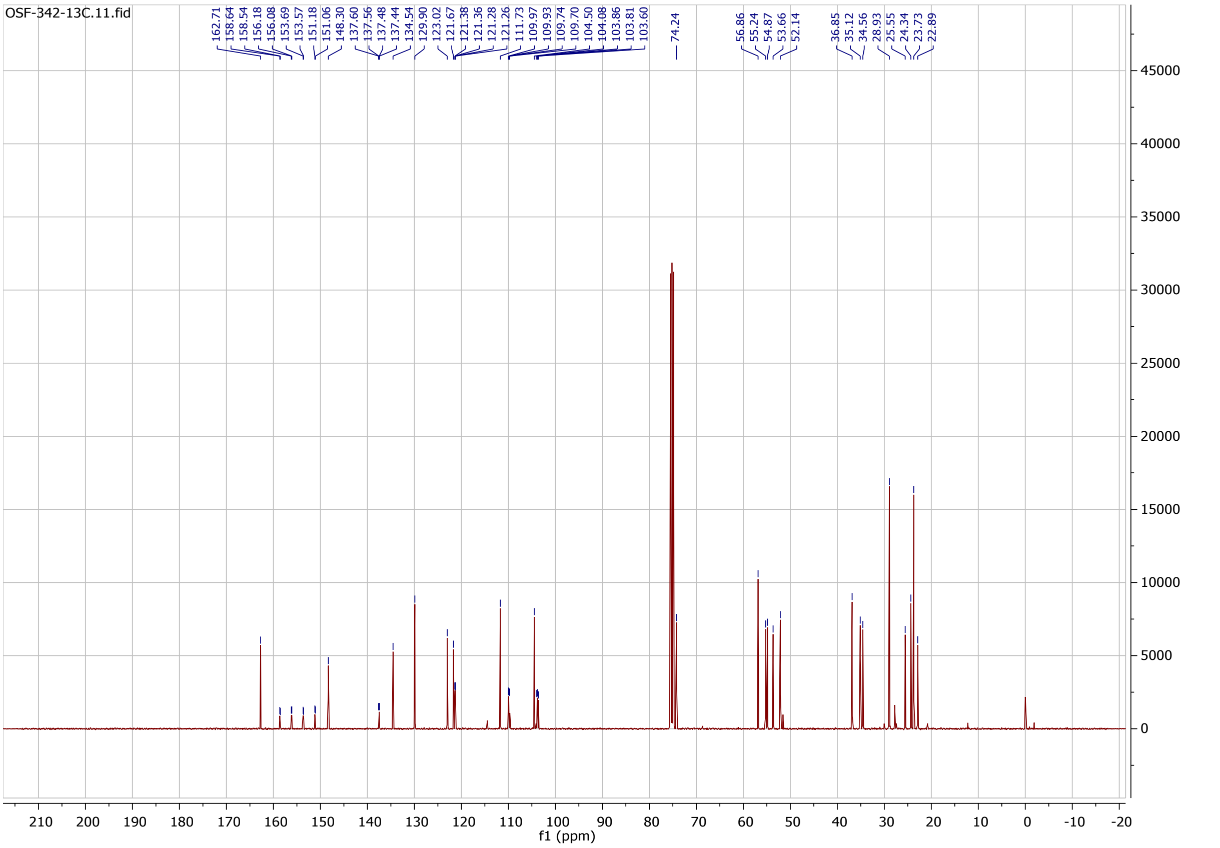

80

^{1}H NMR (400 MHz, $CDCl_3$) δ 8.40 – 8.36 (m, 2H), 7.85 (d, J = 1.0 Hz, 1H), 7.14 (td, J = 9.0, 5.4 Hz, 1H), 7.04 (ddd, J = 10.4, 8.2, 3.0 Hz, 1H), 7.00 (s, 1H), 6.97 – 6.90 (m, 1H), 4.25 (d, J = 7.3 Hz, 2H), 3.62 (d, J = 6.2 Hz, 2H), 3.52 – 3.42 (m, 4H), 2.57 (t, J = 6.0 Hz, 2H), 2.42 (t, J = 4.6 Hz, 4H), 2.06 – 1.94 (m, 1H), 1.75 – 1.63 (m, 4H), 1.63 – 1.49 (m, 2H), 1.24 – 1.12 (m, 2H), 1.10 – 0.97 (m, 2H).

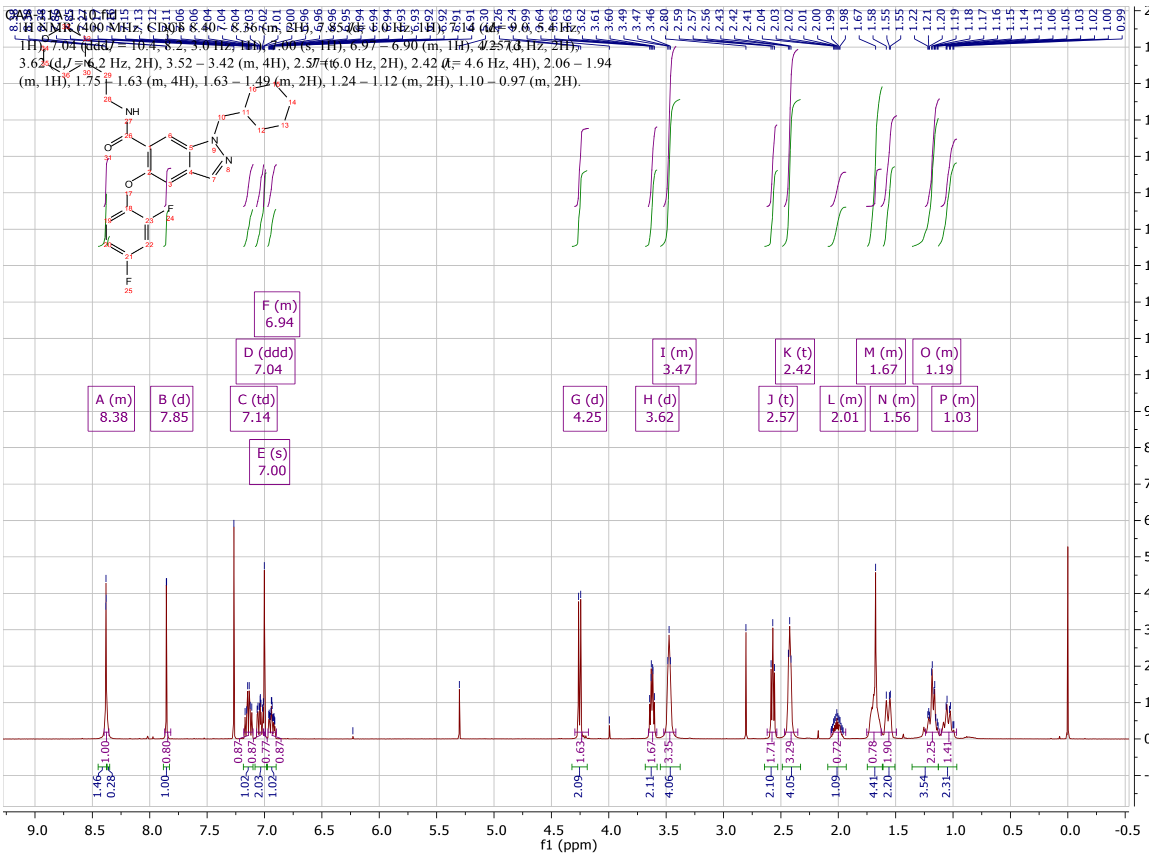

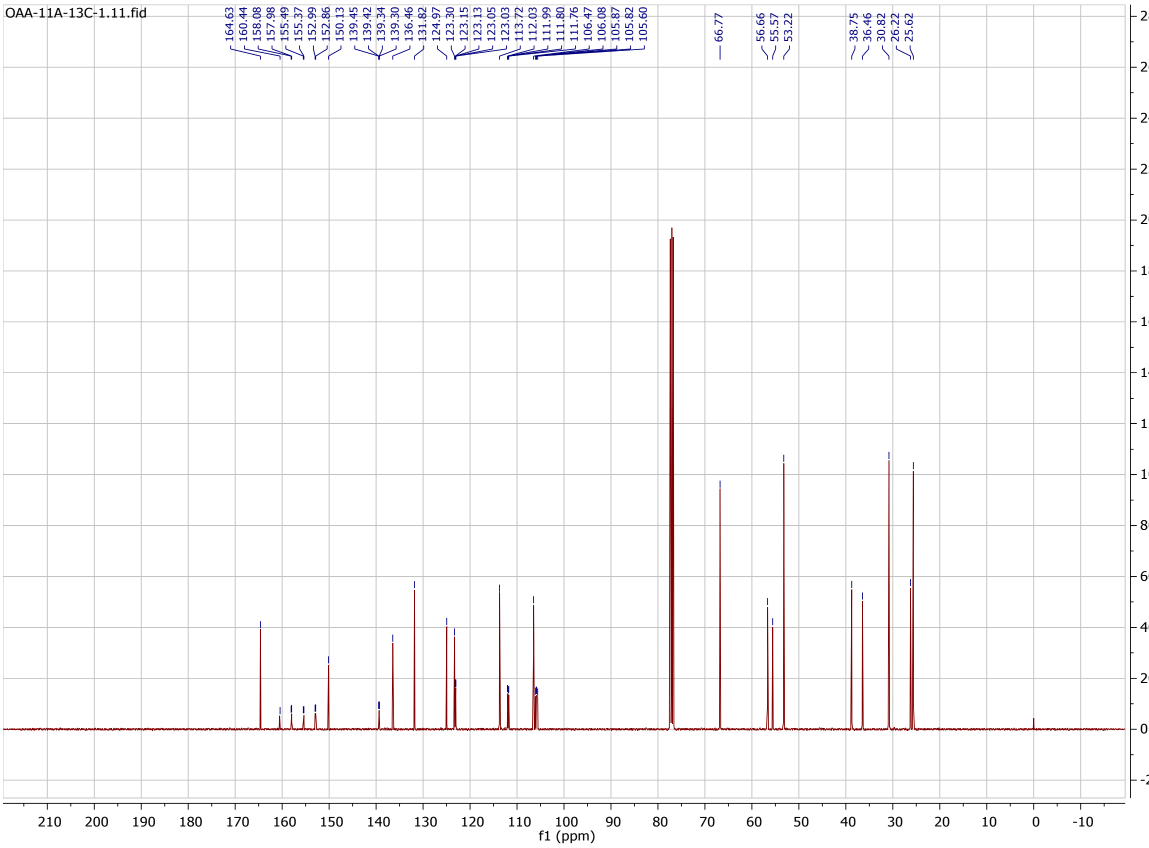

81

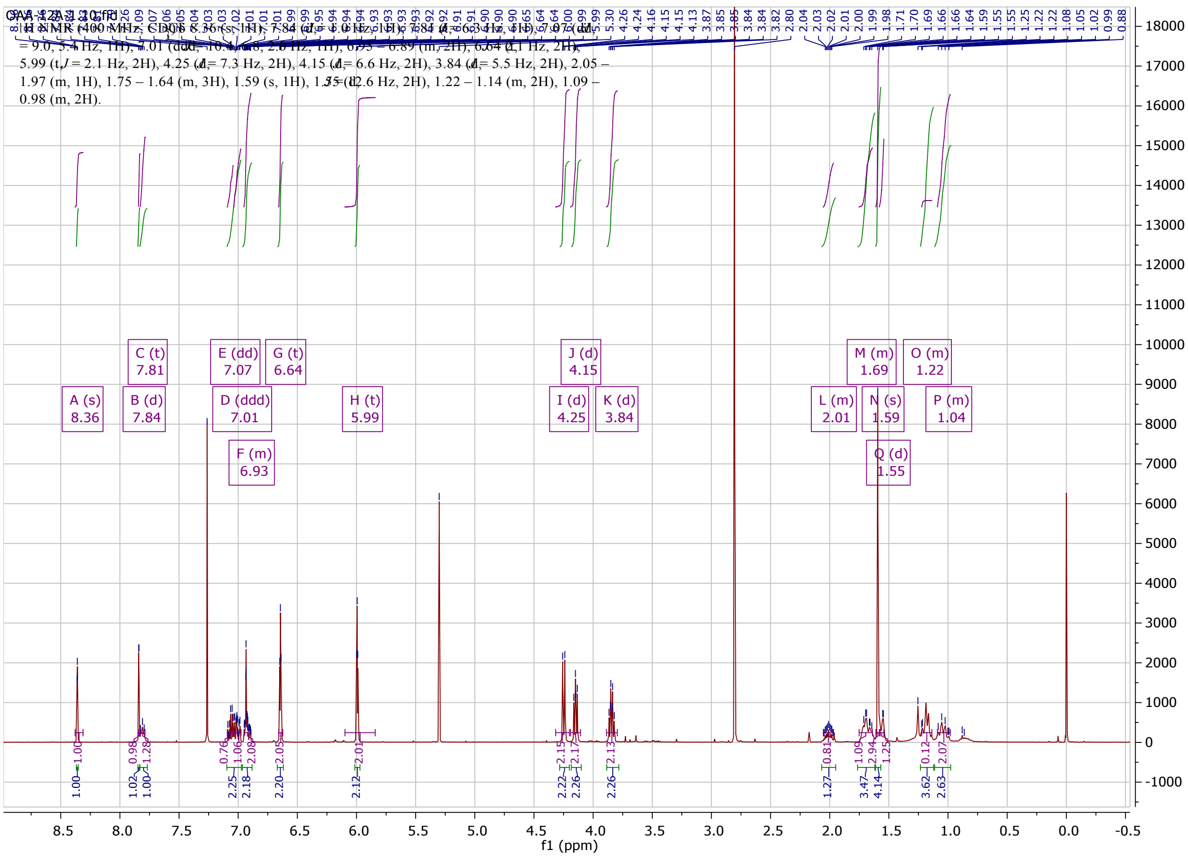

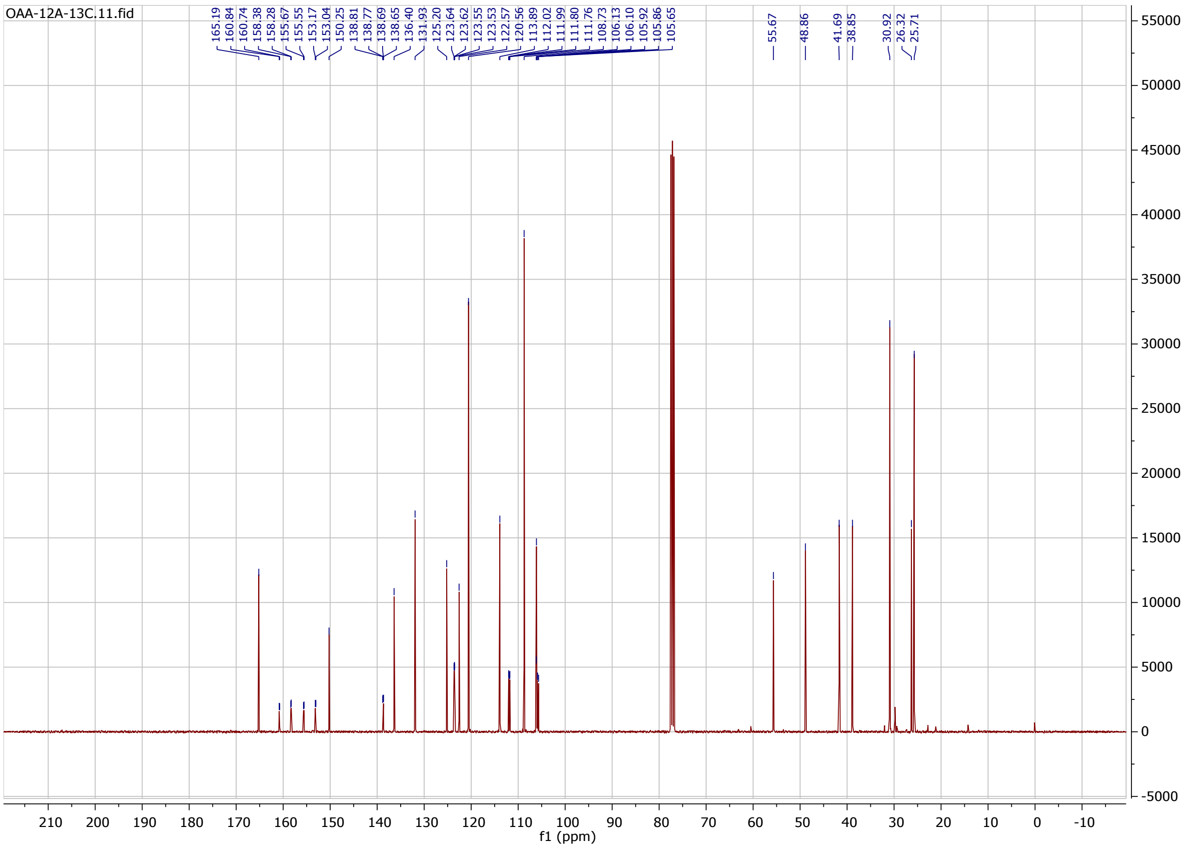

82

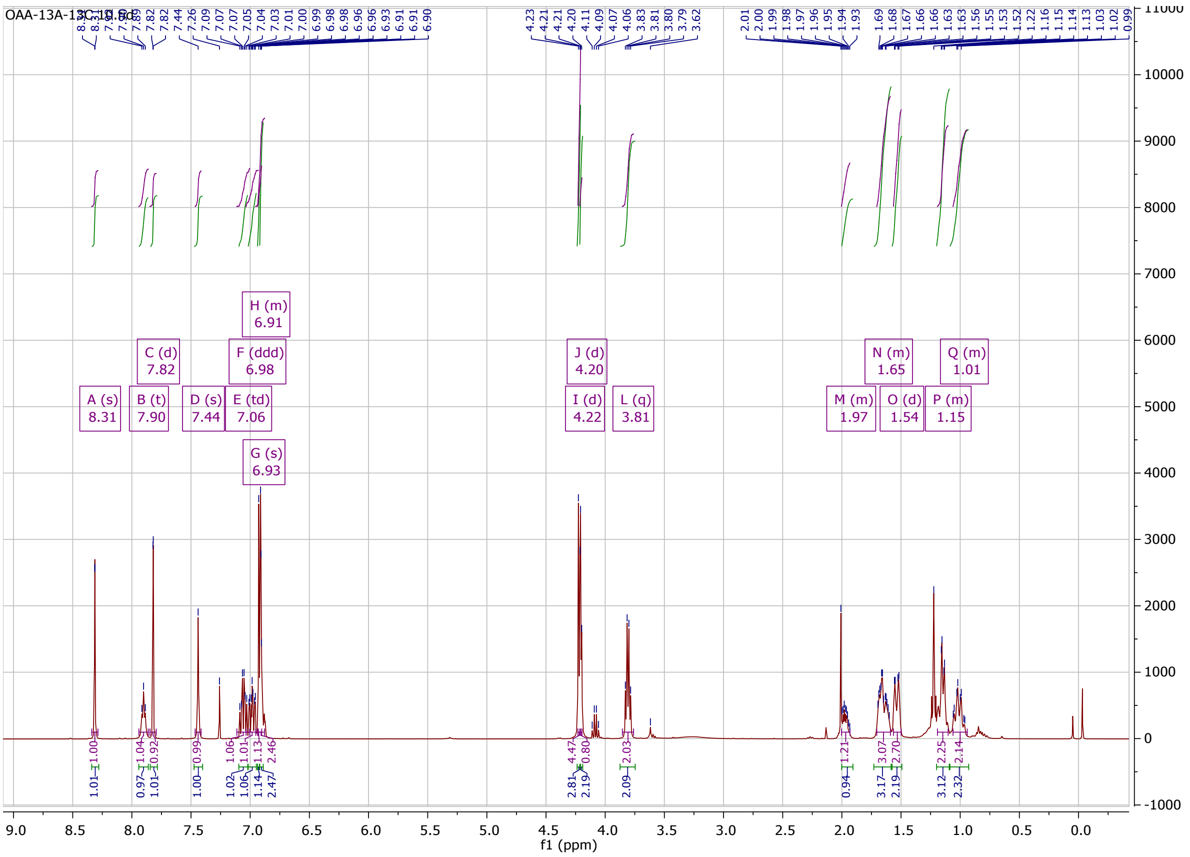
OAA-13A-13C.10.fid
A (s) 8.31
B (t) 7.90
C (d) 7.82
D (s) 7.44
E (td) 7.06
F (ddd) 6.98
G (s) 6.93
H (m) 6.91
I (d) 4.22
J (d) 4.20
L (q) 3.81
M (m) 1.97
N (m) 1.65
O (d) 1.54
P (m) 1.15
Q (m) 1.01
f1 (ppm)

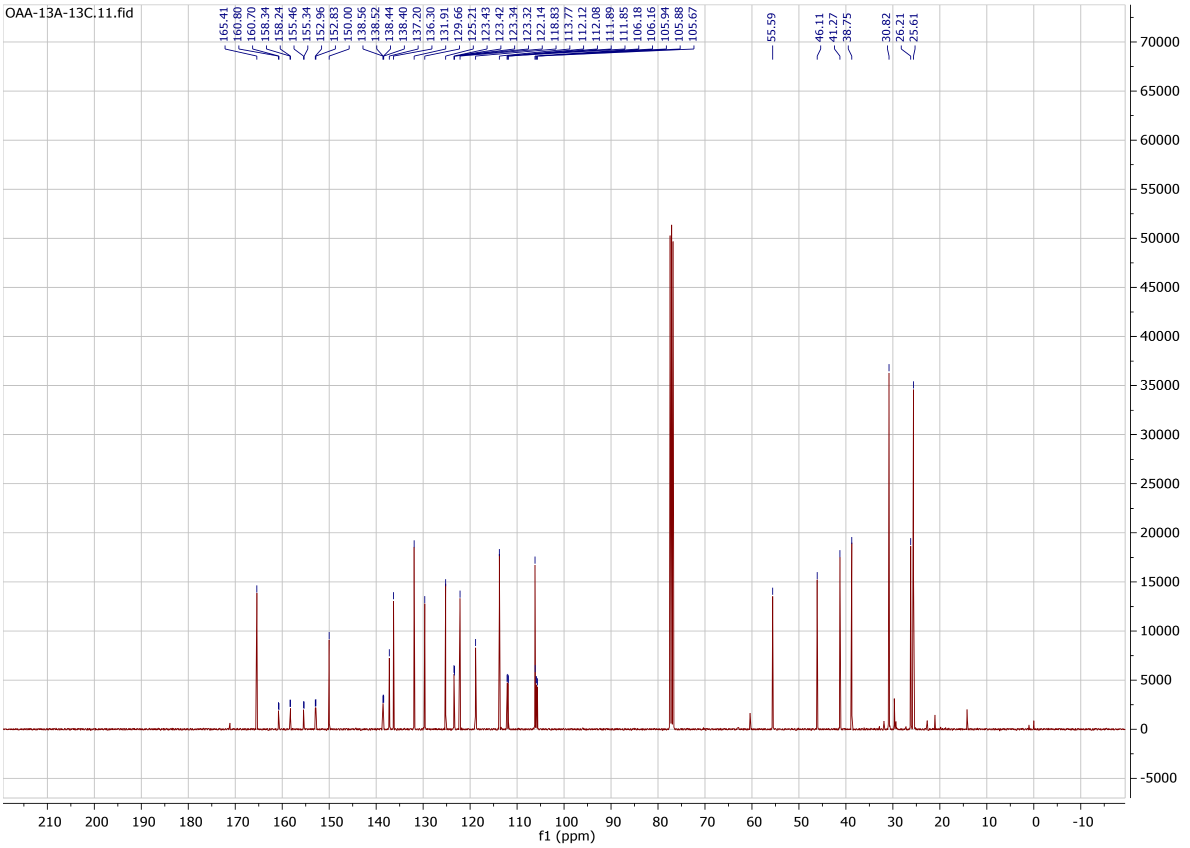
OAA-13A-13C.11.fid
165.41 160.80 160.70 158.34 158.24 155.46 155.34 152.96 152.83 150.00 138.56 138.52 138.44 138.40 137.20 136.30 131.91 129.66 125.21 123.42 123.34 123.32 122.14 118.83 113.77 112.12 112.08 111.89 111.85 106.18 106.16 105.94 105.88 105.67
55.59
46.11 41.27 38.75
30.82 26.21 25.61
f1 (ppm)

83

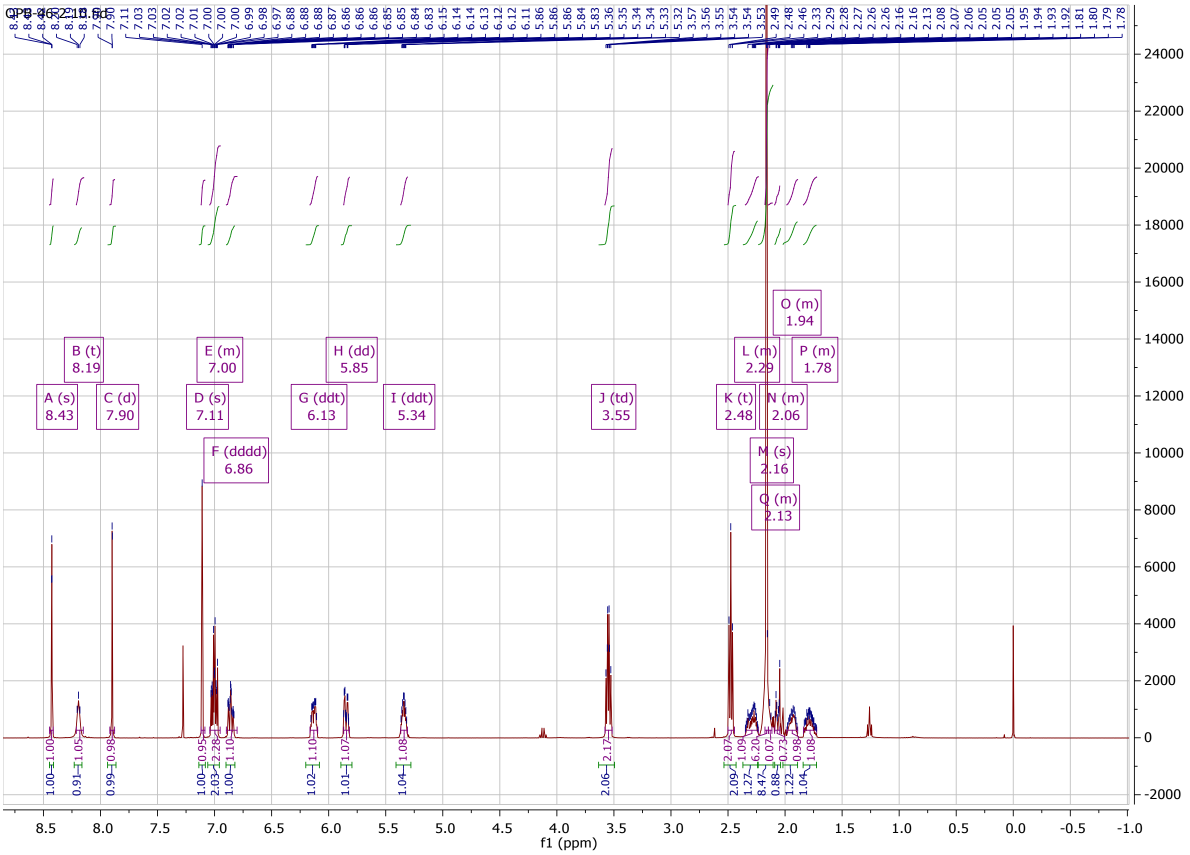
OPB-46-2 1H.fid
A (s)
8.43
B (t)
8.19
C (d)
7.90
D (s)
7.11
E (m)
7.00
F (dddd)
6.86
G (ddt)
6.13
H (dd)
5.85
I (ddt)
5.34
J (td)
3.55
K (t)
2.48
L (m)
2.29
M (s)
2.16
N (m)
2.06
O (m)
1.94
P (m)
1.78
Q (m)
2.13
f1 (ppm)

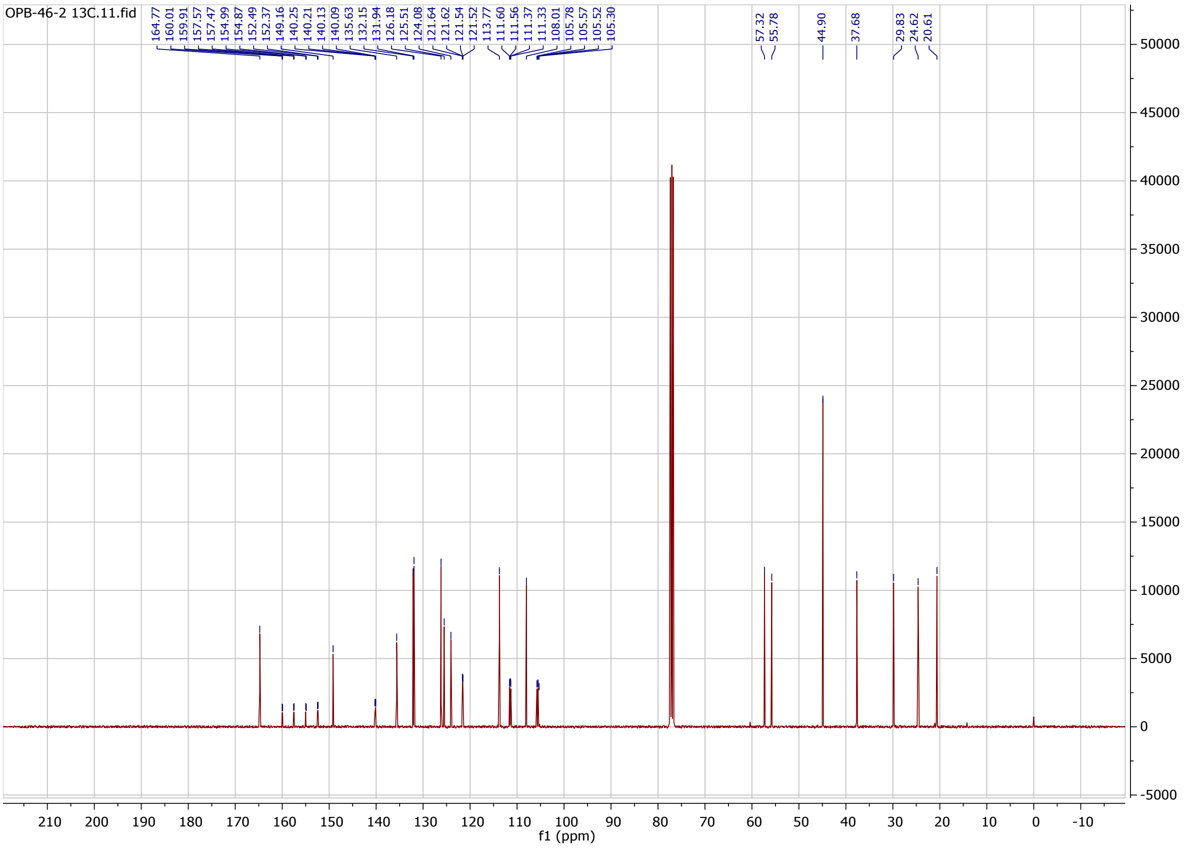
OPB-46-2 13C.11.fid
164.77
160.01
159.91
157.57
157.47
154.99
154.87
152.49
152.37
149.16
140.25
140.21
140.13
140.09
135.63
132.15
131.94
126.18
125.51
124.08
121.64
121.62
121.54
121.52
113.77
111.60
111.56
111.37
111.33
108.01
105.78
105.57
105.52
105.30
57.32
55.78
44.90
37.68
29.83
24.62
20.61
f1 (ppm)

84

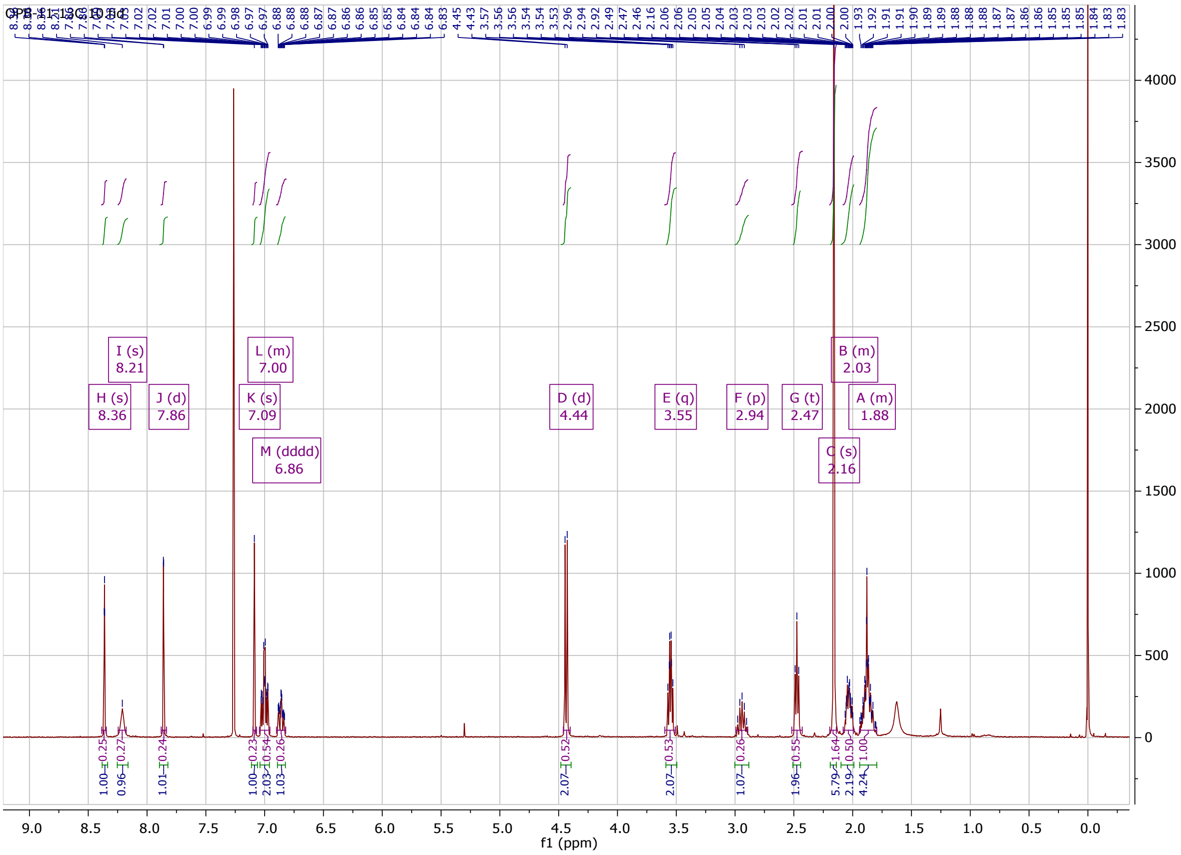

H (s)
8.36
I (s)
8.21
J (d)
7.86
K (s)
7.09
L (m)
7.00
M (dddd)
6.86
D (d)
4.44
E (q)
3.55
F (p)
2.94
G (t)
2.47
C (s)
2.16
B (m)
2.03
A (m)
1.88
f1 (ppm)

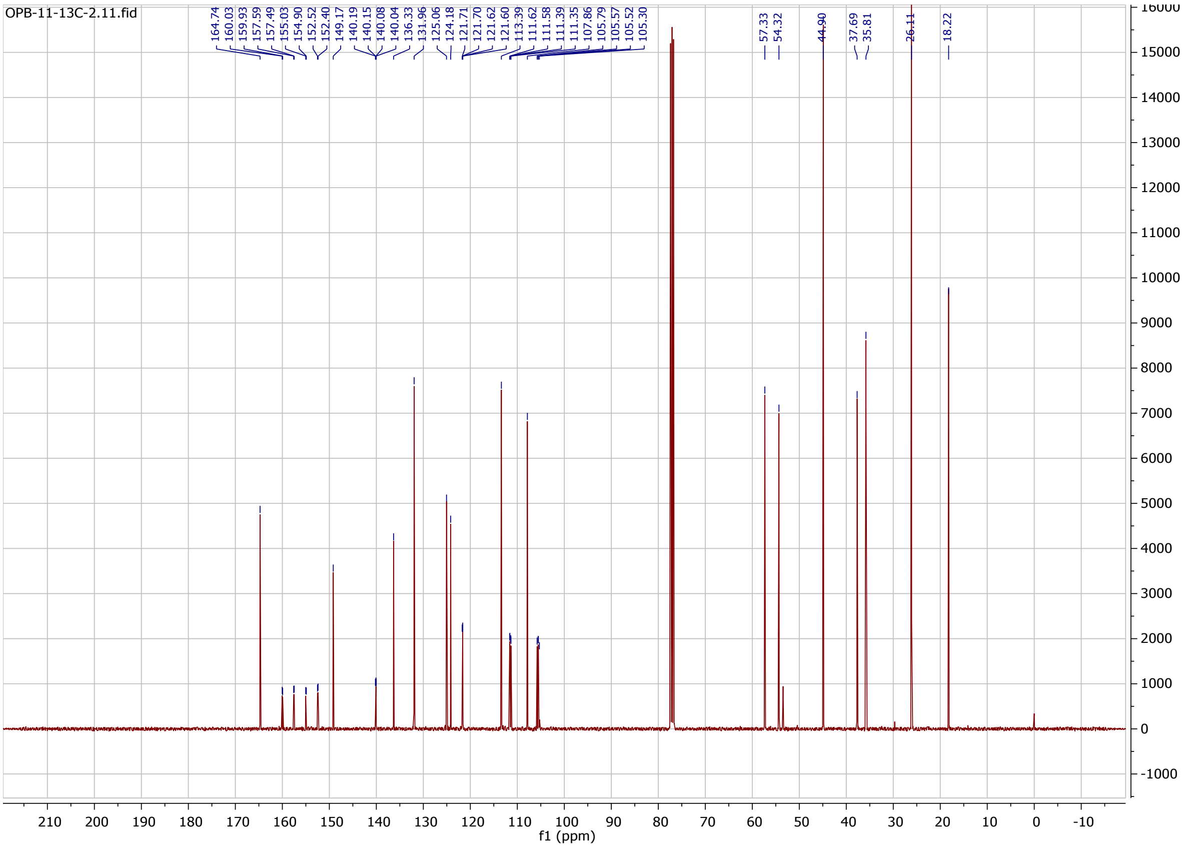

OPB-11-13C-2.11.fid
164.74
160.03
159.93
157.59
157.49
155.03
154.90
152.52
152.40
149.17
140.19
140.15
140.08
140.04
136.33
131.96
125.06
124.18
121.71
121.70
121.62
121.60
113.39
111.62
111.58
111.39
111.35
107.86
105.79
105.57
105.52
105.30
57.33
54.32
44.90
37.69
35.81
26.11
18.22
f1 (ppm)

85

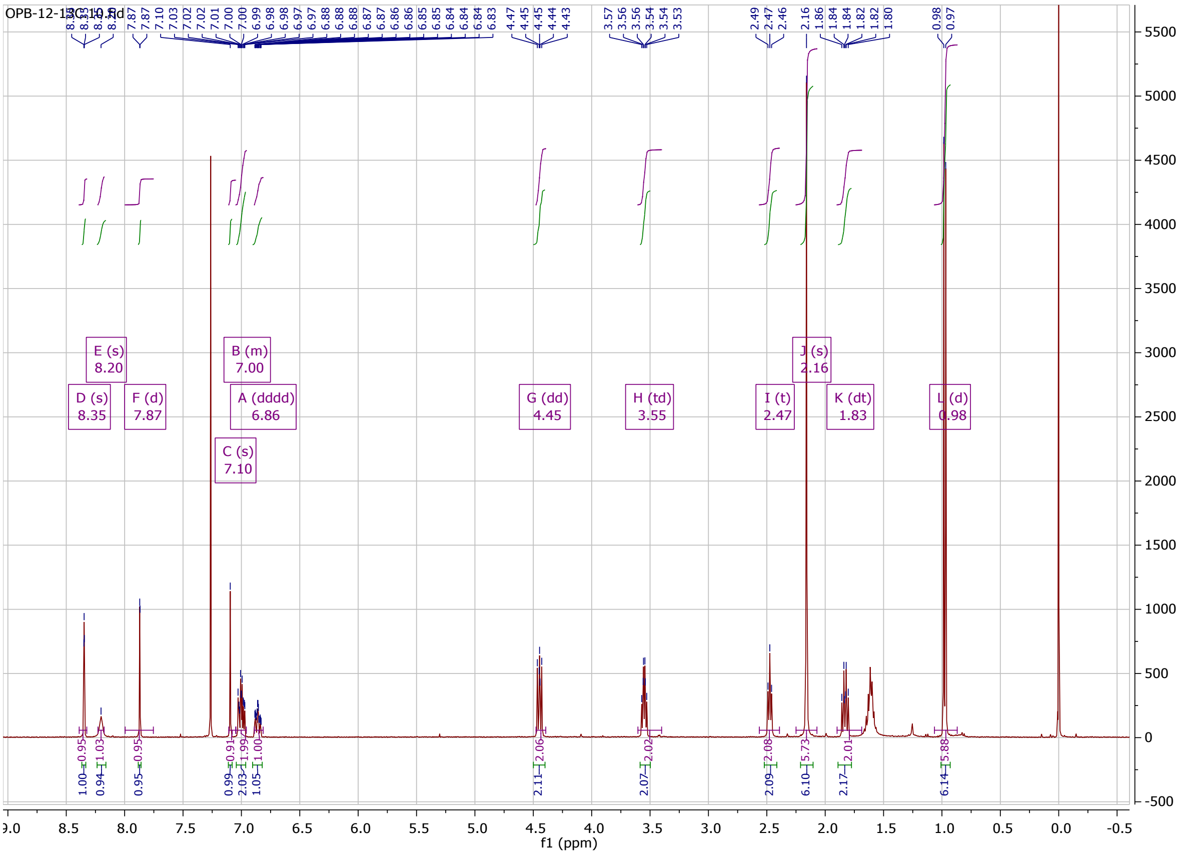

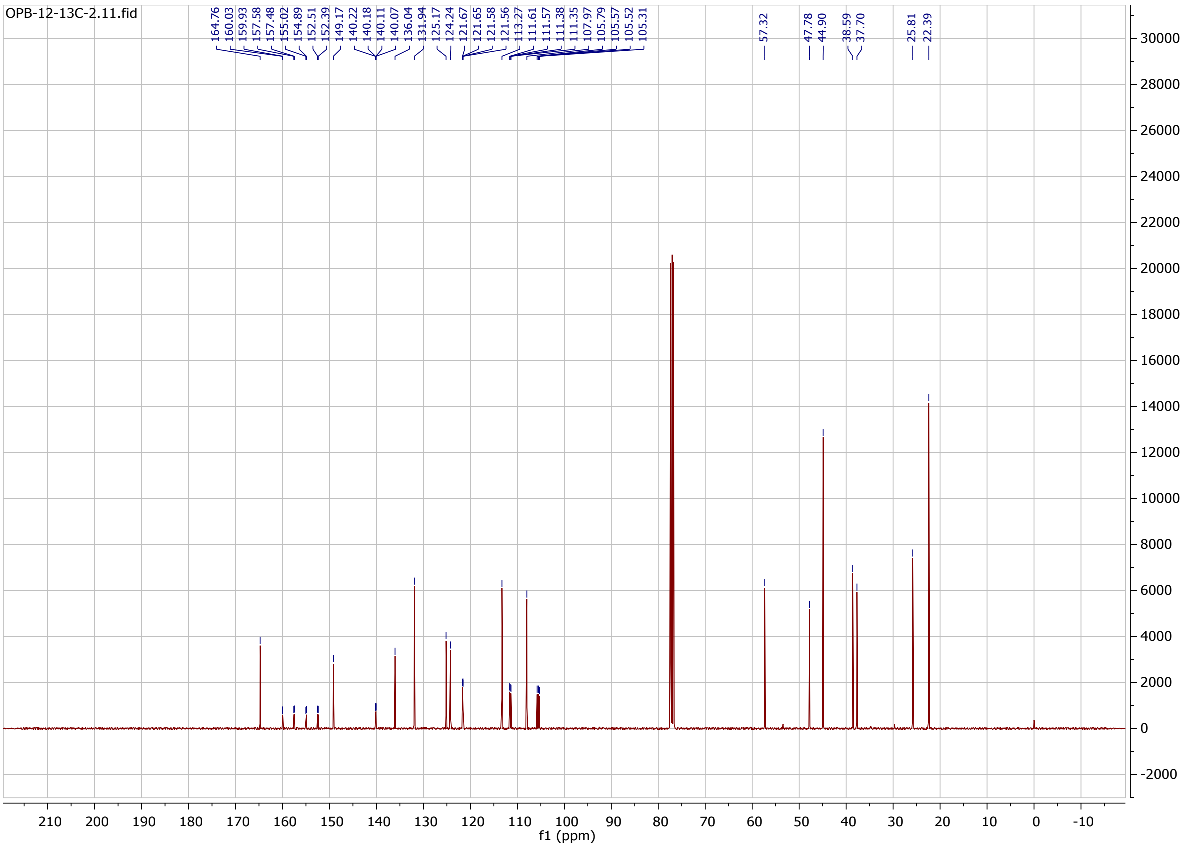

86

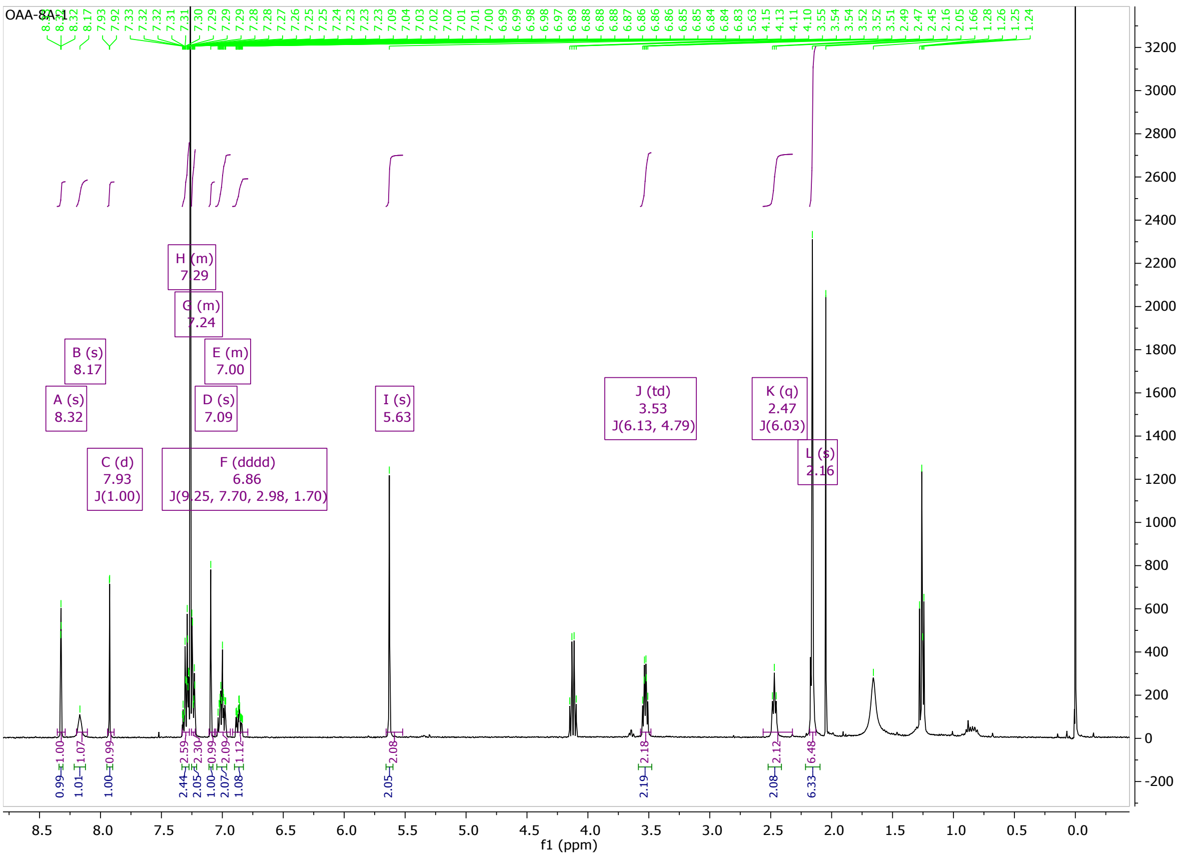

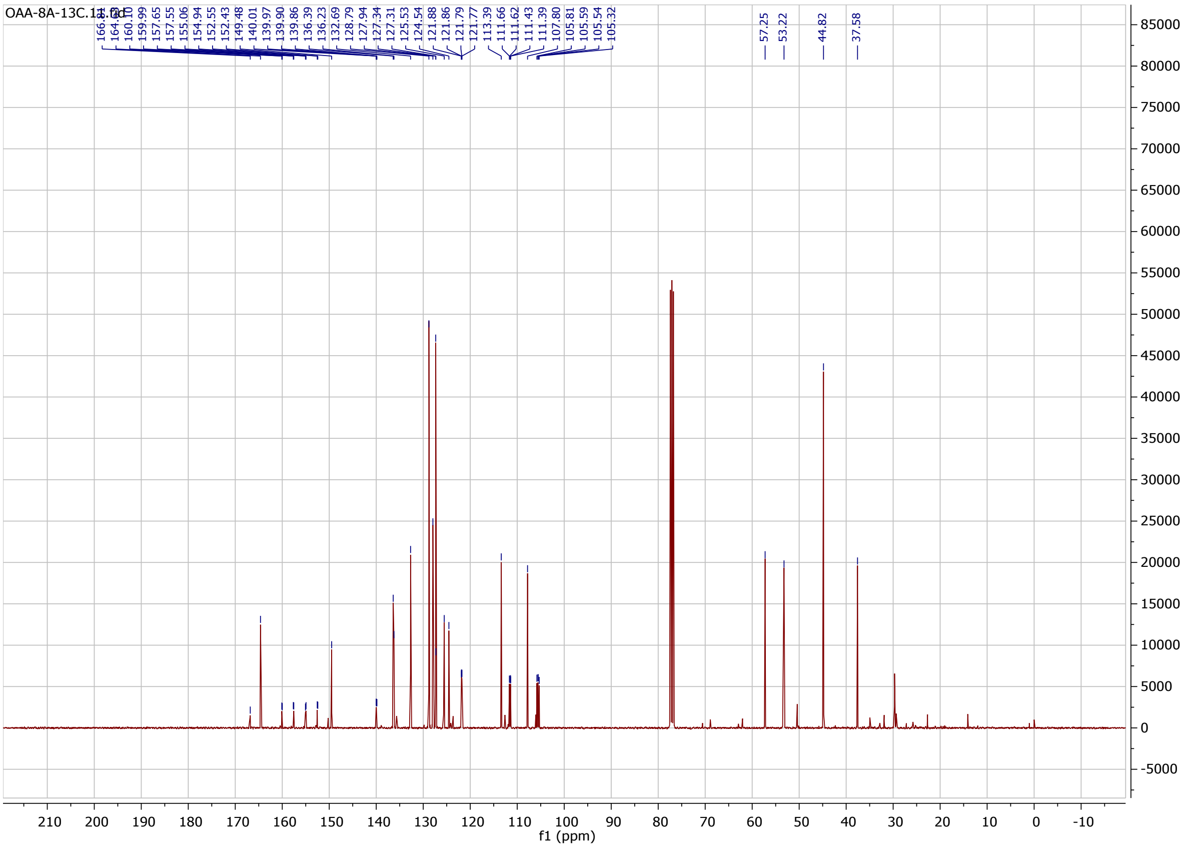

88

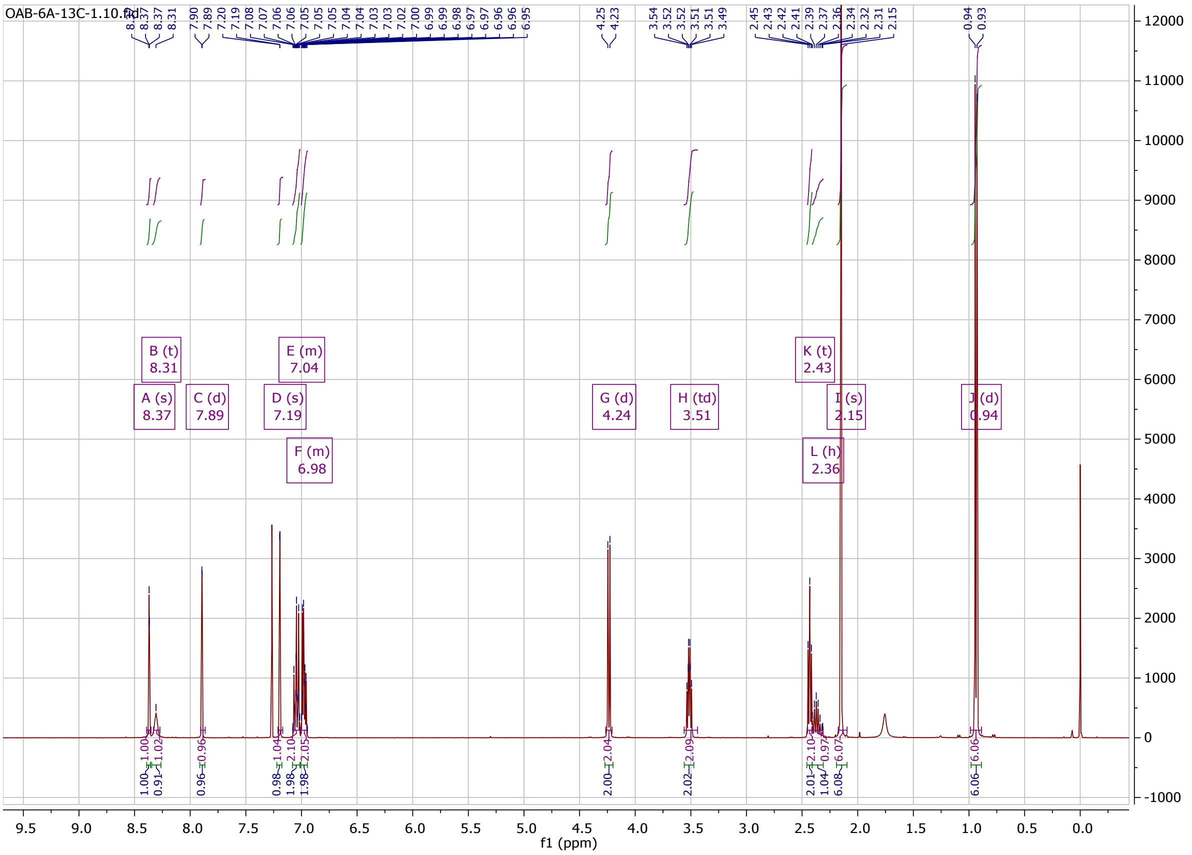
OAB-6A-13C-1.10.fid
A (s) 8.37
B (t) 8.31
C (d) 7.89
D (s) 7.19
E (m) 7.04
F (m) 6.98
G (d) 4.24
H (td) 3.51
I (s) 2.15
J (d) 0.94
K (t) 2.43
L (h) 2.36
f1 (ppm)

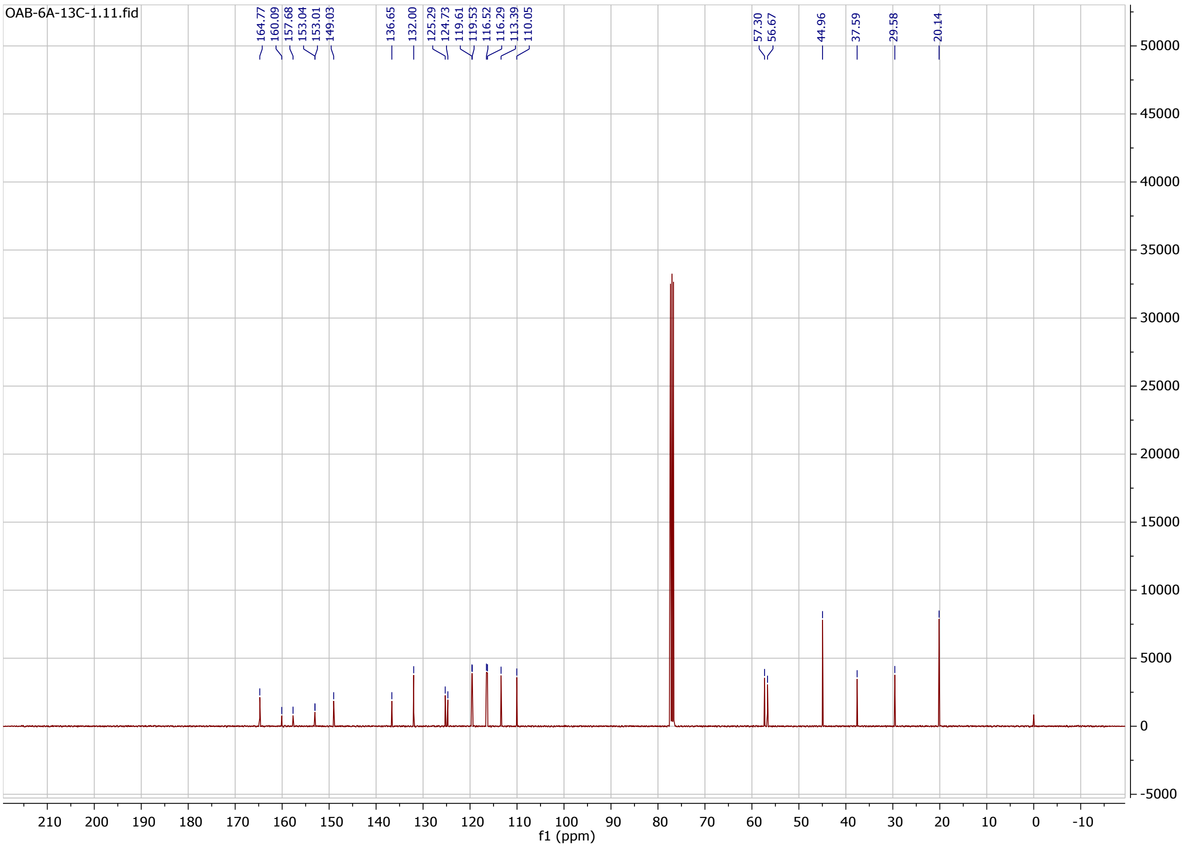
OAB-6A-13C-1.11.fid
164.77
160.09
157.68
153.04
153.01
149.03
136.65
132.00
125.29
124.73
119.61
119.53
116.52
116.29
113.39
110.05
57.30
56.67
44.96
37.59
29.58
20.14
f1 (ppm)

89

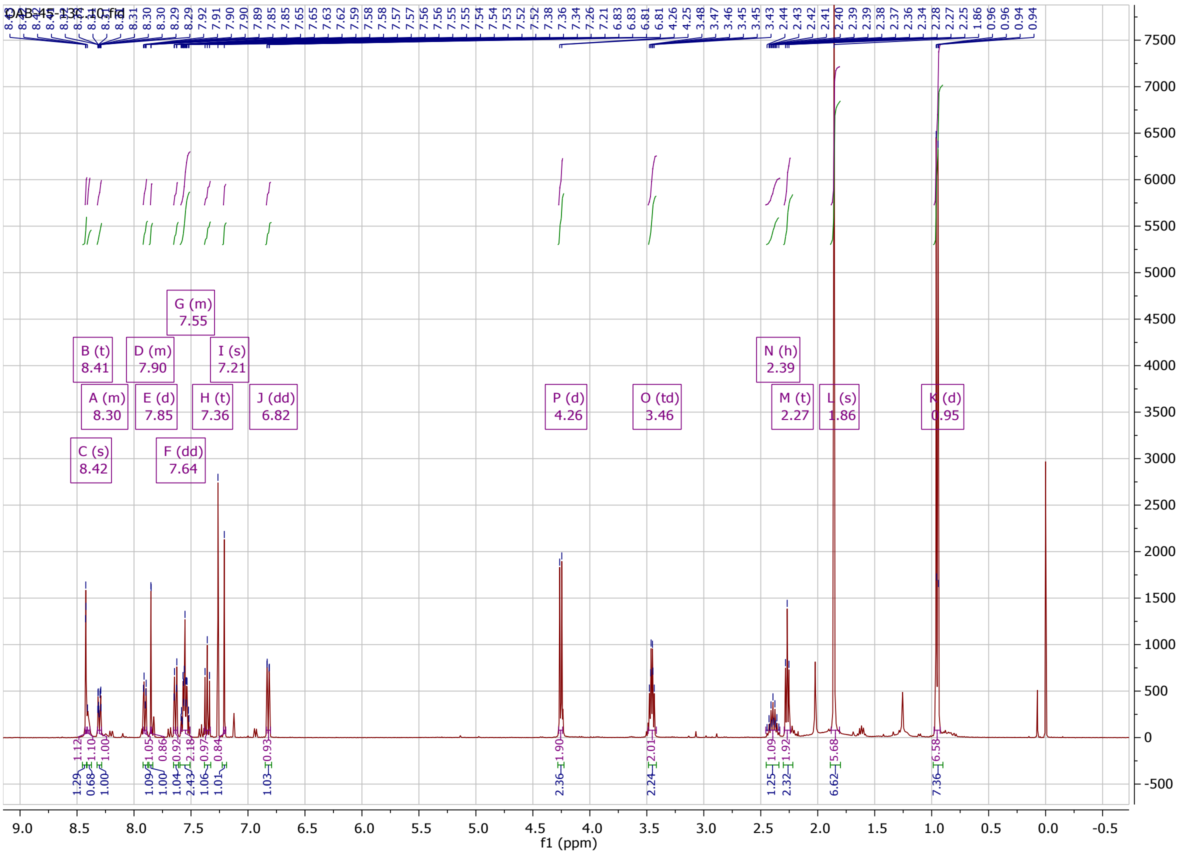
OAB-45-13C.10.fid
B (t) 8.41
D (m) 7.90
G (m) 7.55
I (s) 7.21
A (m) 8.30
E (d) 7.85
H (t) 7.36
J (dd) 6.82
C (s) 8.42
F (dd) 7.64
P (d) 4.26
O (td) 3.46
N (h) 2.39
M (t) 2.27
L (s) 1.86
K (d) 0.95
f1 (ppm)

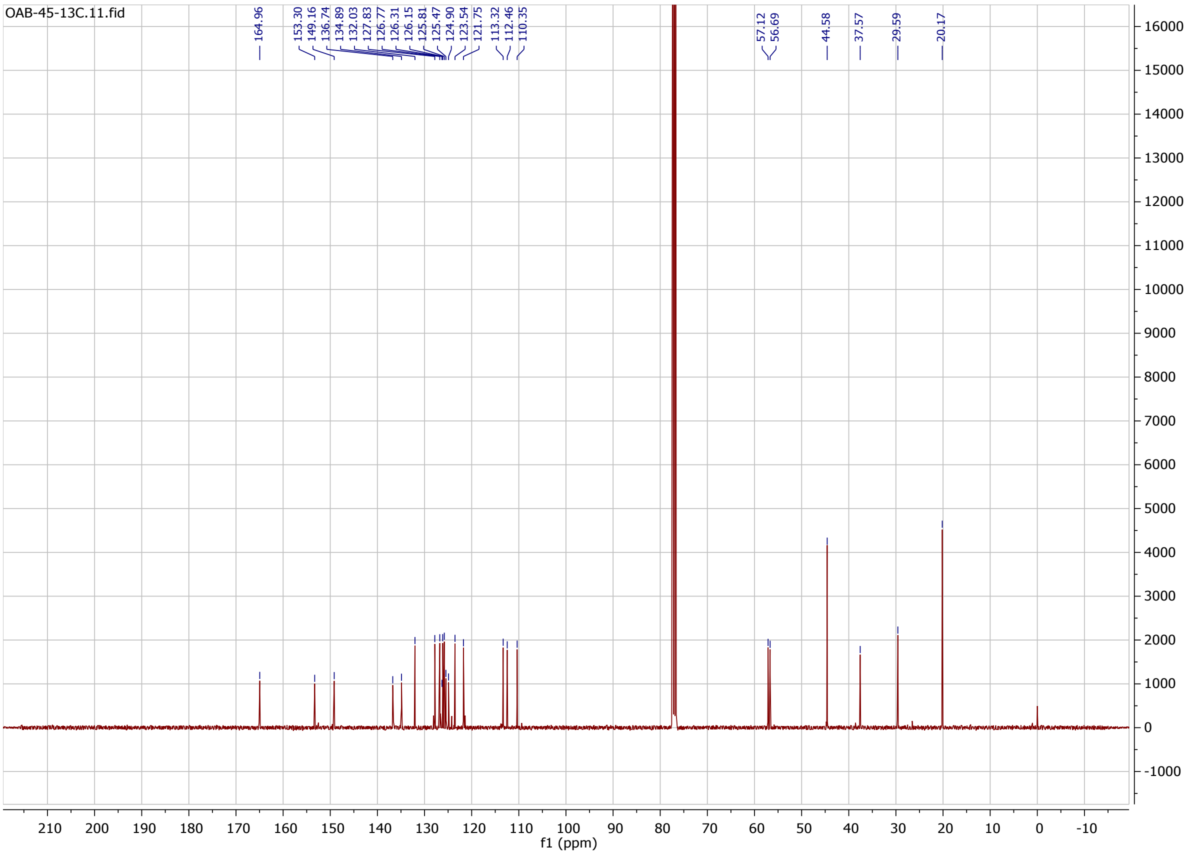
OAB-45-13C.11.fid
164.96
153.30
149.16
136.74
134.89
132.03
127.83
126.77
126.31
126.15
125.81
125.47
124.90
123.54
121.75
113.32
112.46
110.35
57.12
56.69
44.58
37.57
29.59
20.17
f1 (ppm)

90

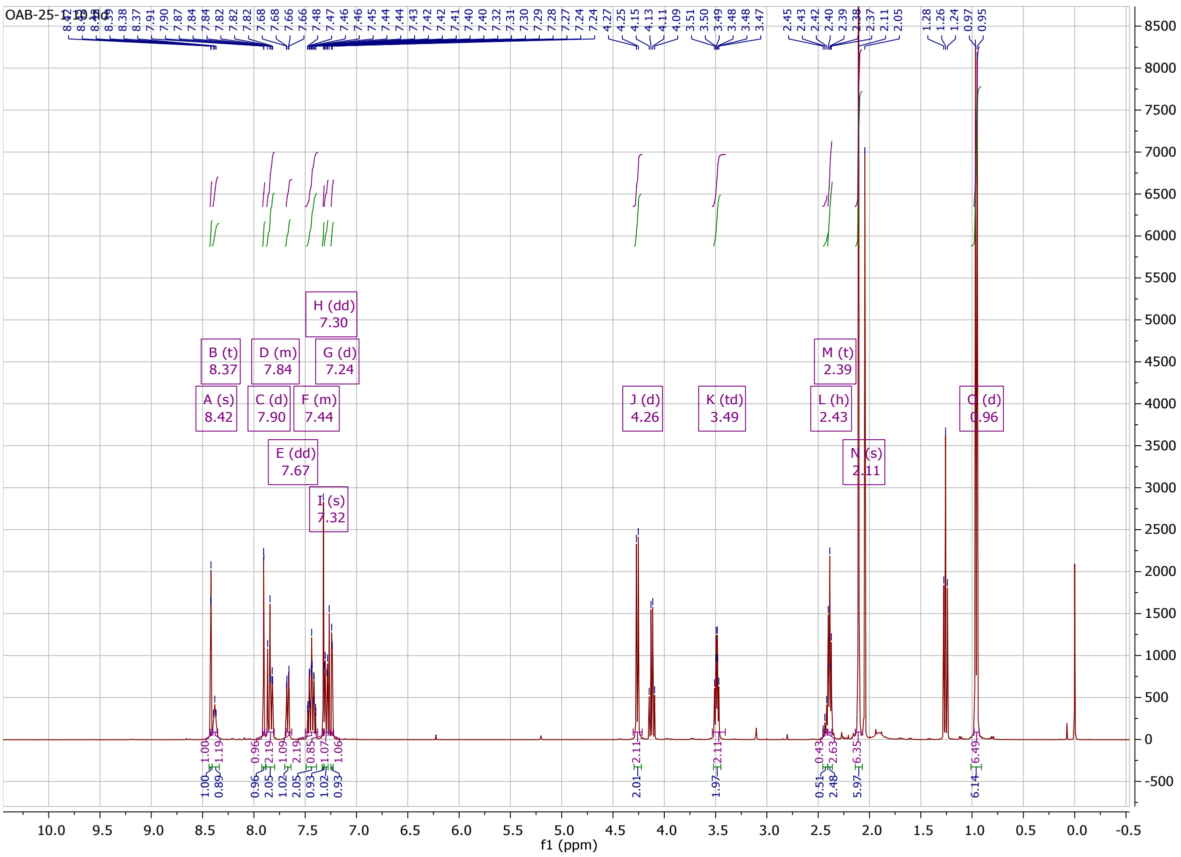
OAB-25-1 1H.10.fid
A (s) 8.42
B (t) 8.37
C (d) 7.90
D (m) 7.84
E (dd) 7.67
F (m) 7.44
G (d) 7.24
H (dd) 7.30
I (s) 7.32
J (d) 4.26
K (td) 3.49
L (h) 2.43
M (t) 2.39
N (s) 2.11
O (d) 0.96
f1 (ppm)

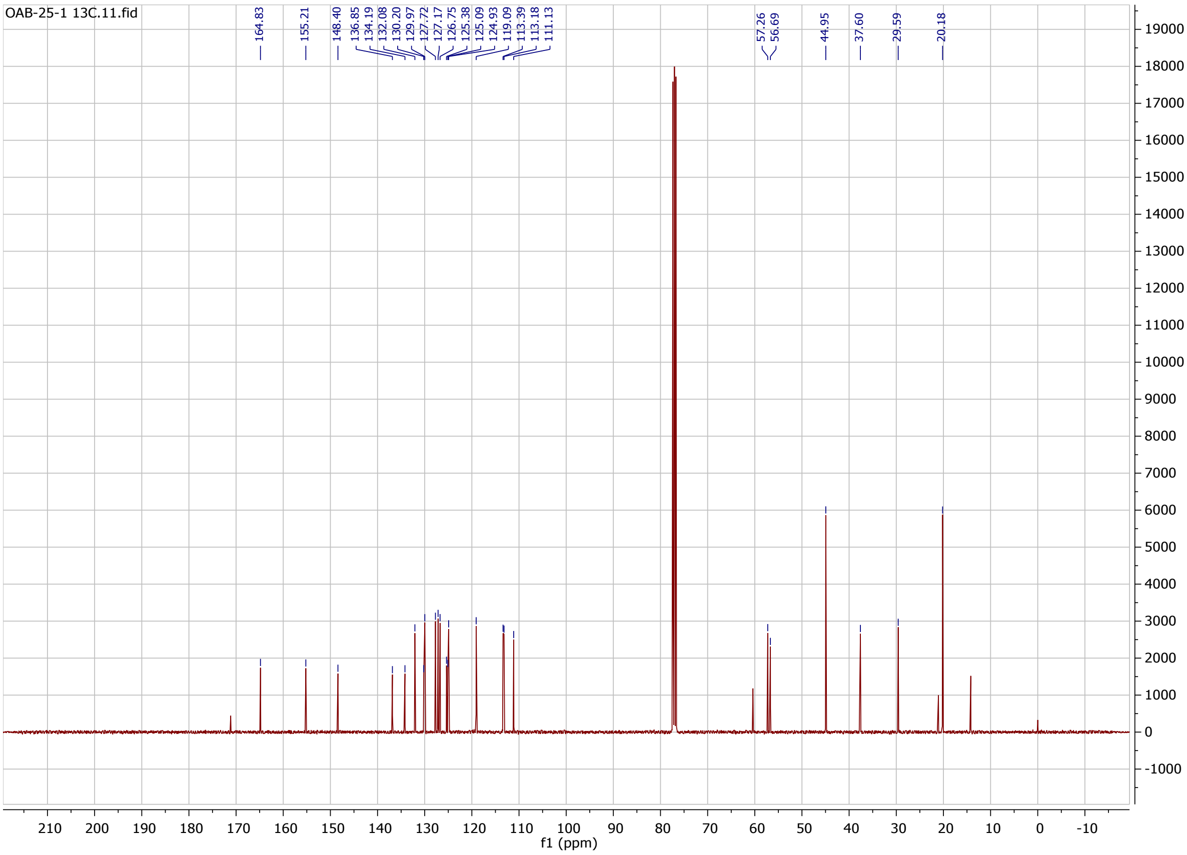
OAB-25-1 13C.11.fid
164.83
155.21
148.40
136.85
134.19
132.08
130.20
129.97
127.72
127.17
126.75
125.38
125.09
124.93
119.09
113.39
113.18
111.13
57.26
56.69
44.95
37.60
29.59
20.18
f1 (ppm)

91

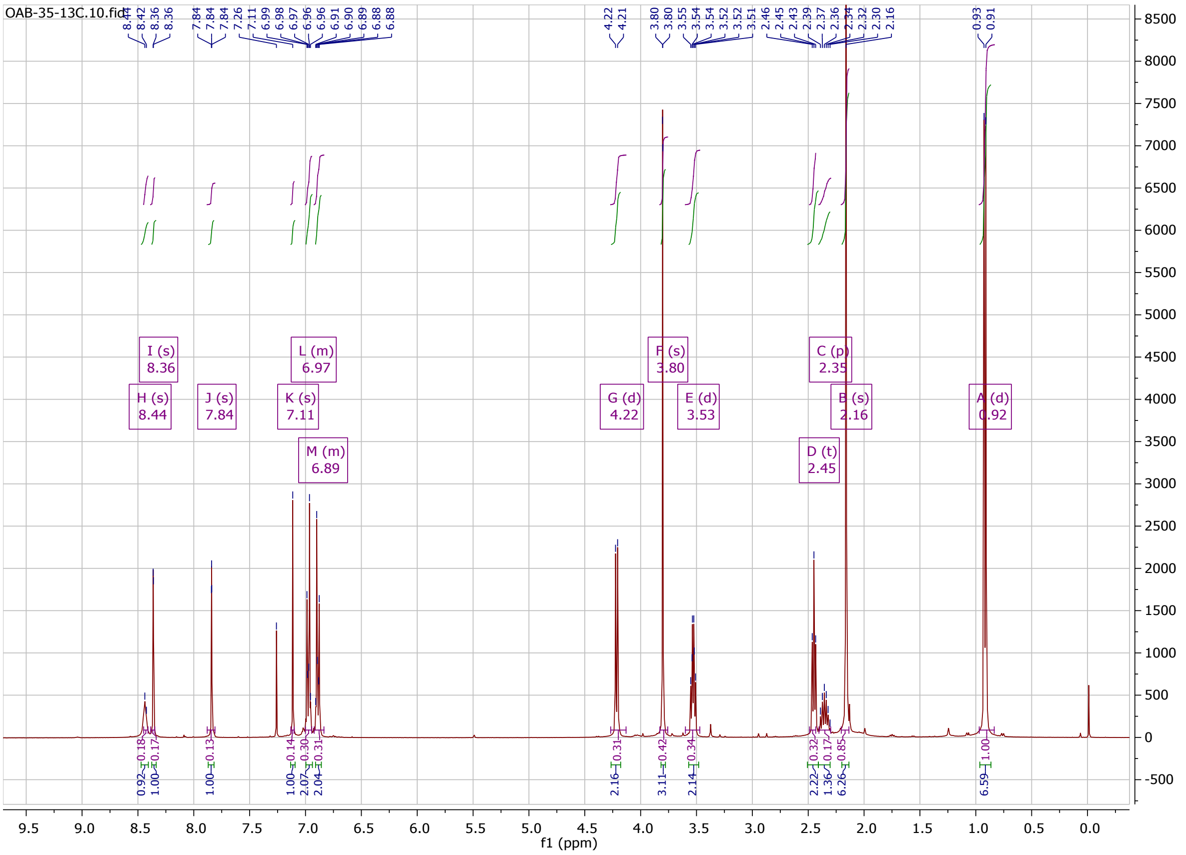
OAB-35-13C.10.fid
8.44
8.42
8.36
8.36
7.84
7.84
7.84
7.26
7.11
6.99
6.98
6.97
6.96
6.96
6.91
6.90
6.89
6.88
6.88
4.22
4.21
3.80
3.80
3.55
3.54
3.54
3.52
3.52
3.51
2.46
2.45
2.43
2.39
2.37
2.36
2.34
2.32
2.30
2.16
0.93
0.91
I (s) 8.36
H (s) 8.44
J (s) 7.84
L (m) 6.97
K (s) 7.11
M (m) 6.89
G (d) 4.22
F (s) 3.80
E (d) 3.53
C (p) 2.35
B (s) 2.16
D (t) 2.45
A (d) 0.92
0.92
1.00
1.00
1.00
2.07
2.04
2.16
3.11
2.14
2.22
1.36
6.26
6.59
f1 (ppm)

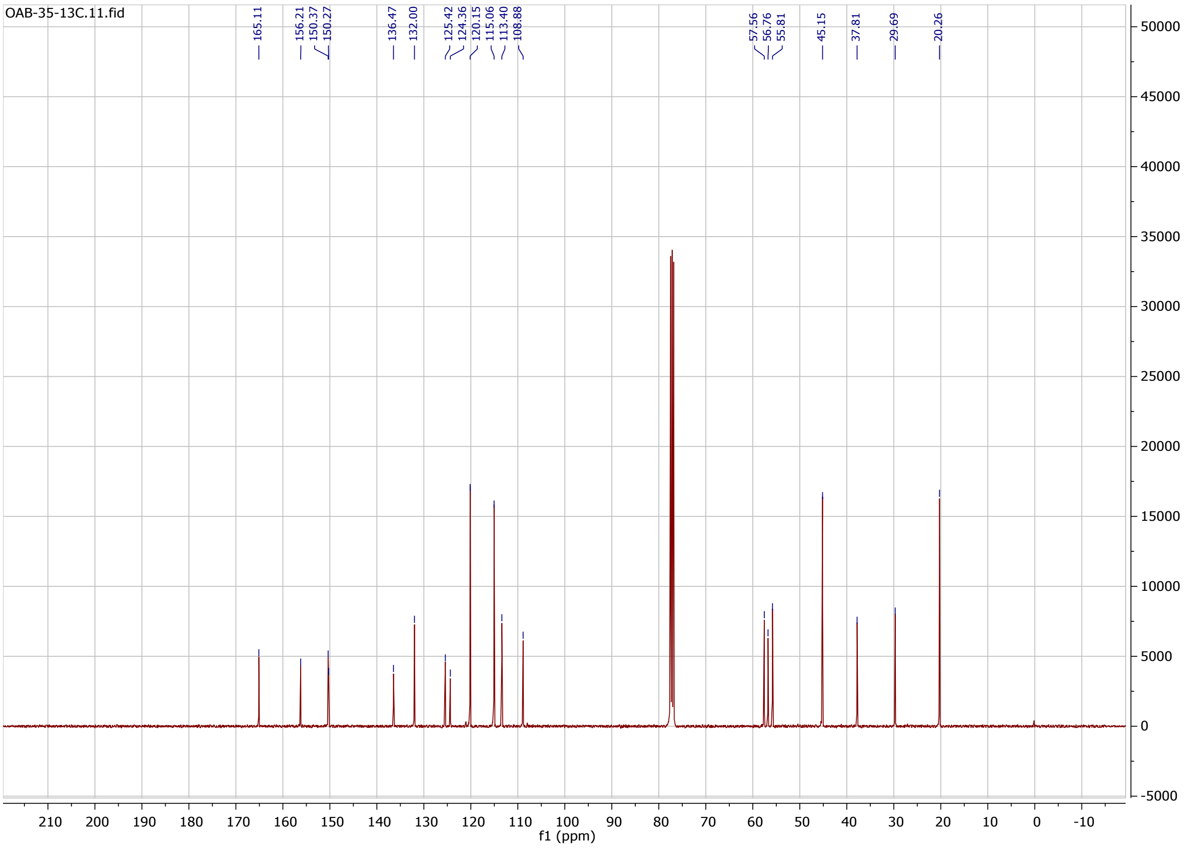
OAB-35-13C.11.fid
165.11
156.21
150.37
150.27
136.47
132.00
125.42
124.36
120.15
115.06
113.40
108.88
57.56
56.76
55.81
45.15
37.81
29.69
20.26
f1 (ppm)

92

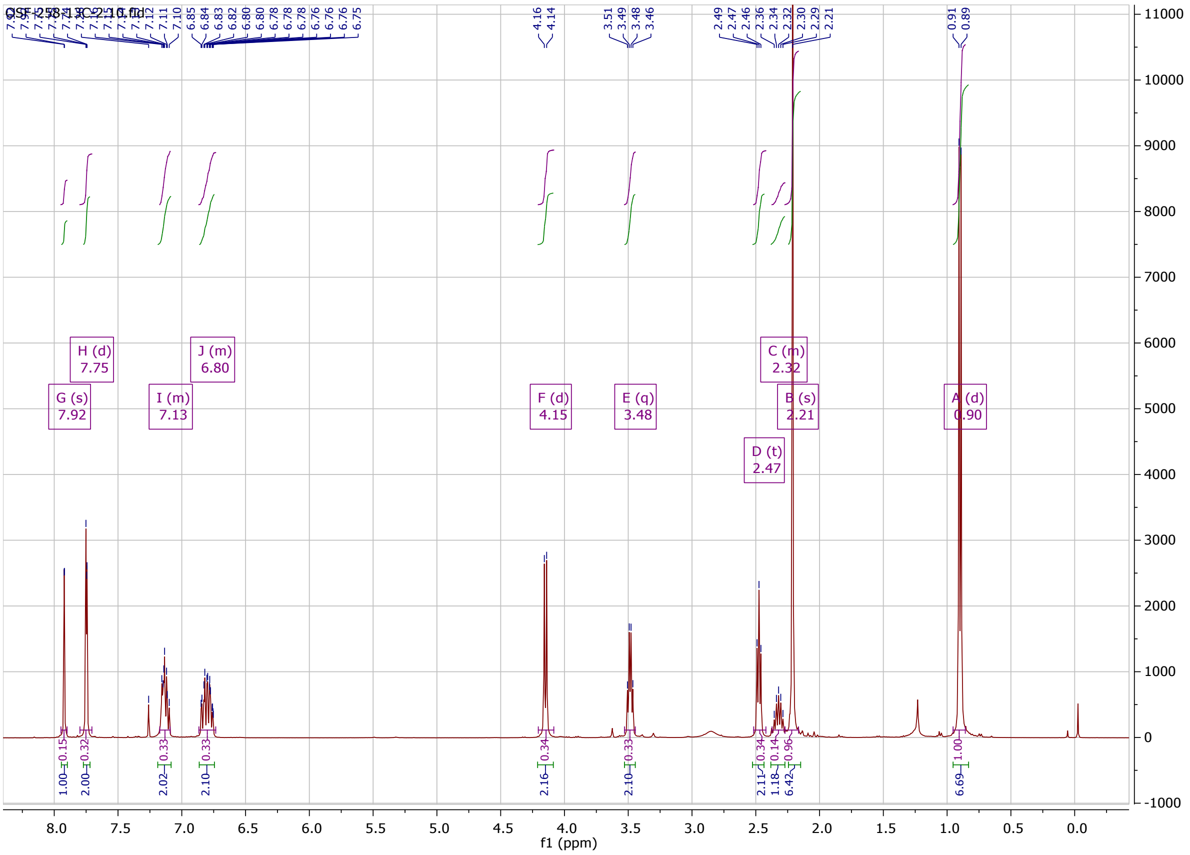

OSF-258-1H-2.10.fid
G (s) 7.92
H (d) 7.75
I (m) 7.13
J (m) 6.80
F (d) 4.15
E (q) 3.48
D (t) 2.47
C (m) 2.32
B (s) 2.21
A (d) 0.90
f1 (ppm)

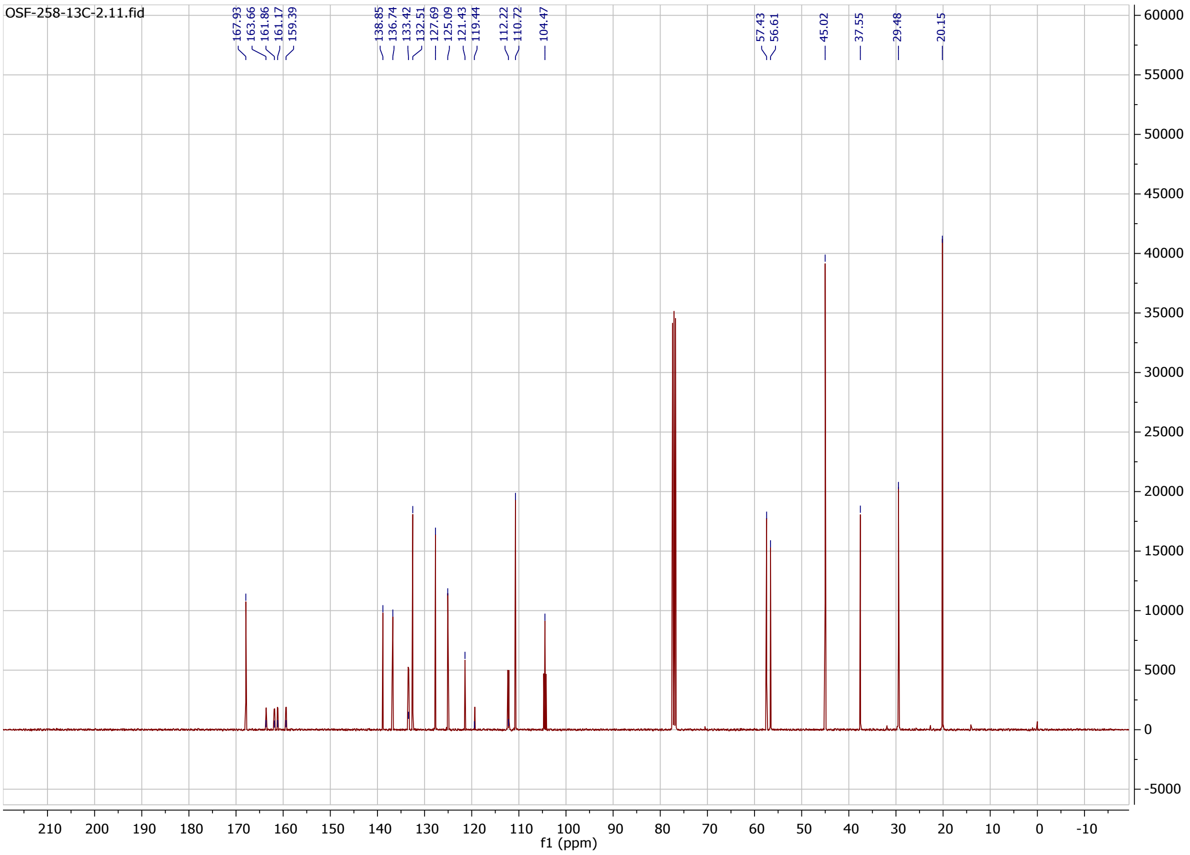

OSF-258-13C-2.11.fid
167.93
163.66
161.86
161.17
159.39
138.85
136.74
133.42
132.51
127.69
125.09
121.43
119.44
112.22
110.72
104.47
57.43
56.61
45.02
37.55
29.48
20.15
f1 (ppm)

93

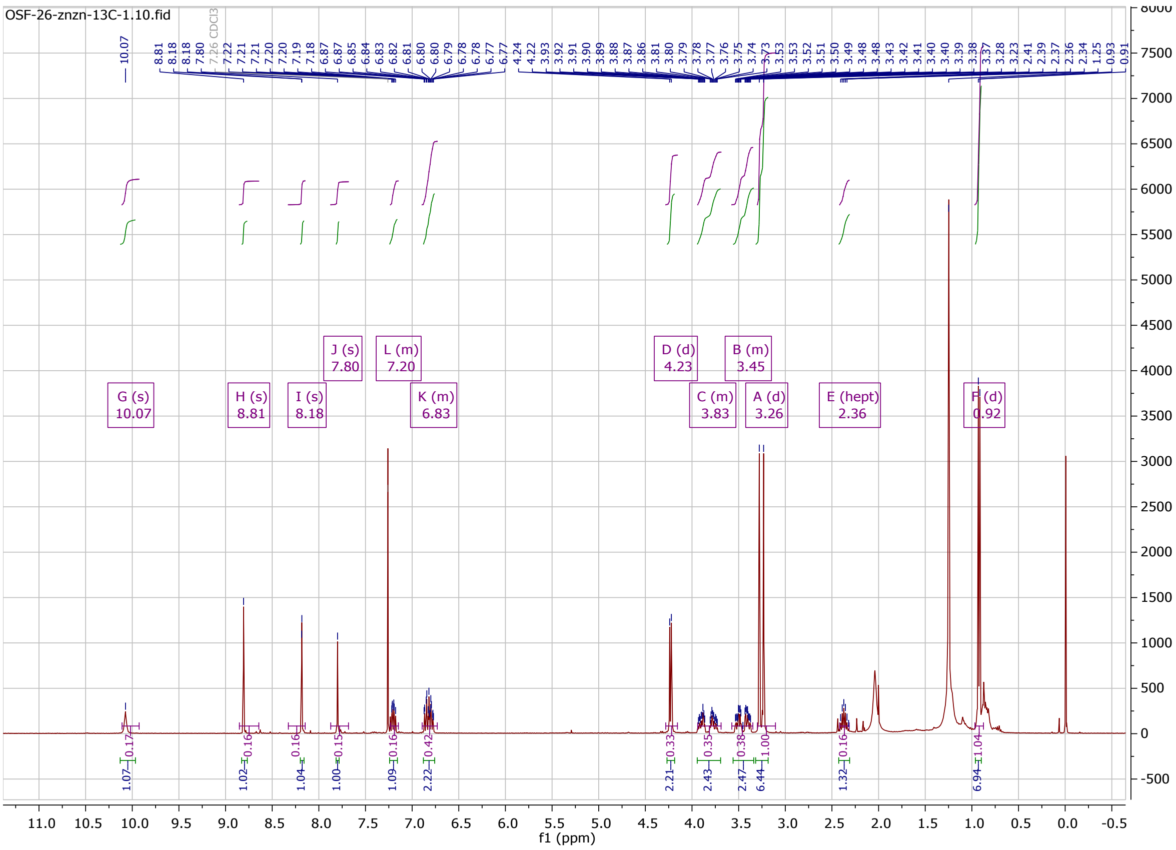

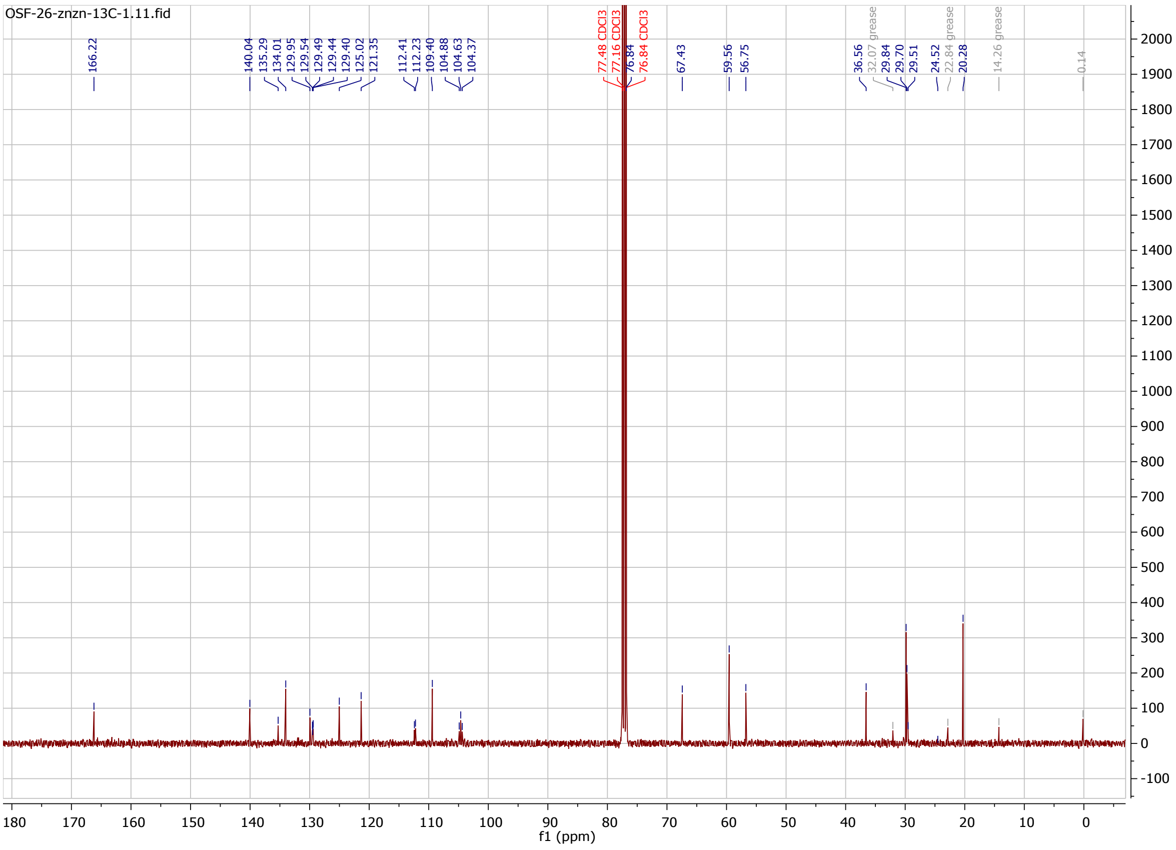

Note: The additional signals at 32.07, 22.84 and 14.26 ppm correspond to traces of grease.

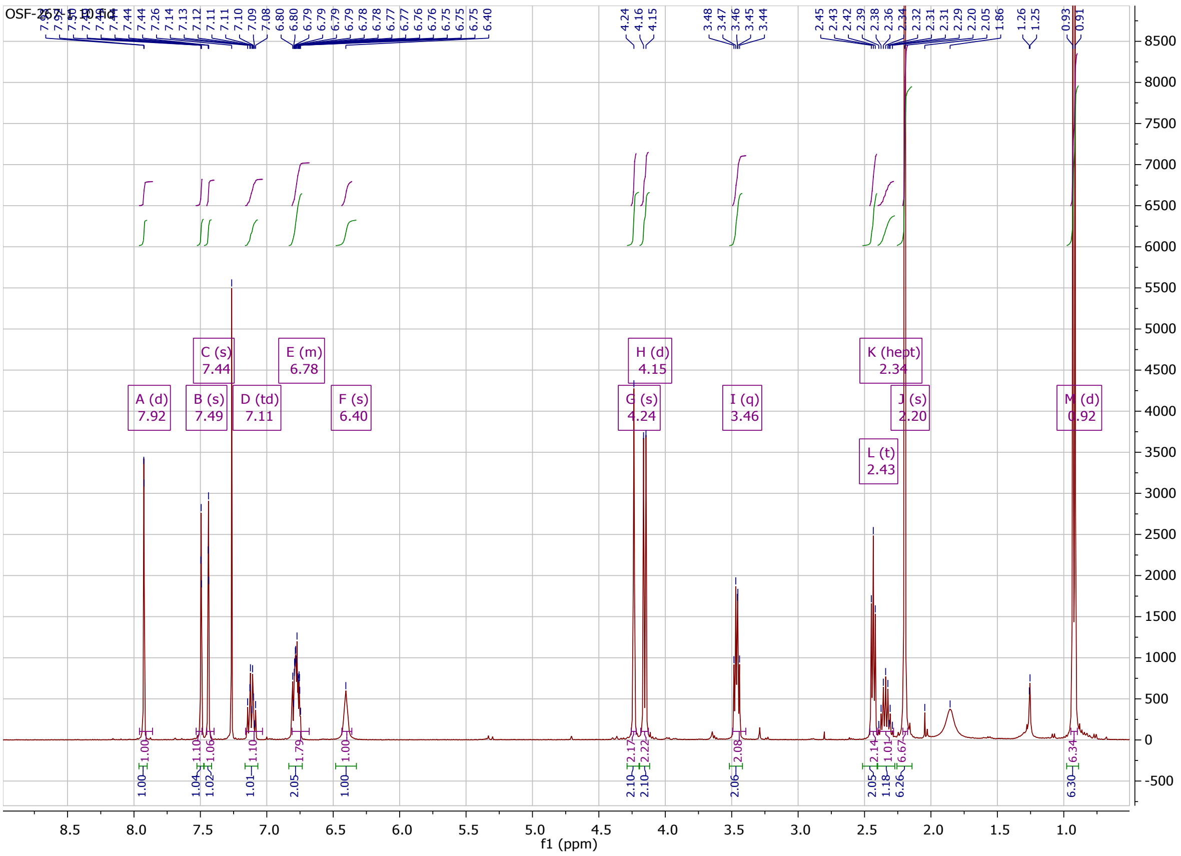

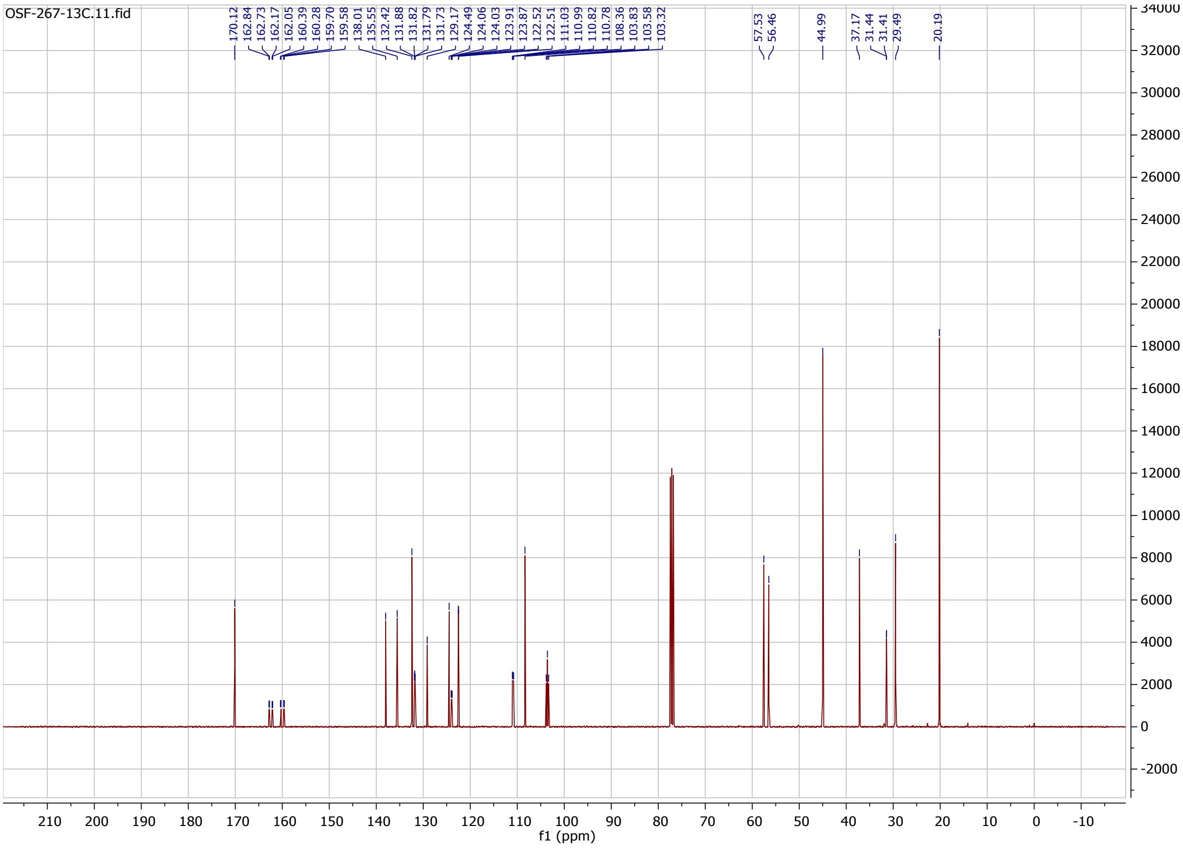

Chromatogram and Results

Injection Details

Injection Name:	**Cpd. 94**	*Run Time (min):*	**8,00**
Vial Number:	**BA8**	*Injection Volume:*	**5,00**
Injection Type:	**Unknown**	*Channel:*	**UV_VIS_2**
Calibration Level:		*Wavelength:*	**254**
Instrument Method:	**General method (0.1TFA_MeCN_20-90)_FKKT**	*Bandwidth:*	**16**
Processing Method:	**Quantitative**	*Dilution Factor:*	**1,0000**
Injection Date/Time:	**31.maj.23 19:36**	*Sample Weight:*	**1,0000**

Chromatogram

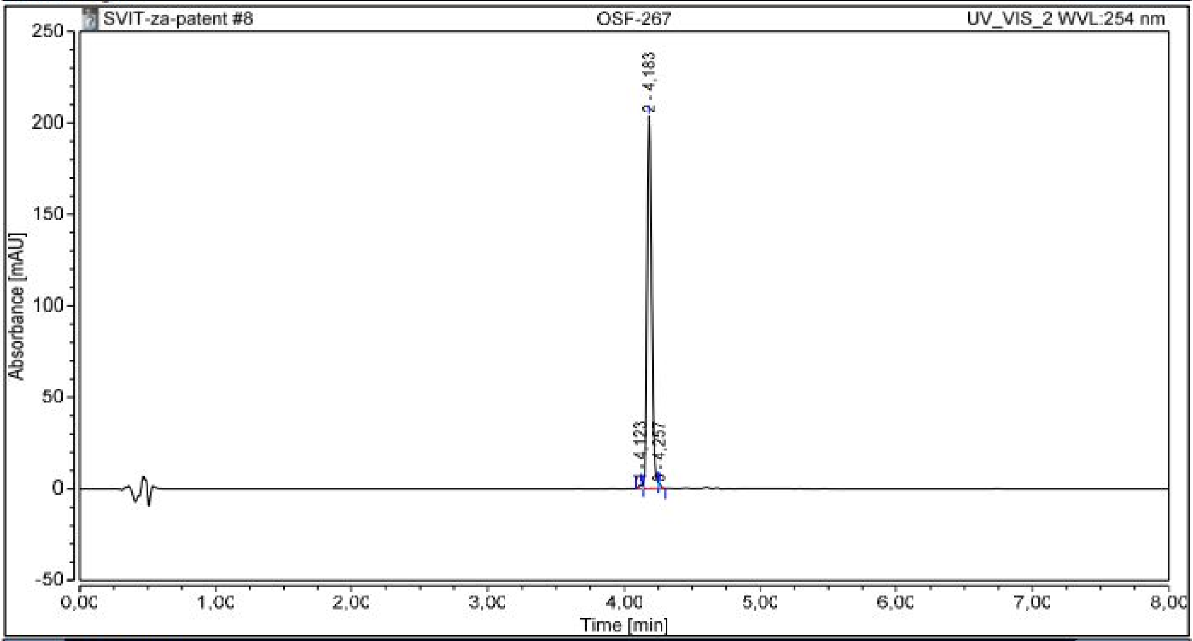

Integration Results

No.	Peak Name	Retention Time min	Area mAU*min	Height mAU	Relative Area %	Relative Height %	Amount n.a.
1		4,123	0,065	2,232	0,77	1,07	n.a.
2		4,183	8,285	203,562	98,57	97,68	n.a.
3		4,257	0,055	2,613	0,65	1,25	n.a.
**Total:**			**8,405**	**208,406**	**100,00**	**100,00**	

95

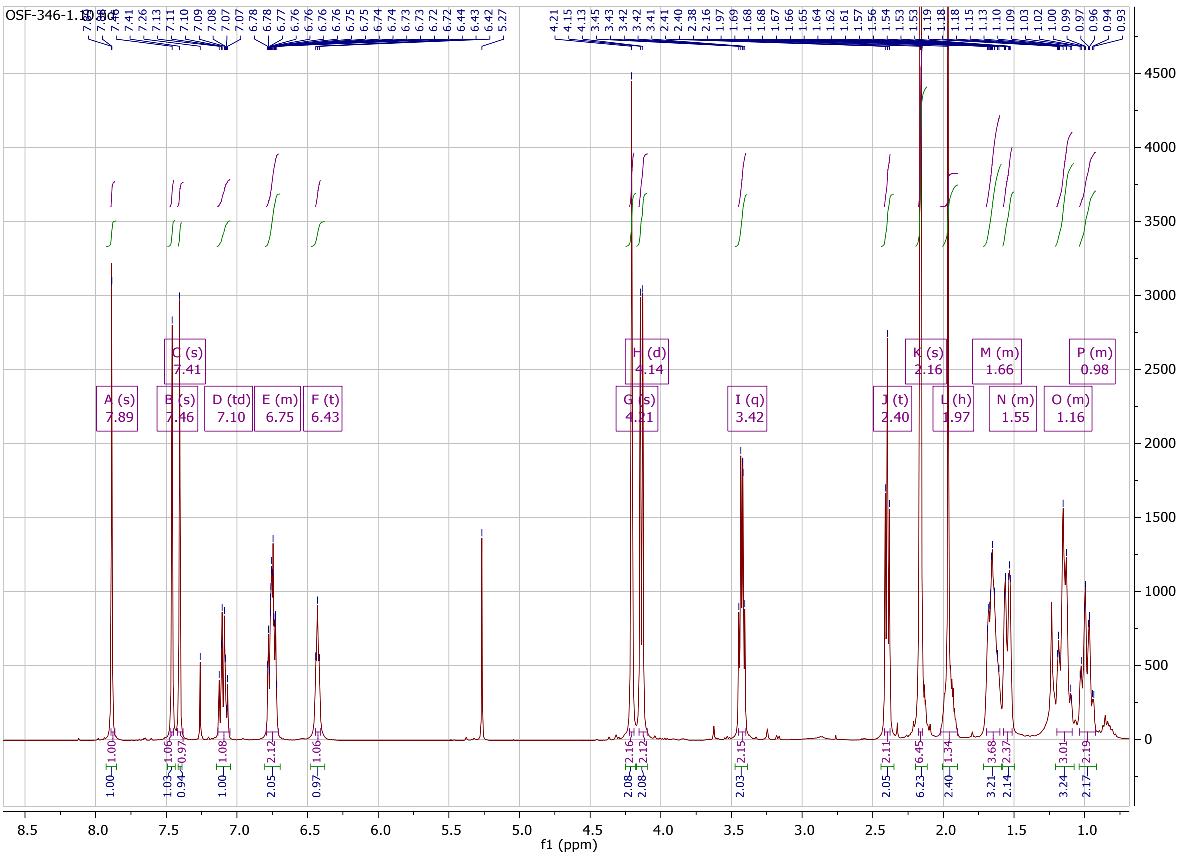

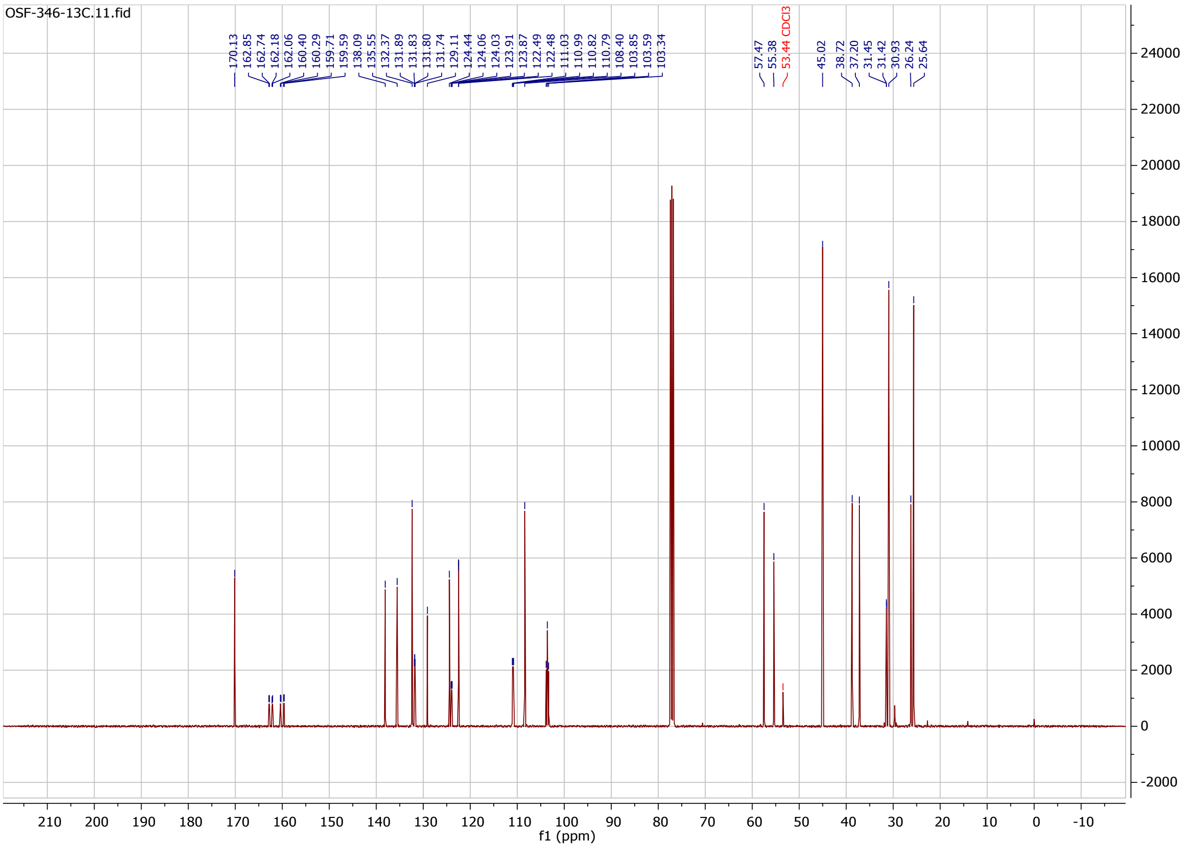

Note: The additional signal at 53.44 ppm corresponds to the residual DCM after solvent evaporation.

Chromatogram and Results

Injection Details

Injection Name:	**Cpd. 95**	*Run Time (min):*	**8,00**
Vial Number:	**BB3**	*Injection Volume:*	**5,00**
Injection Type:	**Unknown**	*Channel:*	**UV_VIS_2**
Calibration Level:		*Wavelength:*	**254**
Instrument Method:	**General method (0.1TFA_MeCN_20-90)_FKKT**	*Bandwidth:*	**16**
Processing Method:	**Quantitative**	*Dilution Factor:*	**1,0000**
Injection Date/Time:	**31.maj.23 20:11**	*Sample Weight:*	**1,0000**

Chromatogram

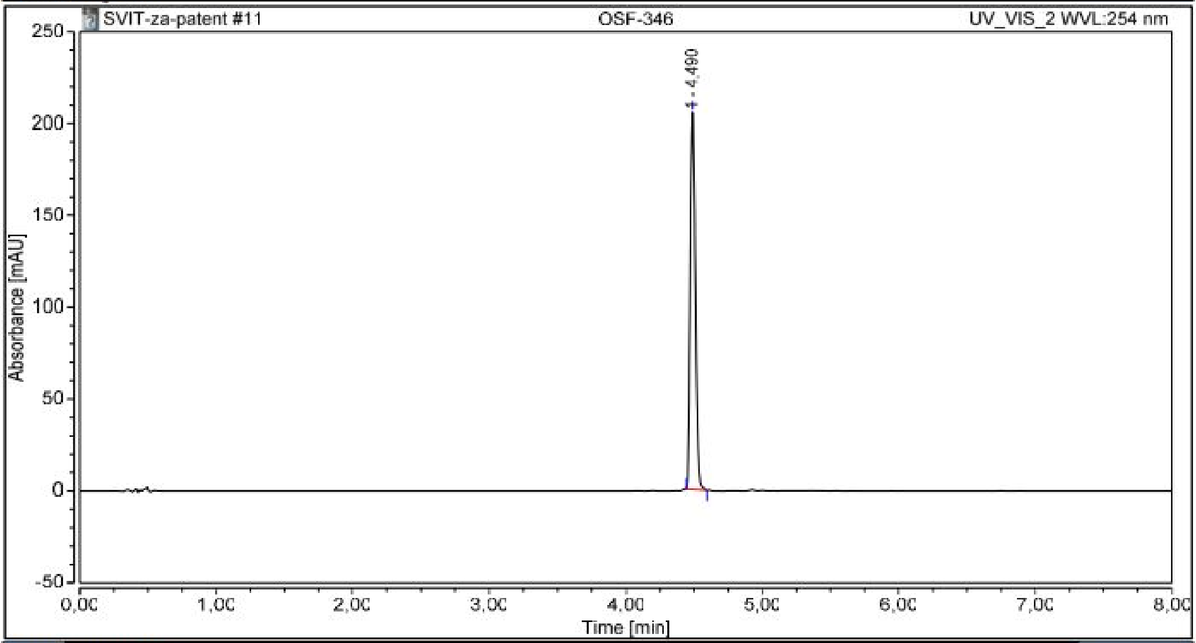

Integration Results

No.	Peak Name	Retention Time min	Area mAU*min	Height mAU	Relative Area %	Relative Height %	Amount n.a.
1		4,490	8,781	205,209	100,00	100,00	n.a.
**Total:**			**8,781**	**205,209**	**100,00**	**100,00**	

96

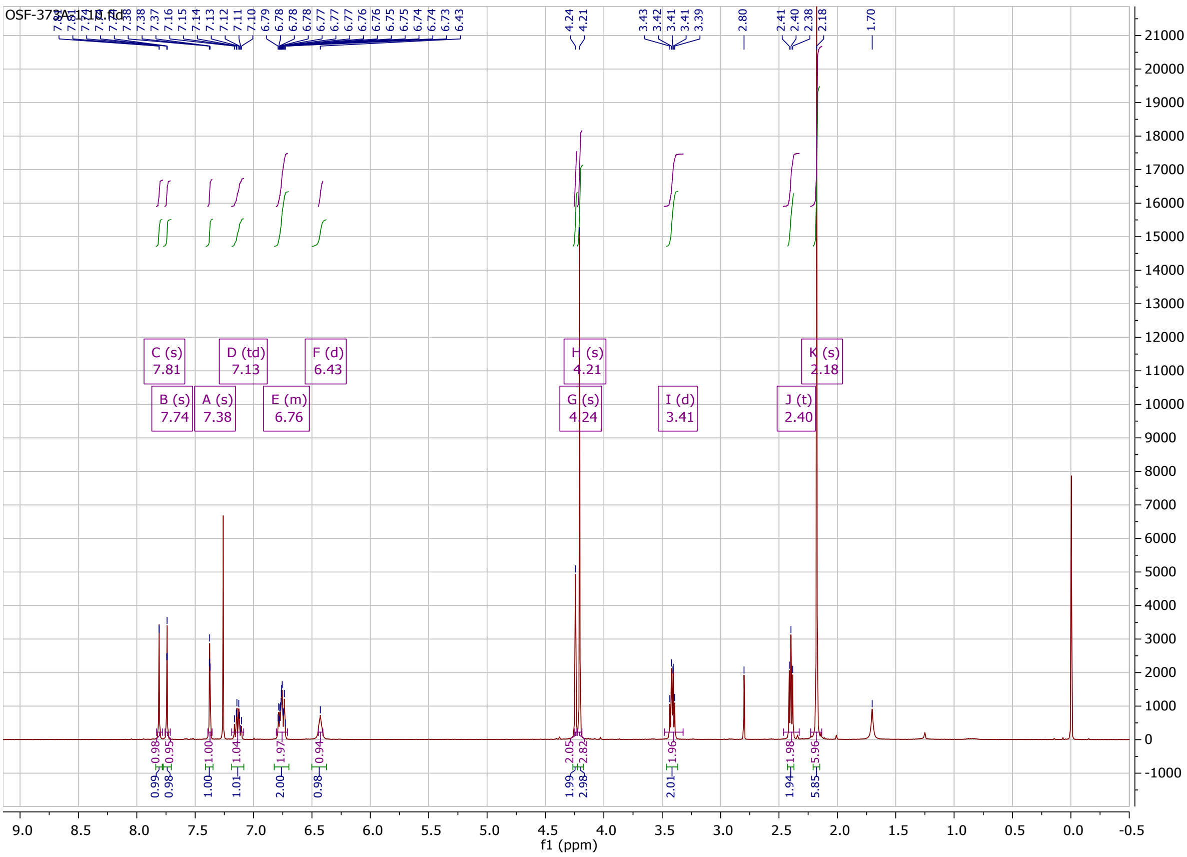
OSF-373A-1H.fid
C (s) 7.81
B (s) 7.74
A (s) 7.38
D (td) 7.13
E (m) 6.76
F (d) 6.43
G (s) 4.24
H (s) 4.21
I (d) 3.41
J (t) 2.40
K (s) 2.18
f1 (ppm)

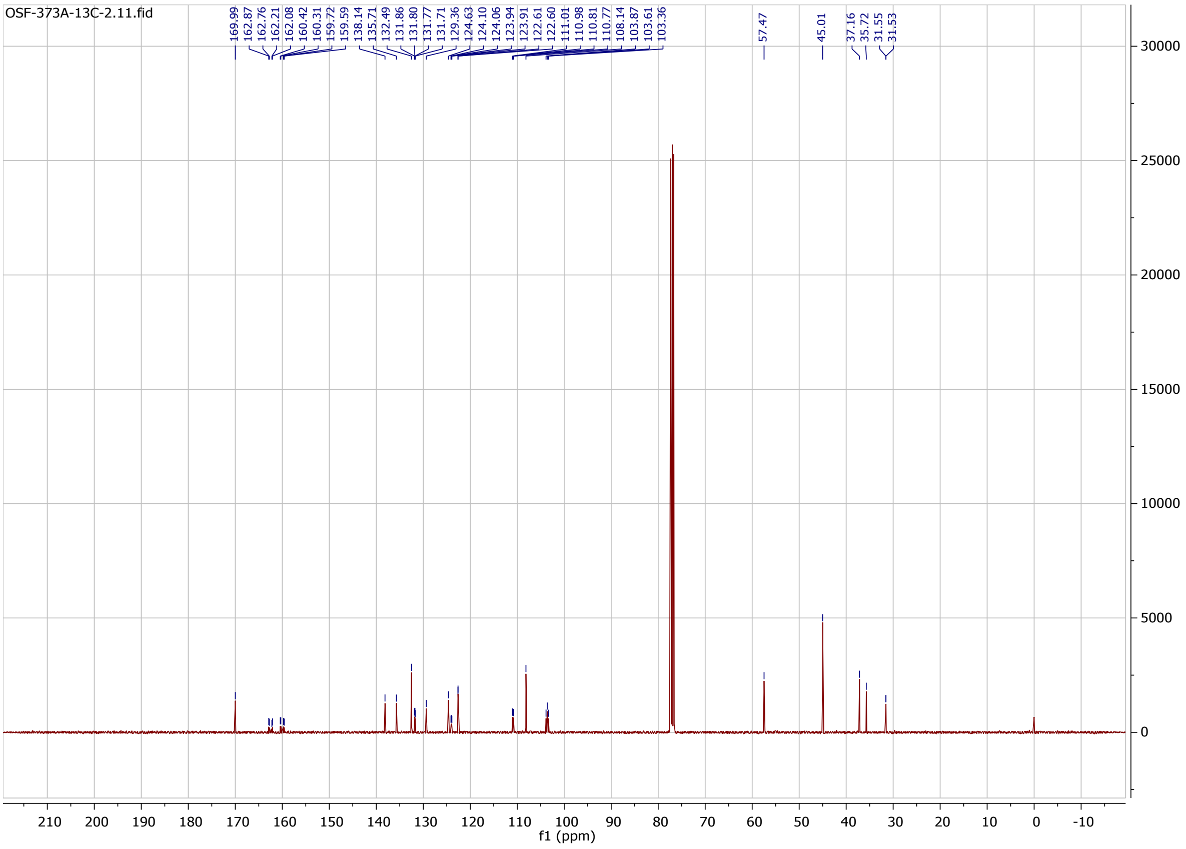
OSF-373A-13C-2.11.fid
169.99
162.87
162.76
162.21
162.08
160.42
160.31
159.72
159.59
138.14
135.71
132.49
131.86
131.80
131.77
131.71
129.36
124.63
124.10
124.06
123.94
123.91
122.61
122.60
111.01
110.98
110.81
110.77
108.14
103.87
103.61
103.36
57.47
45.01
37.16
35.72
31.55
31.53
f1 (ppm)

97

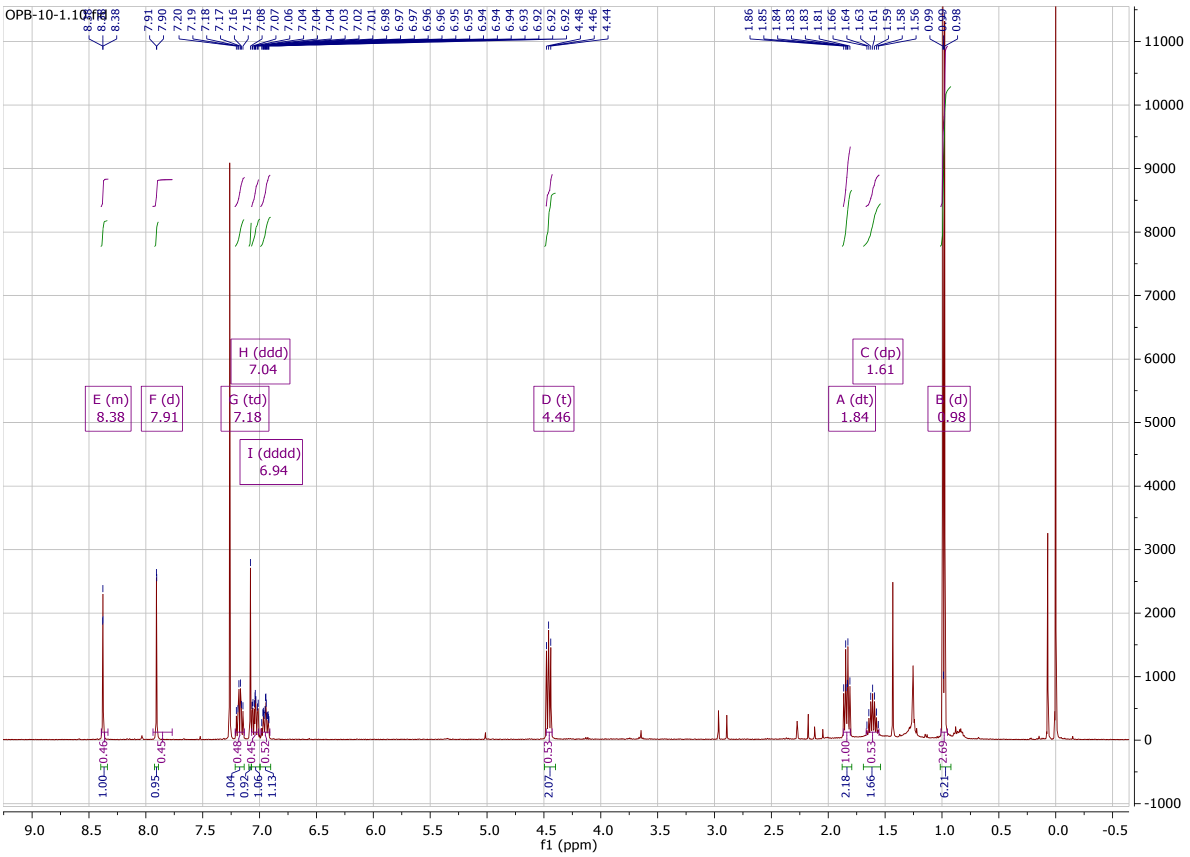

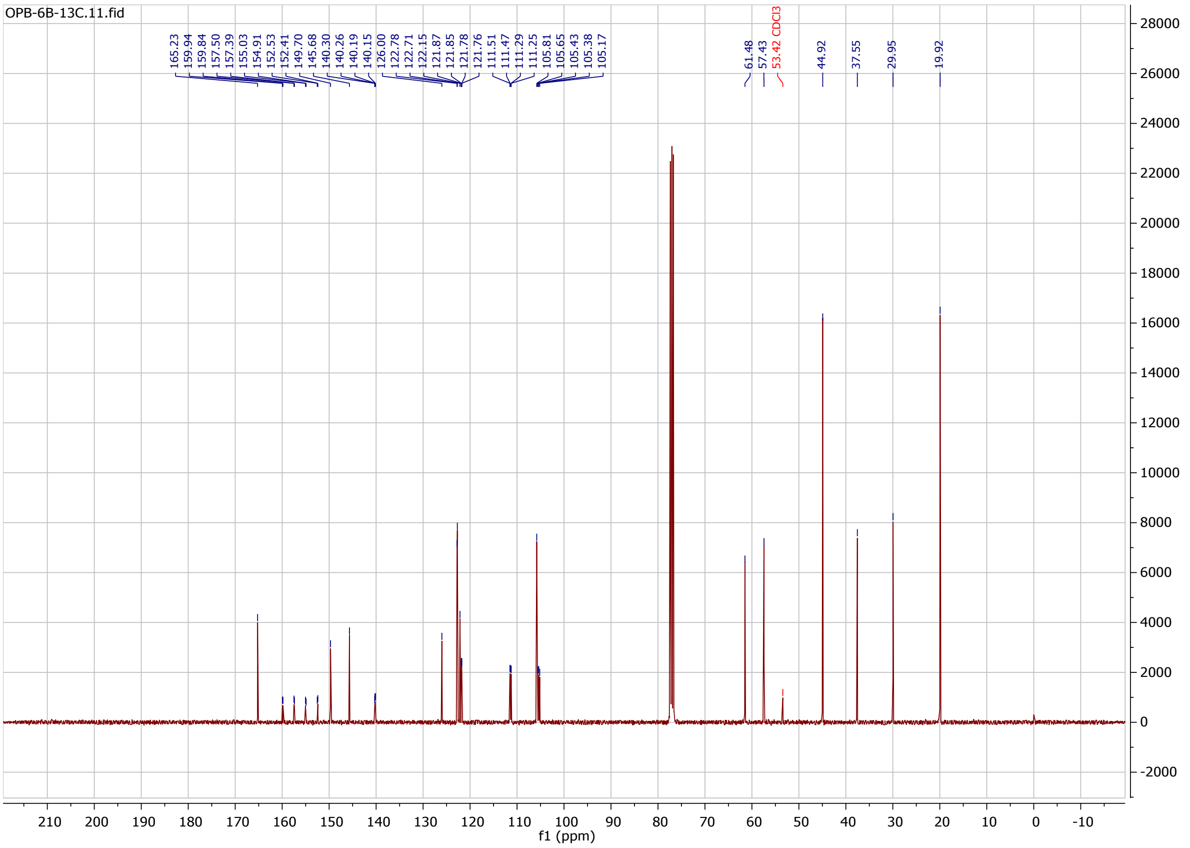

Note: The additional signal at 53.42 ppm corresponds to the residual DCM after solvent evaporation.

98

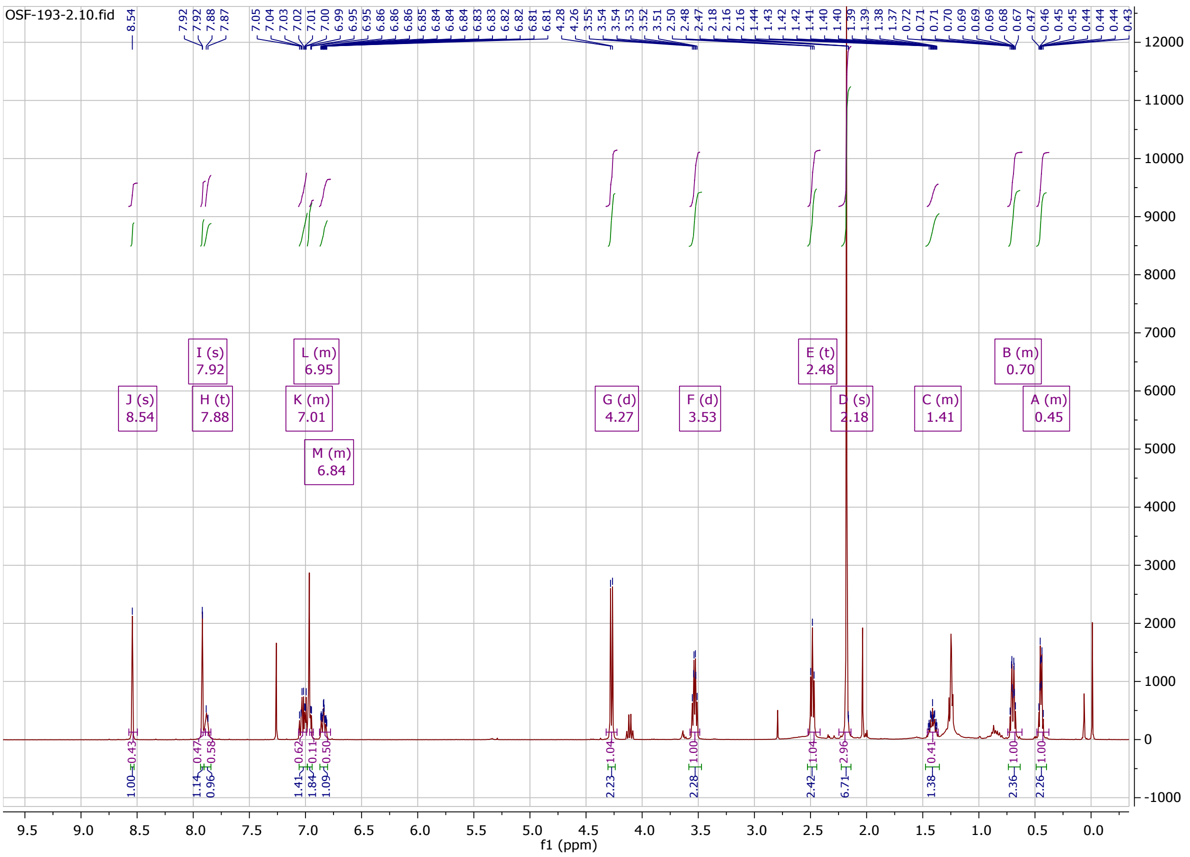

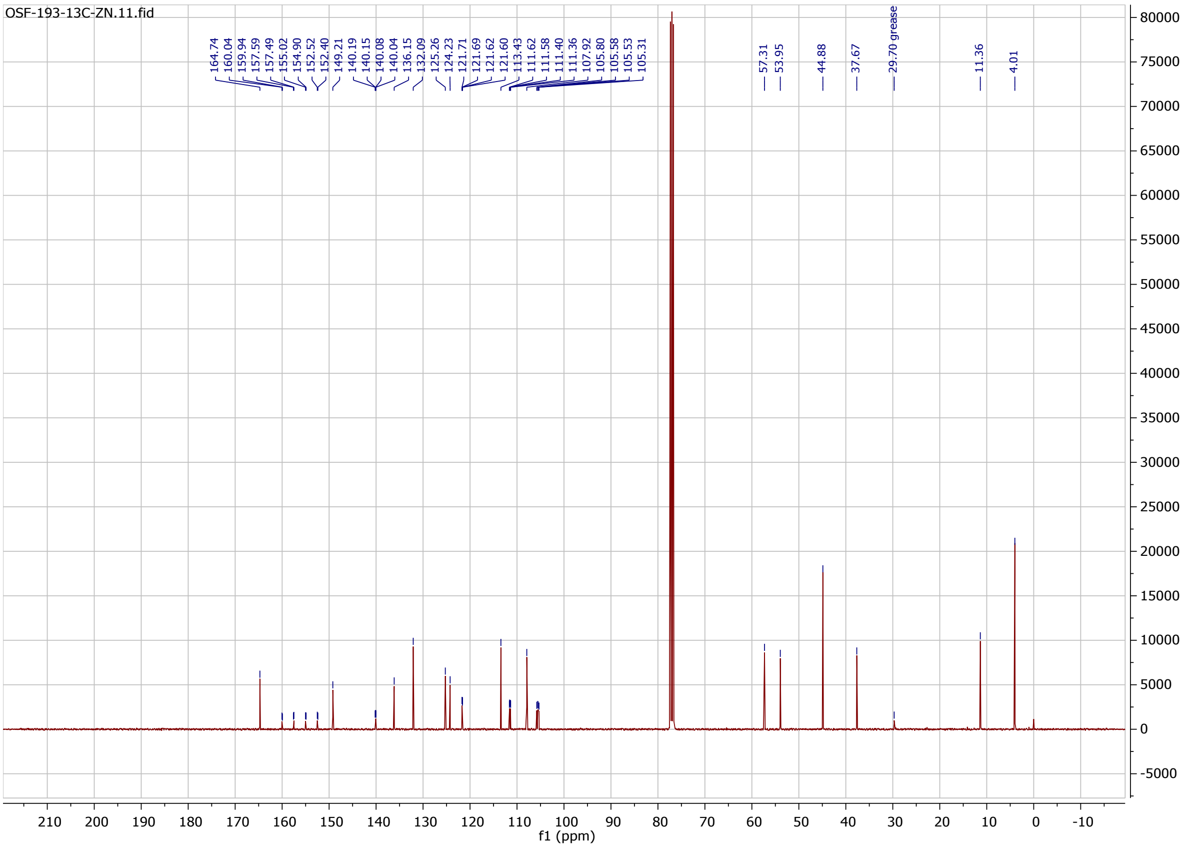

Note: The additional signal at 29.70 corresponds to and is labelled as grease.

99

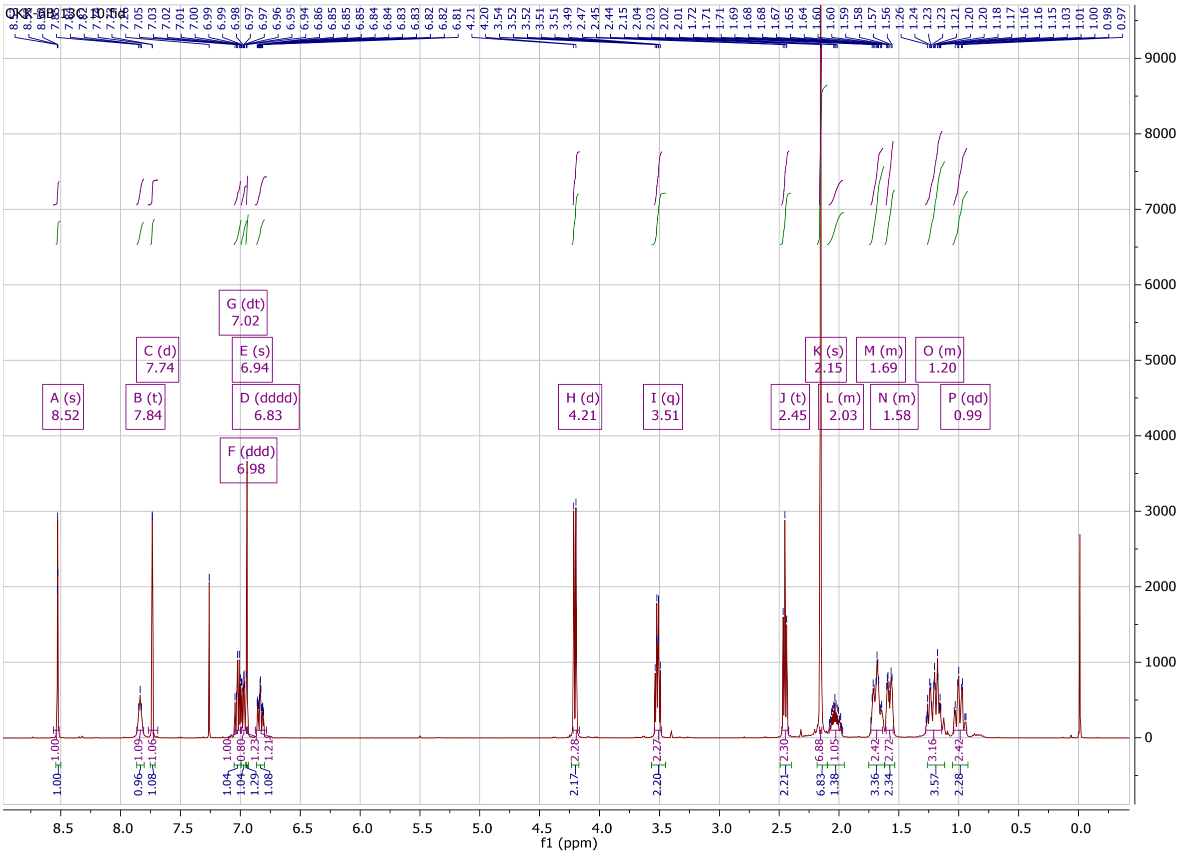
OKK-6B-13C.10.fid
A (s) 8.52
B (t) 7.84
C (d) 7.74
D (dddd) 6.83
E (s) 6.94
F (ddd) 6.98
G (dt) 7.02
H (d) 4.21
I (q) 3.51
J (t) 2.45
K (s) 2.15
L (m) 2.03
M (m) 1.69
N (m) 1.58
O (m) 1.20
P (qd) 0.99
f1 (ppm)

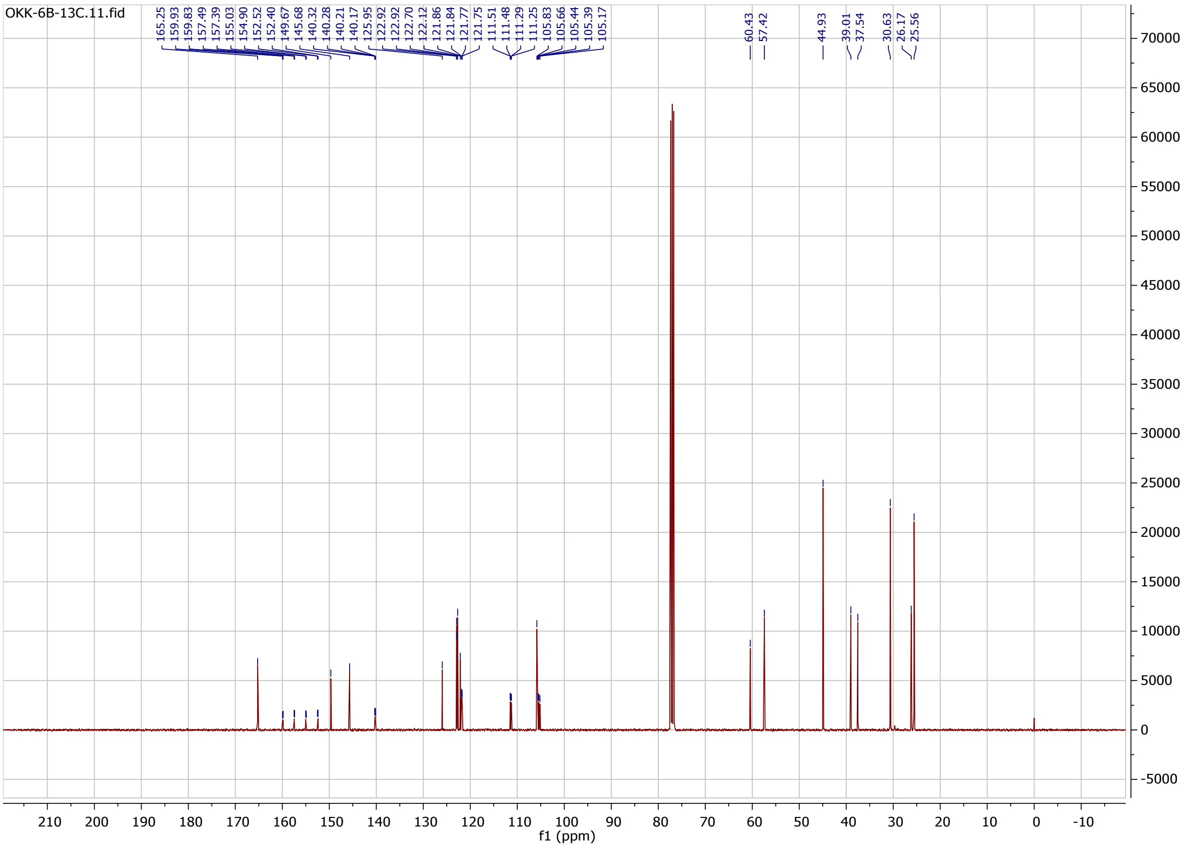
OKK-6B-13C.11.fid
165.25
159.93
159.83
157.49
157.39
155.03
154.90
152.52
152.40
149.67
145.68
140.32
140.28
140.21
140.17
125.95
122.92
122.92
122.70
122.12
121.86
121.84
121.77
121.75
111.51
111.48
111.29
111.25
105.83
105.66
105.44
105.39
105.17
60.43
57.42
44.93
39.01
37.54
30.63
26.17
25.56
f1 (ppm)

100

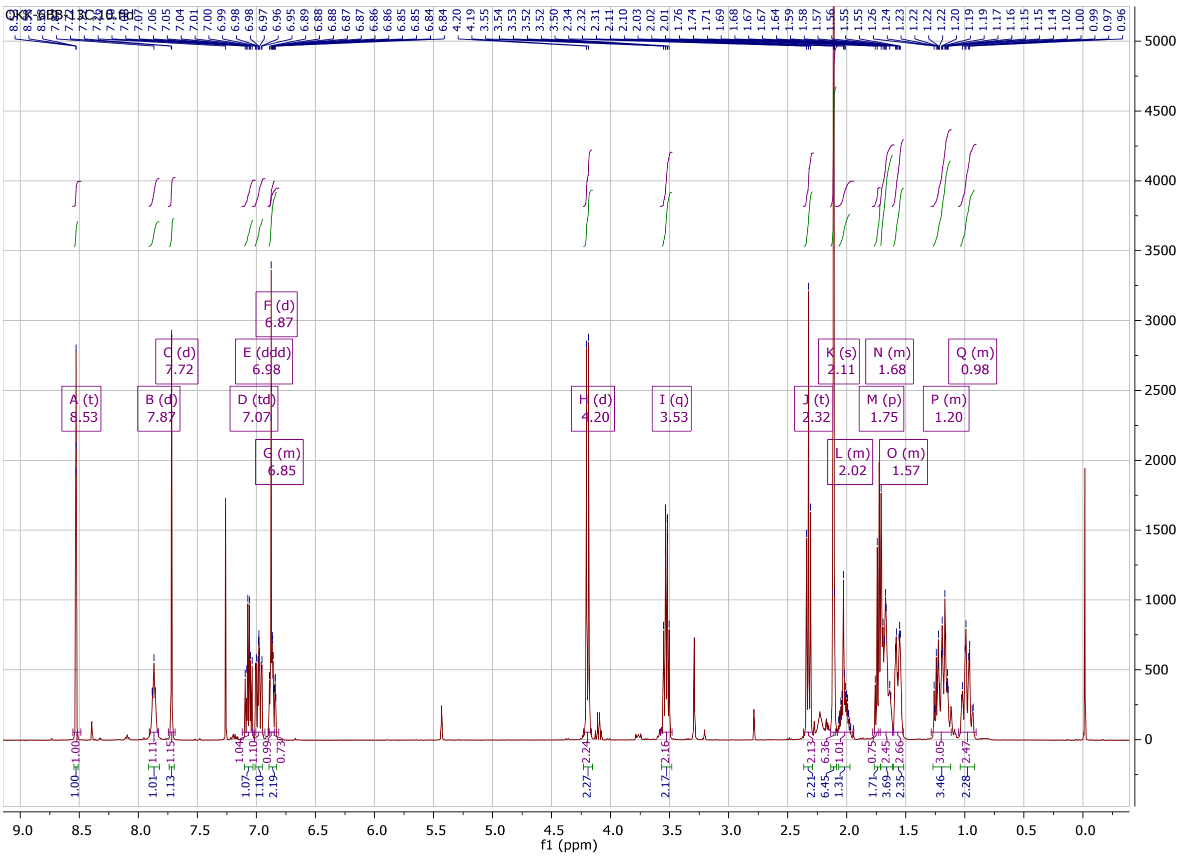

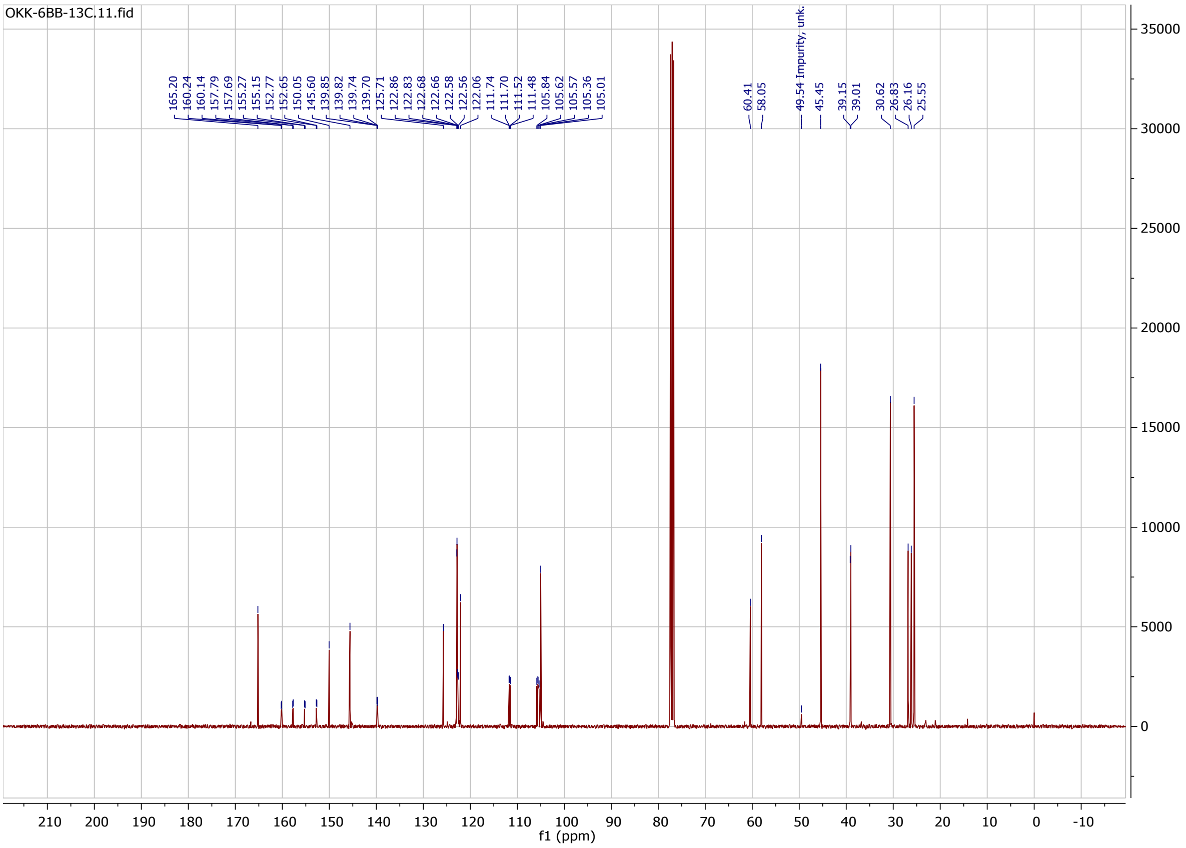

Note: The additional peak at 49.54 corresponds to and is labelled as an unknown impurity.

101

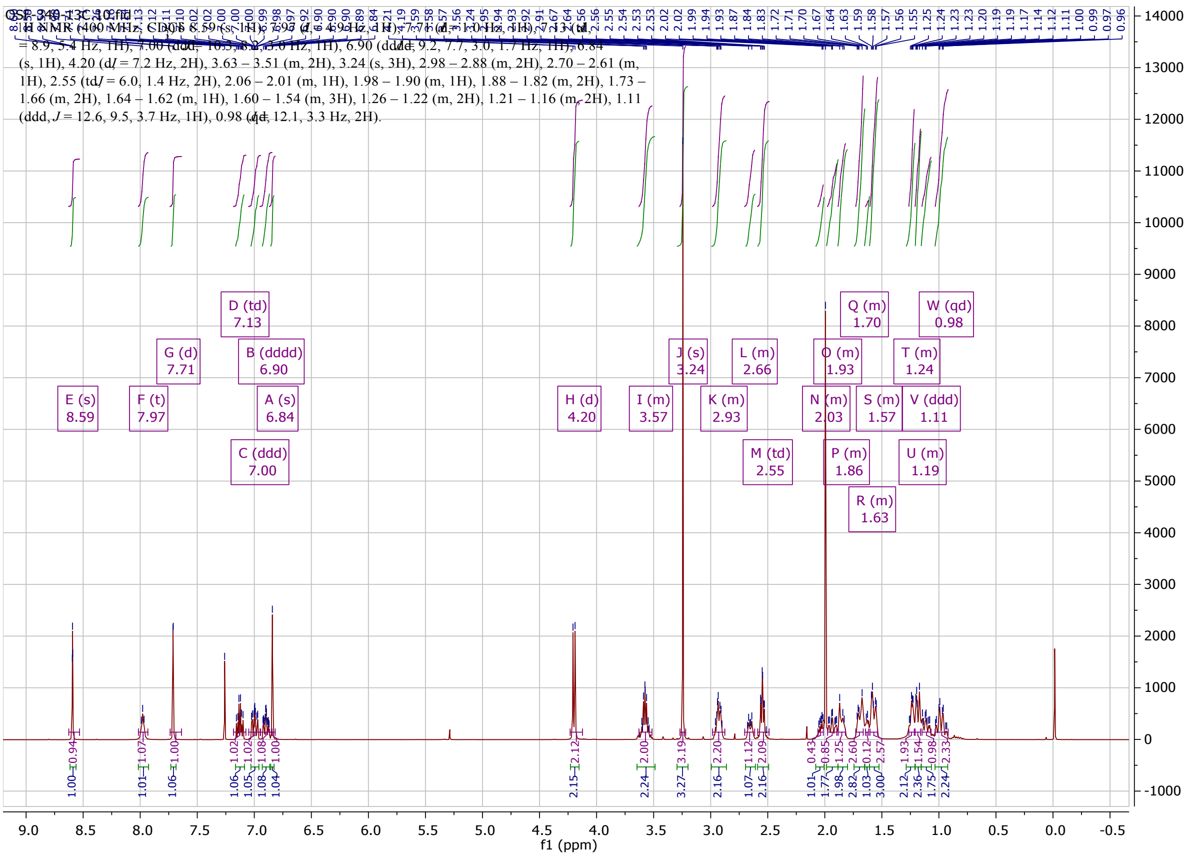

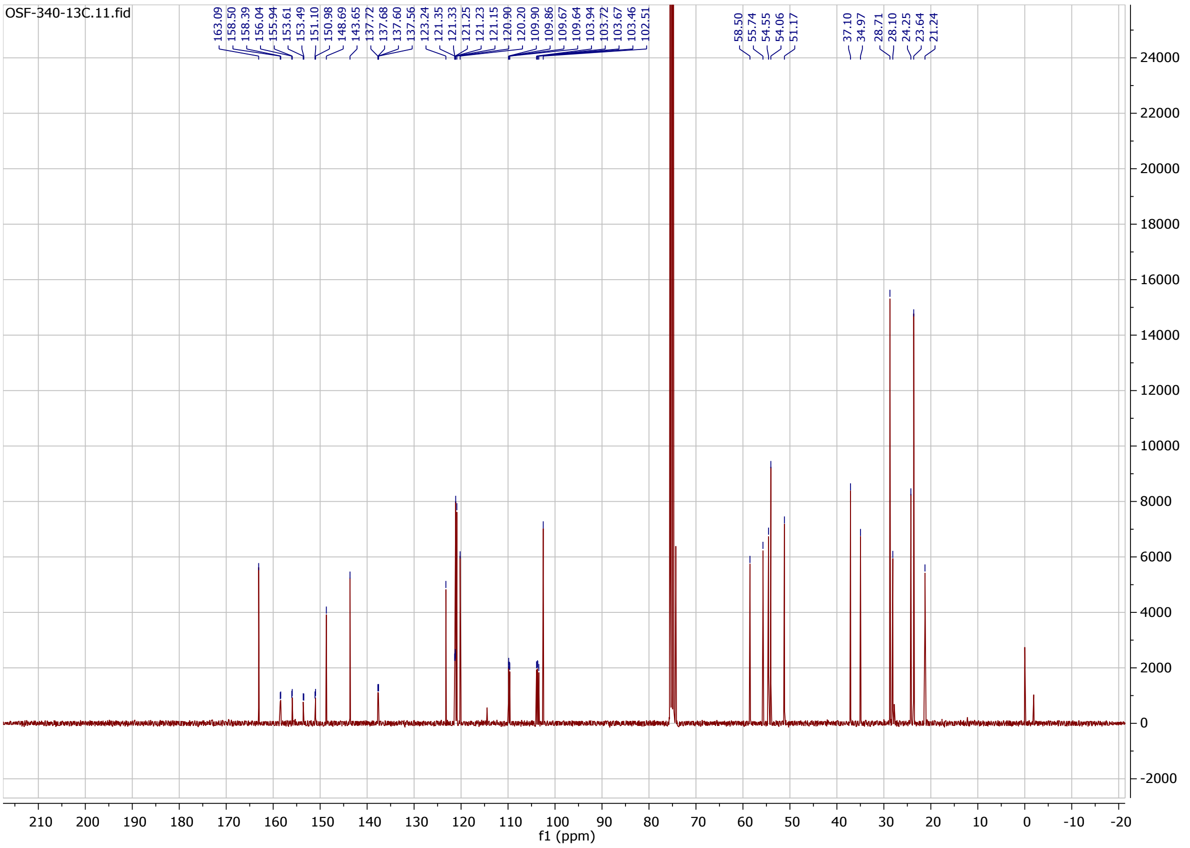

102

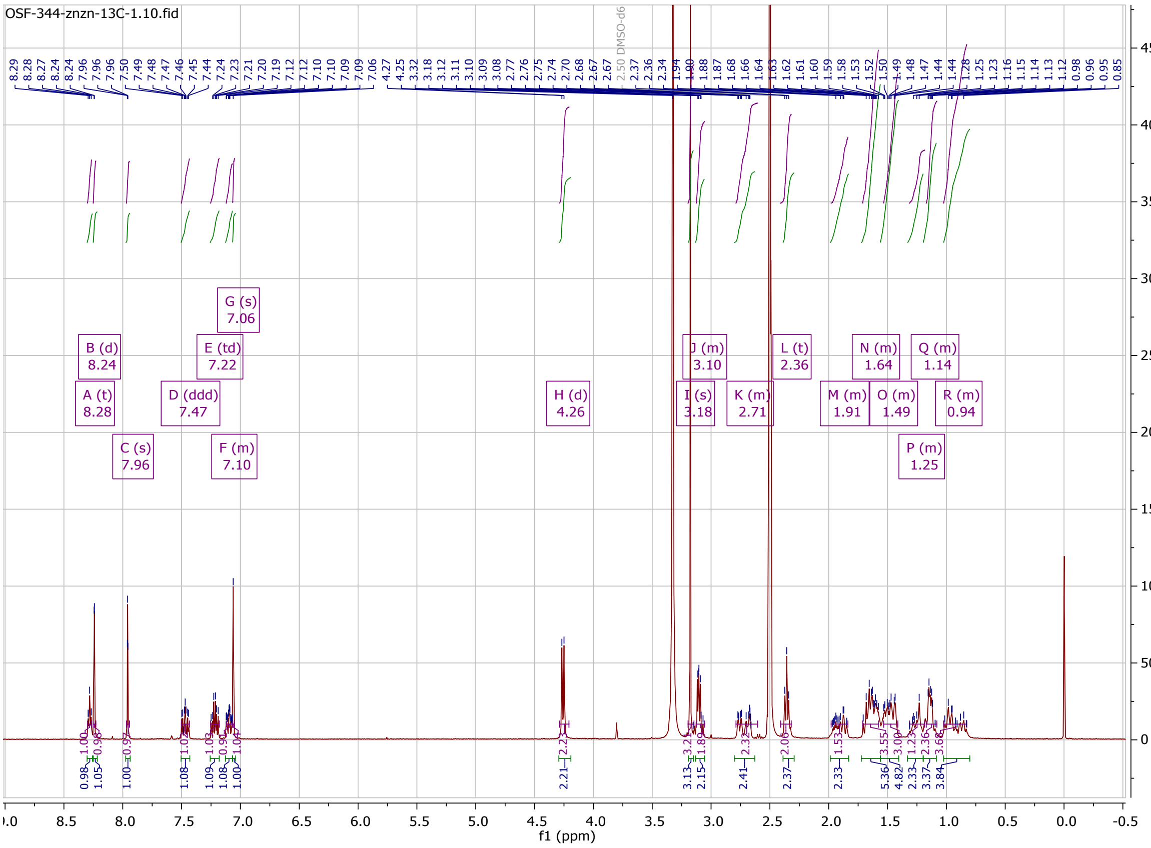

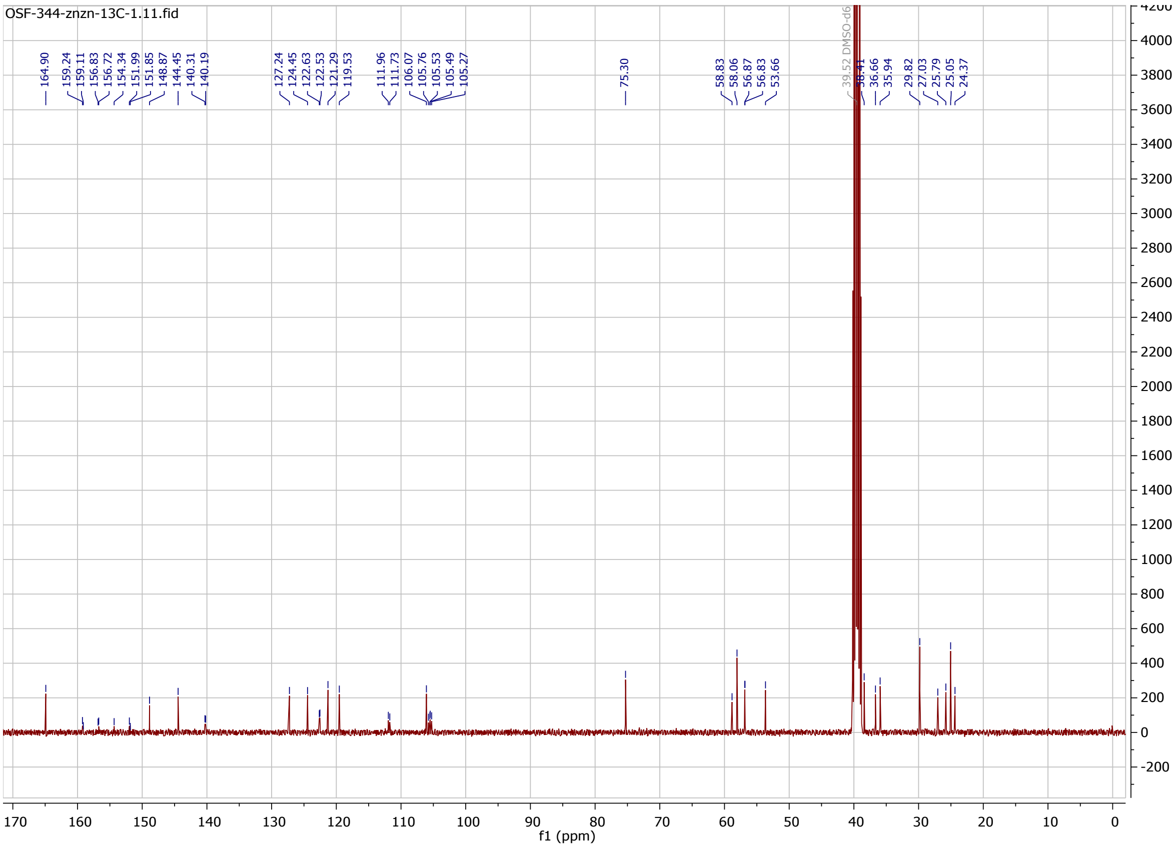

103

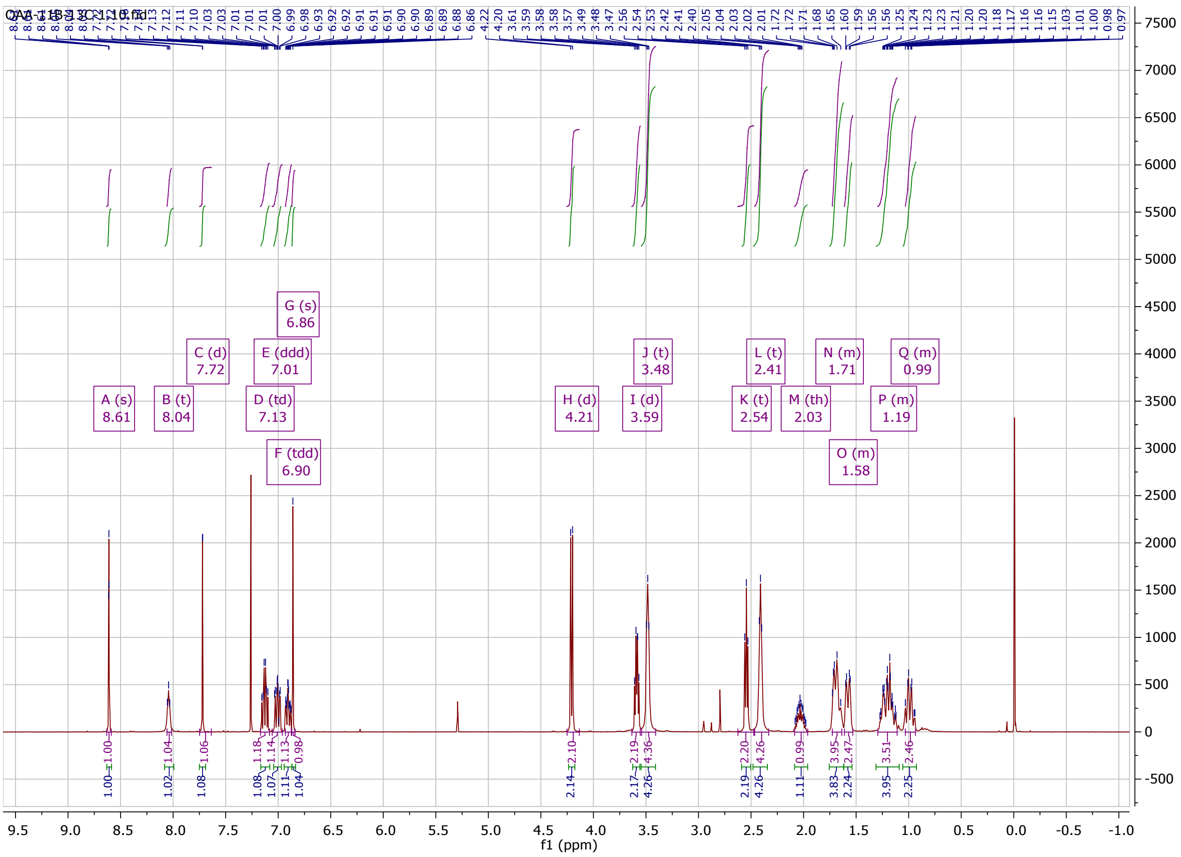

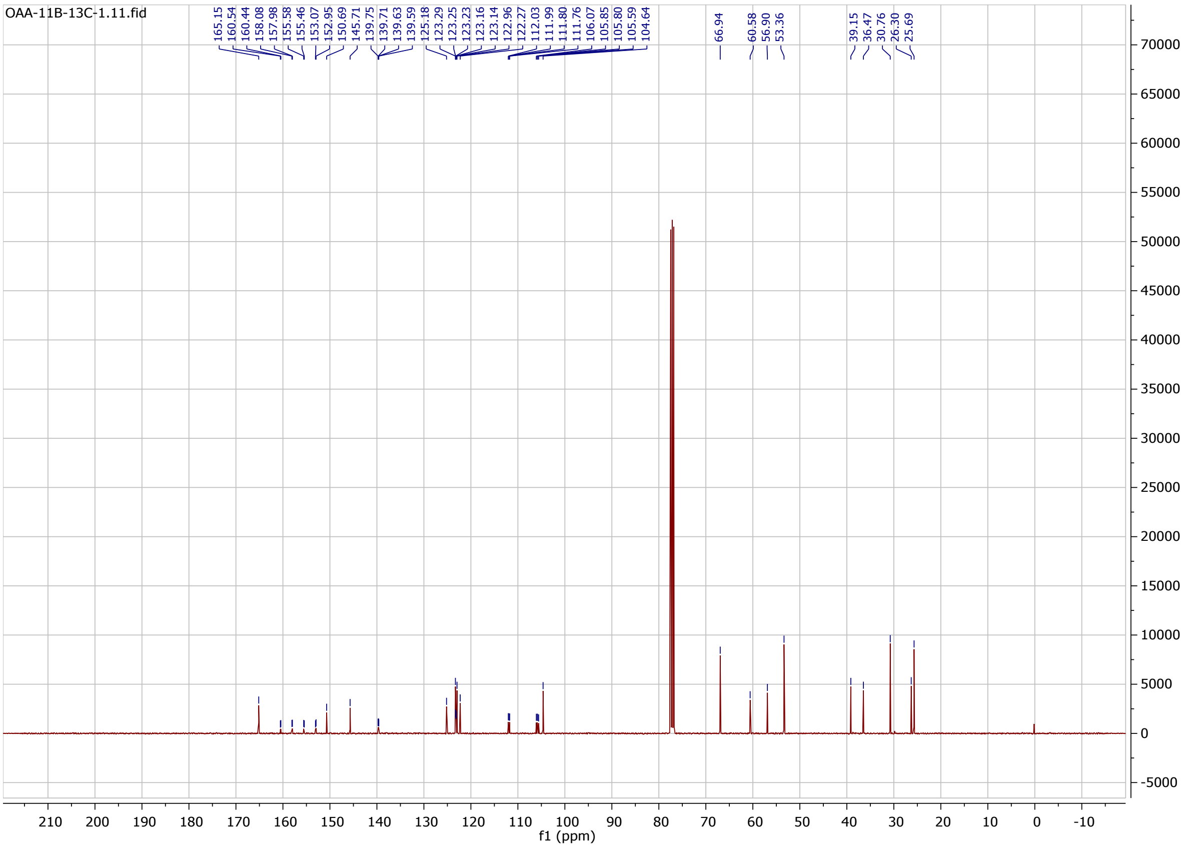

104

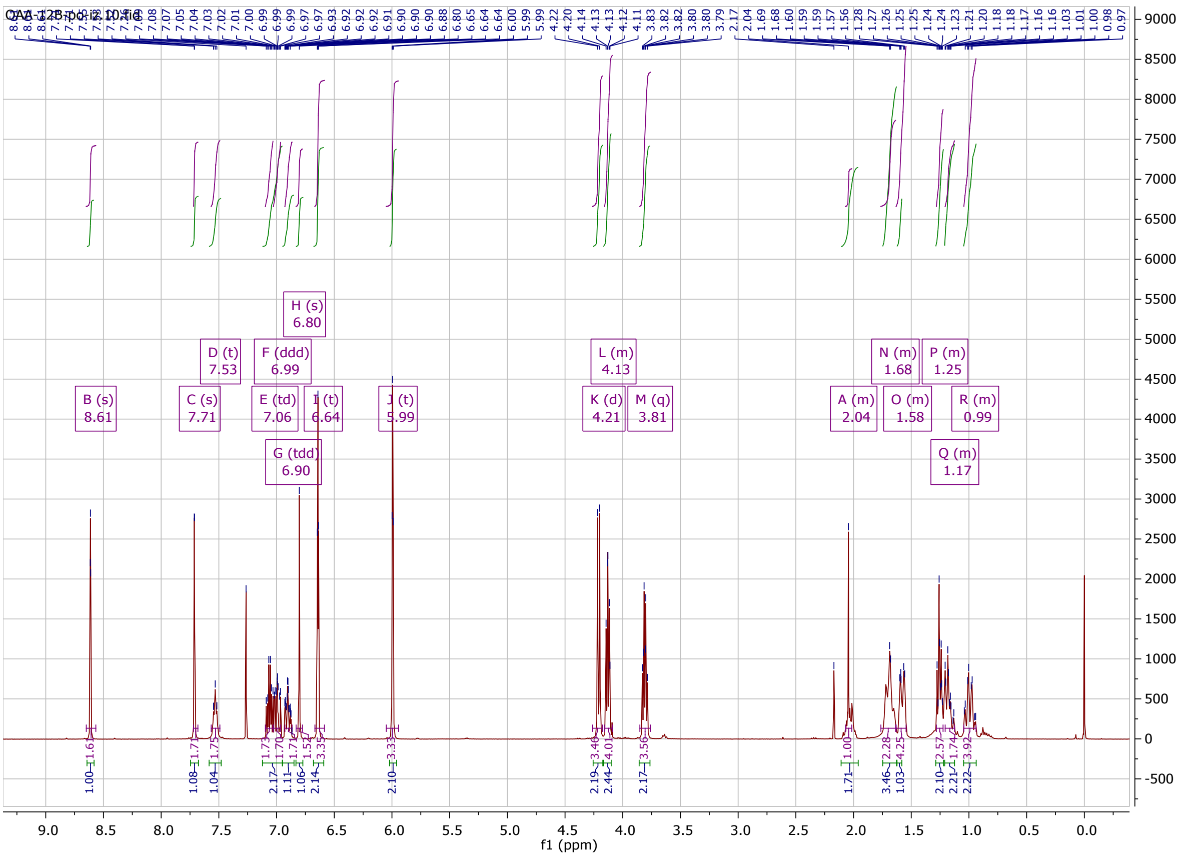

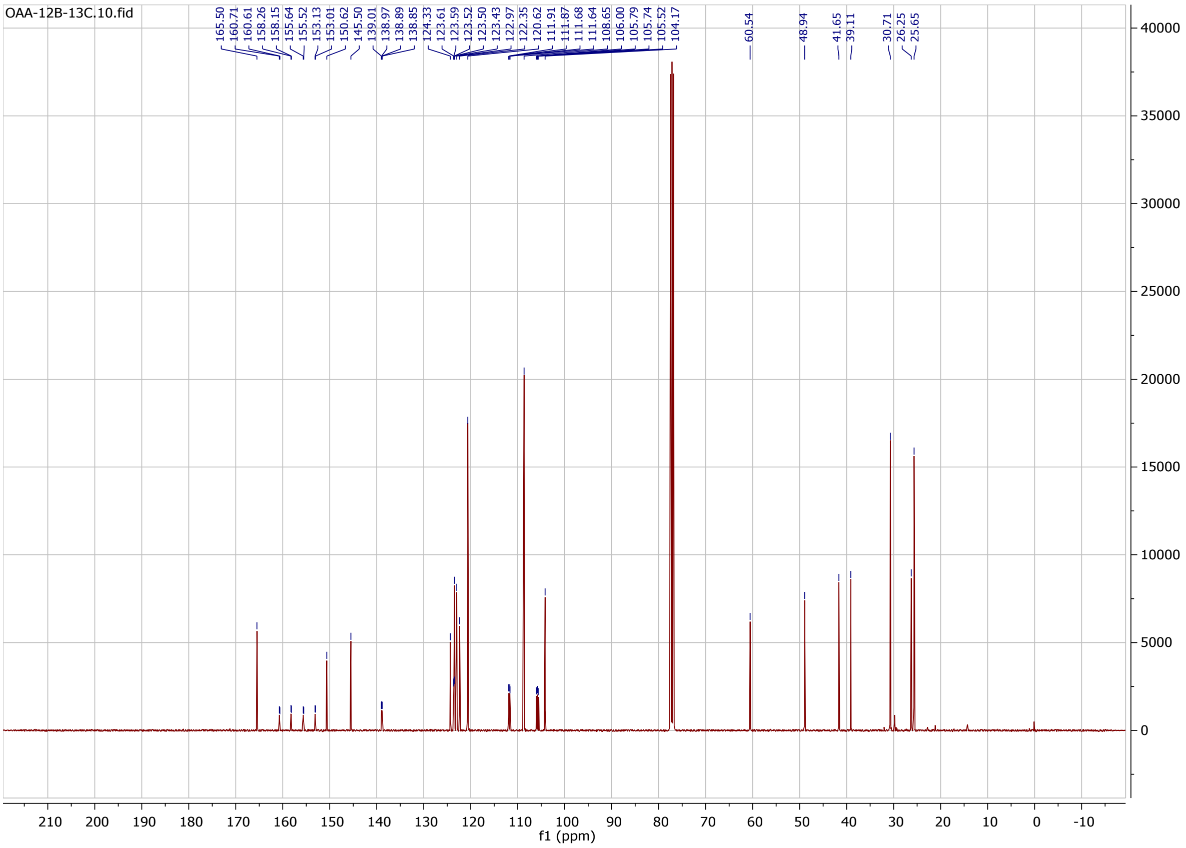

105

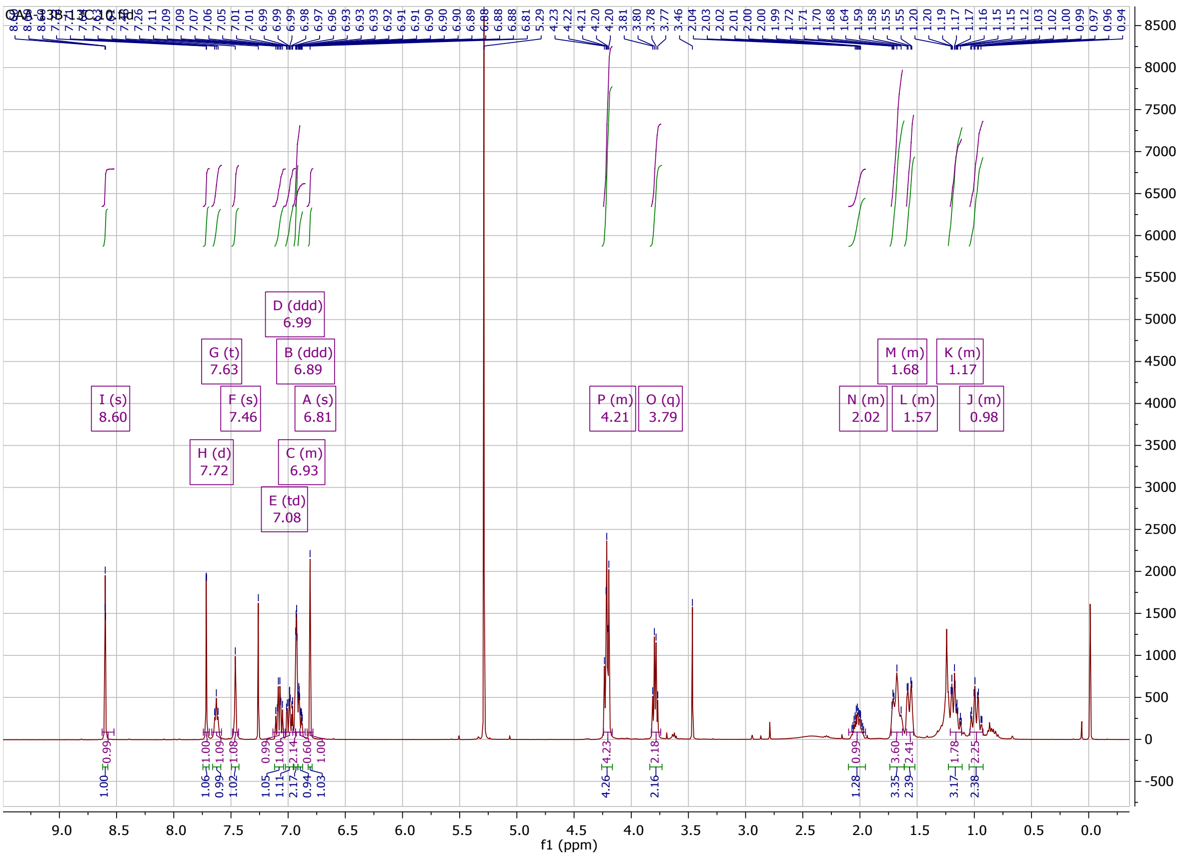
OAA-13B-13C.10.fid
I (s) 8.60
G (t) 7.63
H (d) 7.72
F (s) 7.46
D (ddd) 6.99
B (ddd) 6.89
A (s) 6.81
C (m) 6.93
E (td) 7.08
P (m) 4.21
O (q) 3.79
N (m) 2.02
M (m) 1.68
L (m) 1.57
K (m) 1.17
J (m) 0.98
f1 (ppm)

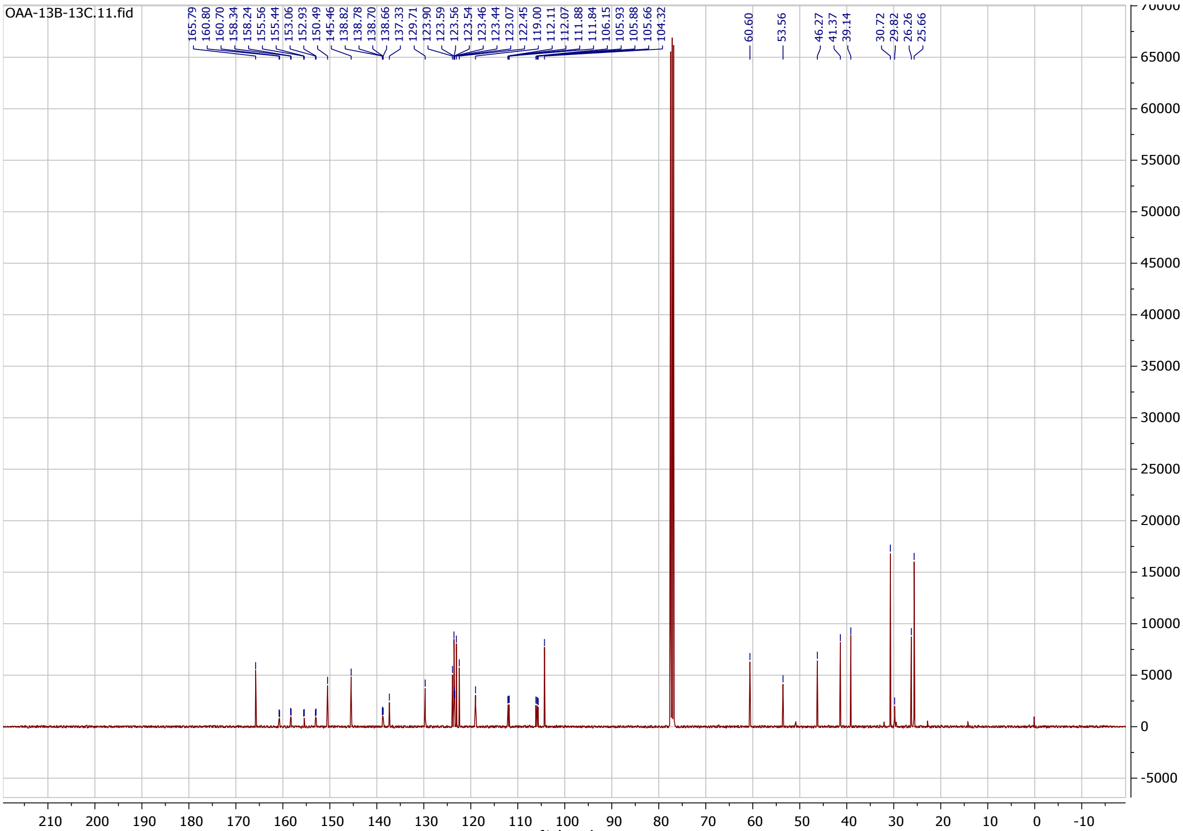
OAA-13B-13C.11.fid
165.79
160.80
160.70
158.34
158.24
155.56
155.44
153.06
152.93
150.49
145.46
138.82
138.78
138.70
138.66
137.33
129.71
123.90
123.59
123.56
123.54
123.46
123.44
123.07
122.45
119.00
112.11
112.07
111.88
111.84
106.15
105.93
105.88
105.66
104.32
60.60
53.56
46.27
41.37
39.14
30.72
29.82
26.26
25.66
f1 (ppm)

106

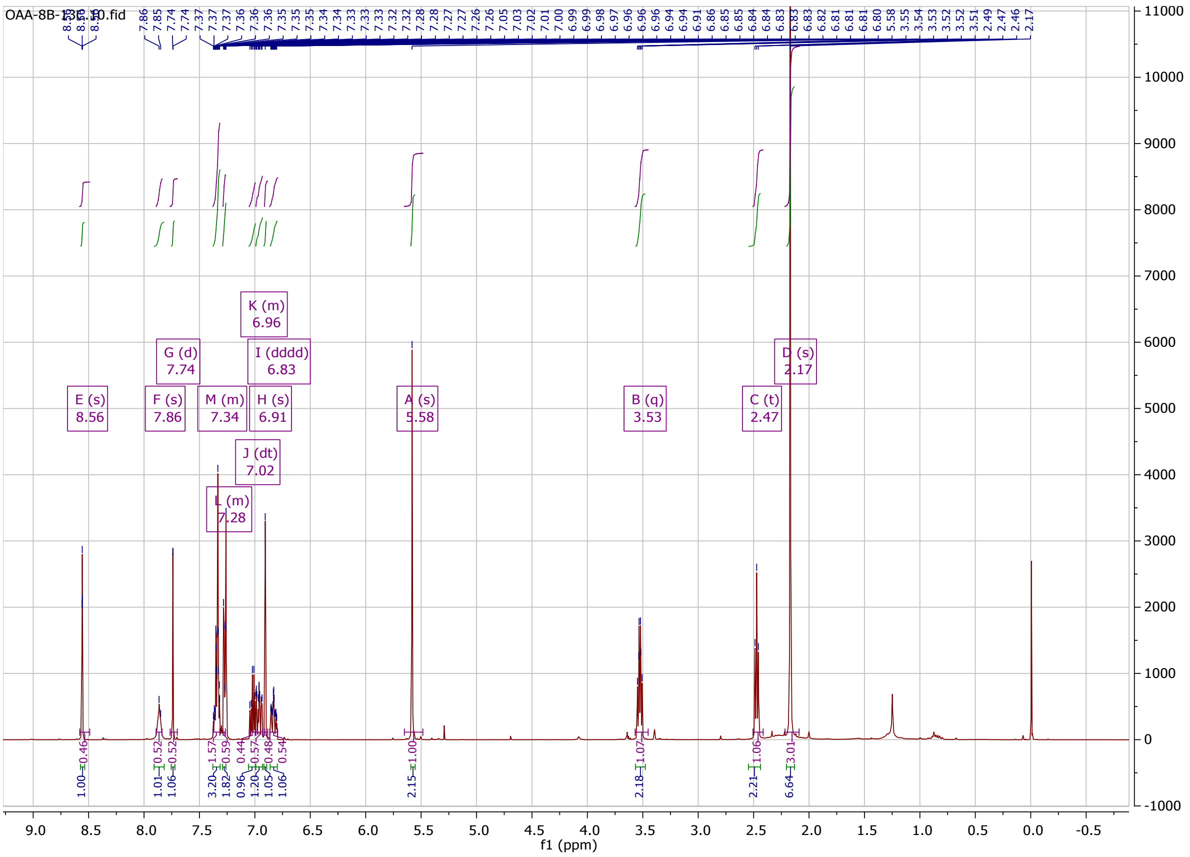

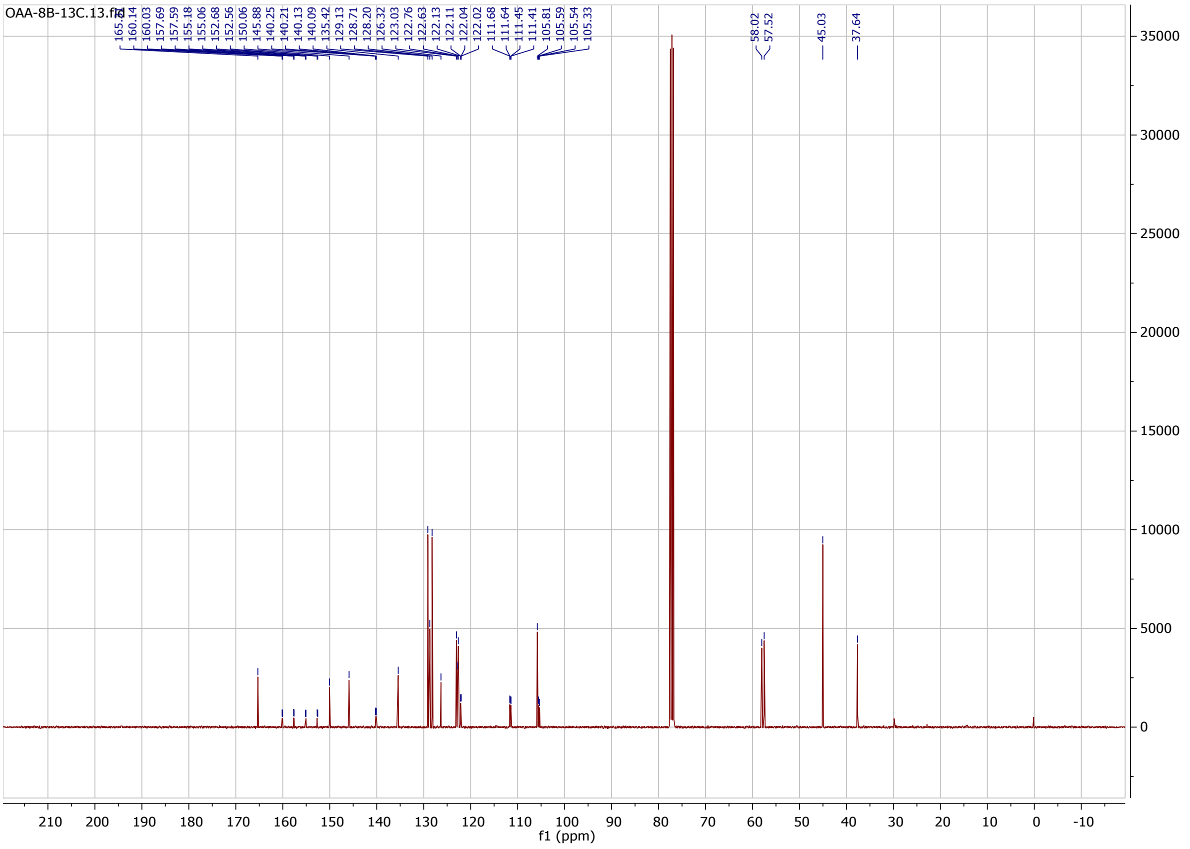

107

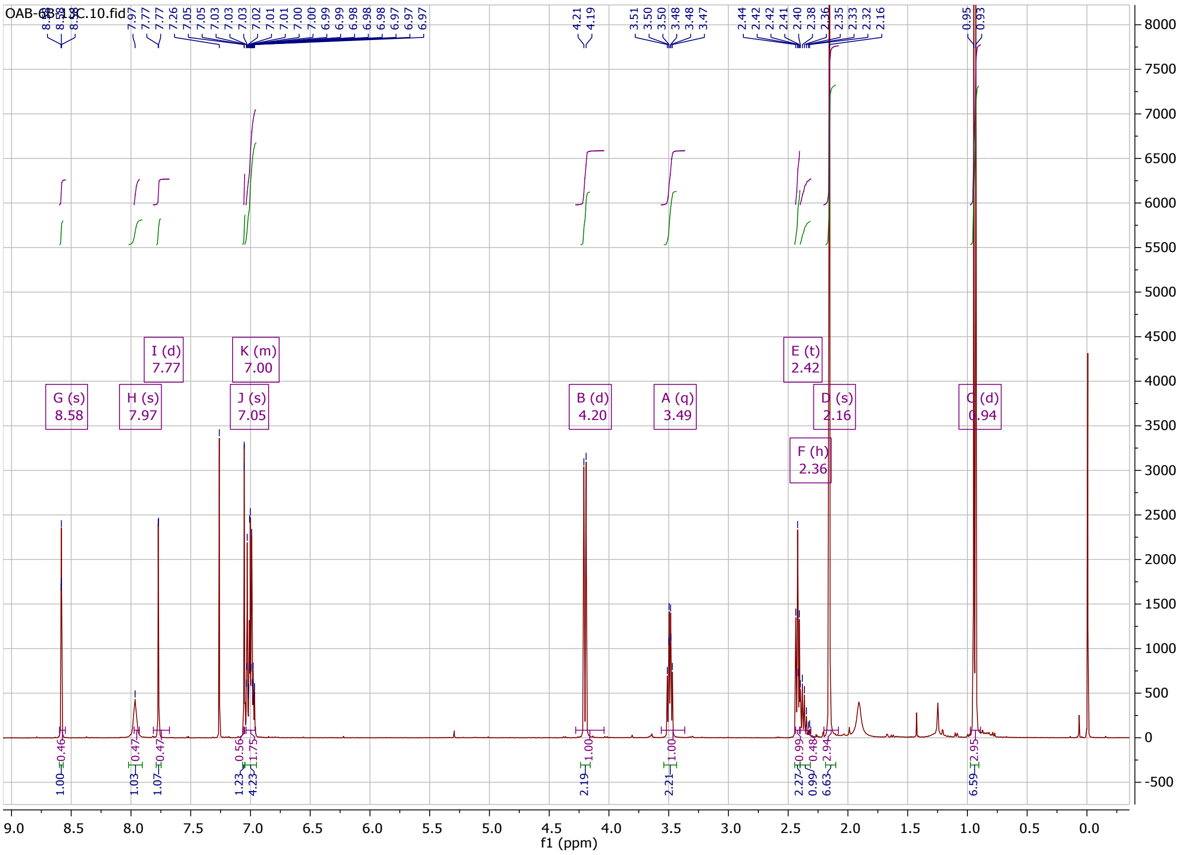
OAB-6B-13C.10.fid
G (s)
8.58
H (s)
7.97
I (d)
7.77
J (s)
7.05
K (m)
7.00
B (d)
4.20
A (q)
3.49
E (t)
2.42
D (s)
2.16
F (h)
2.36
C (d)
0.94
f1 (ppm)

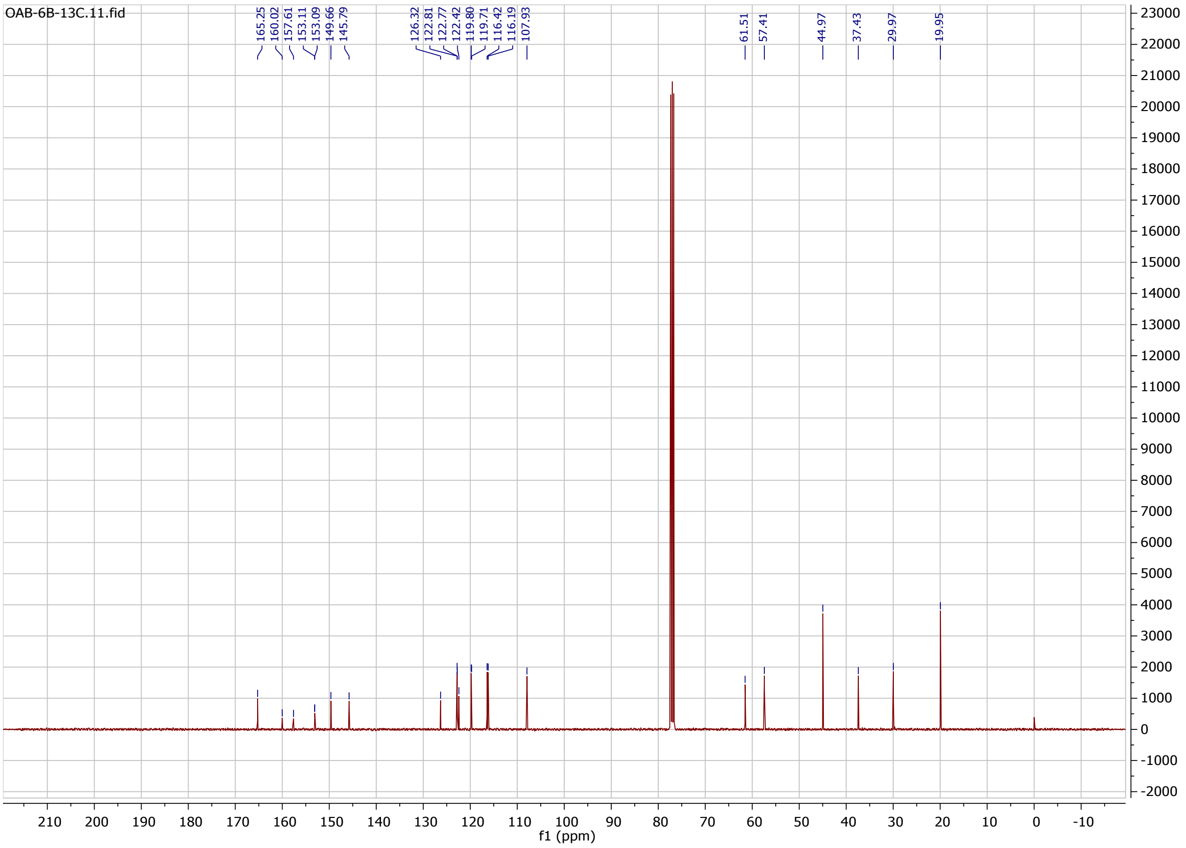
OAB-6B-13C.11.fid
165.25
160.02
157.61
153.11
153.09
149.66
145.79
126.32
122.81
122.77
122.42
119.80
119.71
116.42
116.19
107.93
61.51
57.41
44.97
37.43
29.97
19.95
f1 (ppm)

108

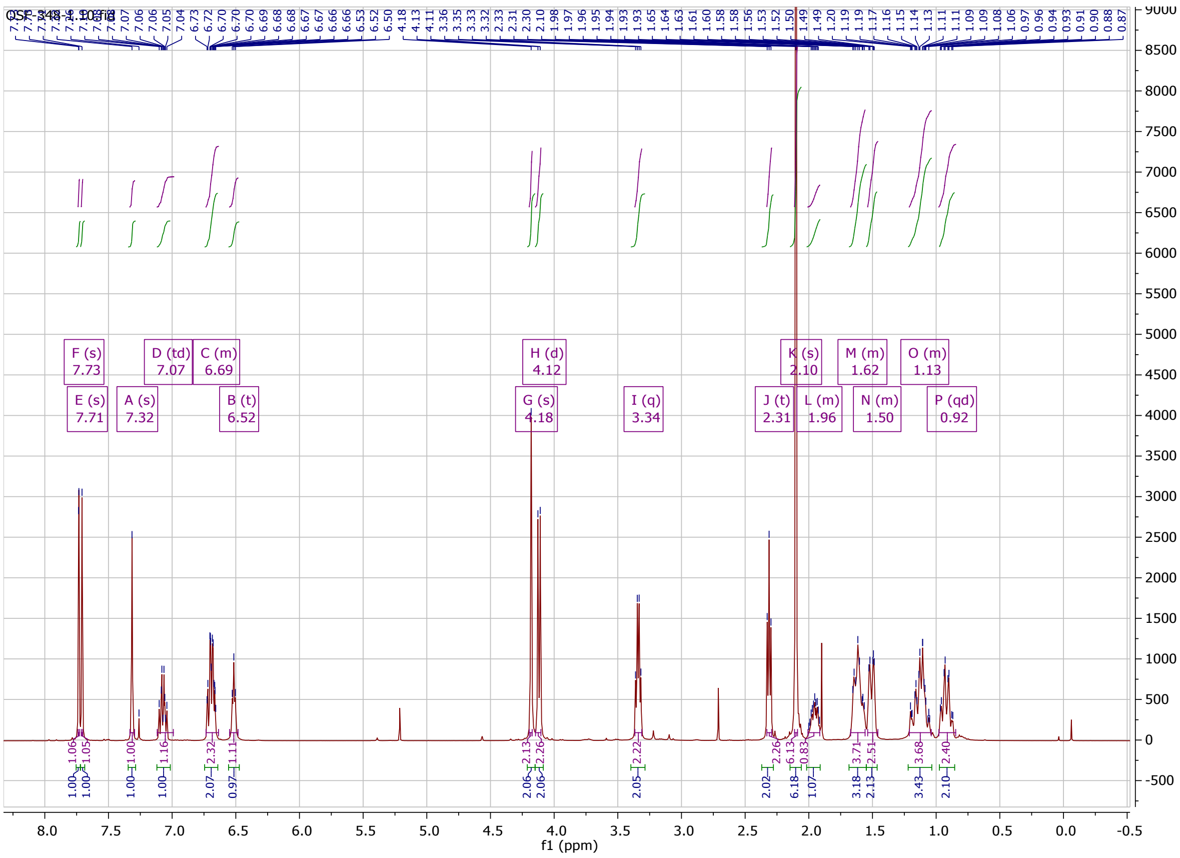

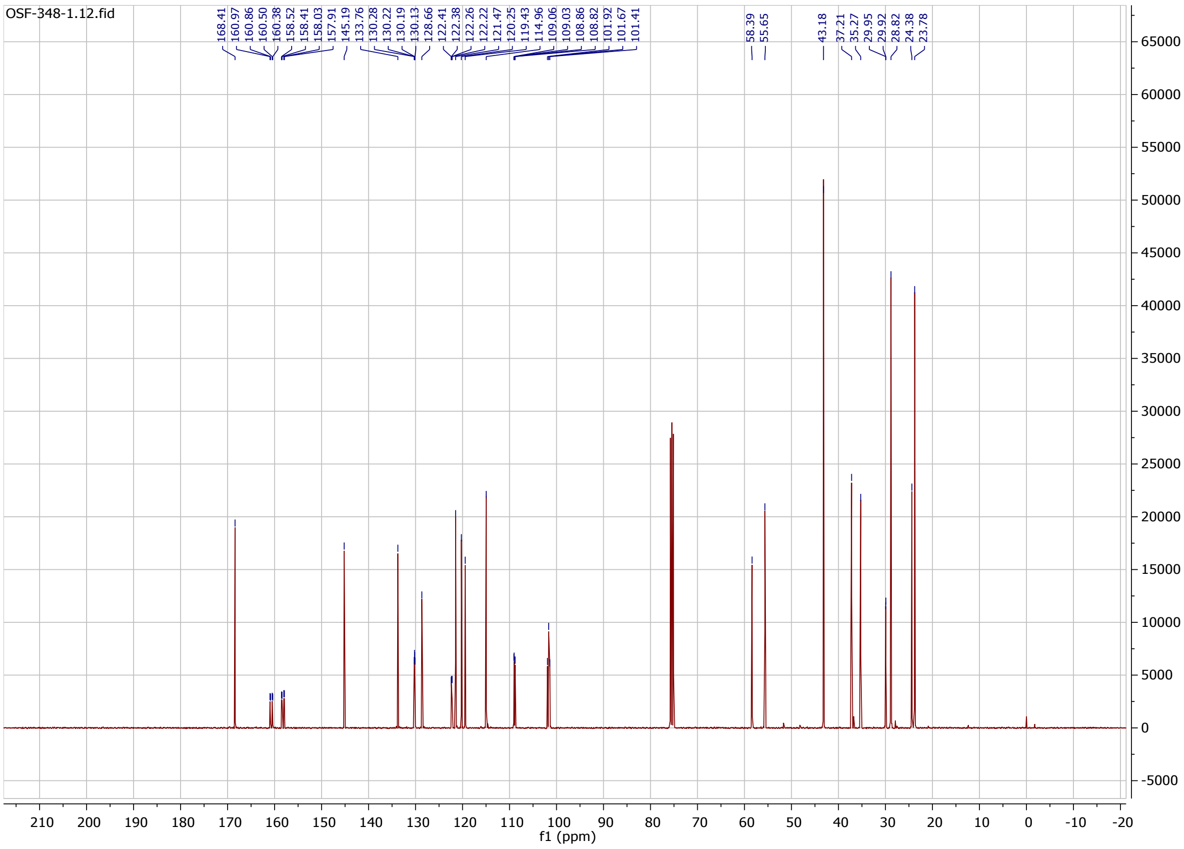

109

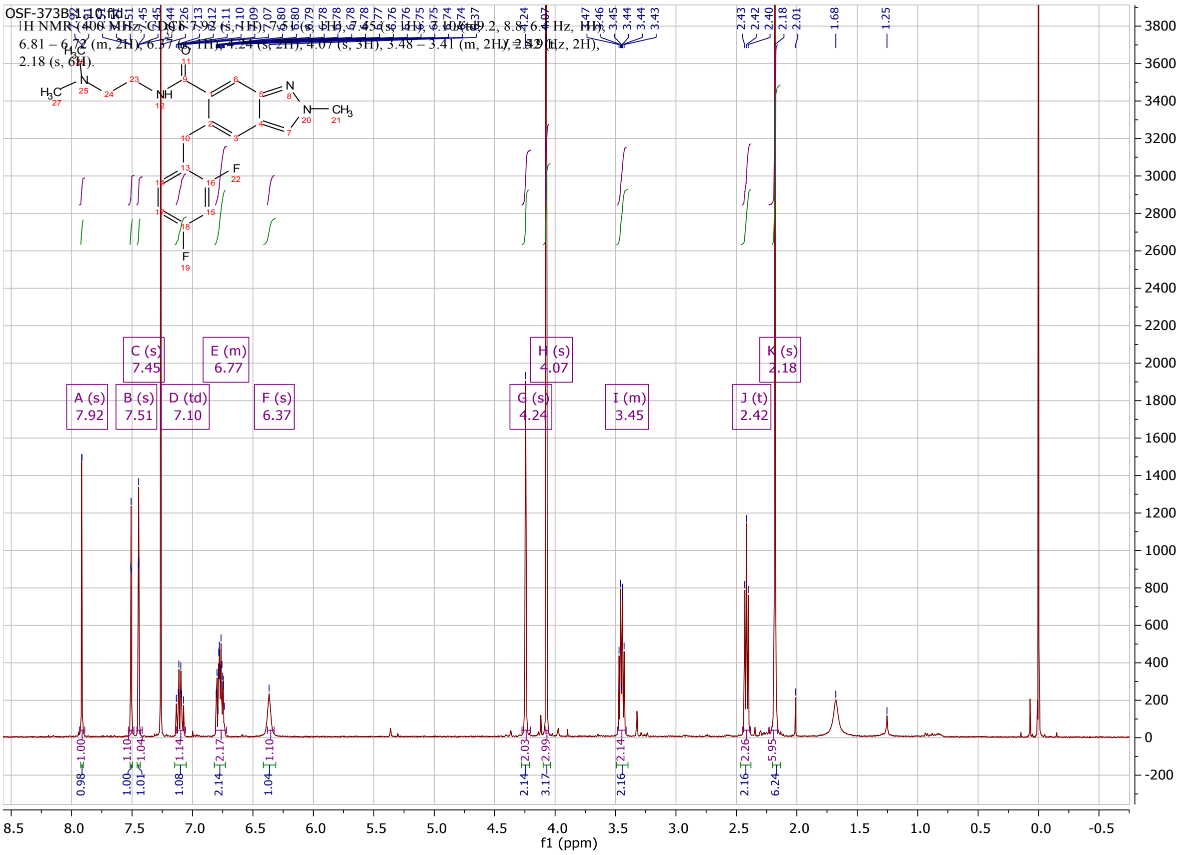
OSF-373B-1.10.fid
A (s) 7.92
B (s) 7.51
C (s) 7.45
D (td) 7.10
E (m) 6.77
F (s) 6.37
G (s) 4.24
H (s) 4.07
I (m) 3.45
J (t) 2.42
K (s) 2.18
f1 (ppm)

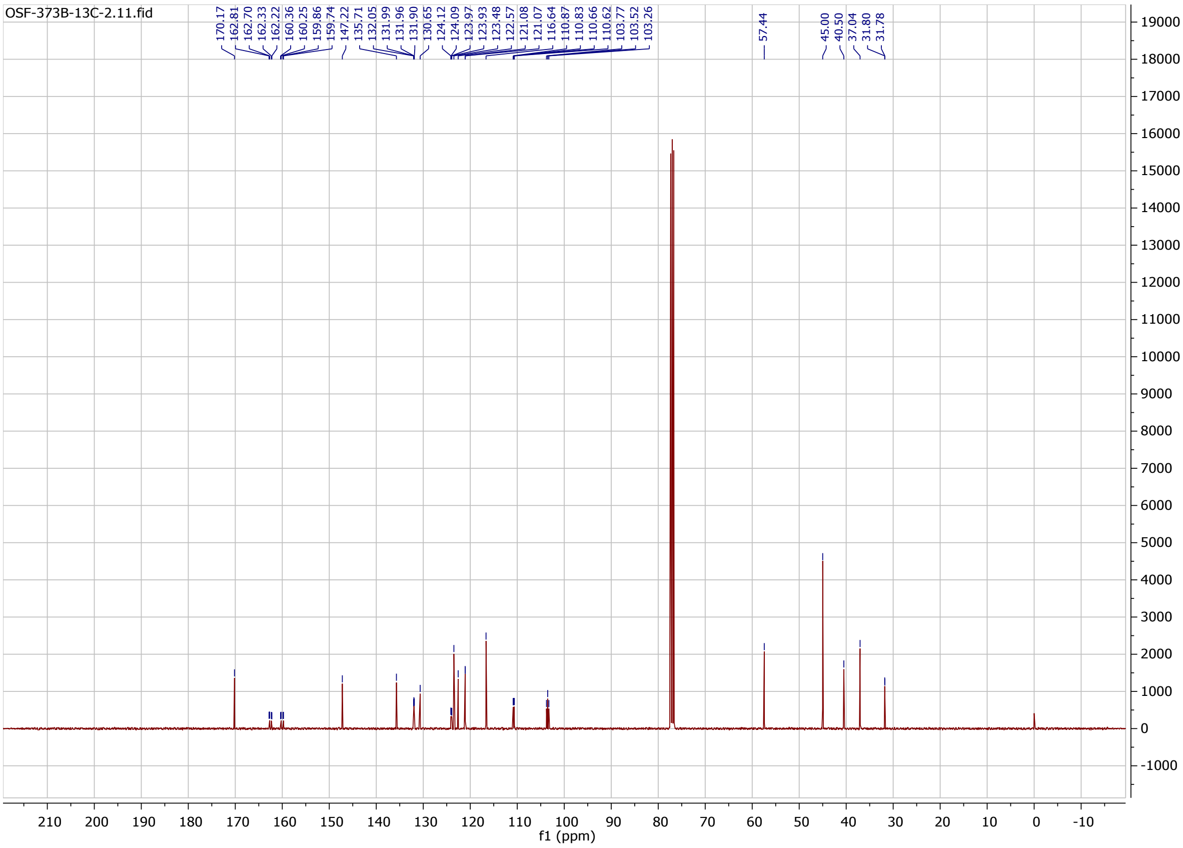
OSF-373B-13C-2.11.fid
170.17
162.81
162.70
162.33
162.22
160.36
160.25
159.86
159.74
147.22
135.71
132.05
131.99
131.96
131.90
130.65
124.12
124.09
123.97
123.93
123.48
122.57
121.08
121.07
116.64
110.87
110.83
110.66
110.62
103.77
103.52
103.26
57.44
45.00
40.50
37.04
31.80
31.78
f1 (ppm)

Supplementary References

(1) Ellman, G. L.; Courtney, K. D.; Andres, V.; Feather-Stone, R. M. A New and Rapid Colorimetric Determination of Acetylcholinesterase Activity. *Biochem Pharmacol* **1961**, *7*, 88–95. https://doi.org/10.1016/0006-2952(61)90145-9.

(2) Zegzouti, H.; Zdanovskaia, M.; Hsiao, K.; Goueli, S. A. ADP-Glo: A Bioluminescent and Homogeneous ADP Monitoring Assay for Kinases. *Assay Drug Dev Technol* **2009**, *7* (6), 560–572. https://doi.org/10.1089/adt.2009.0222.

(3) Wright, D.; Winski, S. L.; Anderson, D.; Lee, P.; Munson, M.; Winkler, J. ARRY-797, a Potent and Selective Inhibitor of P38 Map Kinase, Inhibits LPS-Induced IL-6 and In Vivo Growth of RPMI-8226 Human Multiple Myeloma Cells. *Blood* **2006**, *108* (11), 3478. https://doi.org/10.1182/blood.V108.11.3478.3478.

(4) Sun, J.; Zhang, X.; Broderick, M.; Fein, H. Measurement of Nitric Oxide Production in Biological Systems by Using Griess Reaction Assay. *Sensors* **2003**, *3* (8), 276–284. https://doi.org/10.3390/s30800276.
